# The genomes of 5 mantises provide insights into sex chromosome evolution and Mantodea phylogeny clarification

**DOI:** 10.1093/gigascience/giaf158

**Published:** 2025-12-18

**Authors:** Hangwei Liu, Lihong Lei, Fan Jiang, Bo Zhang, Hengchao Wang, Yutong Zhang, Hanbo Zhao, Guirong Wang, Wei Fan

**Affiliations:** Guangdong Laboratory for Lingnan Modern Agriculture (Shenzhen Branch), Genome Analysis Laboratory of the Ministry of Agriculture and Rural Affairs, Agricultural Genomics Institute at Shenzhen, Chinese Academy of Agricultural Sciences, Shenzhen, Guangdong 518120, China; Department of Entomology, College of Plant Protection, Yangzhou University, Yangzhou 225009, China; Guangdong Laboratory for Lingnan Modern Agriculture (Shenzhen Branch), Genome Analysis Laboratory of the Ministry of Agriculture and Rural Affairs, Agricultural Genomics Institute at Shenzhen, Chinese Academy of Agricultural Sciences, Shenzhen, Guangdong 518120, China; State Key Laboratory of Crop Stress Adaptation and Improvement, School of Life Sciences, Henan University, Kaifeng 475004, China; Shenzhen Research Institute of Henan University, Shenzhen 518000, China; Guangdong Laboratory for Lingnan Modern Agriculture (Shenzhen Branch), Genome Analysis Laboratory of the Ministry of Agriculture and Rural Affairs, Agricultural Genomics Institute at Shenzhen, Chinese Academy of Agricultural Sciences, Shenzhen, Guangdong 518120, China; Guangdong Laboratory for Lingnan Modern Agriculture (Shenzhen Branch), Genome Analysis Laboratory of the Ministry of Agriculture and Rural Affairs, Agricultural Genomics Institute at Shenzhen, Chinese Academy of Agricultural Sciences, Shenzhen, Guangdong 518120, China; Guangdong Laboratory for Lingnan Modern Agriculture (Shenzhen Branch), Genome Analysis Laboratory of the Ministry of Agriculture and Rural Affairs, Agricultural Genomics Institute at Shenzhen, Chinese Academy of Agricultural Sciences, Shenzhen, Guangdong 518120, China; State Key Laboratory of Crop Stress Adaptation and Improvement, School of Life Sciences, Henan University, Kaifeng 475004, China; Guangdong Laboratory for Lingnan Modern Agriculture (Shenzhen Branch), Genome Analysis Laboratory of the Ministry of Agriculture and Rural Affairs, Agricultural Genomics Institute at Shenzhen, Chinese Academy of Agricultural Sciences, Shenzhen, Guangdong 518120, China; Guangdong Laboratory for Lingnan Modern Agriculture (Shenzhen Branch), Genome Analysis Laboratory of the Ministry of Agriculture and Rural Affairs, Agricultural Genomics Institute at Shenzhen, Chinese Academy of Agricultural Sciences, Shenzhen, Guangdong 518120, China; Department of Entomology, State Key Laboratory for Biology of Plant Diseases and Insect Pests, Institute of Plant Protection, Chinese Academy of Agricultural Sciences, Beijing 100081, China; Guangdong Laboratory for Lingnan Modern Agriculture (Shenzhen Branch), Genome Analysis Laboratory of the Ministry of Agriculture and Rural Affairs, Agricultural Genomics Institute at Shenzhen, Chinese Academy of Agricultural Sciences, Shenzhen, Guangdong 518120, China

**Keywords:** Mantodea, genome, transposable element, X1 × 2Y, evolution

## Abstract

**Background:**

Praying mantises, members of the order Mantodea, play important roles in agriculture, medicine, bionics, and entertainment. However, the scarcity of genomic resources has hindered extensive studies on mantis evolution and behavior.

**Results:**

Here, we present the chromosome-scale reference genomes of 5 mantis species: the European mantis (*Mantis religiosa*), Chinese mantis (*Tenodera sinensis*), triangle dead leaf mantis (*Deroplatys truncata*), orchid mantis (*Hymenopus coronatus*), and metallic mantis (*Metallyticus violacea*). The assembled genome sizes range from ∼2.3 to 4.2 Gb, with contig N50 size 1–109 Mb and 85%–99% of sequence anchored to chromosomes. The annotated protein-coding gene number ranges from 17,804 to 19,017, with a BUSCO complete rate of 96.7%–98.4%. We found that transposable element expansion is the major force governing genome size in Mantodea and suggest that translocations between the X chromosome and an autosome have occurred in the lineage of the family Mantidae. In addition, we found that the lineage of *M. violacea* has accumulated fewer substitutions than the lineages of other mantises. Furthermore, our genome-wide analyses showed that *D. truncata* is sister to *H. coronatus* compared with *M. religiosa* and *T. sinensis*, which helps resolve the phylogenic controversies of the *Deroplatys* genus.

**Conclusions:**

The high-quality genome assemblies of the 5 mantises provide a valuable resource for evolution studies of Mantodea and genetic improvement and breeding of beneficial biological control agents.

## Background

Praying mantises are familiar insects that play important roles in agriculture, medicine, and bionics. As predators of many harmful insect species, praying mantises such as the European mantis (*Mantis religiosa*, NCBI:txid7507) and Chinese mantis (*Tenodera sinensis*, NCBI:txid406589) are widely acknowledged as natural enemies that control plant pests [1], benefiting organic planting where pesticide is prohibited. The mantis ootheca (egg capsule, egg chamber) is a traditional medicine used to cure frequent micturition, strengthen kidney health, and prevent spermatorrhea in East Asian countries [2]. Most praying mantises have 2 sharp and strong forelegs, which are much larger and more powerful than their ancient ancestors. In addition, the femur and tibia of the forelegs are armed with strong spines along their posterior edges. When the femur and tibia fold on each other, a praying mantis can firmly grasp the prey. This distinctive body structure of the praying mantis has been a significant source of inspiration in the bionics of cutting blades [3, 4]. Although most mantises have XY sex chromosomes, some mantises such as *M. religiosa* and *T. sinensis* have the X1 × 2Y type [5–7], making them a special material for studying the evolution of sex chromosomes.

The 2 closely related orders, Mantodea (mantises) and Blattodea (cockroaches and termites), are classified into the superorder Dictyoptera, and phylogenomic analyses revealed that Mantodea split from Blattodea during the Permian [8]. Mantodea has evolved into a group comprising ∼2,500 species with diverse morphological and ecological characteristics, with the highest diversity in tropical and subtropical habitats [9, 10]. From fossils of early Mantodea and Blattodea species, the common ancestor is thought to resemble modern cockroaches in many aspects [11]. The metallic mantis (*Metallyticus violacea*, NCBI:txid406581), in the early diverging mantid lineage, has many morphological features similar to those of modern cockroaches, but the underlying mechanisms have not been discovered [12]. Although Mantodea are well supported as monophyletic, the phylogenetic relationships within Mantodea are still not well resolved. For example, the *Deroplatys* genus has been placed into the Mantidae family traditionally, but these species are more similar to species in the Hymenopodidae family in many aspects, such as morphology and camouflage [9, 13]. Clarifying the relationships within Mantodea will greatly benefit the functional studies as well as genetic breeding of mantises.

Compared with those of many other insect orders, the genomic resources of Mantodea are very limited, with only 3 chromosome-scale reference genomes available: the Chinese mantis (*T. sinensis*, NCBI:txid406589), orchid mantis (*Hymenopus coronatus*, NCBI:txid267205), and Malaysian dead leaf mantis (*Deroplatys lobata*, NCBI:txid1661821) [14, 15]. Here, we present the chromosome-scale reference genomes of 3 other mantis species, the European mantis (*M. religiosa*), triangle dead leaf mantis (*Deroplatys truncata*), and metallic mantis (*M. violacea*), as well as a more complete assembly of *T. sinensis* and *H. coronatus*, to promote the evolutionary and biological studies of Mantodea.

## Results

### Chromosome-scale genome assemblies of 5 mantis species

**Figure 1:**
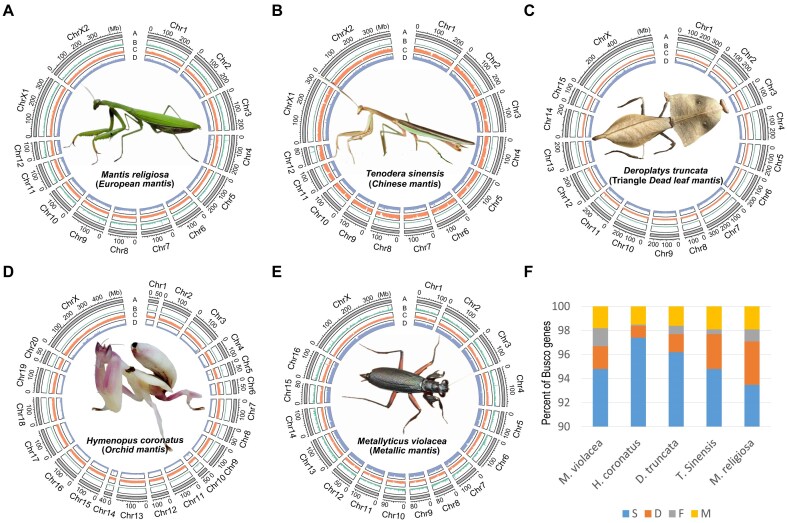
Overall view of genome assembly and annotation. Circos plots for *M. religiosa* (A), *T. sinensis* (B), *D. truncata* (C), *H. coronatus* (D), and *M. violacea* (E). Each circos plot has 4 tracks: track A represents chromosome length, track B represents gene density, track C represents transposable element (TE) density, and track D represents GC percentage. Feature density and GC percentage were calculated by sliding 1-Mb windows. The images of the sequenced specimens are shown in the center of circos plots. (F) BUSCO assessment (database: Insecta from OrthoDB v10) of gene sets for 5 mantis species. C: complete; F: fragmented; M: missing.

We generated 26–63× PacBio HiFi data and 35–79× Illumina Hi-C data for *M. religiosa, T. sinensis, D. truncata, H. coronatus*, and *M. violacea*, respectively (Supplementary Tables S1, S2). The PacBio HiFi reads were used to assemble the contig sequences, with a total size of 2.3–4.2 Gb and N50 sizes of 1–109 Mb. The Illumina Hi-C reads were mapped to the contig sequences, and the valid Hi-C read pairs were used for the scaffolding assembly (Supplementary Table S3), resulting in 85.39%–98.51% of the contig sequences anchored into 14–21 chromosome-level scaffolds (Fig. 1A–E, Supplementary Fig. S1, Supplementary Fig. S2, Table 1, Supplementary Table S4). Notably, only the chromosome numbers for *M. religiosa* and *T. sinensis* have been karyotyped [16, 17], whereas the others are inferred only from the genome assembly. Using the Genome Character Estimation (GCE) method that estimates genome size with *k*-mer frequency distribution from sequencing reads [18], the estimated genome sizes are 3.5 Gb (*M. religiosa*), 2.8 Gb (*T. sinensis*), 4.3 Gb (*D. truncata*), 3.1 Gb (*H. coronatus*), and 2.3 Gb (*M. violacea*), consistent with assembled genome sizes. Based on the GCE heterozygosity model [18], the estimated heterozygous rates approach 2% for *M. religiosa* and *T. sinensis*, whereas they are around 0.05%–1% for the other 3 species. Owing to the higher heterozygosity rate (Supplementary Fig. S3), the contig sizes for the *M. religiosa* and *T. sinensis* are shorter than those for the other 3 species.

**Table 1:** Statistics of genome assembly and annotation

Genomic features	*M. religiosa*	*T. sinensis*	*D. truncata*	*H. coronatus*	*M. violacea*
Genome assembly					
Estimated genome size by *k*-mer (bp)	**3,519,843,697**	**2,865,686,147**	**4,337,798,490**	**3,167,239,197**	**2,331,221,057**
Total assembly size (bp)	**3,680,002,721**	**2,687,426,722**	**4,290,792,545**	**3,127,590,514**	**2,322,129,794**
Contig N50 size (bp)	**1,407,320**	**12,728,340**	**44,444,664**	**71,519,735**	**109,157,195**
Scaffold N50 size (bp)	**210,326,877**	**190,002,057**	**248,405,437**	**159,059,693**	**125,733,329**
No. of assembly-inferred chromosomes	**12+X1+X2**	**12+X1+X2**	**15+X**	**20+X**	**16+X**
% sequence anchored to chromosome	**85.39%**	**95.63%**	**97.47%**	**98.27%**	**98.51%**
Genome annotation					
Length and % of tandem sequences (bp)	**396,842,330 (10.8%)**	**403,304,947 (15.0%)**	**471,243,565 (11.0%)**	**238,530,960 (7.6%)**	**186,949,249 (8.1%)**
Length and % of TE sequences (bp)	**2,501,898,483 (68%)**	**1,710,668,926 (64%)**	**2,928,636,453 (68%)**	**2,122,785,940 (68%)**	**1,351,077,317 (58%)**
Number of protein-coding gene models	**19,017**	**19,007**	**18,156**	**18,536**	**17,804**
Mean CDS length (bp)	**1,551**	**1,782**	**1,601**	**1,523**	**1,152**
Mean exon number	**6.07**	**5.93**	**6.34**	**6.33**	**5.54**

“No. of assembly-inferred chromosomes” refers to autosome and sex chromosome number in females.

Recently, Huang et al. [14] published a reference genome for *H. coronatus* with a much shorter contig N50 size of 15.7 Mb, and Yuan et al. [15] published a reference genome of *T. sinensis* with a contig N50 size of 2.36 Mb, which is also much shorter than that of this study. The difference in assembly continuity is mainly caused by the applied sequencing technologies. We used HiFi long reads with over 99% accuracy, while they used 1-pass long reads with only ∼85% accuracy. From syntenic alignments of the 2 assemblies for *H. coronatus*, we found that most chromosomes were largely consistent except for the X chromosome (Supplementary Fig. S4). One complete X chromosome in our assembly corresponds to 3 fragmented chromosomes in the assembly by Huang et al. [14]. The X chromosome is the largest chromosome, making it more difficult to assemble than the autosomes. With much longer assembled contigs and EndHiC that is specially designed for scaffolding large contigs, we achieved a complete assembly of the X chromosome for *H. coronatus*. We also compared another reference genome published by Huang et al. [14], the Malaysian dead leaf mantis (*D. lobata*, CRA010804, in the National Genomics Data Center, China), to our assembled reference genome of *D. truncata* (Supplementary Fig. S5). Belonging to the same genus, most chromosomes have high synteny, except for 4 chromosomes involved in chromosome-level rearrangements, which are more likely due to species divergence than assembly errors. Both reference genomes of *T. sinensis* (Yuan et al.[15] and the present study) showed high synteny for all chromosomes (Supplementary Fig. S6).

By integrating homology and transcription evidence, 17,804–19,017 protein-coding gene models were annotated as the reference genes for each mantis (Table 1, Supplementary Table S5). The BUSCO complete rates for the reference genes of these mantis species range from 96.7% to 98.4% (Fig. 1F), which are higher than or comparable to those of previously published mantis genomes [14, 15]. Furthermore, 97.2%–98.6% of the reference genes in these 5 mantis species were assigned functions according to at least one of the NCBI-NR, KEGG, InterPro, or GO databases.

### Distinct transposable element expansions in various mantid lineages

**Figure 2:**
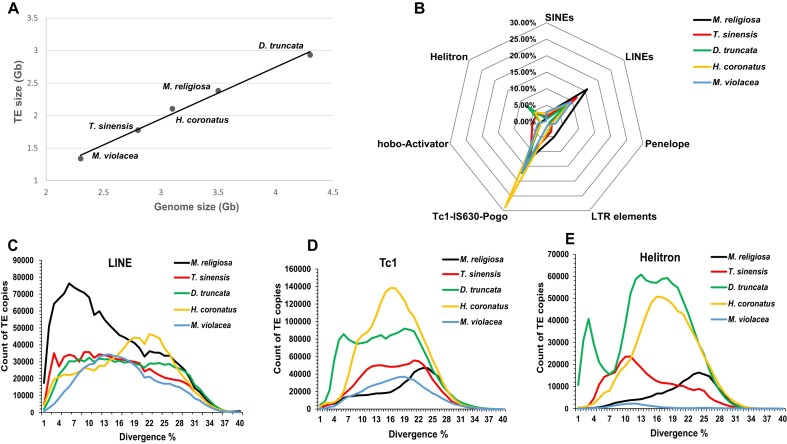
TE distribution in 5 mantis genomes. (A) The relationship between genome size and TE content. A linear trend line of the average of all the points is shown. Phylogenetic generalized least squares regression (PGLS) was applied, resulting in *R* = 0.954 and *P* = 0.008 < 0.01, suggesting that there is a very strong correlation between genome size and TE content. (B) The radar chart for major components of TE. Each vertex refers to a type of TE, and species are represented by different colors. The percentage for each height level of the vertex represents the percentage of the genome occupied by TEs. LINEs, Tc1-IS630-Pogo, and Helitrons have sharp peaks, indicating a burst of TEs for that type. (C–E) The divergence (%) distribution of LINE, Tc1, and Helitrons, respectively.

Increasing evidence has shown that transposable elements (TEs) contribute significantly to the genome size and influence the genome architecture, along with insertions, deletions, translocations, and so on [19]. We analyzed the total TE content (ratio) among the 5 species and found that genome size was linearly correlated with TE abundance (Fig. 2A, Supplementary Fig. S7). Currently, the orders of Mantodea and Blattodea are placed within the superorder Dictyoptera. Mantidae is a representative mantis family, which belongs to Mantoidea (superfamily) in Mantodea. The 2 Mantidae species (*M. religiosa* and *T. sinensis*) have relatively smaller genome sizes (2.3–2.8 Gb) and lower TE contents (58%–63%), than the other 3 mantises, with relatively larger genome sizes (3.1–3.5 Gb) and higher TE contents (67%–68%), suggesting that genome size differences are mostly determined by TE contents in mantids.

Abundant retrotransposons, DNA transposons, and rolling-circle transposons were found in these mantis genomes, but their ratios in genome differ across species (Fig. 2B, Supplementary Tables S6, S7). For the 2 Mantidae species, Long Interspersed Nuclear Elements (LINEs) are the largest components, and a sharp expansion of LINEs with divergence of approx. 7% was found in *M. religiosa* (Fig. 2C). However, no recent large-scale expansion of LINEs has occurred in *T. sinensis*, which may explain why its genome size (2.8 Gb) is smaller than that of *M. religiosa* (3.5 Gb). In contrast, *D. truncata* and *H. coronatus* have massive DNA transposons, with Tc1 (especially Tc1-IS630-Pogo) being the largest component in these 2 species, consistent with the findings of a former study [14]. *D. truncata* has undergone both a recent sharp expansion and an ancient burst of Tc1 in its genome, leading to the largest genome size (4.3 Gb) found in this study, whereas only an ancient explosion of Tc1 was observed in *H. coronatus* (Fig. 2D). Both *D. truncata* and *H. coronatus* also have a large rolling-circle transposon, Helitrons. Both a recent and an ancient burst of Helitrons were observed in *D. truncata*, whereas only an ancient burst of Helitrons was found in *H. coronatus* (Fig. 2E). *M. violacea* shows no recent accumulation of any category of TEs, which may explain why its genome size (2.3 Gb) was the smallest among these mantises.

These results collectively suggest that TE expansion is the major force behind genome size variation in Mantodea. In addition, the components and divergence times of the various TE types are distinct among the different mantid lineages.

### Translocation between X chromosome and autosomes in Mantidae lineage

Sex chromosomes generally evolved from autosomes and occasionally from B chromosomes, playing important roles in tissue development, mating, and speciation [20]. The types of sex chromosomes found in insects vary among species, and sex chromosome systems exhibit significant diversity across insect species [5–7]. Most insects have XY, ZW, or XO sex chromosome systems, but there are other rare sex chromosome types, such as the X1 × 2Y type, which has 2 X chromosomes and 1 Y chromosome. Some hemipterans, such as *Philaenus italosignus* [21], and some mantids, such as *M. religiosa* [22], exhibit this sex chromosome type. Studying mantis sex chromosomes will uncover the formation mechanism underling the X1 × 2Y type.

**Figure 3:**
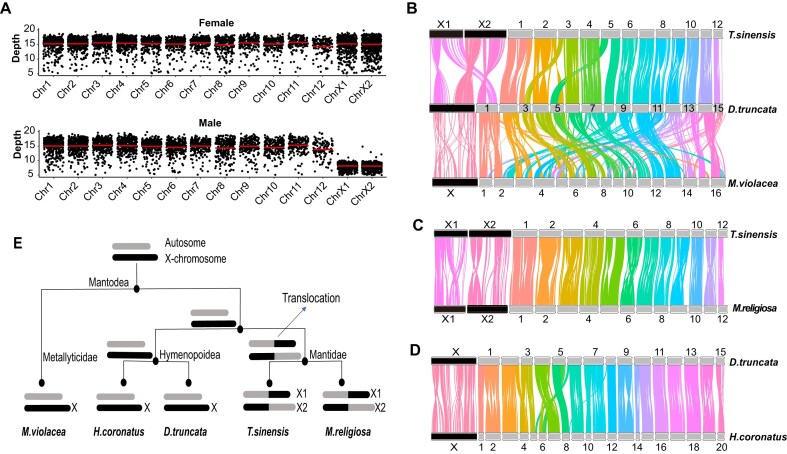
Evolution of X chromosomes in Mantodea. (A) Identification of X chromosome in *T. sinensis* by comparing depths between male and female individuals. The sequencing depth distributions were plotted in 500-Kb windows. The red line represents the average sequencing depth for each chromosome. (B) The synteny band plot among *T. sinensis, D. truncata*, and *M. violacea*, using 9,117 reciprocal best hits between *T. sinensis* and *D. truncata*, as well as 8,765 reciprocal best hits between *D. truncata* and *M. violacea*. Many more chromosome rearrangements were observed between *M. violacea* and *D. truncata*, in comparison to those between *T. sinensis* and *D. truncata*. (C) The dual synteny between *M. religiosa* and *T. sinensis*, using 9,917 reciprocal best hits between *M. religiosa* and *T. sinensis*. All chromosomes have a 1:1 relationship. (D) The dual synteny between *D. truncata* and *H. coronatus*, using 9,821 reciprocal best orthologous genes between *D. truncata* and *H. coronatus*. Four chromosome breaks and 3 interchromosome translocations were observed. (E) The diagram shows the evolutionary process of the X chromosome along various lineages of Mantodea. For subfigures B–E, the black box represents X chromosomes, while gray box represents autosomes.

To identify the X chromosomes from the assembled pseudochromosomes, we generated 15× short-read sequencing data for the female and male *T. sinensis* individuals, respectively. Sequencing coverage revealed that all 14 chromosomes in females have comparable coverage depths, whereas in males, 2 chromosomes have approximately half the coverage depth (Fig. 3A). It has been reported that most members of the family Mantidae have 2 X chromosomes, X1 and X2, derived from fusion or translocation between the X chromosome and an autosome [16]. In addition, the genome assembly of *T. sinensis* was derived from a female individual, which lacked the Y chromosome. Thus, the 2 chromosomes with half-coverage depths are concluded to be the X1 and X2 chromosomes. Notably, they are the largest and second largest of our assembled pseudochromosomes, consistent with previous reports based on karyotyping [16, 23].

Macroscale synteny analysis identified the corresponding X chromosomes in the other 4 species and allowed comparative analysis among the mantid X chromosomes. Synteny alignments revealed that both Mantidae species *M. religiosa* and *T. sinensis* have 2 sex chromosomes, X1 and X2; however, the other species have only 1 sex chromosome, X. In addition, only part of X1 (X1L) and X2 (X2L) in Mantidae was aligned with the X chromosomes of the other 3 species (Fig. 3B–D, Supplementary Fig. S8). These results suggest that the ancestral mantid had 1 X chromosome and that the translocation of large fragments between the X chromosome and an autosome occurred in Mantidae (Fig. 3E). Previous studies have revealed that the common ancestor of Dictyoptera had a XY sex chromosome system [16, 23] (Supplementary Fig. S9). We inferred that the common ancestor of the Mantidae family evolved the X1 × 2Y sex chromosome system, and our results support a model in which the generation of the X1 and X2 chromosomes resulted from the translocation between 1 X chromosome and an autosome.

Furthermore, based on macroscale synteny analysis, we were able to identify the breakpoints as a site falling within a 6.65-Mb region on the X1 chromosome and a site falling within a 2.56-Mb region on the X2 chromosome of *T. sinensis*. Inside these 2 regions, transposon and tandem repeats dominate the sequence (Supplementary Fig. S10), posing great difficulties for accurate genome assembly and interspecies genomic sequence alignment. In the future, as the assembly continuity improves, it is possible to narrow down the breakpoint range, which will approach or surpass the resolution of traditional cytological technologies such as C-banding, silver staining, and living-cell images of the meiosis process [16, 23].

### Mantodea phylogeny provide taxonomic evidences for *D. truncata*

Comparative analysis of Mantodea genomes within a phylogenetic context is essential for understanding their evolution and diversity. Phylogenomic analyses were performed on these 5 Mantodea species, which span 5 genera and 3 families with diverse habitats and morphologies. Two Blattodea species, the German cockroach (*Blattella germanica*) [24] and the dampwood termite (*Zootermopsis nevadensis*) [25], were used as the outgroup (Supplementary Table S8). From gene family clustering, 69,603 orthologous groups (OGs) were generated for all the analyzed species, including 4,014 single-copy OGs in which each mantis must be present and have only a single copy.

**Figure 4:**
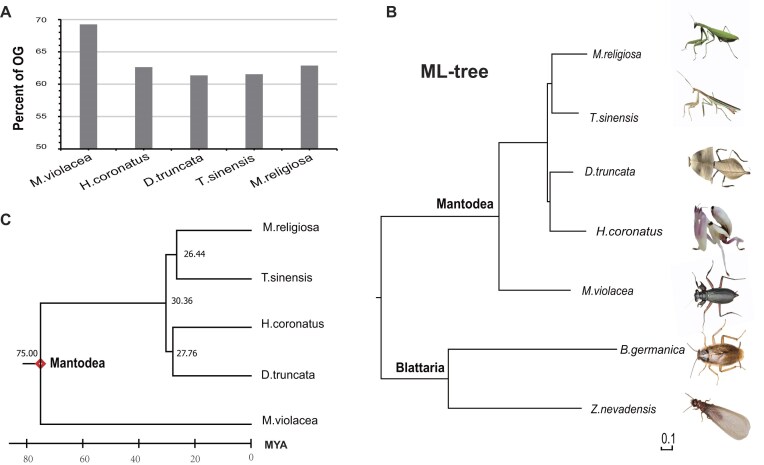
Orthologous groups (OGs) and phylogeny of Mantodea. (A) Percentage of OGs shared with cockroach for each mantis species. When an OG contain 1 or more genes for both an analyzed mantis and the cockroach, it was counted for the analyzed mantis as shared OGs. (B) Phylogeny is based on protein sequence alignment of 4,014 single-copy genes (mantises, cockroaches, and termites) with the maximum likelihood (ML) method using the LG amino acid substitution model. The branch length is in proportion to the substitution rate. (C) Time tree inferred by the Reltime-Branch lengths method in MEGA, with 1 calibration constraint (70–80 Mya between *M. violacea* and *M. religiosa*).

*M. violacea* belongs to the superfamily Metallyticoidea, exhibiting significant morphological differences compared to other mantises. *M. violacea* shares many characteristics with its cockroach relatives, including dull body coloration, a prostrate body posture, and a relatively shorter prothorax. The cockroach-like body morphology was thought to be an ancestral trait of the Mantodea order [11]. The Metallyticoidea lineage is sister to the other mantis lineages [26]. *M. violacea* shares many more OGs with cockroaches than the other 4 species (69% vs. 61%–63%) (Fig. 4A), which may partly explain its strong morphological resemblance to cockroaches.

The phylogenetic tree was constructed based on 4,014 single-copy OGs, and the divergence time along branches was estimated. From the phylogenetic tree, we observed that the branch length from the Mantodea node to the *M. violacea* node is much shorter than that from the Mantodea node to the species nodes of other mantises (Fig. 4B). Branch length in an undated tree represents the number of substitutions per site in the sequence alignment. Thus, the shorter branch length of *M. violacea* means fewer substitutions accumulated from the Mantodea ancestor to *M. violacea*. The divergence time was inferred by the Reltime-Branch lengths method, using 1 calibration constraint (70–80 million years ago [Mya] between *M. violacea* and *M. religiosa*). The results showed that 4 modern mantises (*D. truncata, H. coronatus, M. religiosa*, and *T. sinensis*) emerged within a short time period (26–31 Mya) (Fig. 4C), posing difficulties for phylogenic inference within this lineage.

The phylogenetic tree also revealed that *D. truncata* is closer to *H. coronatus* (Hymenopodidae) than *M. religiosa* and *T. sinensis* (Mantidea) (Fig. 4B), and the same topology was obtained by the Neighbor Joining (NJ) method, differing from the phylogenic assignment from some previous studies that place *D. truncata* within Mantidea [9, 13]. After adding the genomic data for *D. lobata*, both Deroplatys species were sistered to *H. coronatus* (Supplementary Fig. S11). Therefore, genome-wide data are helpful to clarify phylogeny controversies, providing important evidences for further species classification of Deroplatys.

## Discussion

In this study, we generated chromosome-level genome assemblies for 5 mantis species via a combination of PacBio HiFi and Hi-C sequencing technologies. For *H. coronatus* and *T. sinensis*, both the contig N50 and N90 sizes of our assembly are approximately 5 times greater than those of the previously published reference genomes [14, 15]. In our results, assembly continuity for *M. religiosa* and *T. sinensis* is relatively lower than that for the other 3 mantises due to the differences in heterozygosity, suggesting that high heterozygosity can be problematic for genome assembly. Compared with those of cockroaches and termites, the much larger genome sizes of mantises are mainly the result of expansions of various types of transposable elements.

Mantodea has occupied an important position in the evolution of insects, with distinctive morphology, camouflage behavior, and an unusual sex determination mechanism. One of its major sublineages, the family Mantidae, has a special X1 × 2Y sex determination system. Through comparative genomics analysis, we inferred that the mantid common ancestor had only 1 X chromosome, and translocation between the X chromosome and an autosome occurred in the ancestor of Mantidae. The *M. violacea* genome shares more orthologous genes with cockroaches than with the other mantises. Our phylogenetic analyses with genome-wide data also suggest that the 2 *Deroplatys* species are closer to *H. coronatus* than to the 2 Mantidea mantises, which may help further phylogenetic clarification and accurate species classification of *Deroplatys*. Based on very limited taxon sampling, we made a set of preliminary conclusions in this study, which will be verified by future studies as more genomes are sequenced. In addition, more advanced phylogenomic methods, such as coalescent-based approaches, may improve the accuracy of phylogenic inference for lineages that quickly diversified.

Although praying mantises are efficient predators, they do not exclusively target harmful insects, hindering their wide application in organic planting. Thus, the genomic resources generated in this study will also facilitate the molecular breeding of praying mantises, aiming to enhance their effectiveness as beneficial insects in pest control.

## Methods

### Insect collection and sequencing

Mantis adults were collected at different locations: *M. religiosa* and *T. sinensis* from the forest of Guangzhou, China; *H. coronatus* from the rainforest of Xishuangbanna, China; and *D. truncata* and *M. violacea* from 2 captive breeding centers in Beijing, China. The species were confirmed by morphological characters, and the photos for sequenced individuals are shown in Fig. 1. All mantis samples for sequencing had the intestine removed to avoid contamination by bacteria, fungi, and residual prey bodies. All the tissues were cleaned with 30% ethanol and ddH_2_O and then immersed in liquid nitrogen for cryopreservation.

For Pacific Biosciences (PacBio) HiFi sequencing, libraries with ∼15-kb insert sizes were constructed from a female adult of every mantis and sequenced on a PacBio Sequel II system (RRID: SCR_017,990). Subreads were generated with an N50 size of 14.5 kb, and consensus reads (CCS reads) were generated via ccs software (v.3.0.0) [27] with the following parameters: -min-passes 0 -min-rq 0.99 -min-length 100 -max-length 50,000. Then, Hi-C data were generated using the same individuals applied for HiFi sequencing. Nuclear DNA was cross-linked by soaking leaf tissues in formaldehyde solution, and the cross-linked genomic DNA was extracted, digested, repaired, ligated to circular fragments, sheared into 350-bp inserts, converted to a short-read sequencing library by the Truseq DNA Library Prep Kit (Illumina), and sequenced on the Illumina NovaSeq 6000 platform (RRID:SCR_016387). To identify the sex chromosome, short-read sequencing of a male adult and another female adult of *T. sinensis* was performed on the Illumina NovaSeq 6000 platform (RRID:SCR_016387), using DNA library with a 400-bp insert size constructed via the Truseq DNA Sample Prep Kit (Illumina).

Total RNA from the abdomen, hind leg, middle leg, foreleg, thorax, head, and eye of a female adult for each species were extracted with TRIzol reagent (Invitrogen) and used to construct cDNA libraries with the Truseq RNA Sample Prep Kit (Illumina). Transcriptome sequencing data were generated via the Illumina NovaSeq 6000 system in PE150 mode.

### Genome assembly and quality assessment

*K*-mer frequencies from HiFi reads of 5 mantises were calculated via Kmerfreq [18], and then genome sizes were estimated via GCE (RRID:SCR_017332). The PacBio HiFi reads were assembled into contigs via Hifiasm (v0.14) (RRID:SCR_021069) [28] with the following parameters: -l 1 -s 0.7. To filter duplicated contigs in the assembly, purge_dups (v1.2.3) (RRID:SCR_021173) [27] was adopted with the following parameters: -2 -a 50. The completeness of the assembly was evaluated using BUSCO (v5.2.2) (RRID:SCR_015008) based on the OrthoDB (v10) (RRID:SCR_011980) Insecta database [29].

For Hi-C scaffolding, 2 strategies, YaHS and EndHiC, were applied. YaHS is capable of constructing chromosome-level scaffolds with relatively shorter contigs, while EndHiC is more suitable for relatively larger contigs. For *M. religiosa*, whose assembly was fragmented into more contigs, Hi-C reads were mapped to contigs via the Arima mapping pipeline (ArimaGenomics), and then, YaHS (v1.2a.1) (RRID:SCR_0229650) [30] was used to assemble the contigs into pseudo-chromosomes. For the other 4 mantises, whose contigs are much larger, Hi-C reads were mapped to contigs by Bowtie 2 (v 2.2.2.7) (RRID:SCR_016368) [31], HiC-Pro (v2.11.0-beta) (RRID:SCR_017643) [32] was adopted to identify valid ligation pairs and generate Hi-C link matrices among different contigs, and finally, the contigs were clustered, ordered, and oriented into pseudo-chromosomes using EndHiC (v1.0) (RRID:SCR_022110) [33] based on the Hi-C linkage information among contig ends.

### Genome annotation

A *de novo* TE library was constructed with RepeatModeler (v2.0.2) (RRID:SCR_015027) with the parameters -engine ncbi-database [34], and then RepeatMasker (v4.1.0) (RRID:SCR_012954) was used to identify TEs in the reference genome, using both the *de novo* TE library and the public Repbase TE library (v26.05) (RRID:SCR_021169). The tandem repeat elements in the genome were subsequently identified using Tandem Repeats Finder (TRF) (RRID:SCR_022193) (v4.09) [35].

The protein-coding gene models were annotated in 2 rounds. In the first round, the genes were predicted by integrating evidence from *de novo* gene predictions and transcriptome-based gene predictions. *De novo* gene prediction was performed on the TE-masked genome assembly with AUGUSTUS (v3.4.0) (RRID:SCR_008417) [36]. For transcriptome-based gene prediction, the RNA sequencing data were filtered by Fastp (v0.23.1) (RRID:SCR_016962) [37] and then mapped to the genome using Bowtie2 (v2.2.7) [31], and StringTie (v1.3.3b) (RRID:SCR_016323) was then used to construct the gene models [38]. All the gene models obtained via the above 2 approaches were subsequently integrated with EVidenceModeler (v1.1.1) (RRID:SCR_014659) [39]. In the second round, for each mantis, the protein sequences from the other 4 sequenced mantises in this study were mapped to this genome assembly with Exonerate (v2.4.0) (RRID:SCR_016088) [40], and incomplete gene models were filtered. Finally, for each mantis, the *de novo* gene predictions, the transcriptome-based gene predictions, and the homology-based gene predictions were integrated with EVidenceModeler (v1.1.1) to generate a high-confidence and nonredundant gene set.

The completeness of the gene sets was assessed using BUSCO based on OrthoDB (v10) for Insecta. For gene functional annotation, the mantis protein sequences were aligned to the KEGG (RRID:SCR_012773), eggNOG (RRID:SCR_002456), NR, and UniProt (SwissProt) databases using DIAMOND (v0.9.24.125) (RRID:SCR_009457) [41], and only the best hits with E-values less than 1e^−5^ were retained. Moreover, InterProScan (v5.38) (RRID:SCR_005829) was used to annotate the protein domains and GO terms [42].

### X chromosome identification and analysis

To identify the X chromosome of *T. sinensis*, the clean Illumina paired reads from female and male samples were mapped to the genome of *T. sinensis* via BWA (v0.7.17-r1188) (RRID:SCR_010910) [43]. The bam files were filtered using SAMtools (v1.6) (RRID:SCR_002105) [44] with the parameters “-q 60 -F 1804,” and paired reads mapped onto different chromosomes were also filtered. To assess the sequencing depth of each chromosome, SAMtools depth (v1.6) was used to calculate the average base coverage. The 2 chromosomes in males, whose sequencing depth was approximately half that of the other chromosomes, were identified as X-derived chromosomes. For consistency with the karyotype results for *T. sinensis* [22] and *M. religiosa* [16], the larger one was denoted X2, whereas the smaller one was denoted X1.

Pairwise collinearity analyses were conducted using the protein sequences of 5 mantis species as markers. DIAMOND (v0.9.24.125) with the parameters “blastp -f 6” was used to align the protein sequences of each species pair, and the reciprocal best pairs were used as inputs for MCScanX (RRID:SCR_022067) to identify syntenic blocks [45]. The interspecies syntenic genomic blocks were visualized via the R package Ideogram [46]. Based on the collinearity alignments of the 5 mantises, the X chromosomes of the other 4 mantises were also identified. In addition, the translocation sites on chromosomes X1 and X2 were inferred from the collinearity alignment.

### Evolutionary analysis

Seven Dictyoptera species, including the 5 mantises sequenced in this study, as well as *B. germanica* (PRJNA203136 in NCBI) [24] and *Z. nevadensis* (PRJNA203242 in NCBI) [25], were used to construct OGs and infer orthologous genes via OrthoFinder (v2.5.4) (RRID:SCR_017118) with the default parameters [47]. The protein sequences of single-copy genes from each species were multiply aligned using MAFFT (v7.487) (RRID:SCR_011811) and then concatenated into 1 super protein sequence. Using the concatenated super protein sequence alignment, RAxML (v8.2.12) (RRID:SCR_006086) was subsequently employed to construct a maximum likelihood phylogenetic tree with the PROTGAMMALGX (“PROT” means protein sequence, “GAMMA” means gamma distribution, “LG” refers to amino acid substitution model, “X” means maximum likelihood estimation) model [48]. To verify the topology of the ML tree, a NJ tree was also constructed using the neighbor joining algorithm in MEGA (X) (RRID:SCR_002805) [49]. The time tree was inferred using the Reltime-Branch lengths method in MEGA, with input of user-supplied branch lengths derived from the maximum likelihood (ML) method. The time tree was computed using 1 calibration constraint (70–80 Mya between *M. violacea* and *M. religiosa*, [50]).

## Additional Files

**Supplementary Fig. S1**. Hi-C heatmap of genome assembly for *M. religiosa, T. sinensis, D. truncata, H. coronatus*, and *M. violacea*. The resolution (window size) is 1,000 Kb, and color represents log_2_(Links number). Links number is the number of Hi-C links falling between the 2 analyzed genomic windows.

**Supplementary Fig. S2**. Bandage view of scaffolding results for *M. religiosa, T. sinensis, D. truncata, H. coronatus*, and *M. violacea*. Each rectangle represents a contig. The chromosome ends assembled with telomere-specific tandem repeats (unit: TTAGG) are highlighted with a red circle.

**Supplementary Fig. S3**. Distribution of *k*-mer (*k*-size 17) frequencies in sequencing data for *M. religiosa, T. sinensis, D. truncata, H. coronatus*, and *M. violacea*. For each mantis, the left peak reflects the heterozygous regions, while the right peak reflects the homozygous regions, which are marked by an arrow. With a higher heterozygous rate, the left/right peak height ratios will be larger. The ratio of the left/right peak heights shows that *M. religiosa* and *T. sinensis* have a much higher heterozygosity than the other 3 mantis species.

**Supplementary Fig. S4**. Syntenic comparison of our *H. coronatus* assembly with the published assembly from Huang et al. (2023). The whole-genome alignment is performed by minimap2 with the “-x asm10” parameter.

**Supplementary Fig. S5**. Syntenic comparison of our *D. truncata* assembly with the published assembly of *D. lobata* from Huang et al. (2023). The whole-genome alignment is performed by minimap2 with the “-x asm10” parameter.

**Supplementary Fig. S6**. Syntenic comparison of our *T. sinensis* assembly with the published assembly from Ruizhong Yuan et al. (2023). The whole-genome alignment is performed by minimap2 with the “-x asm10”parameter.

**Supplementary Fig. S7**. The relationship between genome size and TE ratio of 5 Mantodea species. A linear trend line representing the average of all points is shown. The Pearson’s correlation coefficient (*r*) is as high as 0.894, with the *t*-test *P* ≈ 0.038 < 0.05, suggesting that there is a strong correlation between genome size and TE ratio.

**Supplementary Fig. S8**. Syntenic comparison of *T. sinensis* with the other 4 mantises. The whole-genome alignment is performed by minimap2 with the “-x asm10” parameter.

**Supplementary Fig. S9**. Sex determination system of Mantodea (mantises) and Blattodea (cockroaches and termites). The plot refers to “evobir.shinyapps.io/PolyneopteraDB/.”

**Supplementary Fig. S10**. Location of the broken site for translocation on the X1 and X2 chromosomes of *T. sinensis*. TE refers to transposable elements, and TR refers to tandem repeats. The gene density is shown in a heatmap, while the TE/TR density is shown as distribution curves. Through a macroscale synteny analysis between *T. sinensis* and *D. truncata* using reciprocal best orthologous genes as markers, we identified a broken site on ChrX1 (length 318,123,395 bp) within a 6.65-Mb region (107,057,327–113,703,447 bp). Additionally, we located a broken site on ChrX2 (length 396,970,917 bp) within a 2.56-Mb region (171,940,730–174,498,078 bp). X1L and X2L were derived from the ancestral X chromosome, while X1R and X2R were derived from the ancestral autosome. Each broken site region has an assembly gap inside, which is filled with absolute 1,000 N characters. Therefore, the size estimation of the broken site region was not as accurate. In addition, there were abundant TR and TEs in the broken site range, making it difficult to identify the accurate position where translocation happened. *M. religiosa* was not suitable for this analysis, because its current genome assembly is too fragmental.

**Supplementary Fig. S11**. Phylogeny tree for 8 Dictyoptera species. The phylogenetic tree was built based on 2,201 single-copy orthologs, with maximum likelihood methods (“raxmlHPC --m PROTGAMMALGX”). Bger: *B. germanica*; Dlob: *D.lobata*; Dtru: *D. truncata*; Hcor: *H. coronatus*; Mrel: *M. religiosa*; Mvio: *M. violacea*; Tsin: *T. sinensis*; Zoot: *Z. nevadensis*.

**Supplementary Table S1**. Background information of the sequenced mantis species.

**Supplementary Table S2**. Statistics of genome sequencing data.

**Supplementary Table S3**. Statistics of Hi-C data mapping to contigs.

**Supplementary Table S4**. Statistics of genome assembly.

**Supplementary Table S5**. Statistics of RNA-seq mapping and assembly.

**Supplementary Table S6**. Statistics of tandem repeats annotation.

**Supplementary Table S7**. Statistics of TE content in various classes.

**Supplementary Table S8**. Public genome data information.

## Abbreviations

BLAST: Basic Local Alignment Search Tool; bp: base pairs; BUSCO: Benchmarking Universal Single-Copy Orthologs; BWA: Burrows–Wheeler Aligner; CCS: circular consensus sequencing; Gb: gigabase pairs; GO: Gene Ontology; kb: kilobase pairs; KEGG: Kyoto Encyclopedia of Genes and Genomes; Ma: megaannus; Mb: megabase pairs; Mya: million years ago; NCBI: National Center for Biotechnology Information; NR: Non-Redundant; OG: orthologous groups; PacBio: Pacific Biosciences; PE: paired end; RAxML: randomized axelerated maximum likelihood; TRF: Tandem Repeats Finder; TE: transposable element; TPM: transcripts per million; YaHS: yet another Hi-C scaffolding.

## Supplementary Material

giaf158_Supplemental_File

giaf158_Authors_Response_To_Reviewer_Comments_original_submission

giaf158_Authors_Response_To_Reviewer_Comments_Revision_1

giaf158_GIGA-D-25-00308_original_submission

giaf158_GIGA-D-25-00308_Revision_1

giaf158_GIGA-D-25-00308_Revision_2

giaf158_Reviewer_1_Report_original_submission Xue-xin Chen -- 9/10/2025

giaf158_Reviewer_1_Report_revision_1 Xue-xin Chen -- 10/30/2025

giaf158_Reviewer_2_Report_original_submission Charlotte Julie Wright, MPhil -- 9/21/2025

giaf158_Reviewer_2_Report_revision_1 Charlotte Julie Wright, MPhil -- 10/24/2025

giaf158_Reviewer_2_Report_revision_2 Charlotte Julie Wright, MPhil -- 12/9/2025

## Data Availability

The genomic and transcriptomic sequencing reads have been deposited in NCBI-SRA under accession PRJNA987019, PRJNA989593, PRJNA989036, PRJNA988270, and PRJNA989282 for *M. religiosa, T. sinensis, D. truncata, H. coronatus*, and *M. violacea*, respectively. The corresponding genome assemblies and annotations have been deposited at NCBI-Genome under the accessions JAUKNK000000000, JAUKNM000000000, JAUKNL000000000, JAUKNX000000000, and JAUJEO000000000 and are also available at Figshare [51–55]. Other data supporting this work are available in the *GigaScience* database, GigaDB [56].
