# Supplementary material for: The genomes of 5 mantises provide insights into sex chromosome evolution and Mantodea phylogeny clarification
Source: Gigascience. 2025 Dec 18;15:giaf158. doi: 10.1093/gigascience/giaf158 (PMC12908712; doi:10.1093/gigascience/giaf158)
Supplement: giaf158_Supplemental_File [file giaf158_supplemental_file.docx]

**Supporting Information**

**Supplementary figures**


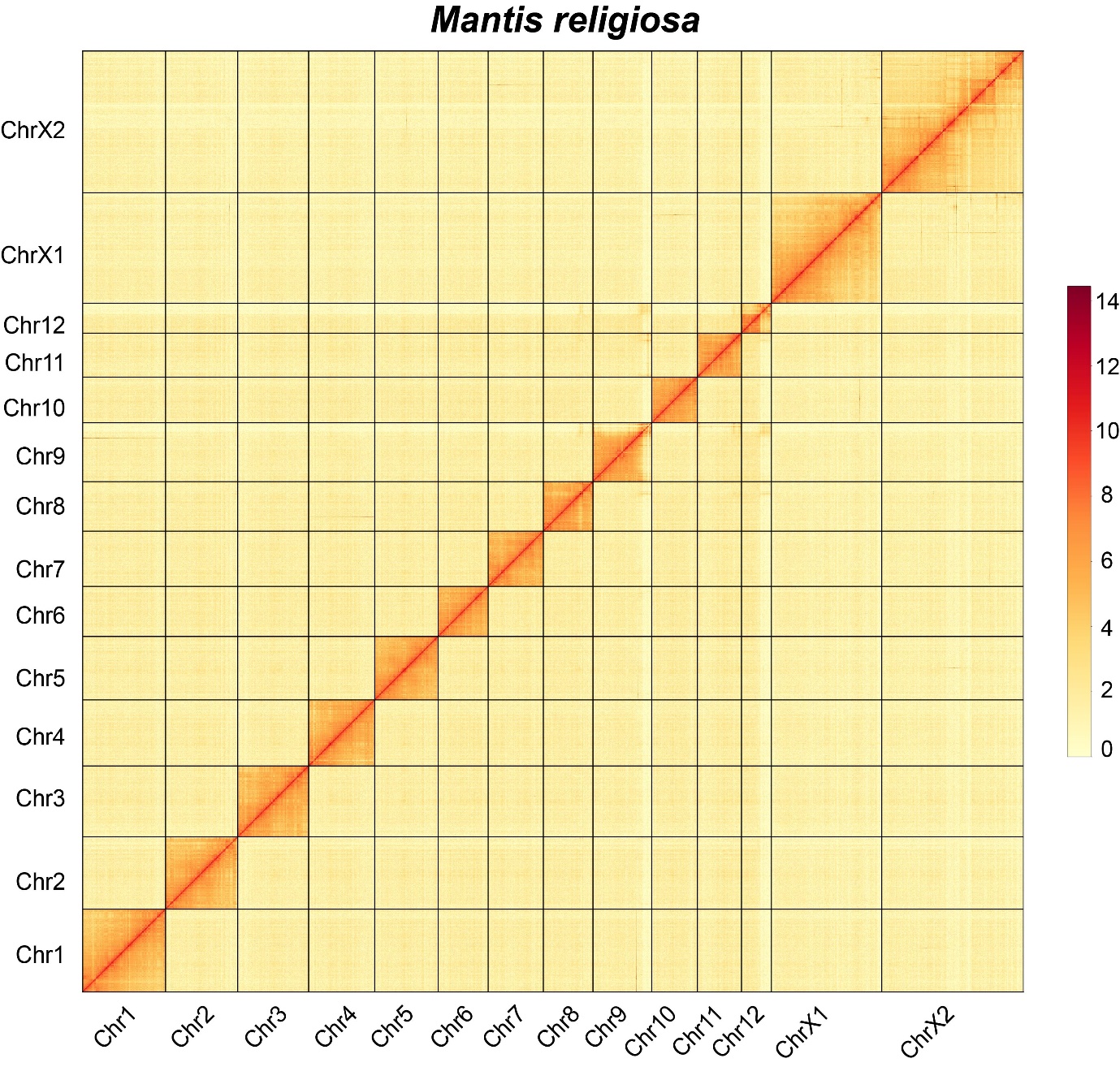

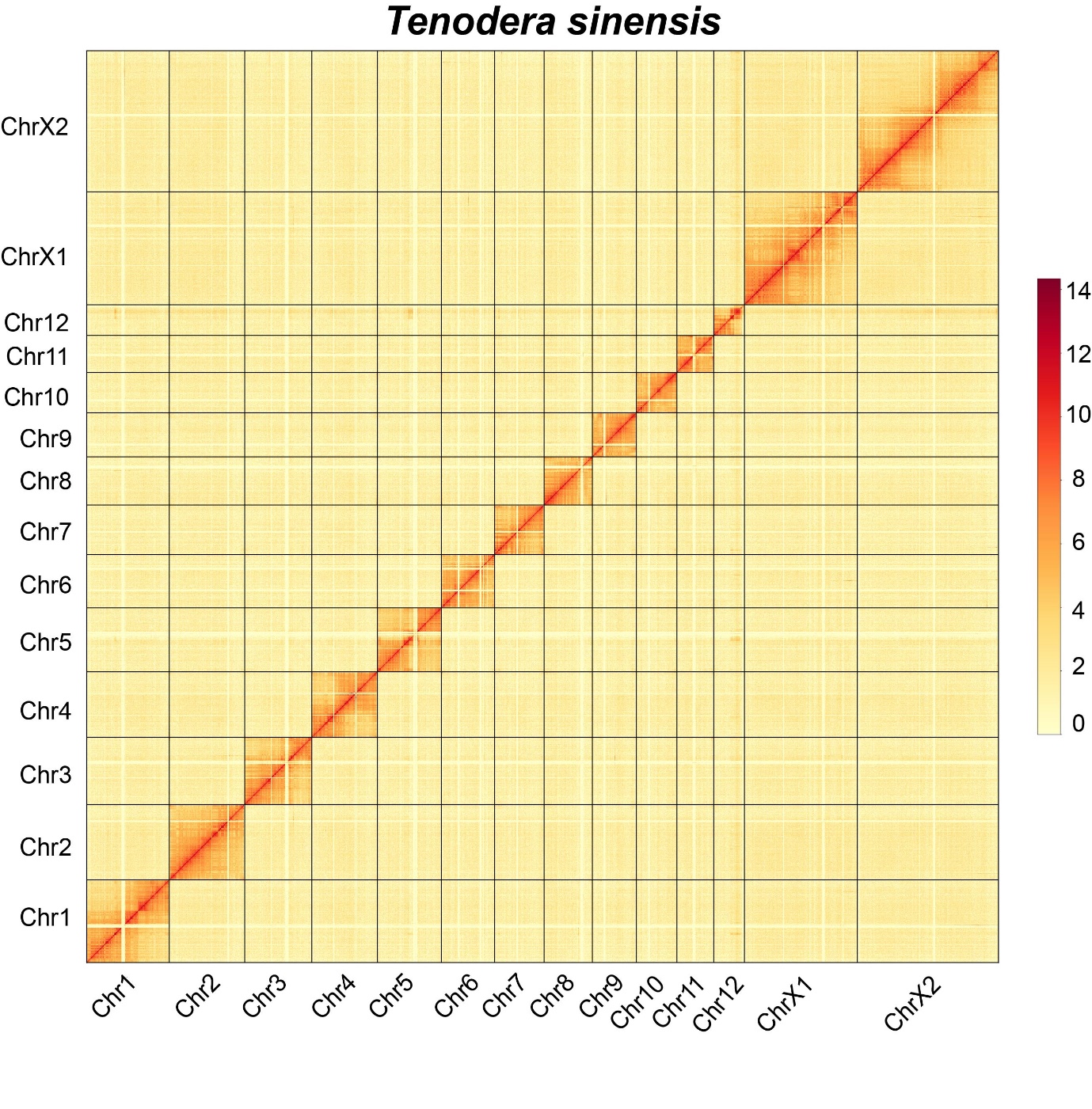

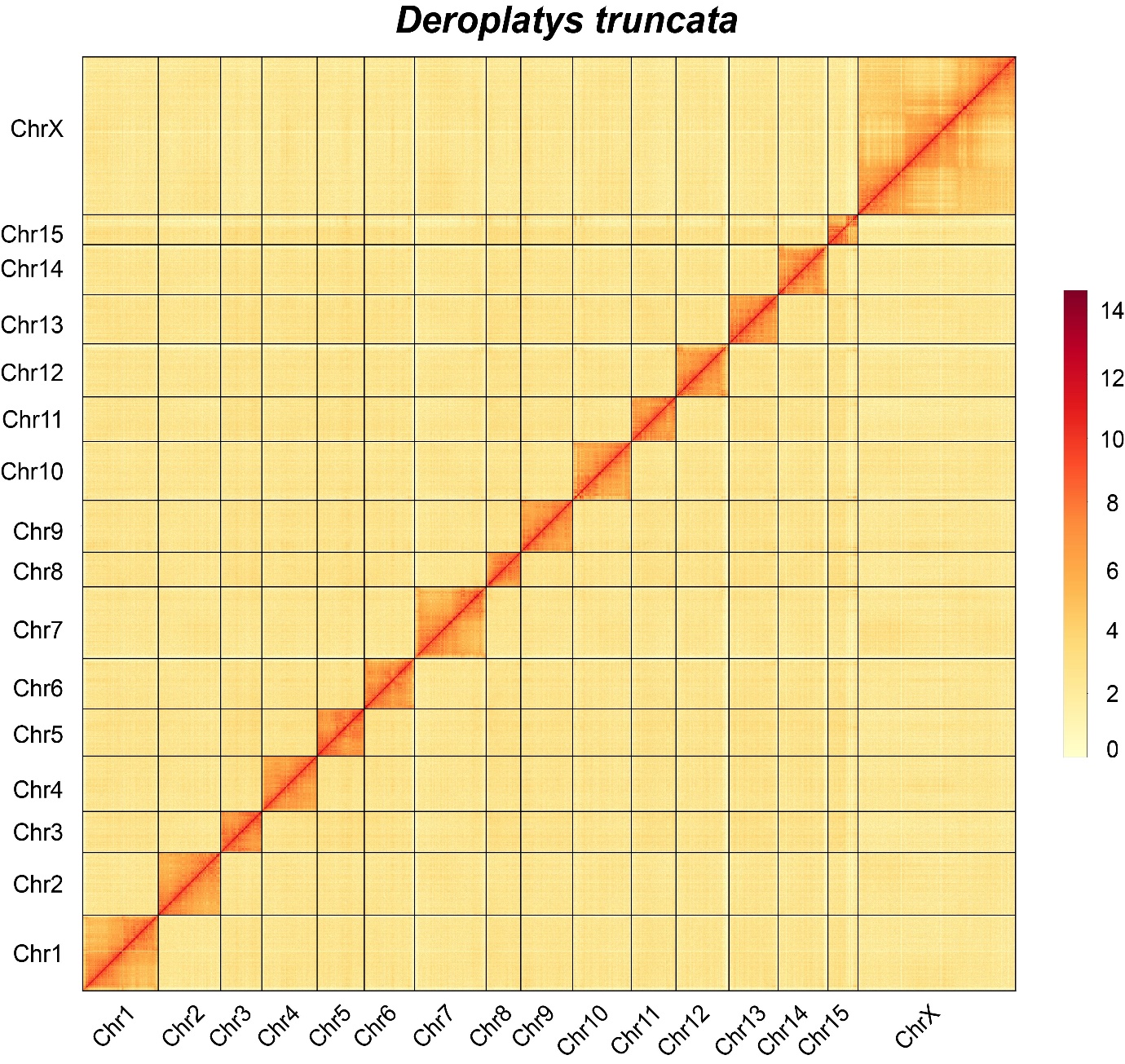

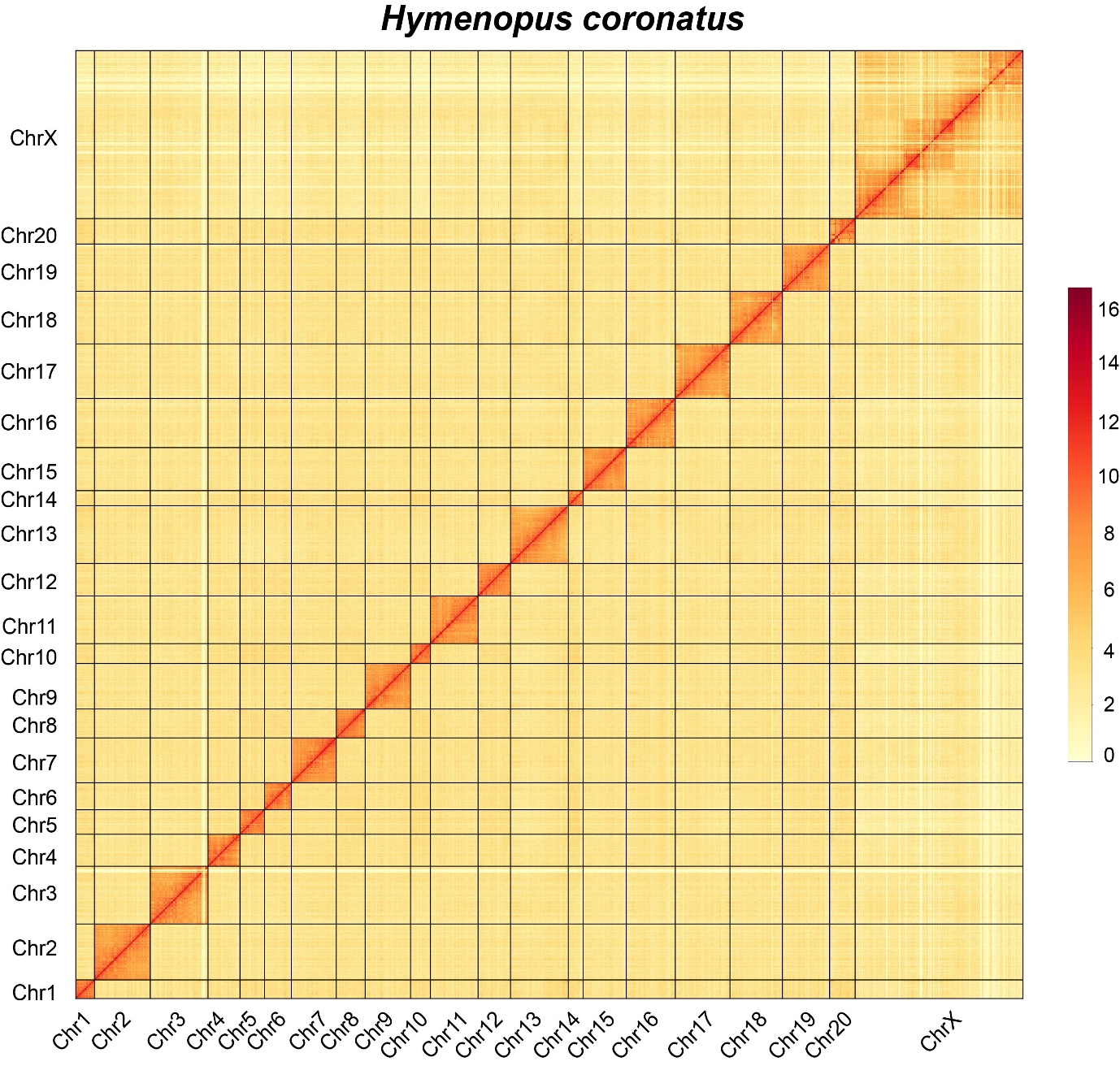


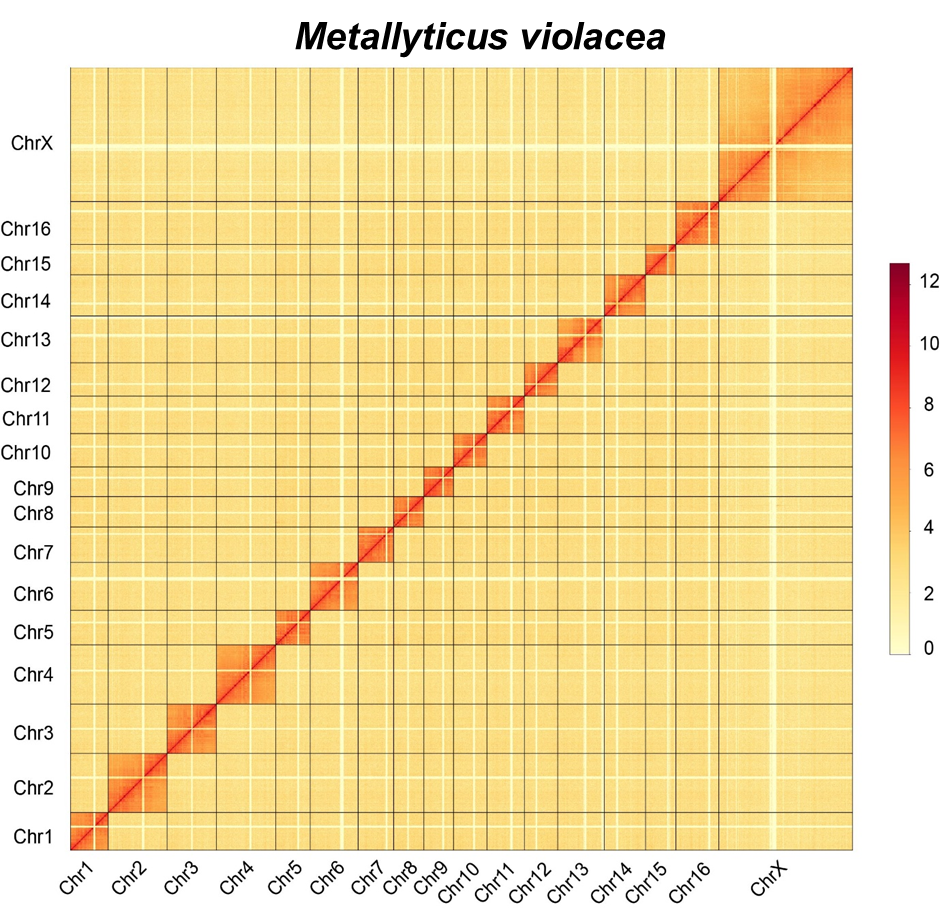


**Figure S1.** **Hi-C heatmap of genome assembly for *M. religiosa*, *T. sinensis*, *D. truncata*, *H. coronatus*,** **and *M. violacea*.** The resolution (window size) is 1000-Kb, and color represents log_2_(Links number). Links number is the number of Hi-C links falling between the two analyzed genomic windows.


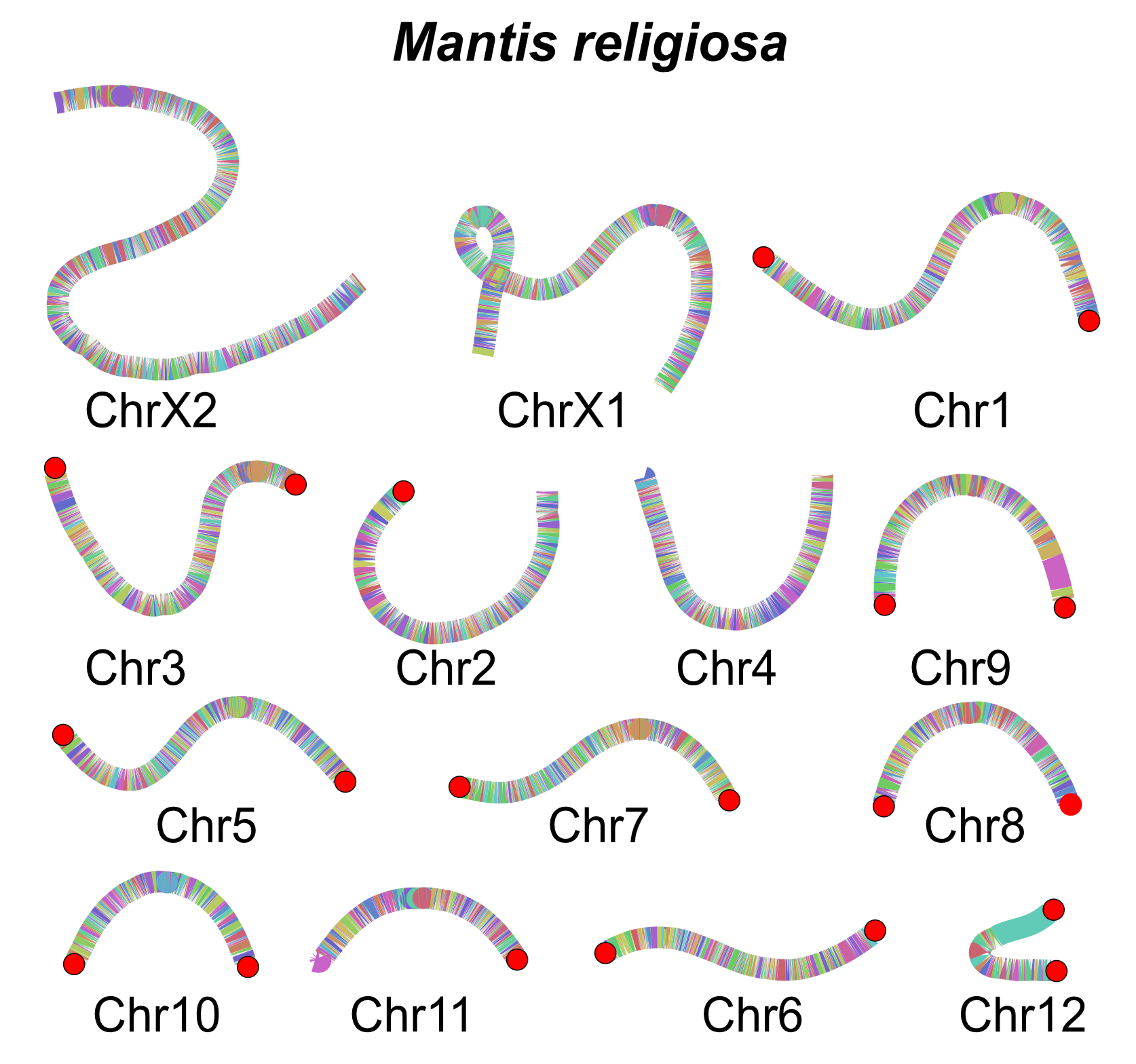


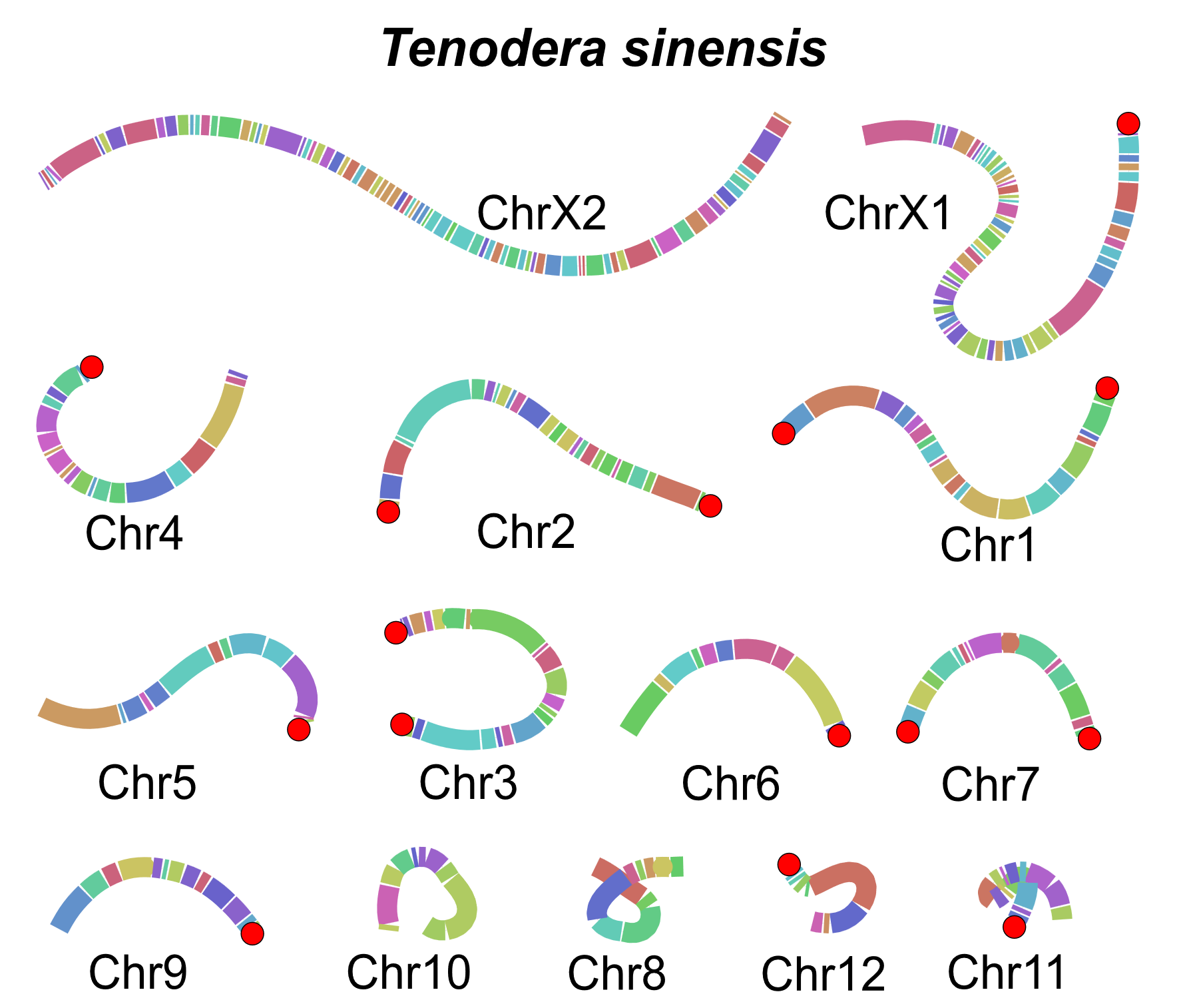

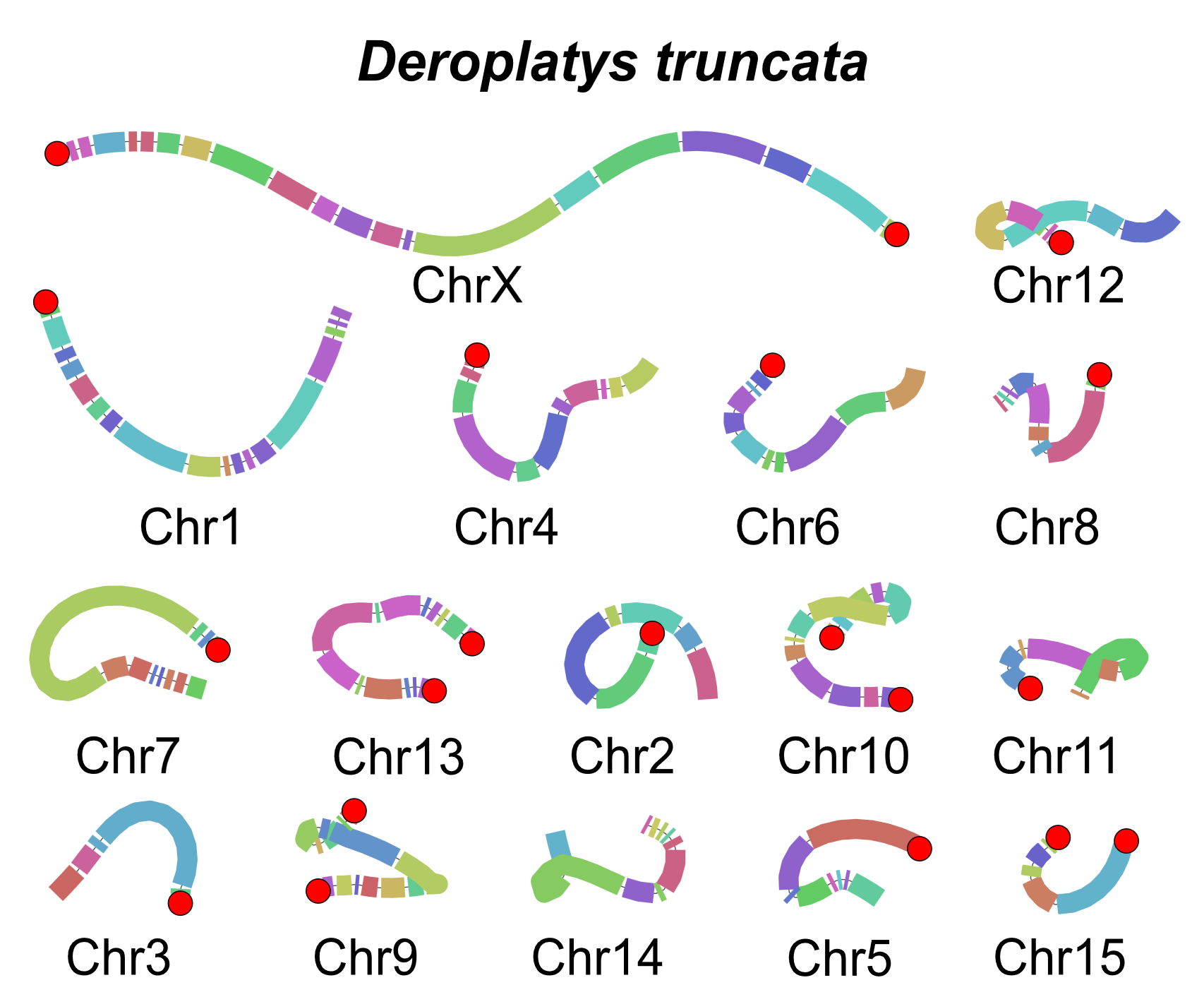

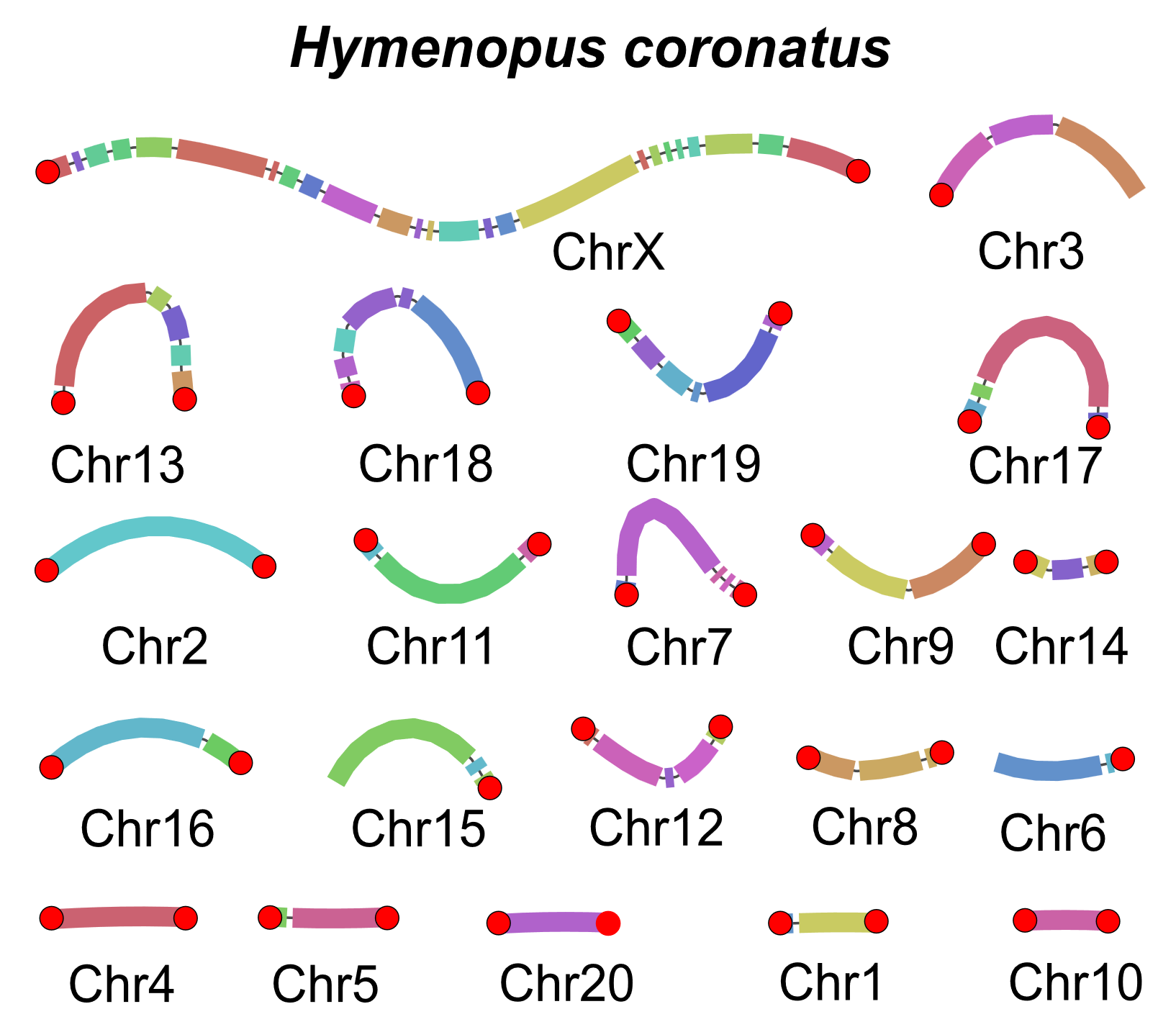


**
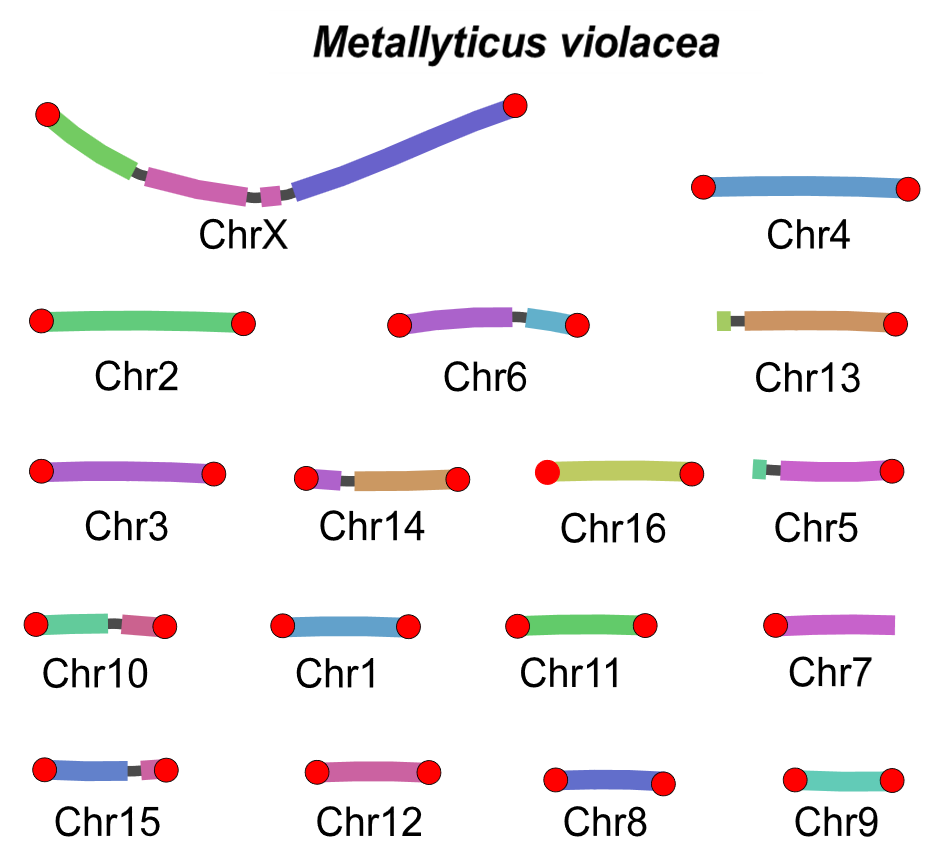
**

**Figure S2. Bandage view of scaffolding results for** ***M. religiosa*, *T. sinensis*, *D. truncata*, *H. coronatus*, and *M. violacea*.** Each rectangle represents for a contig. The chromosome ends assembled with telomere-specific tandem repeats (unit: TTAGG) were highlighted with red circle.

**
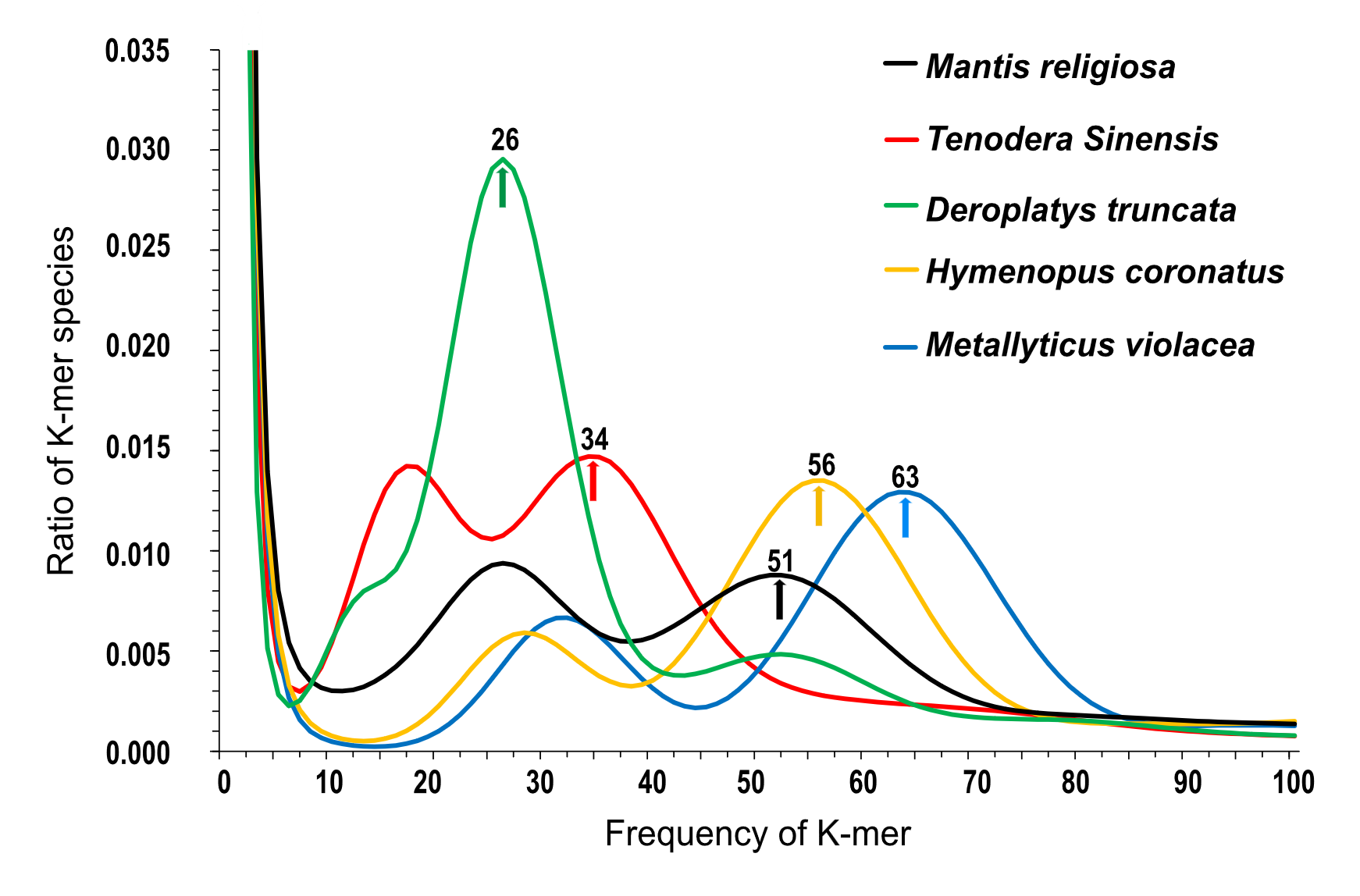
**

**Figure S3.** **Distribution of K-mer (K-size 17) frequencies in sequencing data for *M. religiosa*, *T. sinensis*, *D. truncata*, *H. coronatus*, and *M. violacea*.** For each mantis, the left peak reflects the heterozygous regions, while the right peak reflects the homozygous regions, which were marked by arrow. With higher heterozygous rate, the left/right peak height ratios will be larger. The ratio of the left/right peak heights show that *M. religiosa* and *T. sinensis* have much higher heterozygosity than the other 3 mantis species.


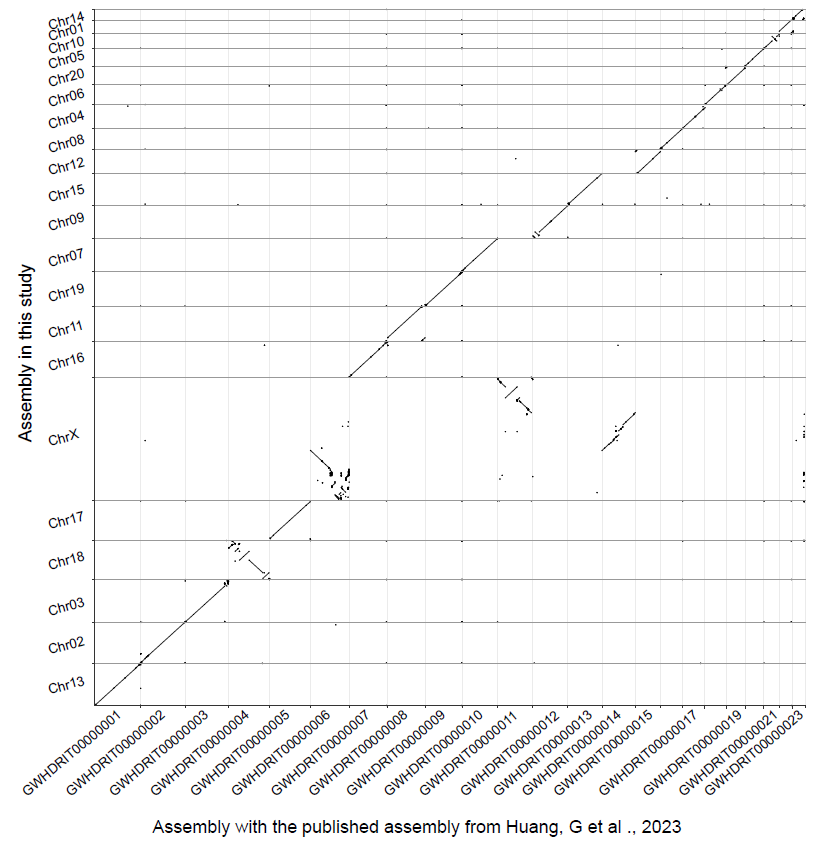


**Fig. S4. Syntenic comparison of our *H. coronatus* assembly with the published assembly from Huang, G *et al*., 2023.** The whole genome alignment is performed by minimap2 with “-x asm10” parameter.


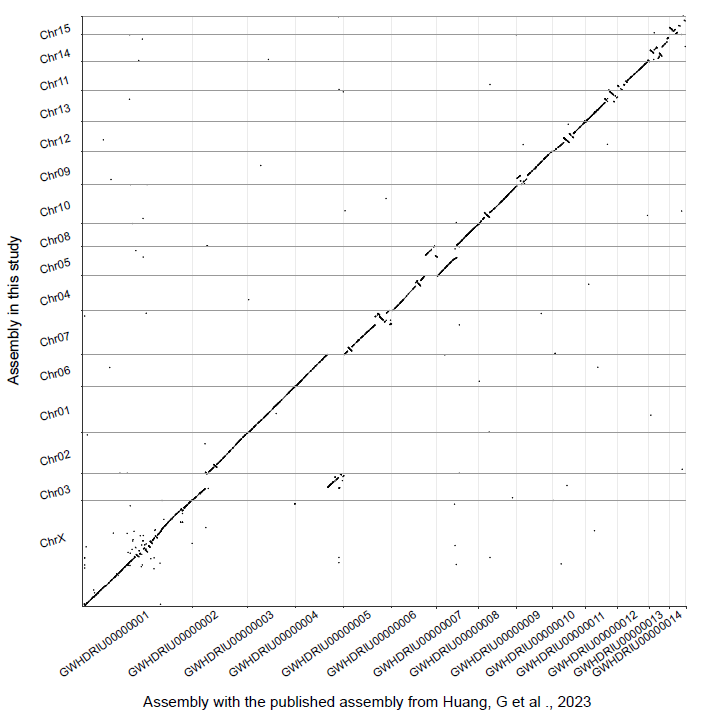


**Fig. S5. Syntenic comparison of our *D. truncata* assembly with the published assembly of *D. lobata* from Huang, G *et al.*, 2023.** The whole genome alignment is performed by minimap2 with “-x asm10” parameter.

***
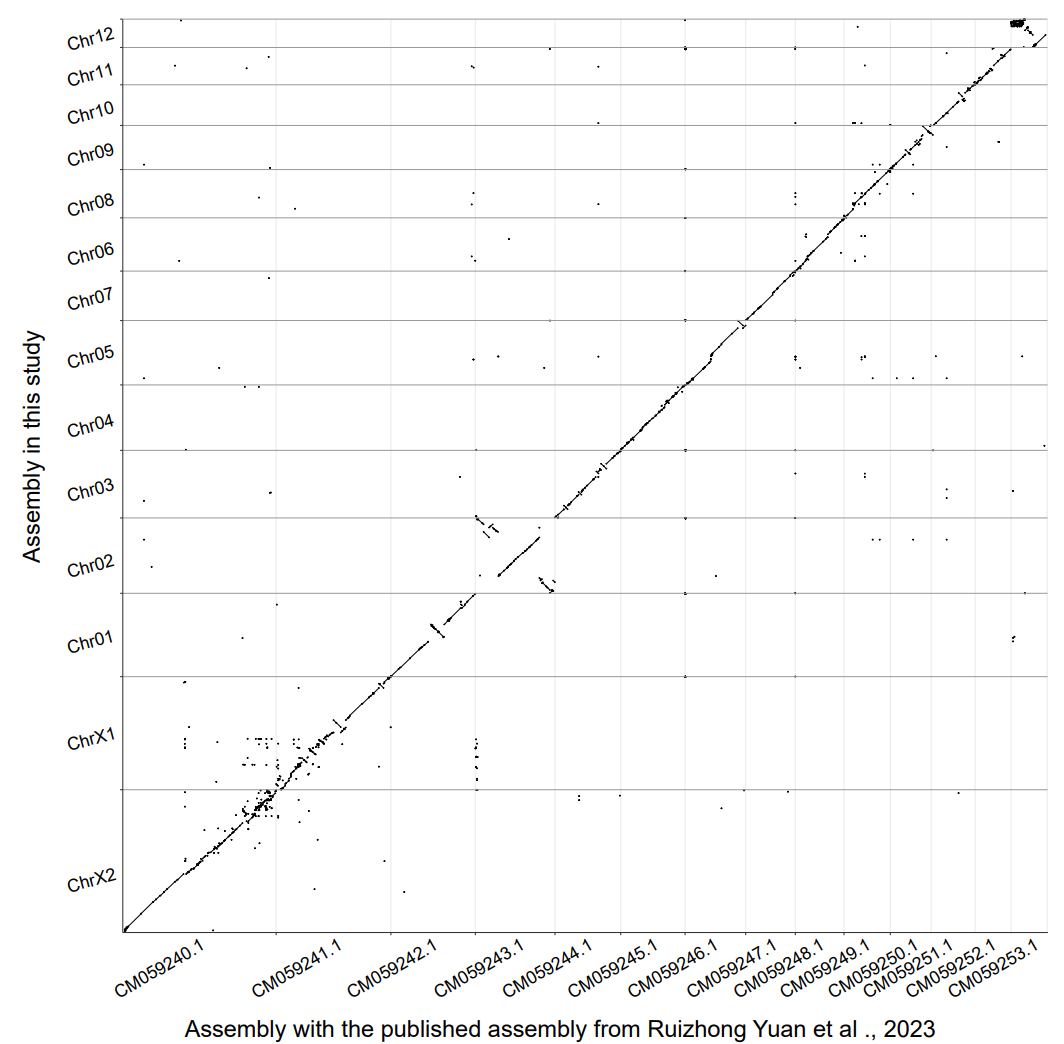
***

**Fig. S6. Syntenic comparison of our *T. sinensis* assembly with the published assembly from Ruizhong Yuan *et al.*, 2023.** The whole genome alignment is performed by minimap2 with “-x asm10”parameter.

**Figure S7.** The relationship between genome size and TE ratio of 5 Mantodea species. A linear trend Line in average of all the points was shown. The Pearson's correlation coefficient (r) is as high as 0.894, with t-test p≈0.038<0.05, suggesting that there is strong correlation between genome size and TE ratio.


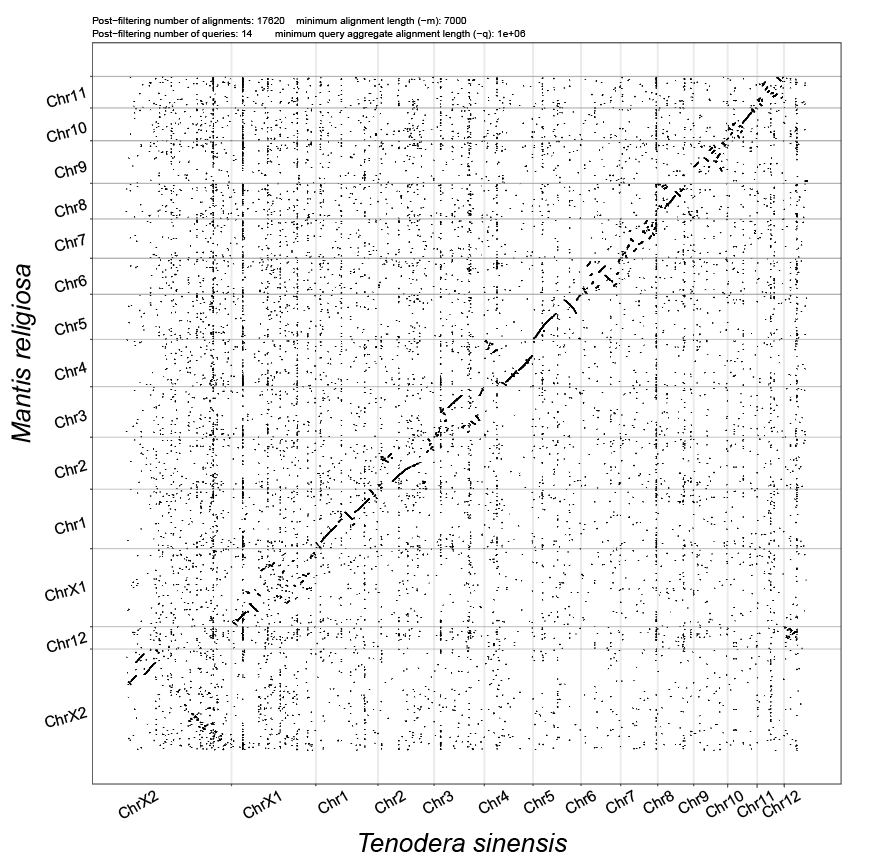


**
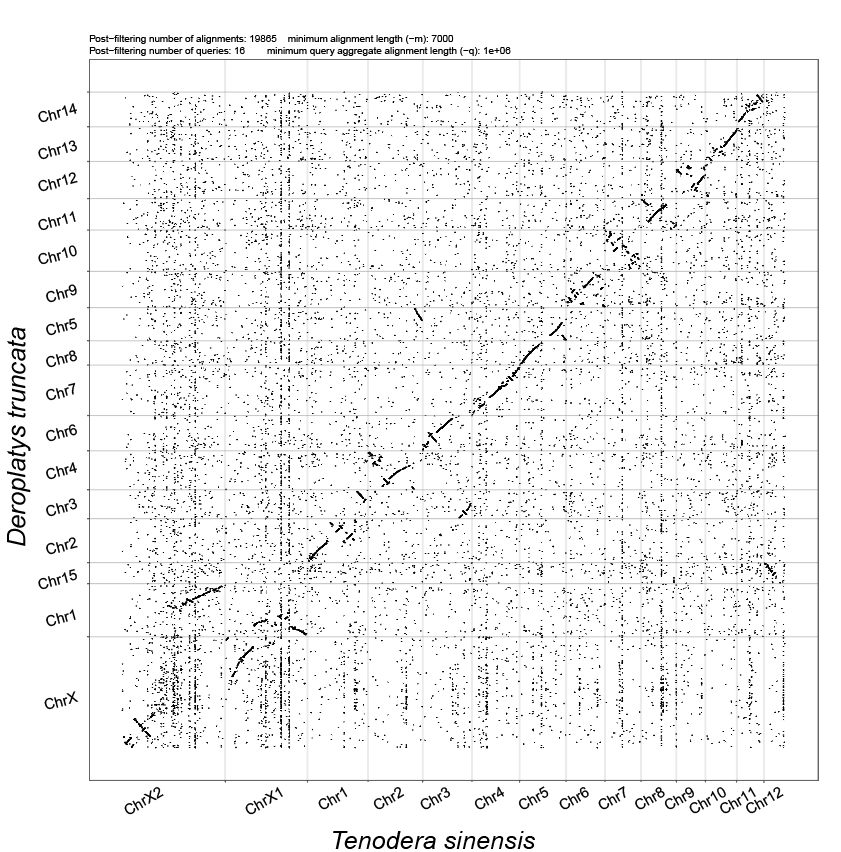
**

**
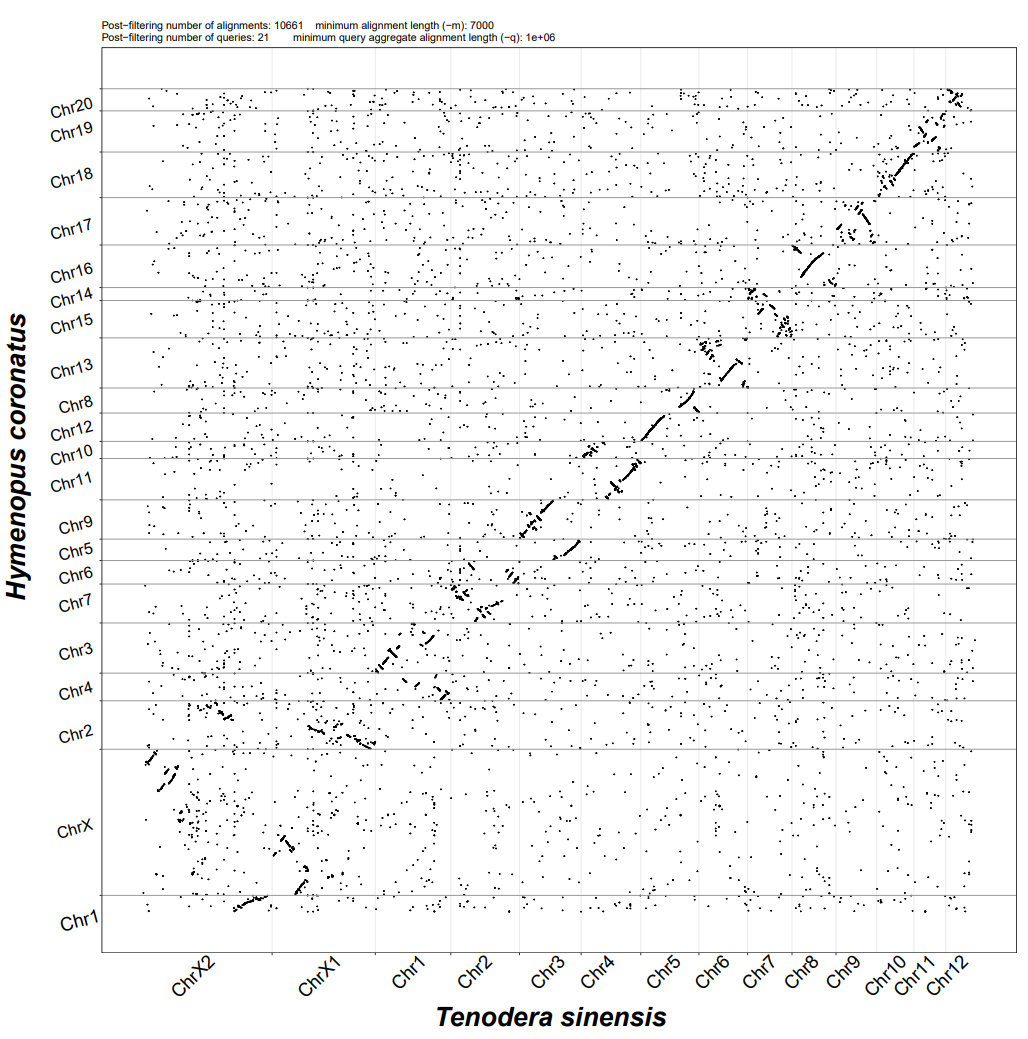
**

**
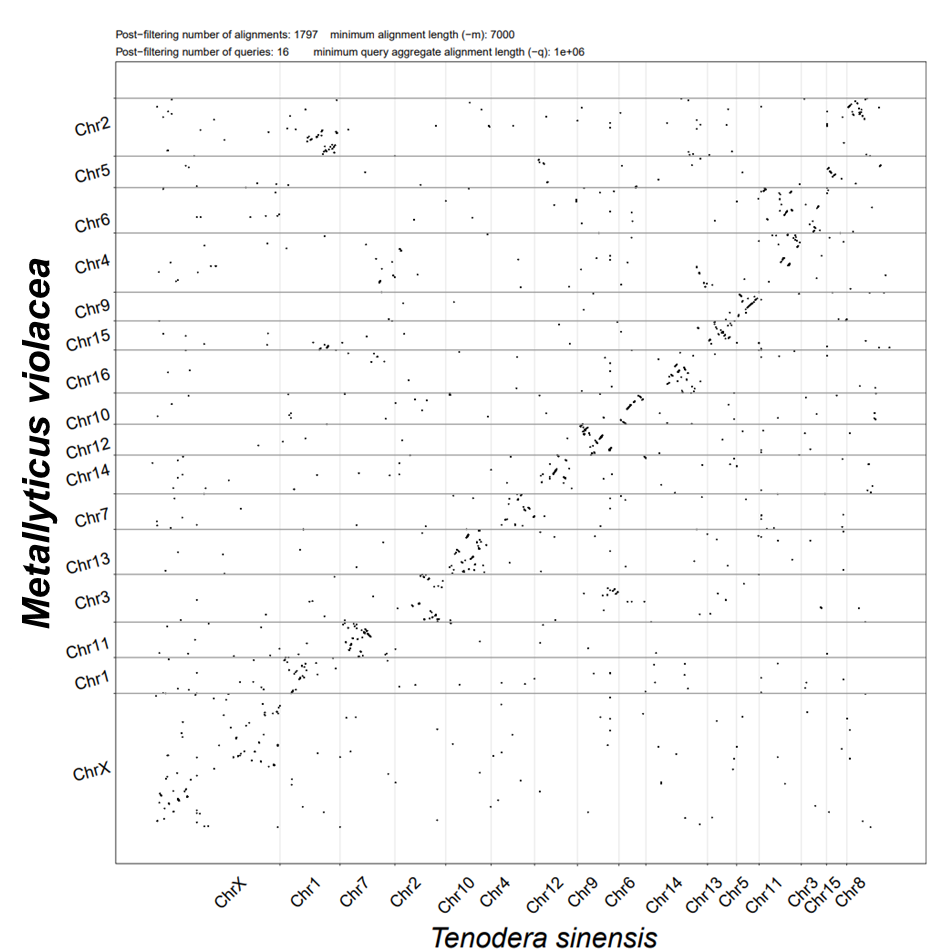
**

**Fig. S8. Syntenic comparison of *T. sinensis* with the other 4 mantises.** The whole genome alignment is performed by minimap2 with “-x asm10” parameter.


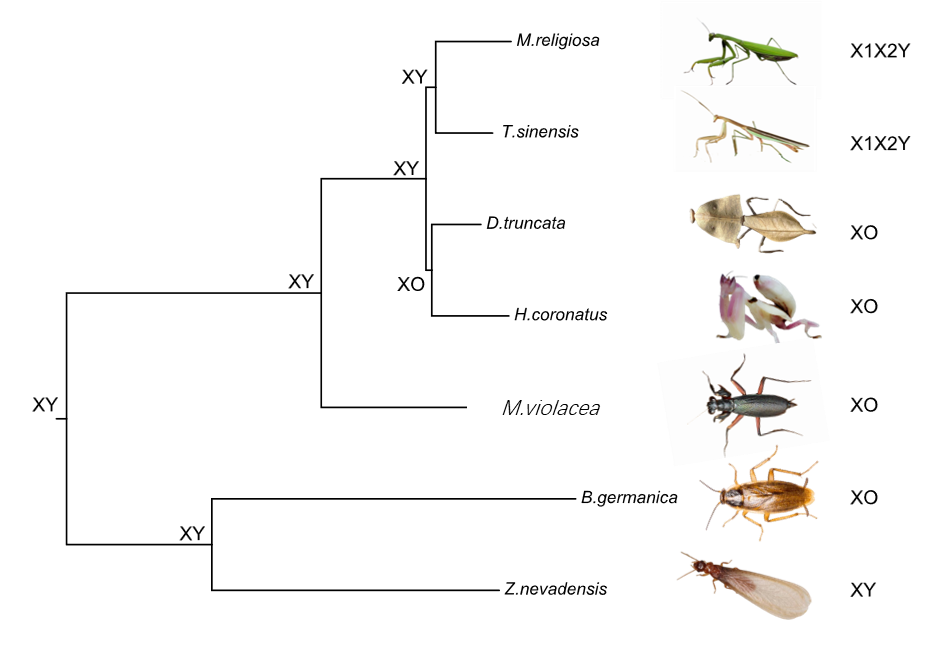


**Figure S9. Sex determination system of Mantodea (mantises) and Blattodea (cockroaches and termites).** The plot was drawn refer to “evobir.shinyapps.io/PolyneopteraDB/”**.**


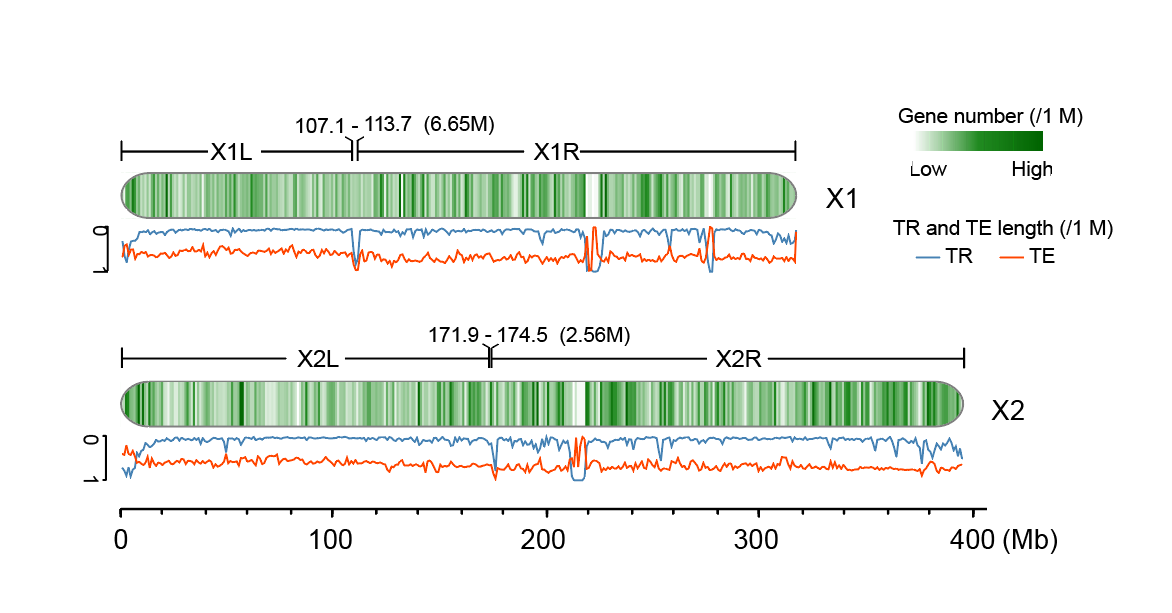


**Figure S10.** Location of the broken site for translocation on the X1 and X2 chromosomes of *T. sinensis*. TE refers to transposable element, and TR refers to tandem repeats. The gene density was shown in heatmap, while the TE/TR density were shown in distribution curves. Through macroscale synteny analysis between *T. sinensis* and *D. truncata* using reciprocal-best orthologous genes as markers, the broken site on ChrX1 (length 318,123,395 bp) was located to a 6.65 Mb region (107,057,327- 113,703,447 bp), and the broken site on ChrX2 (length 396,970,917 bp) was located to a 2.56 Mb region (171,940,730-174,498,078 bp). X1L and X2L were derived from ancestral X chromosome, while X1R and X2R were derived from ancestral autosome. Each broken site region has an assembly gap inside, which are filled with absolute 1,000 N characters. Therefore, the size estimation of broken site region was not so accurate. In addition, there are abundant TR and TEs in the broken site range, making it difficult to identify the accurate position where translocation happened. *M. religiosa* was not suitable for this analysis, because its current genome assembly is too fragmental.


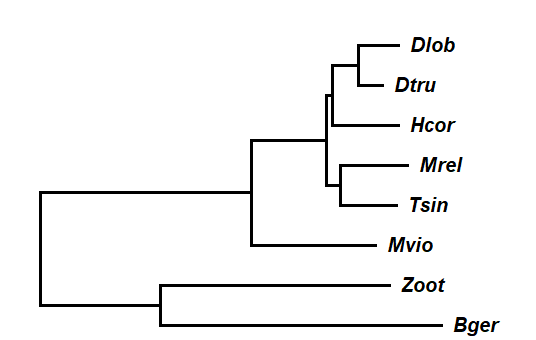


**Figure S11. Phylogeny tree for 8 Dictyoptera species.** The phylogenetic tree was built based on 2201 single-copy orthologs, with maximum likelihood methods (“raxmlHPC ­-m PROTGAMMALGX”). Dlob: *D.lobata,* Dtru: *D. truncata*, Hcor: *H. coronatus,* Mrel: *M. religiosa,*Tsin: *T. sinensis,* Mvio: *M. violacea ,*Zoot: *Z. nevadensis,* Bger*:* *B. germanica.*

**Supplementary tables**

**Table S1.** Background information of the sequenced mantis species.

| **Species name** | **Family / Sub-family** | **Chromosome number** | **source** |
| --- | --- | --- | --- |
| *Mantis religiosa* | Mantidae / Mantinae | 2n = 28 | China: Guangzhou |
| *Tenodera sinensis* | Mantidae / Mantinae | 2n = 28 | China: Guangzhou |
| *Deroplatys truncata* | Mantidae / Deroplatyinae | 2n = 32 | Malaysia: Cameron |
| *Hymenopus coronatus* | Hymenopoidea / Hymenopodidae | 2n = 42 | China: Xishuangbanna |
| *Metallyticus violacea* | Metallyticidae / Metallyticidae | 2n = 34 | Malaysia: Kuala Lumpur |

Note: The chromosome number of *M. religiosa* was obtained from del Cerro’s paper (Synaptonemal complex analysis of the X1X2Y trivalent in Mantis religiosa L. males: inferences on the origin and maintenance of the sex-determining mechanism. Chromosome Research. 1998;6:5-11), while the chromosome number of *T. sinensis* was obtained from Yuan R’paper (The chromosome-level genome of Chinese praying mantis Tenodera sinensis (Mantodea: Mantidae) reveals its biology as a predator. GigaScience. 2023;12). The chromosome number of *D. truncata*, *H. coronatus*, and *M. violacea* were determined by genome assembly, as no karyotype information was available for these 3 mantis species.

**Table S2.** Statistics of genome sequencing data.

|  | **Genomic Hifi (CCS)** | | **Genomic HiC** | | **Illumina RNAseq PE150** | |
| --- | --- | --- | --- | --- | --- | --- |
|  | **reads** | **bases** | **reads** | **bases** | **reads** | **bases** |
| *Mantis religiosa* | 10,736,312 | 179,683,809,522 | 847,343,050 | 127,101,457,500 | 618,200,522 | 92,730,078,300 |
| *Tenodera sinensis* | 4,901,197 | 97,511,748,151 | 749,492,290 | 112,423,843,500 | 487,921,752 | 73,188,262,800 |
| *Deroplatys truncata* | 7,744,738 | 112,906,676,547 | 1,023,722,878 | 153,558,431,700 | 624,484,224 | 93,672,633,600 |
| *Hymenopus coronatus* | 10,167,730 | 177,528,078,708 | 1,214,765,322 | 182,214,798,300 | 630,950,136 | 94,642,520,400 |
| *Metallyticus violacea* | 9,250,962 | 147,014,941,985 | 1,225,352,740 | 183,802,911,000 | - | - |

**Table S3.** Statistics of Hi-C data mapping to contigs.

| **HiC pro results** | ***Mantis religiosa*** | | ***Tenodera sinensis*** | | ***Deroplatys truncata*** | | ***Hymenopus coronatus*** | | ***Metallyticus violacea*** | |
| --- | --- | --- | --- | --- | --- | --- | --- | --- | --- | --- |
|  | **Reads number** | **Reads %** | **Reads number** | **Reads %** | **Reads number** | **Reads %** | **Reads number** | **Reads %** | **Reads number** | **Reads %** |
| Total pairs processed | 423,671,525 | 100.0% | 374,746,145 | 100.0% | 511,861,439 | 100.0% | 607,382,661 | 100.0% | 612,676,370 | 100.0% |
| Unmapped pairs | 20,914,316 | 4.9% | 31,898,335 | 8.5% | 5,258,539 | 1.0% | 8,315,587 | 1.4% | 26,580,086 | 4.3% |
| Low qual pairs | 157,153,623 | 37.1% | 148,457,384 | 39.6% | 201,124,421 | 39.3% | 213,630,868 | 35.2% | 141,182,216 | 23.0% |
| Pairs with singleton | 80,162,675 | 18.9% | 123,773,101 | 33.0% | 44,326,963 | 8.7% | 73,893,690 | 12.2% | 85,201,198 | 13.9% |
| Unique paired alignments | 165,440,911 | 39.0% | 70,617,325 | 18.8% | 261,151,516 | 51.0% | 311,542,516 | 51.3% | 359,712,870 | 58.7% |
| Valid interaction pairs | 85,602,445 | 20.2% | 56,106,670 | 15.0% | 228,045,907 | 44.6% | 273,688,263 | 45.1% | 129,362,070 | 21.1% |
| Dangling end pairs | 73,543,590 | 17.4% | 9,246,952 | 2.5% | 28,174,610 | 5.5% | 27,962,589 | 4.6% | 205,423,801 | 33.5% |
| Religation pairs | 3,169,697 | 0.7% | 860,676 | 0.2% | 1,493,478 | 0.3% | 4,059,892 | 0.7% | 16,514,464 | 2.7% |
| Self Cycle pairs | 141,033 | 0.0% | 60,031 | 0.0% | 418,528 | 0.1% | 455,795 | 0.1% | 3,822,912 | 0.6% |
| Filtered pairs | 2,978,737 | 0.7% | 4,328,894 | 1.2% | 3,016,583 | 0.6% | 5,365,973 | 0.9% | 4,562,422 | 0.7% |
| Dumped pairs | 5,409 | 0.0% | 14,102 | 0.0% | 2,410 | 0.0% | 10,004 | 0.0% | 27,201 | 0.0% |
| valid interaction | 85,602,445 | 20.2% | 56,106,670 | 15.0% | 228,045,907 | 44.6% | 273,688,263 | 45.1% | 129,362,070 | 21.1% |
| valid interaction rmdup | 71,796,880 | 16.9% | 43,658,431 | 11.7% | 181,375,815 | 35.4% | 202,919,881 | 33.4% | 34,648,667 | 5.7% |

Note: The statistics numbers were obtained from Hi-C pro result files: *.mpairstat, *.mRSstat, and * allValidPairs.mergestat. The “valid interaction rmdup” represents non-redundant and valid Hi-C read pairs, which were used by EndHiC for scaffolding.

**Table S4.** Statistics of genome assembly.

| **Species** | **Total contig size （bp)** | **Contig N50 size （bp)** | **Contig N90 size （bp)** | **Total scaffold size （bp)** | **Scaffold N50 size （bp)** | **Scaffold N90 size （bp)** | **% anchored to chromosomes** | **BUSCO results with insecta odb10** |
| --- | --- | --- | --- | --- | --- | --- | --- | --- |
| *Mantis religiosa* | 3,675,712,721 | 1,407,320 | 339,785 | 3,680,002,721 | 210,326,877 | 3,517,080 | 85.39% | C:98.7% [S:94.1%, D:4.6%] |
| *Tenodera sinensis* | 2,687,116,722 | 13,794,865 | 2,923,070 | 2,687,426,722 | 190,002,057 | 104,358,734 | 95.63% | C:99.3% [S:95.6%, D:3.7%] |
| *Deroplatys truncata* | 4,290,634,545 | 44,541,363 | 9,432,610 | 4,290,792,545 | 248,405,437 | 184,535,799 | 97.47% | C:98.6% [S:96.6%, D:2.0%] |
| *Hymenopus coronatus* | 3,127,524,514 | 71,519,735 | 15,255,482 | 3,127,590,514 | 159,059,693 | 82,661,183 | 98.27% | C:99.0% [S:97.7%, D:1.3%] |
| *Metallyticus violacea* | 2,322,120,794 | 109,157,195 | 61,664,404 | 2,322,129,794 | 125,733,329 | 88,780,966 | 98.51% | C:98.9% [S:97.4%, D:1.5%] |

Note: % anchored to chromosomes represents for percent of contig sequences assembled into chromosome-level scaffolds. In the BUSCO results, C means complete gene, S means single copy gene, while D means duplicated gene. S and D are both complete genes.

**Table S5.** Statistics of RNAseq mapping and assembly.

| **Species** | **Samples** | **Total RNAseq reads number** | **HiSAT2 mapping rate %** | **StringTie mRNA number** | **StringTie exon number** |
| --- | --- | --- | --- | --- | --- |
| *Mantis religiosa* | Sample_1 | 88,785,004 | 91.45% | 242,954 | 614,681 |
|  | Sample_2 | 86,154,304 | 91.46% |  |  |
|  | Sample_3 | 82,340,876 | 92.14% |  |  |
|  | Sample_4 | 87,545,542 | 92.31% |  |  |
|  | Sample_5 | 64,013,662 | 88.66% |  |  |
|  | Sample_6 | 79,818,902 | 88.58% |  |  |
|  | Sample_7 | 119,602,504 | 89.11% |  |  |
| *Tenodera sinensis* | Sample_1 | 67,885,312 | 94.91% | 24,901 | 180,176 |
|  | Sample_2 | 74,463,660 | 94.68% |  |  |
|  | Sample_3 | 69,051,400 | 94.34% |  |  |
|  | Sample_4 | 73,588,644 | 93.78% |  |  |
|  | Sample_5 | 70,815,820 | 94.53% |  |  |
|  | Sample_6 | 65,008,126 | 93.26% |  |  |
|  | Sample_7 | 67,108,790 | 93.57% |  |  |
| *Deroplatys truncata* | Sample_1 | 96,379,192 | 97.48% | 22,917 | 562,724 |
|  | Sample_2 | 76,125,082 | 97.21% |  |  |
|  | Sample_3 | 108,158,576 | 96.86% |  |  |
|  | Sample_4 | 86,689,652 | 97.34% |  |  |
|  | Sample_5 | 83,429,828 | 97.33% |  |  |
|  | Sample_6 | 80,351,926 | 97.16% |  |  |
|  | Sample_7 | 93,349,968 | 97.03% |  |  |
| *Hymenopus coronatus* | Sample_1 | 121,597,780 | 96.88% | 115,759 | 400,507 |
|  | Sample_2 | 81,361,518 | 96.95% |  |  |
|  | Sample_3 | 99,558,770 | 97.35% |  |  |
|  | Sample_4 | 79,018,200 | 96.95% |  |  |
|  | Sample_5 | 85,621,424 | 97.14% |  |  |
|  | Sample_6 | 78,941,540 | 96.95% |  |  |
|  | Sample_7 | 84,850,904 | 97.04% |  |  |

Note: The StringTie mRNA coordinates were converted into hints gff format, and used as the hints data in augustus gene prediction.

**Table S6.** Statistics of tandem repeats annotation.

| **Species** | **Total scaffold size (bp)** | **Total TR size (bp)** | **TR rate %** | **TR N50 size (bp)** |
| --- | --- | --- | --- | --- |
| *M. religiosa* | 3,680,002,721 | 396,842,330 | 10.8% | 3,188 |
| *T. sinensis* | 2,687,426,722 | 403,304,947 | 15.0% | 30,747 |
| *D. truncata* | 4,290,792,545 | 471,243,565 | 11.0% | 35,332 |
| *H. coronatus* | 3,127,590,514 | 238,530,960 | 7.6% | 6,018 |
| *M. violacea* | 2,322,129,794 | 186,949,249 | 8.1% | 2,372,101 |

Note: TR represents for “tandem repeat”.

**Table S7.** Statistics of TE content in various classes.

| **TE class** | ***Mantis religiosa*** | | ***Tenodera sinensis*** | | ***Deroplatys truncata*** | | ***Hymenopus coronatus*** | | ***Metallyticus violacea*** | |
| --- | --- | --- | --- | --- | --- | --- | --- | --- | --- | --- |
|  | **Length (bp)** | **Percent** | **Length (bp)** | **Percent** | **Length (bp)** | **Percent** | **Length (bp)** | **Percent** | **Length (bp)** | **Percent** |
| SINEs | 88,675,710 | 2.41% | 41,364,524 | 1.51% | 34,634,604 | 0.81% | 79,926,835 | 2.56% | 68,180,146 | 2.94% |
| Penelope | 179,087,096 | 4.87% | 46,171,123 | 1.69% | 40,398,887 | 0.94% | 56,917,120 | 1.82% | 65,477,245 | 2.82% |
| LINEs | 579,512,280 | 15.77% | 321,787,695 | 11.75% | 369,853,954 | 8.62% | 299,105,729 | 9.56% | 226,842,133 | 9.77% |
| LTR elements | 191,759,399 | 5.22% | 92,024,175 | 3.36% | 106,555,013 | 2.48% | 64,704,975 | 2.07% | 22,924,553 | 0.99% |
| DNA transposons | 775,059,780 | 21.09% | 521,921,270 | 19.06% | 1,039,790,255 | 24.23% | 1,023,083,723 | 32.71% | 516,358,740 | 22.24% |
| hobo-Activator | 80,830,494 | 2.20% | 120,355,965 | 4.39% | 72,581,984 | 1.69% | 53,762,543 | 1.72% | 62,477,753 | 2.69% |
| Tc1-IS630-Pogo | 487,487,116 | 13.26% | 307,146,368 | 11.22% | 743,125,454 | 17.32% | 904,769,393 | 28.93% | 391,997,649 | 16.88% |
| PiggyBac | 25,568,568 | 0.70% | 14,271,115 | 0.52% | 13,683,942 | 0.32% | 6,242,598 | 0.20% | 9,228,673 | 0.40% |
| Tourist/Harbinger | 3,509,956 | 0.10% | 6,656,014 | 0.24% | 10,579,236 | 0.25% | 4,453,869 | 0.14% | 2,199,720 | 0.09% |
| Other (Mirage, P-element, Transib) | 46,791,717 | 1.27% | 5,008,403 | 0.18% | 6,326,642 | 0.15% | 3,570,992 | 0.11% | 993,579 | 0.04% |
| Rolling-circles | 29,521,374 | 0.80% | 105,886,680 | 3.87% | 324,281,627 | 7.56% | 139,155,718 | 4.45% | 5,674,177 | 0.24% |
| Total interspersed repeats | 2,458,966,257 | 66.90% | 1,591,021,662 | 58.09% | 2,589,727,245 | 60.36% | 1,981,438,372 | 63.35% | 1,343,923,274 | 57.87% |

**Table S8.** Public genome data information.

| **Species Name** | **Ploidy** | **Sequencing technology** | **Genome Size (bp)** | **Contig N50 size** | **Assemly level / Scaffold N50 size** | **Data Address** | **Data version** |
| --- | --- | --- | --- | --- | --- | --- | --- |
| *Zootermopsis nevadensis* | 2n = 52 | Illumina HiSeq | 485,009,472 | 22.8 Kb | Scaffold level, 751.1 Kb | NCBI | ZooNev1.0 |
| *Blattella germanica* | 2n = 24 | Illumina | 2,037297555 | 12.1 Kb | Scaffold level, 1.1 Mb | NCBI | Bger_1.1 |
| *Deroplatys lobata* | 2n = 28 | nanopore 59.86 | 3,962,954,272 | 6.11 Mb | chromosome level, 285.05 Mb | CNCB | Dlob_genome_v1.0 |
