# Supplementary material for: The genomes of 5 mantises provide insights into sex chromosome evolution and Mantodea phylogeny clarification
Source: Gigascience. 2025 Dec 18;15:giaf158. doi: 10.1093/gigascience/giaf158 (PMC12908712; doi:10.1093/gigascience/giaf158)
Supplement: giaf158_GIGA-D-25-00308_original_submission [file giaf158_giga-d-25-00308_original_submission.pdf]

GigaScience

The genomes of five mantises provide insights into sex determination mechanism and evolution of Mantodea

--Manuscript Draft--

**Manuscript Number:**	GIGA-D-25-00308
**Full Title:**	The genomes of five mantises provide insights into sex determination mechanism and evolution of Mantodea
**Article Type:**	Research
**Funding Information:**	Shenzhen Science and Technology Program (KQTD20180411143628272) — Professor Guirong Wang; Fund of Key Laboratory of Shenzhen (ZDSYS20141118170111640) — Dr Wei Fan
**Abstract:**	Background Praying mantises, members of the order Mantodea, play important roles in agriculture, medicine, bionics, and entertainment. However, the scarcity of genomic resources has hindered extensive studies on mantis evolution and behaviour.<br>Results Here, we present the chromosome-scale reference genomes of five mantis species: the European mantis (Mantis religiosa), Chinese mantis (Tenodera sinensis), triangle dead leaf mantis (Deroplatys truncata), orchid mantis (Hymenopus coronatus), and metallic mantis (Metallyticus violaceus). We found that transposable element expansion is the major force governing genome size in Mantodea, and deduced that translocations between the X chromosome and an autosome have occurred in the lineage of the superfamily Mantidae. In addition, we found a much lower substitution rate for the lineage of M. violaceus than the lineages of other mantises. Furthermore, our genome-wide analyses showed that D. truncata is closer to H. coronatus than M. religiosa and T. sinensis, providing important evidences for resolving the phylogenic controversies of Deroplatys.<br>Conclusions The high-quality genome assemblies of the five mantises provide a valuable repository for evolution studies of Mantodea and genetic breeding of efficient enemy insect.
**Corresponding Author:**	Wei Fan<br>Chinese Academy of Agricultural Sciences<br>shenzhen, guangdong CHINA
**Corresponding Author Secondary Information:**	
**Corresponding Author's Institution:**	Chinese Academy of Agricultural Sciences
**Corresponding Author's Secondary Institution:**	
**First Author:**	Wei Fan
**First Author Secondary Information:**	
**Order of Authors:**	Wei Fan
	Guirong Wang
	Hangwei Liu
	Fan Jiang
	Hengchao Wang
	Bo Zhang
	Yutong Zhang
	Hanbo Zhao
**Order of Authors Secondary Information:**	

Powered by Editorial Manager® and ProduXion Manager® from Aries Systems Corporation

Additional Information:	
**Question**	**Response**
Are you submitting this manuscript to a special series or article collection?	No
**Experimental design and statistics**<br><br>Full details of the experimental design and statistical methods used should be given in the Methods section, as detailed in our Minimum Standards Reporting Checklist. Information essential to interpreting the data presented should be made available in the figure legends.<br><br>Have you included all the information requested in your manuscript?	Yes
**Resources**<br><br>A description of all resources used, including antibodies, cell lines, animals and software tools, with enough information to allow them to be uniquely identified, should be included in the Methods section. Authors are strongly encouraged to cite Research Resource Identifiers (RRIDs) for antibodies, model organisms and tools, where possible.<br><br>Have you included the information requested as detailed in our Minimum Standards Reporting Checklist?	Yes
**Availability of data and materials**<br><br>All datasets and code on which the conclusions of the paper rely must be either included in your submission or deposited in publicly available repositories (where available and ethically appropriate), referencing such data using a unique identifier in the references and in the “Availability of Data and Materials” section of your manuscript.	Yes

Powered by Editorial Manager® and ProduXion Manager® from Aries Systems Corporation

Have you have met the above requirement as detailed in our Minimum Standards Reporting Checklist?	
GigaScience has policies and guidelines in place for the use of generative AI-writing tools such as ChatGPT. If you have used such writing tools to assist with writing the manuscript this must be declared and cited in the text. Authors should not list AI-writing tools and other AI-assisted technologies as an author or co-author and should acknowledge that they are fully responsible for text generated or refined by AI-writing tools.<p><br><br>A summary of use (particularly in the introduction or among methods) needs to be included at the end of the paper, and the outputs should also be included as a supplementary file hosted in GigaDB or other open repositories. Please read our guidelines for more information. <p><br><br>By submitting to GigaScience, you are aware of the journal's AI-writing tools policy, and if you have declared use of such tools below, you have acknowledged this where appropriate in your manuscript and have made a summary of use and outputs available. <p><br><b>AI-assisted writing tools have been used in the preparation of this manuscript?	No

Maintext, tables, figure legends Click here to access/download;Manuscript;Manuscript.docx

The genomes of five mantises provide insights into sex determination mechanism and evolution of Mantodea

Hangwei Liu[1,2,†], Lihong Lei[1,3,4,†], Fan Jiang[1], Bo Zhang[1], Hengchao Wang[1], Yutong Zhang[3], Anqi Wang[1], Hanbo Zhao[1], Guirong Wang[1,5,*] & Wei Fan[1,*]

[1] Guangdong Laboratory for Lingnan Modern Agriculture (Shenzhen Branch), Genome Analysis Laboratory of the Ministry of Agriculture and Rural Affairs, Agricultural Genomics Institute at Shenzhen, Chinese Academy of Agricultural Sciences, Shenzhen, Guangdong, 518120, China.

[2] College of Plant Protection, Yangzhou University, Yangzhou 225009, China

[3] School of Life Sciences, Henan University, Kaifeng 475004, China

[4] Shenzhen Research Institute of Henan University, Shenzhen 518000, China

[5] State Key Laboratory for Biology of Plant Diseases and Insect Pests, Institute of Plant Protection, Chinese Academy of Agricultural Sciences, Beijing, China.

†These authors contributed equally to this work.

* Correspondence should be addressed to wangguirong@caas.cn and fanwei@caas.cn.

Abstract

Background Praying mantises, members of the order Mantodea, play important roles in agriculture, medicine, bionics, and entertainment. However, the scarcity of genomic resources has hindered extensive studies on mantis evolution and behaviour.

Results Here, we present the chromosome-scale reference genomes of five mantis species: the European mantis (*Mantis religiosa*), Chinese mantis (*Tenodera sinensis*), triangle dead leaf mantis (*Deroplatys truncata*), orchid mantis (*Hymenopus coronatus*), and metallic mantis (*Metallyticus violaceus*). We found that transposable element expansion is the major force governing genome size in Mantodea, and deduced that

translocations between the X chromosome and an autosome have occurred in the lineage of the superfamily Mantidae. In addition, we found a much lower substitution rate for the lineage of *M. violaceus* than the lineages of other mantises. Furthermore, our genome-wide analyses showed that *D. truncata* is closer to *H. coronatus* than *M. religiosa* and *T. sinensis*, providing important evidences for resolving the phylogenic controversies of *Deroplatys*.

Conclusions The high-quality genome assemblies of the five mantises provide a valuable repository for evolution studies of Mantodea and genetic breeding of efficient enemy insect.

Keywords: Mantodea, genome, transposable element, X1X2Y, evolution

Background

Praying mantises are familiar insects that play important roles in agriculture, medicine, and bionics. As predators of many harmful insect species, praying mantises such as the European mantis (*Mantis religiosa*) and Chinese mantis (*Tenodera sinensis*) are widely acknowledged as natural enemies that control plant pests[1], benefiting organic planting where pesticide is prohibited. The mantis ootheca (egg capsule, egg chamber) is a traditional medicine used to cure frequent micturition, strengthen kidney health and prevent spermatorrhea in East Asian countries [2]. Most praying mantises have two sharp and strong forelegs, which are much larger and more powerful than their ancient ancestors. In addition, the femur and tibia of the forelegs are armed with strong spines along their posterior edges. When the femur and tibia fold on each other, a praying mantis can firmly grasp the prey. This distinctive body structure of the praying mantis has been a significant source of inspiration in bionics of cutting blades [3, 4].

The two closely related orders, Mantodea (mantises) and Blattodea (cockroaches and termites), are classified into the superorder Dictyoptera, and phylogenomic analyses revealed that Mantodea split from Blattodea during the Permian [5]. Mantodea has evolved into a group comprising ~2500 species with diverse morphological and ecological characteristics, with the highest diversity in tropical and subtropical habitats

[6, 7]. From fossils of early Mantodea and Blattodea species, the common ancestor is thought to resemble modern cockroaches in many aspects [8]. The metallic mantis (*Metallyticus violaceus*), in the early diverging mantid lineage, has many morphological features similar to those of modern cockroaches [9]. Although Mantodea are well supported as monophyletic, the phylogenetic relationships within Mantodea are still not well resolved. Morphological and molecular phylogenies are often inconsistent with previous classification schemes for some sublineages such as *Deroplatys*, because of the rapid radiation events and convergent evolution of ecomorphological strategies [6, 10].

Compared with those of many other insect orders, the genomic resources of Mantodea are very limited, with only three chromosome-scale reference genomes available: the Chinese mantis (*T. sinensis*), orchid mantis (*H. coronatus*) and Malaysian dead leaf mantis (*Deroplatys lobata*) [11, 12]. Here, we present the chromosome-scale reference genomes of three other mantis species, the European mantis (*M. religiosa*), triangle dead leaf mantis (*D. truncata*), and metallic mantis (*M. violaceus*), as well as a more complete assembly of *T. sinensis* and *H. coronatus*, to promote the evolutionary and biological studies of Mantodea.

Results

Chromosome-scale genome assemblies of five mantis species

We generated 179 Gb (48X) (*M. religiosa*), 97 Gb (36X) (*T. sinensis*), 112 Gb (26X) (*D. truncata*), 177 Gb (56X) (*H. coronatus*), and 147 Gb (63X) (*M. violaceus*) PacBio HiFi data, and 127 Gb (35X) (*M. religiosa*), 112 Gb (42X) (*T. sinensis*), 153 Gb (35X) (*D. truncata*), 182 Gb (58X) (*H. coronatus*), and 183 Gb (79X) (*M. violaceus*) Illumina Hi-C data (Table S1, S2). The PacBio HiFi reads were used to assemble the contig sequences, with a total size of 3.6 Gb (*M. religiosa*), 2.6 Gb (*T. sinensis*), 4.2 Gb (*D. truncata*), 3.1 Gb (*H. coronatus*), and 2.3 Gb (*M. violaceus*) and N50 sizes of 1 Mb (*M. religiosa*), 13 Mb (*T. sinensis*), 44 Mb (*D. truncata*), 71 Mb (*H. coronatus*), and 109 Mb (*M. violaceus*). The Illumina Hi-C reads were mapped to the contig sequences, and the valid Hi-C read pairs were used for scaffolding assembly (Table S3), resulting in

85.39% (*M. religiosa*), 95.63% (*T. sinensis*), 97.47% (*D. truncata*), 98.27% (*H. coronatus*), and 98.51% (*M. violaceus*) of the contig sequences anchored into 14 (*M. religiosa*), 14 (*T. sinensis*), 16 (*D. truncata*), 21 (*H. coronatus*), and 17 (*M. violaceus*) chromosome-level scaffolds (Figure 1A-E, S1, S2; Table 1, S4). Notably, only the chromosome numbers for *M. religiosa* and *T. sinensis* have been karyotyped [13, 14], whereas the others are inferred only from the genome assembly. Using the GCE method [15], the estimated genome sizes are 3.5 Gb (*M. religiosa*), 2.8 Gb (*T. sinensis*), 4.3 Gb (*D. truncata*), 3.1 Gb (*H. coronatus*), and 2.3 Gb (*M. violaceus*), consistent with assembled genome sizes. Owing to the higher heterozygosity rate (Figure S3), the contig sizes for the *M. religiosa* and *T. sinensis* are shorter than those for the other three species.

Recently, Huang et al. published a reference genome for *H. coronatus*, with a much shorter contig N50 size of 15.7 Mb [11], and Yuan et al. published a reference genome of *T. sinensis* with a contig N50 size of 2.36 Mb, which is also much shorter than that of this study [12]. From syntenic alignments of the two assemblies for *H. coronatus*, we found that most chromosomes were largely consistent except for the X chromosome (Figure S4). One complete X chromosome in our assembly corresponds to 3 fragmented chromosomes in Huang's assembly. The X chromosome is the largest chromosome, making it more difficult to assemble than the autosomes. Thus, our assembly of the X chromosome for *H. coronatus* is likely more complete. We also compared another reference genome published by the Huang group [11], the Malaysian dead leaf mantis (*Deroplatys lobata*), to our assembled reference genome of *D. truncata* (Figure S5). Belonging to the same genus, most chromosomes have high synteny, except for four chromosomes involved in chromosome-level rearrangements, which are more likely due to species divergence than assembly errors. Both reference genomes of *T. sinensis* (Yuan et al.[12] and the present study) showed high synteny for all chromosomes (Figure S6).

By integrating homology and transcription evidence, 19,017 (*M. religiosa*), 19,007 (*T. sinensis*), 18,156 (*D. truncata*), 18,536 (*H. coronatus*) and 17,804 (*M. violaceus*) protein-coding gene models were annotated as the reference genes (Table 1, Table S5).

The BUSCO complete rates for the reference genes of these mantis species range from 96.7%-98.4% (Figure 1F), which are higher than or comparable to those of previously published mantis genomes [11] [12]. Furthermore, 97.2%-98.6% of the reference genes in these five mantis species were assigned functions according to at least one of the NCBI-NR, KEGG, InterPro or GO databases.

Distinct TE expansions in various mantid lineages

Increasing evidence has shown that transposable elements (TEs) contribute significantly to the genome size and influence the genome architecture, along with insertions, deletions, translocations, etc [16]. We analyzed the total TE content among the 5 species and found that genome size was linearly correlated with TE abundance (Figure S7). The two Mantidae species (*M. religiosa* and *T. sinensis*) have relatively smaller genome sizes (2.3-2.8 Gb) and lower TE contents (58-63%), than the other 3 mantises, with relatively larger genome sizes (3.1-3.5 Gb) and higher TE contents (67-68%), suggesting that genome size differences are mostly determined by TE contents in mantids.

Abundant retrotransposons, DNA transposons and rolling-circle transposons were found in these mantis genomes, however, their ratios in genome differ across species (Figure 2A, Table S6,S7). For the two Mantidae species, LINEs are the largest components, and a sharp expansion of LINEs with divergence of approx. 7% was found in *M. religiosa* (Figure 2B). However, no recent large-scale expansion of LINEs has occurred in *T. sinensis*, which may explain why its genome size (2.8 Gb) is smaller than that of *M. religiosa* (3.5 Gb). In contrast, *D. truncata* and *H. coronatus* have massive DNA transposons, with Tc1 (especially Tc1-IS630-Pogo) being the largest component in these two species, consistent with the findings of a former study [11]. *D. truncata* has undergone both a recent sharp expansion and an ancient burst of Tc1 in its genome, leading to the largest genome size (4.3 Gb) found in this study, whereas only an ancient explosion of Tc1 was observed in *H. coronatus* (Figure 2C). Both *D. truncata* and *H. coronatus* also have a large rolling-circle transposon, *Helitrons*. Both a recent and an ancient burst of *Helitron* were observed in *D. truncata*, whereas only an ancient burst

of *Helitron* was found in *H. coronatus* (Figure 2D). *M. violaceus* shows no recent accumulation of any category of TEs, which may explain why its genome size (2.3 Gb) was the smallest among these mantises.

These results collectively suggest that TE expansion is the major force behind genome size variation in Mantodea. In addition, the components and divergence times of the various TE types are distinct among the different mantid lineages.

Translocation between X chromosome and autosomes in Mantidae lineage

Sex chromosomes evolved from autosomes and play important roles in tissue development, mating, and speciation [17-19]. The types of sex chromosomes found in insects vary among species, and sex chromosome systems exhibit significant diversity across insect species. Most insects have XY, ZW or XO sex chromosome systems, but there are other rare sex chromosome types, such as the X1X2Y type, which has two X chromosomes and one Y chromosome. Some hemipterans such as *Philaenus italosignus* [20] and some mantids such as *M. religiosa* [21], exhibit this sex chromosome type.

To identify the X chromosomes from the assembled pseudochromosomes, we generated 15X short-read sequencing data for the female and male *T. sinensis* individuals, respectively. Sequencing coverage revealed that all 14 chromosomes in female have comparable coverage depths, whereas in male, two chromosomes have approximately half the coverage depth (Figure 3A). It has been reported that most members of the superfamily Mantidae have two X chromosomes, X1 and X2, derived from fusion or translocation between the X chromosome and an autosome [13]. Thus, the two chromosomes with half coverage depths are concluded to be the X1 and X2 chromosomes. Notably, they are the largest and second largest of our assembled pseudochromosomes, consistent with previous reports based on karyotyping [13, 22].

Macroscale synteny analysis identified the corresponding X chromosomes in the other 4 species, and allowed comparative analysis among the mantid X chromosomes. Synteny alignments revealed that both Mantidae species *M. religiosa* and *T. sinensis* have two sex chromosomes, X1 and X2; however, the other species have only one sex

chromosome X. In addition, only part of X1 (X1L) and X2 (X2L) in Mantidae were aligned with the X chromosomes of the other 3 species (Figure 3B-D, S8). These results suggest that the ancestral mantid had one X chromosome and that the translocation of large fragments between the X chromosome and an autosome occurred in Mantidae (Figure 3E). Previous studies have revealed that the common ancestor of Dictyoptera had a XY sex chromosome system [13, 22] (Figure S9). We inferred that the common ancestor of the Mantidae family evolved the X1X2Y sex chromosome system, and we confirmed that the generation of the X1 and X2 chromosomes resulted from the fragmentation and fusion of one X chromosome and an autosome.

Furthermore, based on conserved sequence alignments, we were able to identify the breakpoint range as a site falling within a 2.5-Mb region on the X chromosome (Figure 3F, S10). Resolution by sequencing is much greater than that previously obtained via cytological techniques, such as C-banding, silver staining and living-cell images of the meiosis process [13, 22].

Low evolutionary rate allows *M. violaceus* conserve more ancestral traits

Comparative analysis of Mantodea genomes within a phylogenetic context is essential for understanding their evolution and diversity. Phylogenomic analyses were performed on these 5 Mantodea species, which span 5 genera and 3 families with diverse habitats and morphologies. Two Blattodea species, the German cockroach (*Blattella germanica*) [23] and the dampwood termite (*Zootermopsis nevadensis*) [24], were used as the outgroup (Table S8). From gene family clustering, 69,603 orthologous groups (OGs) were generated, including 4,014 single-copy OGs.

M. violaceus belongs to the superfamily Metallyticoidea and shares many characteristics with its cockroach relatives but exhibits significant morphological differences compared to other mantises, including dull body colouration, a prostrate body posture, and a relatively shorter prothorax. The Metallyticoidea lineage is sister to the other mantis lineages [25]. *M. violaceus* shares much more OGs with cockroaches than the other 4 species (69% vs 61-63%) (Figure 4A), which may partly explain its strong morphological resemblance to cockroaches.

Phylogenetic tree was constructed based on 4,014 single-copy OGs, and the substitution rates along branches were estimated. The substitution rate for *M. violaceus* branch was the lowest among those of Mantodea (Figure 4B), which may indicate that the evolution rate of *M. violaceus* branch was much slower than that of the other mantises. With a slower evolution rate, *M. violaceus* may preserve more characteristics of the Mantodea ancestor, which also partly explains its morphological resemblance to cockroaches.

Phylogenetic tree also revealed that *D. truncata* is closer to *H. coronatus* (Hymenopodidae) than *M. religiosa and T. sinensis* (Mantidea), differing from the phylogenic assignment from some previous studies that place *D. truncata* within Mantidea (Figure 4B, S11, S12) [6, 10]. After adding the genomic data for *D. lobata*, both Deroplatys species sistered to *H. coronatus* (Figure S13). Therefore, genome-wide data is helpful to clarify phylogeny controversies, providing important evidences for further species classification of Deroplatys.

Discussion

In this study, we generated chromosome-level genome assemblies for 5 mantis species via a combination of PacBio HiFi and Hi-C sequencing technologies. For *H. coronatus* and *T. sinensis*, both the contig N50 and N90 sizes of our assembly are approximately 5 times greater than those of the previously published reference genomes [11, 12]. In our results, assembly continuity for *M. religiosa* and *T. sinensis* is relatively lower than that for the other 3 mantises due to the differences in heterozygosity, suggesting that tissues with no or low heterozygosity are still a priority for *de novo* genome sequencing. Compared with those of cockroaches and termites, the much larger genome sizes of mantises are mainly the result of expansions of various types of transposable elements.

Mantodea has occupied an important position in the evolution of insects, and one of its major sublineages, the superfamily Mantidae, has a special X1X2Y sex determination system. Through comparative genomics analysis, we inferred that the mantid common ancestor had only one X chromosome and translocation between the

X chromosome and an autosome occurred in the ancestor of Mantidae. We were able to narrow this breakpoint to less than a 2.5-Mb range on the original X chromosome, which will promote studies of sex determination systems and chromosome evolution. *M. violaceus* genome shares more orthologous genes with cockroaches than with the other mantises, and the mutation rate of *M. violaceus* genome is the lowest among all the mantises, allowing *M. violaceus* to conserve more ancestral traits. Our phylogenetic analyses with genome-wide data also suggest that the two *Deroplatys* species are closer to *H. coronatus* than to the two Mantidea mantises, which may do some help for further phylogeny clarification and accurate species classification of *Deroplatys*.

Although praying mantises are efficient predators, their hunting objects are not specific to harmful insects, hindering their wide application in organic planting. Thus, the genomic resources generated in this study will also facilitate molecular breeding of the praying mantis, in order to make it a more applicable nature enemy insect.

Methods

Insect collection and sequencing

Mantis adults were collected at different locations: *M. religiosa* and *T. sinensis* from the forest of Guangzhou, China; *H. coronatus* from the rainforest of Xishuangbanna, China; and *D. truncata* and *M. violaceus* from two captive breeding centers in Beijing, China. All mantis samples for sequencing had the intestine removed to avoid contamination by bacteria, fungi, and residual prey bodies. All the tissues were cleaned with 30% ethanol and ddH_2O, and then immersed in liquid nitrogen for cryopreservation.

For Pacific Biosciences (PacBio) HiFi sequencing, libraries with ~15 kb insert sizes were constructed from a female adult of every mantis, and sequenced on a PacBio Sequel II system (RRID: SCR_017990). Subreads were generated with an N50 size of 14.5 kb, and consensus reads (CCS reads) were generated via ccs software (v.3.0.0) [26] with the following parameters: -min-passes 0 -min-rq 0.99 -min-length 100 -max-length 50,000. For Illumina sequencing of a male adult and a female adult of *T. sinensis*, two short paired-end DNA library with a 400 bp insert size was constructed via standard

Illumina protocols respectively, and sequenced on an Illumina NovaSeq 6000 platform (RRID:SCR_016387).

Total RNA from the head, eye, thorax, abdomen, forefoot, midfoot and midfoot of female adults was extracted with TRIzol reagent (Invitrogen) and used to construct cDNA libraries. Transcriptome sequencing data were generated via the Illumina NovaSeq 6000 system in PE150 mode.

Genome assembly and quality assessment

K-mer frequencies from HiFi reads of five mantises were calculated via Kmerfreq (https://github.com/fanagislab/kmerfreq), and then genome sizes were estimated via GCE (GCE, RRID:SCR_017332). The PacBio HiFi reads were assembled into contigs via Hifiasm (v0.14) (Hifiasm, RRID:SCR_021069)[27] with the following parameters: -l 1 -s 0.7. To filter duplicated contigs in the assembly, purge_dups (v1.2.3) (purge dups, RRID:SCR_021173) [26] was adopted with the following parameters: -2 -a 50. The completeness of the assembly was evaluated using BUSCO (v5.2.2) (BUSCO, RRID:SCR_015008) based on the OrthoDB (v10) (OrthoDB, RRID:SCR_011980) Insecta database [28].

For Hi-C scaffolding, two strategies were applied. For *M. religiosa*, whose contigs are more fragmental, Hi-C reads were mapped to contigs via the Arima mapping pipeline (https://github.com/ArimaGenomics/mapping_pipeline), and then, YaHS (v1.2a.1) (YaHs, RRID:SCR_0229650) [29] was used to assemble the contigs into pseudo chromosomes. For the other four mantises, whose contigs are much larger, Hi-C reads were mapped to contigs by Bowtie 2 (v 2.2.2.7) (Bowtie 2, RRID:SCR_016368) [30], then HiC-Pro (v2.11.0-beta) (HiC-Pro, RRID:SCR_017643) [31] was adopted to identify valid ligation pairs and generate Hi-C link matrices among different contigs, and finally, the contigs were clustered, ordered, and oriented into pseudo-chromosomes using EndHiC (v1.0) (EndHiC, RRID:SCR_022110) [32] based on the Hi-C linkage information among contig ends.

Genome annotation

A *de novo* transposable element (TE) library was constructed with RepeatModeler (v2.0.2) (RepeatModeler, RRID:SCR_015027) with the parameters -engine ncbi-database [33], and then RepeatMasker (v4.1.0) (RepeatMasker, RRID:SCR_012954) was used to identify TEs in the reference genome, using both the *de novo* TE library and the public Repbase TE library (v26.05) (Repbase, RRID:SCR_021169). The tandem repeat elements in the genome were subsequently identified using Tandem Repeats Finder (TRF) (Tandem Repeats Finder, RRID:SCR_022193) (v4.09) [34].

The protein-coding gene models were annotated in two rounds. In the first round, the genes were predicted by integrating evidence from *de novo* gene predictions and transcriptome-based gene predictions. *De novo* gene prediction was performed on the TE-masked genome assembly with AUGUSTUS (v3.4.0) (Augustus, RRID:SCR_008417) [35]. For transcriptome-based gene prediction, the RNA-seq data were filtered by Fastp (v0.23.1) (fastp, RRID:SCR_016962) [36]and then mapped to the genome using Bowtie2 (v2.2.7) [30], and StringTie (v1.3.3b) (StringTie, RRID:SCR_016323) was then used to construct the gene models [37]. All the gene models obtained via the above two approaches were subsequently integrated with EVidenceModeler (v1.1.1) (EVidenceModeler, RRID:SCR_014659) [38]. In the second round, for each mantis, the protein sequences from the other 4 mantises were mapped to this genome assembly with Exonerate (v2.4.0) (Exonerate, RRID:SCR_016088) [39], and incomplete gene models were filtered. Finally, for each mantis, the *de novo* gene predictions, the transcriptome-based gene predictions, and the homology-based gene predictions were integrated with EVidenceModeler (v1.1.1) to generate a high-confidence and nonredundant gene set.

The completeness of the gene sets was assessed using BUSCO based on OrthoDB (v10) for Insecta. For gene functional annotation, the mantis protein sequences were aligned to the KEGG (KEGG, RRID:SCR_012773), eggNOG (eggNOG, RRID:SCR_002456), NR, and UniProt (SwissProt) databases using DIAMOND (v0.9.24.125) (DIAMOND, RRID:SCR_009457) [40], and only the best hits with E-values less than $1e^{-5}$ were retained. Moreover, InterProScan (v5.38) (InterProScan, RRID:SCR_005829) was used to annotate the protein domains and GO (Gene Ontology)

terms [41].

X chromosome identification and analysis

To identify the X chromosome, the clean Illumina paired reads from female and male samples were mapped to the genome of *T. sinensis* via BWA (v0.7.17-r1188) (BWA, RRID:SCR_010910) [42]. The bam files were filtered using SAMtools (v1.6) (SAMTOOLS, RRID:SCR_002105) [43] with the parameters '-q 60 -F 1804', and paired reads mapped onto different chromosomes were also filtered. To assess the sequencing depth of each chromosome, SAMtools depth (v1.6) was used to calculate the average base coverage. The two chromosomes in males whose sequencing depth was approximately half that of the other chromosomes, were identified as X-derived chromosomes. For consistency with the karyotype results for *T. sinensis* [21] and *M. religiosa* [13], the larger one was denoted X2, whereas the smaller one was denoted X1.

Pairwise collinearity analyses were conducted using the protein sequences of five mantis species as markers. DIAMOND (v0.9.24.125) with the parameters 'blastp -f 6' was used to align the protein sequences of each species pair, and the reciprocal best pairs were used as inputs for MCScanX (MCScanX, RRID:SCR_022067) to identify syntenic blocks [44]. The inter species syntenic genomic blocks were visualized via the R package Ideogram [45]. Based on the collinearity alignments of the five mantises, the X chromosomes of the other four mantises were also identified. In addition, the translocation sites on chromosomes X1 and X2 were inferred from the collinearity alignment.

Evolutionary analysis

Seven Dictyoptera species, including the five mantises sequenced in this study, as well as *B. germanica* [23] and *Z. nevadensis* [24], were used to infer orthologous genes via OrthoFinder (v2.5.4) (OrthoFinder, RRID:SCR_017118) with the default parameters [46]. The protein sequences of single-copy genes from each species were multiple aligned using MAFFT (v7.487) (MAFFT, RRID:SCR_011811) and then concatenated into one super protein sequence. Using the concatenated super protein sequence,

RAxML (v8.2.12) (RAxML, RRID:SCR_006086) was subsequently employed to construct a maximum-likelihood phylogenetic tree with the PROTGAMMALGX model, and codon alignment of the super protein sequence were used to construct a maximum-likelihood phylogenetic tree with the GTRGAMMA model [47].

Abbreviations

BLAST: Basic Local Alignment Search Tool; bp: base pairs; BUSCO: Benchmarking Universal Single-Copy Orthologs; BWA: Burrows-Wheeler Aligner; CCS: circular consensus sequencing; Gb: gigabase pairs; GO: Gene Ontology; kb: kilobase pairs; KEGG: Kyoto Encyclopedia of Genes and Genomes; Ma: megaannus; Mb: megabase pairs; MYA: million years ago; NCBI: National Center for Biotechnology Information; NR: Non-Redundant; OG: orthologous groups; PacBio: Pacific Biosciences; PE: Paired end; RAxML: Randomized Axelerated Maximum Likelihood; TRF: Tandem Repeats Finder; TE: transposable element; TPM: transcripts per million; YaHS: yet another Hi-C scaffolding.

Acknowledgments

This work was supported by Shenzhen Science and Technology Program (Grant No. KQTD20180411143628272) and Projects subsidized by Special Funds for Science Technology Innovation and Industrial Development of Shenzhen Dapeng New District (Grant No. PT202101-02); Fund of Key Laboratory of Shenzhen (ZDSYS20141118170111640) and The Agricultural Science and Technology Innovation Program.

Data availability

The genomic and transcriptomic sequencing reads have been deposited in NCBI-SRA under the accession PRJNA987019, PRJNA989593, PRJNA989036, PRJNA988270, PRJNA989282 for *M. religiosa*, *T. sinensis*, *D. truncata*, *H. coronatus* and *M. violaceus*, respectively. The corresponding genome assemblies and annotations have been deposited at NCBI-Genome under the accessions JAUKNK000000000,

JAUKNM000000000, JAUKNL000000000, JAUKNX000000000, JAUJEO000000000, and are also available at Figshare (10.6084/m9.figshare.23995398, 10.6084/m9.figshare.23995410, 10.6084/m9.figshare.23995152, 10.6084/m9.figshare.23988987, 10.6084/m9.figshare.23995434).

Author contributions

H.L. and L.L. prepared the sequencing samples, performed data analysis, and wrote the raw manuscript. W.F. and G.W. supervised the project and revised the manuscript. The other authors provided helpful suggestions, and all authors read and approved the final version of this manuscript.

Competing interests

The authors declare no competing interest.

Figures and tables

Figure 1. **Overall view of genome assembly and annotation**. Circos plots for *M. religiosa.* (A) *T. sinensis* (B) *D. truncata* (C) *H. coronatus* (D) and *M. violaceus* (E). Each circos plot has 4 tracks: track A represents chromosome length, track B represents gene density, track C represents transposable element (TE) density, and track D represents GC percentage. Feature density and GC percentage were calculated by sliding 1-Mb windows. (F) BUSCO assessment (database: Insecta from OrthoDB v10) of gene sets for five mantis species. M means missing, F means fragmented, and C means complete.

Figure 2. **TE distribution in five mantis genomes.** (A) The radar chart for major components of TE. (B) The divergence (%) distribution of LINE. (C) The divergence (%) distribution of Tc1. (D) The divergence (%) distribution of *Helitron*.

Figure 3. **Evolution of X chromosome in Mantodea.** (A) Identification of X chromosome in *T. sinensis* by comparing depths between male and female individual. The sequencing depth distributions were plotted in 500 Kb windows. The red line represents the average sequencing depth for each chromosome. (B) The synteny band plot among *T. sinensis*, *D. truncata* and *M.*

violaceus. (C) The dual synteny between *M. religiosa* and *T. sinensis*. (D) The dual synteny between *D. truncata* and *H. coronatus*. (E) The diagram shows the evolutionary process of the X chromosome along various lineages of Mantodea. (F) The range of the broken site for translocation on the X1 and X2 chromosomes of *T. sinensis*, which are 2.51 Mb and 0.02 Mb, respectively.

Figure 4. **Orthologous groups and phylogeny of Mantodea**. (A) Percentage of orthologous groups (OG) shared with cockroach for each mantis species. (B) Phylogeny is based on codon alignment of single copy genes (mantises, cockroaches, and termites) with Maximum Likelihood (ML) method. The branch length is in proportional with the substitution rate.

Table 1. Statistics of genome assembly and annotation

**Genomic features**	***M. religiosa***	***T. sinensis***	***D. truncata***	***H. coronatus***	***M. violaceus***
**Genome assembly**					
Estimated genome size by K-mer (bp)	**3,519,843,697**	**2,865,686,147**	**4,337,798,490**	**3,167,239,197**	**2,331,221,057**
Total assembly size (bp)	**3,680,002,721**	**2,687,426,722**	**4,290,792,545**	**3,127,590,514**	**2,322,129,794**
Contig N50 size (bp)	**1,407,320**	**12,728,340**	**44,444,664**	**71,519,735**	**109,157,195**
Scaffold N50 size (bp)	**210,326,877**	**190,002,057**	**248,405,437**	**159,059,693**	**125,733,329**
# of assembly-inferred chromosomes	**14**	**14**	**16**	**21**	**17**
% sequence anchored to chromosome	**85.39%**	**95.63%**	**97.47%**	**98.27%**	**98.51%**
**Genome annotation**					
Length and % of tandem sequences (bp)	**396,842,330 (10.8%)**	**403,304,947 (15.0%)**	**471,243,565 (11.0%)**	**238,530,960 (7.6%)**	**186,949,249 (8.1%)**
Length and % of TE sequences (bp)	**2,501,898,483 (68%)**	**1,710,668,926 (64%)**	**2,928,636,453 (68%)**	**2,122,785,940 (68%)**	**1,351,077,317 (58%)**
Number of protein-coding gene models	**19,017**	**19,007**	**18,156**	**18,536**	**17,804**
Mean CDS length (bp)	**1551**	**1782**	**1601**	**1523**	**1152**
Mean exon number	**6.07**	**5.93**	**6.34**	**6.33**	**5.54**

References

1. Rankin EEW, Shmerling AJ, Knowlton JL and Hoey-Chamberlain R. Diets of two non-native praying mantids (Tenodera sinensis and Mantis religiosa) show consumption of arthropods across all ecological roles. Food Webs. 2023;35 doi:ARTN e00280
10.1016/j.fooweb.2023.e00280.

2. Song JH, Cha JM, Moon BC, Kim WJ, Yang S and Choi G. Mantidis Ootheca (mantis egg case) original species identification via morphological analysis and DNA barcoding. J Ethnopharmacol. 2020;252 doi:ARTN 112574
10.1016/j.jep.2020.112574.

3. Yu HY, Han ZW, Zhang JQ and Zhang SJ. Bionic design of tools in cutting: Reducing adhesion, abrasion or friction. Wear. 2021;482 doi:ARTN 203955
10.1016/j.wear.2021.203955.

4. Li M, Yang YW, Guo L, Chen DH, Sun HL and Tong J. Design and Analysis of Bionic Cutting Blades Using Finite Element Method. Appl Bionics Biomech. 2015;2015 doi:Artn 471347
10.1155/2015/471347.

5. Evangelista DA, Wipfler B, Bethoux O, Donath A, Fujita M, Kohli MK, et al. An integrative phylogenomic approach illuminates the evolutionary history of cockroaches and termites (Blattodea). Proc Biol Sci. 2019;286 1895:20182076. doi:10.1098/rspb.2018.2076.

6. Svenson GJ and Whiting MF. Reconstructing the origins of praying mantises (Dictyoptera, Mantodea): the roles of Gondwanan vicariance and morphological convergence. Cladistics. 2009;25 5:468-514. doi:10.1111/j.1096-0031.2009.00263.x.

7. Svenson GJ, Hardy NB, Wightman HMC and Wieland F. Of flowers and twigs: phylogenetic revision of the plant-mimicking praying mantises (Mantodea: Empusidae and Hymenopodidae) with a new suprageneric classification. Syst Entomol. 2015;40 4:789-834. doi:10.1111/syen.12134.

8. Hornig MK, Haug JT and Haug C. An exceptionally preserved 110 million years old praying mantis provides new insights into the predatory behaviour of early mantodeans. PeerJ. 2017;5:e3605. doi:10.7717/peerj.3605.

9. Fukui M, Fujita M, Tomizuka S, Mashimo Y, Shimizu S, Lee CY, et al. Egg structure and outline of embryonic development of the basal mantodean, Metallyticus splendidus Westwood, 1835 (Insecta, Mantodea, Metallyticidae). Arthropod Struct Dev. 2018;47 1:64-73. doi:10.1016/j.asd.2017.11.001.

10. Ma Y, Zhang LP, Lin YJ, Yu DN, Storey KB and Zhang JY. Phylogenetic relationships and divergence dating of Mantodea using mitochondrial phylogenomics. Syst Entomol. 2023; doi:10.1111/syen.12596.

11. Huang G, Song L, Du X, Huang X and Wei F. Evolutionary genomics of camouflage innovation in the orchid mantis. Nat Commun. 2023;14 1:4821. doi:10.1038/s41467-023-40355-1.

12. Yuan R, Zheng B, Li Z, Ma X, Shu X, Qu Q, et al. The chromosome-level genome of Chinese praying mantis Tenodera sinensis (Mantodea: Mantidae) reveals its biology as a predator. GigaScience. 2023;12 doi:10.1093/gigascience/giad090.

13. del Cerro AL, Cunado, N. & Santos, J.L. Synaptonemal complex analysis of the X1X2Y trivalent in Mantis religiosa L. males: inferences on the origin and maintenance of the sex-determining mechanism. Chromosome Research. 1998;6:5-11.

doi:https://doi.org/10.1023/A:1009258122785.
14. Li XT and Nicklas RB. Mitotic Forces Control a Cell-Cycle Checkpoint. Nature. 1995;373 6515:630-2. doi:DOI 10.1038/373630a0.
15. Liu B, Shi Y, Yuan J, Hu X, Zhang H, Li N, et al. Estimation of genomic characteristics by analyzing k-mer frequency in de novo genome projects. arXiv: Genomics. 2013.
16. Elliott TA and Gregory TR. Do larger genomes contain more diverse transposable elements? Bmc Evol Biol. 2015;15 doi:ARTN 69
10.1186/s12862-015-0339-8.
17. Bachtrog D, Kirkpatrick M, Mank JE, McDaniel SF, Pires JC, Rice W, et al. Are all sex chromosomes created equal? Trends in Genetics. 2011;27 9:350-7. doi:https://doi.org/10.1016/j.tig.2011.05.005.
18. Bachtrog D, Mank JE, Peichel CL, Kirkpatrick M, Otto SP, Ashman TL, et al. Sex determination: why so many ways of doing it? PLoS Biol. 2014;12 7:e1001899. doi:10.1371/journal.pbio.1001899.
19. Rowe L, Chenoweth SF and Agrawal AF. The Genomics of Sexual Conflict. Am Nat. 2018;192 2:274-86. doi:10.1086/698198.
20. Maryańska-Nadachowska A, Kuznetsova VG, Lachowska D and Drosopoulos S. Mediterranean species of the spittlebug genus Philaenus : Modes of chromosome evolution. Journal of Insect Science. 2012;12 1 doi:10.1673/031.012.5401.
21. King R. Chromosomes of three species of mantidae. Journal of Morphology. 2005;52:525 - 33. doi:10.1002/jmor.1050520208.
22. Paliulis LV, Stowe EL, Hashemi L, Pedraza-Aguado N, Striese C, Tulok S, et al. Chromosome number, sex determination, and meiotic chromosome behavior in the praying mantid Hierodula membranacea. PLoS One. 2022;17 8:e0272978. doi:10.1371/journal.pone.0272978.
23. Harrison MC, Jongepier E, Robertson HM, Arning N, Bitard-Feildel T, Chao H, et al. Hemimetabolous genomes reveal molecular basis of termite eusociality. Nat Ecol Evol. 2018;2 3:557-66. doi:10.1038/s41559-017-0459-1.
24. Terrapon N, Li C, Robertson HM, Ji L, Meng X, Booth W, et al. Molecular traces of alternative social organization in a termite genome. Nature Communications. 2014;5 1:3636. doi:10.1038/ncomms4636.
25. Svenson GJ and Whiting MF. Phylogeny of Mantodea based on molecular data: evolution of a charismatic predator. 2004;29 3:359-70. doi:https://doi.org/10.1111/j.0307-6970.2004.00240.x.
26. Guan D, McCarthy SA, Wood J, Howe K, Wang Y and Durbin R. Identifying and removing haplotypic duplication in primary genome assemblies. Bioinformatics. 2020;36 9:2896-8. doi:10.1093/bioinformatics/btaa025 %J Bioinformatics.
27. Cheng H, Concepcion GT, Feng X, Zhang H and Li H. Haplotype-resolved de novo assembly using phased assembly graphs with hifiasm. Nature Methods. 2021;18 2:170-5. doi:10.1038/s41592-020-01056-5.
28. Simão FA, Waterhouse RM, Ioannidis P, Kriventseva EV and Zdobnov EM. BUSCO: assessing genome assembly and annotation completeness with single-copy orthologs. Bioinformatics. 2015;31 19:3210-2. doi:10.1093/bioinformatics/btv351 %J Bioinformatics.
29. Zhou C, McCarthy SA and Durbin R. YaHS: yet another Hi-C scaffolding tool. Bioinformatics. 2023;39 1 doi:10.1093/bioinformatics/btac808.
30. Langmead B and Salzberg SL. Fast gapped-read alignment with Bowtie 2. Nat Methods. 2012;9

4:357-9. doi:10.1038/nmeth.1923.
31. Servant N, Varoquaux N, Lajoie BR, Viara E, Chen CJ, Vert JP, et al. HiC-Pro: an optimized and flexible pipeline for Hi-C data processing. Genome Biol. 2015;16:259. doi:10.1186/s13059-015-0831-x.
32. Wang S, Wang H, Jiang F, Wang A, Liu H, Zhao H, et al. EndHiC: assemble large contigs into chromosome-level scaffolds using the Hi-C links from contig ends. BMC Bioinformatics. 2022;23 1:528. doi:10.1186/s12859-022-05087-x.
33. Flynn JM, Hubley R, Goubert C, Rosen J, Clark AG, Feschotte C, et al. RepeatModeler2 for automated genomic discovery of transposable element families. Proceedings of the National Academy of Sciences. 2020;117 17:9451-7. doi:10.1073/pnas.1921046117.
34. Benson G. Tandem repeats finder: a program to analyze DNA sequences. Nucleic Acids Res. 1999;27 2:573-80. doi:10.1093/nar/27.2.573 %J Nucleic Acids Research.
35. Stanke M, Keller O, Gunduz I, Hayes A, Waack S and Morgenstern B. AUGUSTUS: ab initio prediction of alternative transcripts. Nucleic Acids Research. 2006;34 suppl_2:W435-W9. doi:10.1093/nar/gkl200 %J Nucleic Acids Research.
36. Chen S, Zhou Y, Chen Y and Gu J. fastp: an ultra-fast all-in-one FASTQ preprocessor. Bioinformatics. 2018;34 17:i884-i90. doi:10.1093/bioinformatics/bty560 %J Bioinformatics.
37. Pertea M, Pertea GM, Antonescu CM, Chang TC, Mendell JT and Salzberg SL. StringTie enables improved reconstruction of a transcriptome from RNA-seq reads. Nat Biotechnol. 2015;33 3:290-5. doi:10.1038/nbt.3122.
38. Haas BJ, Salzberg SL, Zhu W, Pertea M, Allen JE, Orvis J, et al. Automated eukaryotic gene structure annotation using EVidenceModeler and the Program to Assemble Spliced Alignments. Genome Biology. 2008;9 1:R7. doi:10.1186/gb-2008-9-1-r7.
39. Slater GS and Birney E. Automated generation of heuristics for biological sequence comparison. BMC Bioinformatics. 2005;6:31. doi:10.1186/1471-2105-6-31.
40. Buchfink B, Reuter K and Drost HG. Sensitive protein alignments at tree-of-life scale using DIAMOND. Nat Methods. 2021;18 4:366-8. doi:10.1038/s41592-021-01101-x.
41. Jones P, Binns D, Chang HY, Fraser M, Li W, McAnulla C, et al. InterProScan 5: genome-scale protein function classification. Bioinformatics. 2014;30 9:1236-40. doi:10.1093/bioinformatics/btu031.
42. Li H and Durbin R. Fast and accurate short read alignment with Burrows-Wheeler transform. Bioinformatics. 2009;25 14:1754-60. doi:10.1093/bioinformatics/btp324.
43. Li H, Handsaker B, Wysoker A, Fennell T, Ruan J, Homer N, et al. The Sequence Alignment/Map format and SAMtools. Bioinformatics. 2009;25 16:2078-9. doi:10.1093/bioinformatics/btp352.
44. Wang Y, Tang H, Debarry JD, Tan X, Li J, Wang X, et al. MCScanX: a toolkit for detection and evolutionary analysis of gene synteny and collinearity. Nucleic Acids Res. 2012;40 7:e49. doi:10.1093/nar/gkr1293.
45. Hao Z, Lv D, Ge Y, Shi J, Weijers D, Yu G, et al. RIdeogram: drawing SVG graphics to visualize and map genome-wide data on the idiograms. PeerJ Comput Sci. 2020;6:e251. doi:10.7717/peerj-cs.251.
46. Emms DM and Kelly S. OrthoFinder: phylogenetic orthology inference for comparative genomics. Genome Biol. 2019;20 1:238. doi:10.1186/s13059-019-1832-y.
47. Kozlov AM, Darriba D, Flouri T, Morel B and Stamatakis A. RAxML-NG: a fast, scalable and

user-friendly tool for maximum likelihood phylogenetic inference. Bioinformatics. 2019;35 21:4453-5. doi:10.1093/bioinformatics/btz305.

Figure 1

Click here to access/download;Figure;figure1.pdf

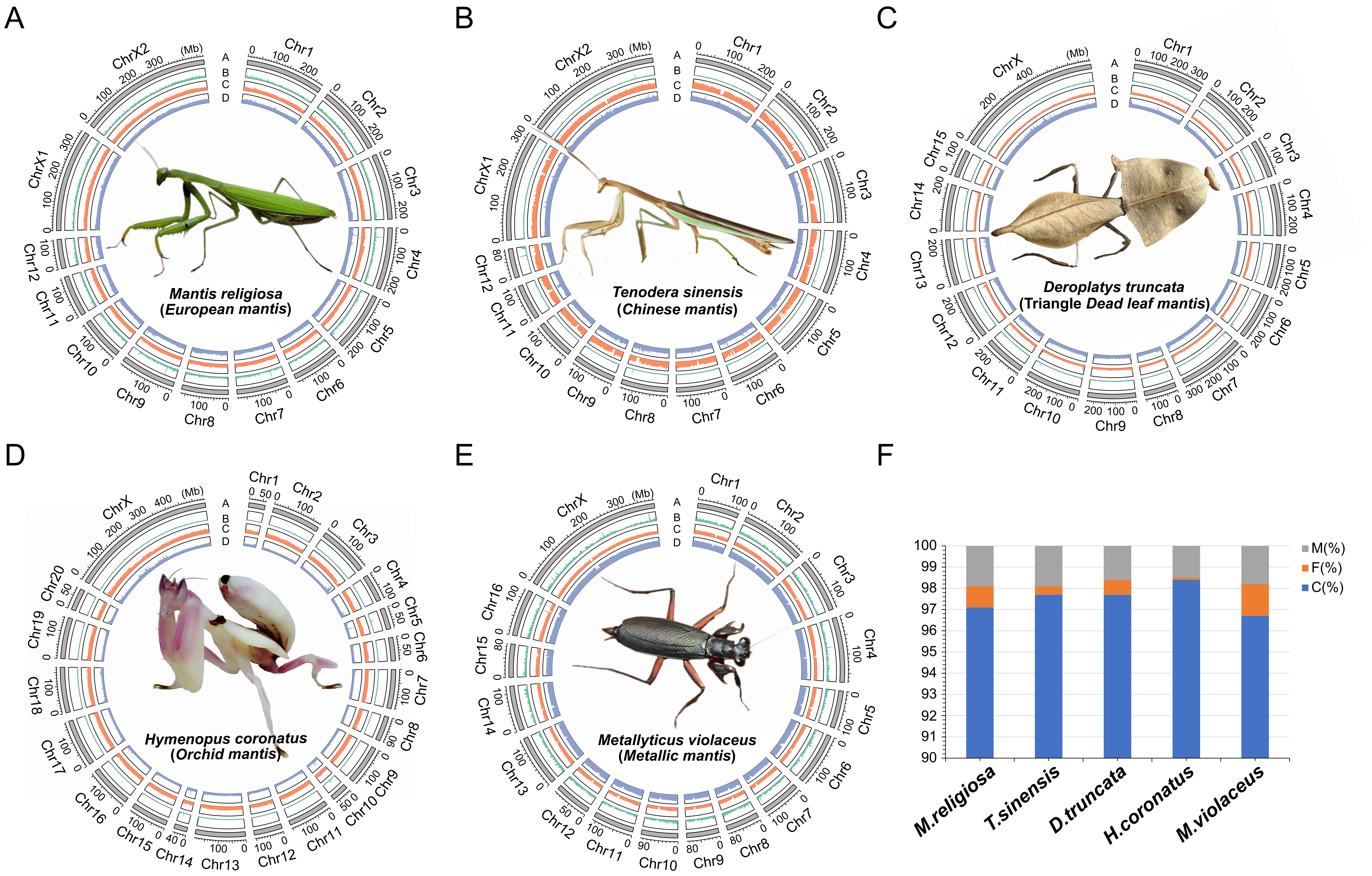

Figure 2

Click here to access/download;Figure;Figure2.pdf

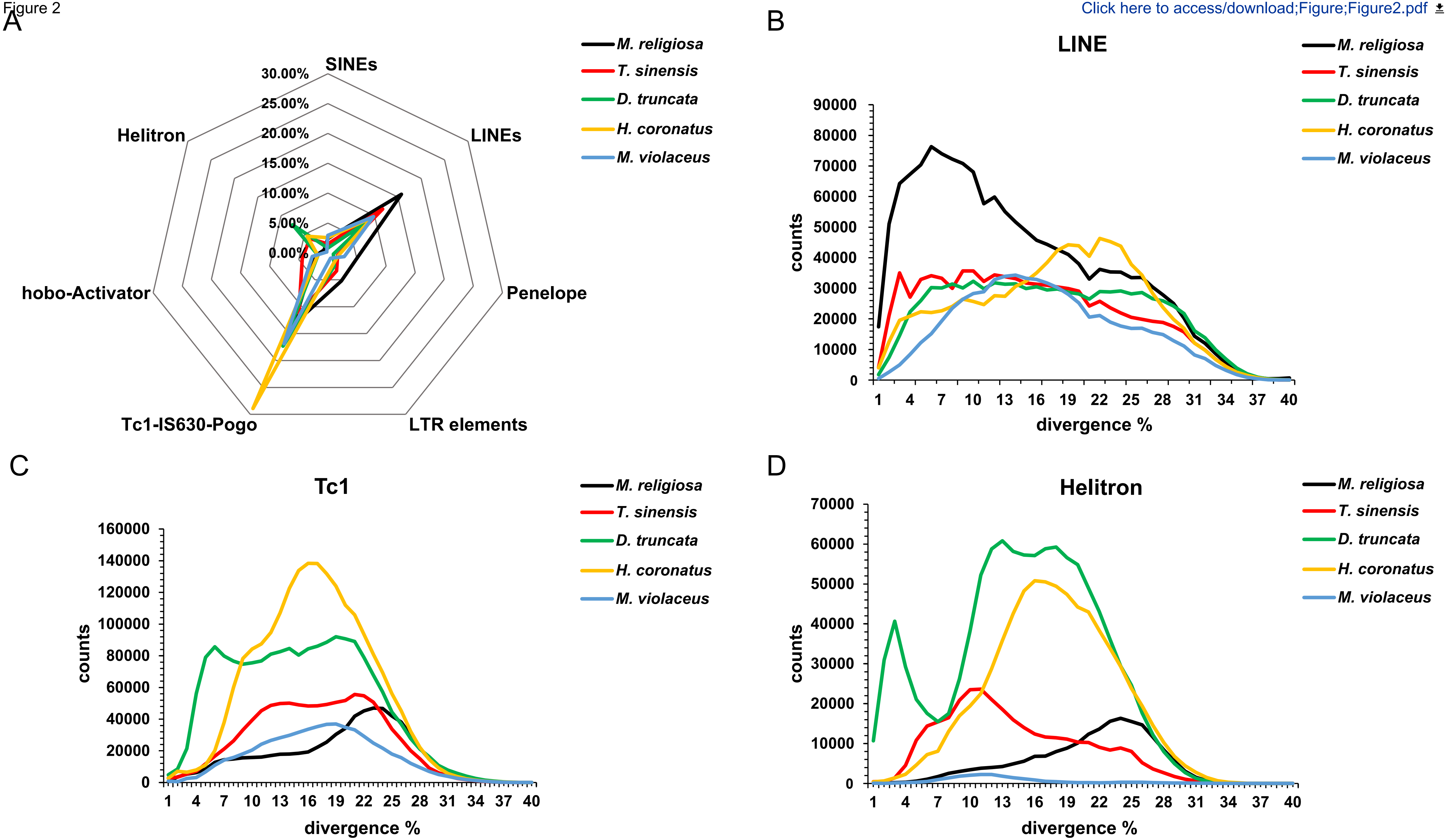

Figure 3 Click here to access/download;Figure;Figure3.pdf

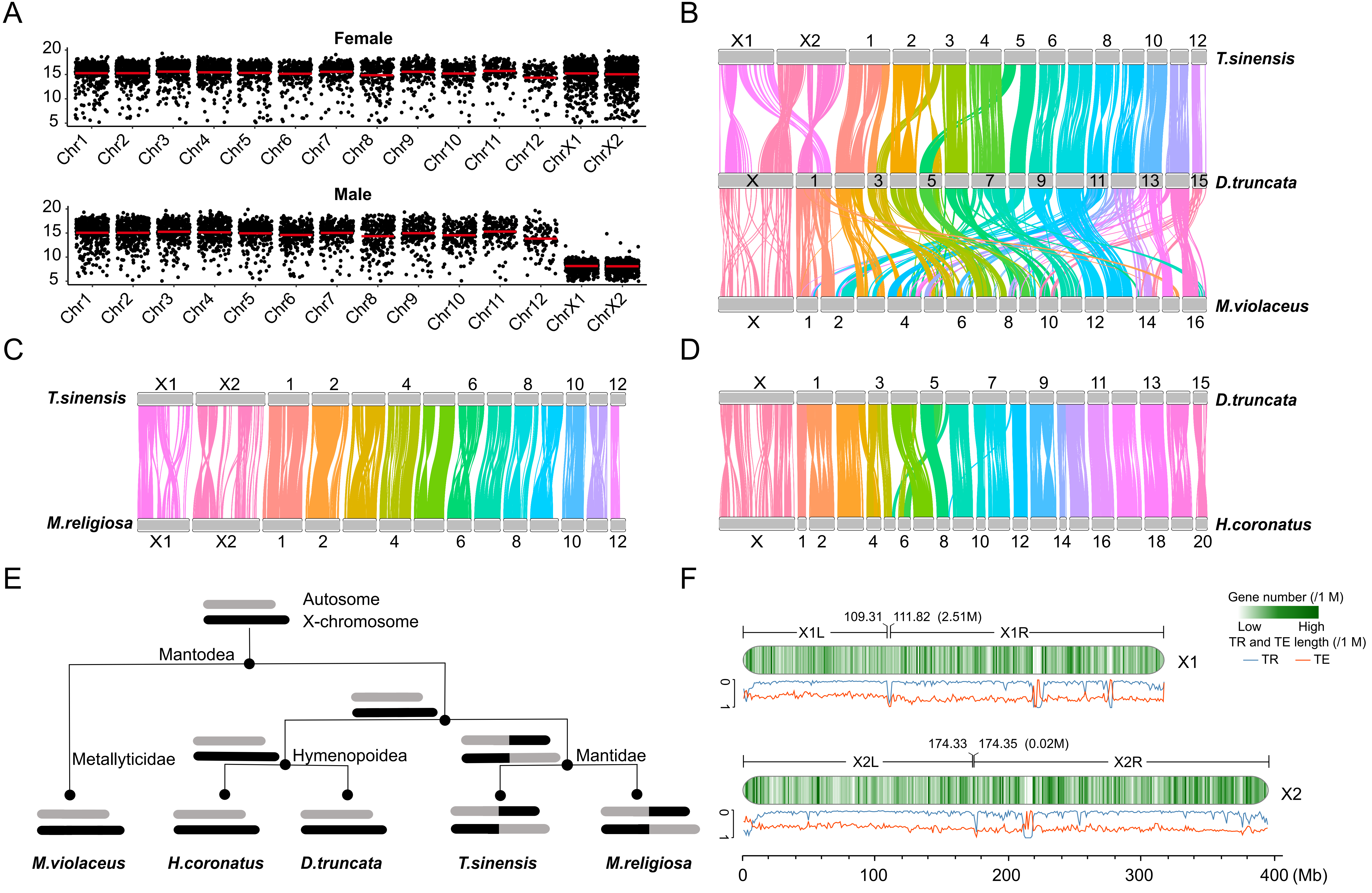

Figure 4

Click here to access/download;Figure;Figure4.pdf

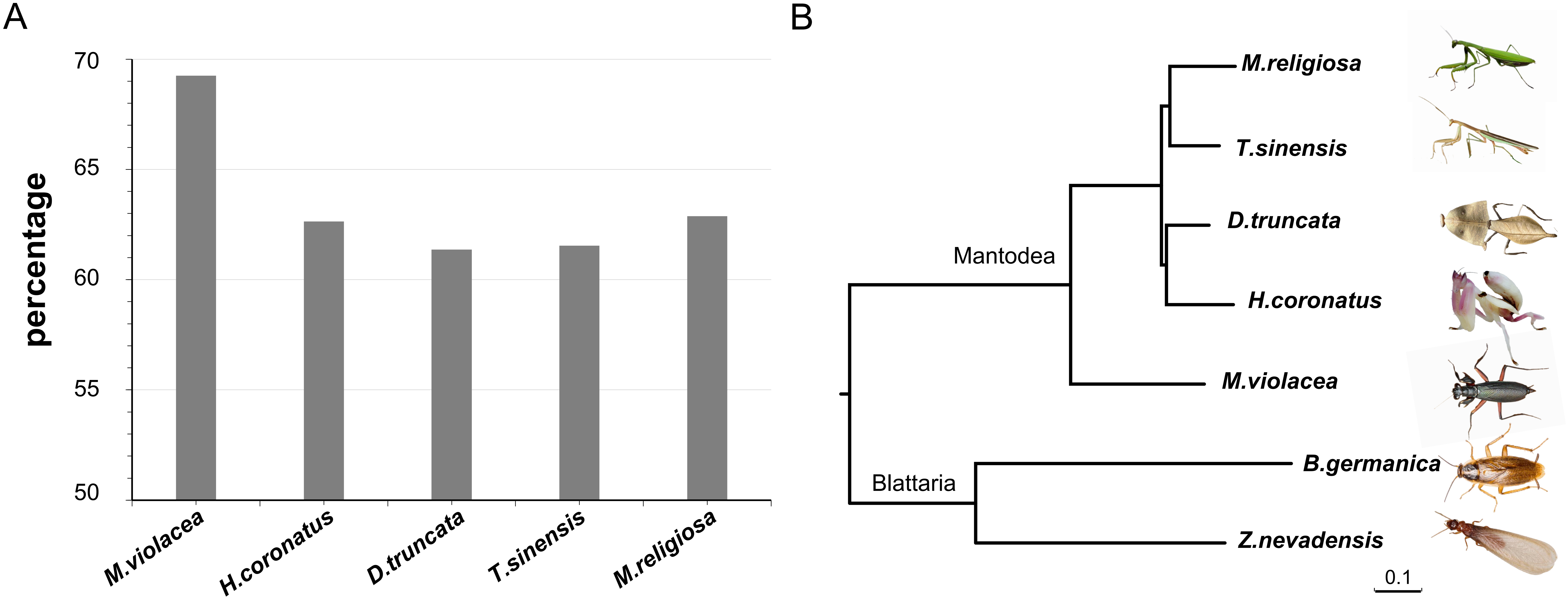

Click here to access/download
Supplementary Material
Supplemental materials.docx

Click here to access/download;Personal Cover;Response to Editor and Reviewers.docx

GIGA-D-23-00361
Mantodea phylogenomics provides new insights into X-chromosome progression, phylogeny clarification, and adaptive evolution
hangwei liu; lihong lei; fan jiang; bo zhang; hengchao wang; yutong zhang; hanbo zhao; anqi wang; Wei Fan; guirong wang
GigaScience

Dear Dr Fan,

Thank you for considering GigaScience. Peer review of your manuscript is now complete and, in the light of the reports, and my own assessment as Editor, I regret to inform you that your manuscript cannot be accepted for publication in GigaScience.

Please find the reviewers' reports at the end of this email. Please also take a moment to check our website at https://www.editorialmanager.com/giga/ for any additional comments that were saved as attachments.

If at some stage you are able to fully address these concerns, you may wish to submit to GigaScience with the revised manuscript. If you are able to do this a full cover letter, explaining the revisions made, should accompany the submission.

Reply: Thanks for the editor's suggestions, and we have tried our best to address the reviewer's concerns.

The most change is removal of two result sections "The expansion of Ninjurin2 may contribute to mimicry in orchid and triangle dead leaf mantis" and "Expansion of olfactory receptor enhances camouflage and hunting ability in orchid mantises". We agree that these inferences were only based on genomic sequences, without any experimental validation. We can't provide more solid evidences in a reasonable time. Therefore, we decided to remove these two sections from our manuscript.

Besides, we tune down our conclusions in two other result sections "Metallic mantis evolves much slower than other mantises" and "Triangle dead leaf mantis should be moved out of superfamily Mantoidea". To be concise, these two sections were merged into one section in the revised manuscript. We thought that these results are still valuable to the scientific field, although our evidences may not be enough strong. We insist to keep them in the revised manuscript, and we agree to make further revisions according to editor's and reviewer's suggestions.

Last, we have made modifications responding to all the other concerns raised by the 3 reviewers. So, we want to resubmit the revised manuscript to GigaScience and hope you will reconsider our work again. The results present in this revised manuscript is much convincing with data supported. We still submit to GigaScience as an Research paper, but if you think it is more suitable for a dataNote paper, we can also accept.

I wish you every success with your research and hope that you will consider us again in the future.

Best wishes,

Hongfang Zhang
GigaScience
www.gigasciencejournal.com

Reviewer reports:

Reviewer #1: This manuscript delved into the chromosome-level genomes of 5 praying mantis species, unraveling the evolution of the mantis X chromosome and shedding light on their phylogenetic relationships. The research also specifically investigated the ecological adaptation of the triangle dead leaf and orchid mantis, focusing on the programmed cell death gene Ninjurin2 and the expansion of olfactory receptor genes. The work as a whole is somewhat innovative and worth considering for publication on GigaScience. However, I think there are still some problems to be solved before publication.

Major comments

1. Figure 3: The authors propose an interesting model of translocation between X-chromosome and autosome in Mantoidea lineage through chromosome-level rearrangement (Figure 3E). However, this model apparently lacks data analysis to support their assumption. Since they have Hi-C data, they should be able to find significant difference at the level of three-dimensional genome, such as compartment, TAD, and loop. Without such a detailed story, the genomic analysis is not able to catch the eyeballs of the audience.

Reply: Thanks for the reviewer's suggestions. Our Hi-C data was generated specially for contig scaffolding, it may not be suitable for three-dimensional analyses. The major skills of our group are sequence level analyses, and it is better for other groups who expertise in three-dimensional analyses and have interest to perform this job.

2. Figure 5: The authors note heightened expression of expanded Ninjurin2 genes in the abdomen. They posit that this expansion in the genomes of triangle dead leaf and orchid mantises enhances the precision of tissue or organ development, contributing to impeccable mimicry. However, this conclusion appears inconsistent, particularly as orchid mantises lack extended edges on their abdomens like the triangle dead leaf. Notably, Ninjurin2 genes exhibit a slight upregulation in the hind and forelegs of orchid mantises, suggesting a need for further examination of their expression levels in petal-shaped femoral lobes. Additionally, where is Fig. 5D and Fig. 5E (Line 304-314). I do not understand how the authors align the

three domains in Figure 5B., which looks strange.

Reply: The result section related with Figure 5 was removed.

3. Figure 6: The author posits that the expansion of olfactory receptor genes in the orchid mantis contributes to its ability to locate flowers for camouflage and predation. However, a critical assumption in this study is that the orchid mantises frequently stop on flowers. This claim lacking support, and recent research contradicts this assumption, reporting that orchid mantises rarely inhabit flowers (Zhao et al., 2023, Evol Ecol. https://doi.org/10.1007/s10682-023-10230-y). Consequently, this aspect of the study appears to be unfounded. Adding to the perplexity, the authors employed different flower odor molecules for molecular docking with two highly expressed OR proteins in orchid mantises. Surprisingly, their findings suggested that orchid volatiles exhibit a higher binding capacity with other plant volatiles. These results are incredible, and the authors neglected to conduct a comprehensive literature research on the name of the "orchid mantis", revealing no inherent connection to actual orchids. The experimental results, relying heavily on AlphaFold2 predictions, raise significant doubts about their validity.

Reply: The result section related with Figure 6 was removed.

Minor comments

Line 21: Change "import" to "important", also for Line 48.
Reply: Corrected.

Line 48: Change "public" to "publicly".
Reply: “public” was deleted.

Line 211-215: Figure S10 should be cited here. If feasible, there's a recommendation to incorporate Figure S10 into the main Figures section due to perceived inadequacy in the results of Figure 3 to substantiate the sex determination systems of these mantises. Especially for D. truncate.

Reply: We cited Figure S10 in the position as the reviewer suggested. However, we don’t want to incorporate Figure S10 into the main Figures, because that the findings in Figure S10 are public known and not our results. The X1X2Y sex determination has long been known in Mantidae, and our results confirmed it on the genome-sequence-level.

Line 244: Change "thorax" to "prothorax".
Reply: Corrected.

Line 289: This ecological strategy of triangle dead leaf and orchid mantises is recommended

as camouflage.
Reply: This section was removed.

Line 297: Change "2" to "two".
Reply: This section was removed.

Line 321: As with the questions raised above, the expansion of OR is not used to locate flowers, nor to help them camouflage. However, I think there is a possibility that the orchid mantis can release some of the Oriental Honeybee's hormones to attract them and promote predation (Mizuno et al., 2014, Zoolog Sci. https://doi.org/10.2108/zs140126). Do these ORs play a role in recognizing prey and promoting the release of the Oriental Honeybee's hormones? You can conduct some tentative analyses, such as homology comparison and functional analysis, with orchid mantis potential prey ORs.

Reply: This section was removed.

ine 395: Doesn't match the results.
Reply: This section was removed.

Line 433: Checking the format.
Reply: Corrected.

Reviewer #2: Review of "Mantodea phylogenomics provides new insights into X-chromosome progression, phylogeny clarification, and adaptive evolution" by Liu et al.

This is a manuscript that describes chromosome-level assemblies of three mantis genomes, and a comparative genomic analysis of all available mantis genomes (6). It represents an important contribution to science since there is a general lack of high-quality polyneopteran genomes available at this moment. Mantids are charismatic insects, and the genomic resources that the authors have generated will be useful to the community. The manuscript includes several standard analyses of genomic data, some of which should have been done a bit more rigorously. The inferences made from the data are speculative and overreaching. Below I give specific comments on several points. There are several technical issues, but the most important issues are the writing. Many of the arguments are without evidence, and this work cannot be accepted in its current format.

Reply: Thanks for all the reviewer's suggestions. We have removed two result sections (Ninjurin2 and olfactory receptor) and tone down our conclusions on other two result sections (Metallic mantis evolves slower and phylogenic classification of Triangle dead leaf mantis). We

also significantly improved the writing.

Title: I am not sure whether the data really provides new insights into all the things mentioned in the title. I suggest you tone down the title.

Reply: We have tone down the title into “The genomes of five mantises provide insights into sex determination mechanism and evolution of Mantodea”.

Introduction: Authors introduce mantises in a very general manner. What is the main hypothesis of the study? The introduction should be written to build up to highlight how the data can test the hypothesis. The first to the fourth paragraphs are informative only in terms of introducing mantids to someone who does not know the group. The potential readers of this manuscript should already by well aware of the general aspects of mantis biology. The introduction should end with specific hypotheses, which are not presented.

Reply: We agree with the reviewer, indeed, the introduction is written in a very general manner. We have tried to revise it to be more professional. The first paragraph talks about the importance of mantis, in agriculture, medicine, and bionics, that is the major reason we are going to sequence their genomes. The second paragraph summarizes the previous phylogeny and evolution researches in Mantodea, which is highly related with our major analyses. The third paragraph reviews all the genomic studies in Mantodea, and brings to this research project.

Results (from L103): The way the results for six mantid species is presented is verbose and can be shown easily as tables or figures. It's not clear to me how many flow cells of PacBio Sequel II and Illumina NovaSeq are used to generate this data. Because these insects have relatively large genomes, it is important to increase the sequencing depth. The fact that N50 values are relatively low (such as 1Mb, 13Mb) means that the sequencing depth was not sufficient. Since HiC sequencing was done, please provide contact maps using Juicebox.

Reply: We have improved the presentation of the assembly results for five mantises, and these numbers are also available in Table 1. The revised sentences are much easier for reading: We generated 179 Gb (48X) (M. religiosa), 97 Gb (36X) (T. sinensis), 112 Gb (26X) (D. truncata), 177 Gb (56X) (H. coronatus), and 147 Gb (63X) (M. violaceus) PacBio HiFi data, and 127 Gb (35X) (M. religiosa), 112 Gb (42X) (T. sinensis), 153 Gb (35X) (D. truncata), 182 Gb (58X) (H. coronatus), and 183 Gb (79X) (M. violaceus) Illumina Hi-C data.

The sequencing task was performed by a commercial sequencing company, which only provides the total base number but not flow cell number. The PacBio Hifi data was used to assembly the contigs, and HiFi depth range from 26-63 X. The two mantis species with relatively low contig N50 values, 1 Mb (M. religiosa), 13 Mb (T. sinensis), have sequencing

depth 48X (M. religiosa) and 36X (T. sinensis), which are not the lowest among all the 5 mantis species. The real reason is that the sequencing samples of M. religiosa and T. sinensis have much higher heterozygosity than the other 3 mantis species (Figure S3), which largely influence contig N50 sizes. The contact maps for HiC data was shown in Figure S1, which was drawn by EndHiC instead of Juicebox.

Sampling (from L410): Since some of the specimens were collected from other countries, such as Cameroon (where collecting and export permits are extremely difficult to obtain), and Malaysia, please provide proper collecting and export permit documentation. The project must follow the Nagoya Protocol (https://www.cbd.int/abs/). Please provide additional information about transcriptome sequencing in terms of how the data were generated.

Reply: None of the samples were collected from foreign countries, but all in our homeland China. Thus, export permits were not needed. Details can be found in the method section: Mantis adults were collected at different locations: M. religiosa and T. sinensis from the forest of Guangzhou, China; H. coronatus from the rainforest of Xishuangbanna, China; and D. truncata and M. violaceus from two captive breeding centers in Beijing, China.

The details for transcriptome sequencing can be found in the method section: Total RNA from the head, eye, thorax, abdomen, forefoot, midfoot and midfoot of female adults was extracted with TRIzol reagent (Invitrogen) and used to construct cDNA libraries. Transcriptome sequencing data were generated via the Illumina NovaSeq 6000 system in PE150 mode.

Synteny analysis (from L485): The way that collinearity analysis was done was based on mapping protein coding genes to the chromosomal map. Please indicate how many protein coding genes were used. Also, it appears that T. sinensis genome was used as a reference for the synteny analysis, and this needs to be justified. Phylogenetically it may make sense to use an ancestral lineage as a reference.

Reply: In our collinearity analysis, all the protein coding genes of the two compared species were aligned, and only the reciprocal best pairs were used as inputs for MCScanX. We did not use T. sinensis genome as a reference, but perform the synteny analysis in pairwise.

Phylogenetic analysis (from L507): The authors built a phylogenomic tree based on 7 dictyopterans. The information about the data matrix (completeness, missing data, etc) is missing. Running such a large dataset in a proper way would require a supercomputer, and that information is missing. It is not clear why the NJ analysis was even performed. That is not considered a phylogenetic analysis. Divergence time analysis was performed using MEGA which is not what typical phylogeneticists would use. Please perform a rigorous analysis using proper calibration points, and provide the source of information.

Reply: Phylogenetic tree was constructed based on 4,014 single-copy OGs. We run all the analyses in a supercomputer. NJ was the standard method for phylogeny tree construction with genome-wide data. We also run ML method, and three phylogeny trees of both methods consist well. In the revised manuscript, we have removed the part for divergence time analyses.

TE expansion (from L150): The TE analysis descriptive at best. The inference that "the component and divergence times of various TE types are significantly distinct among different Mantodea lineages, which may be responsible for the adaption to various environments for different mantis" is totally unjustified and speculative. The authors have not demonstrated the functional role of TE and there is no connection between the TE expansion and adaptation to various environment. Please refrain from making unjustified comments.

Reply: We have removed this comment as the reviewer suggested.

Metallic mantis (from L233): I am not sure the statement "Metallic mantis evolves much slower than other mantises" is correct. Maybe the substitution rates are slower, but that does not mean that it's a relic species or that did not evolve fully. What are ancestral traits of Mantodea? Morphological similarities to cockroaches? Well, the authors did not mention a single morphological trait that was retained in this lineage. This is an incorrect phylogenetic interpretation about morphological evolution.
Reply: The ancestral traits of Mantodea are more like cockroaches, and Metallic mantis is also more similar to cockroaches than to the other mantises. Because Metallic mantis is morphologically more similar to the ancestor of Mantodea, Metallic mantis shares more orthologous groups to cockroaches than the other mantises, and Metallic mantis has much lower substitution rate compared to other mantises, these evidences collectively suggest that Metallic mantis evolves much slower than other mantises.

Phylogenetic inference (from L256): This work is based on 6 mantids. A phylogenetic inference based a small taxon sampling cannot be a sole source of classification changes. The authors did not even perform a proper topology testing (such as SH test) and did not attempt to figure out where the source of phylogenetic signal was. This entire section is not justified.

Reply: We agree with the reviewer that we have used a small taxon sampling, and we have tone down on conclusion. We will not claim to change the species classification based on our results, but only claim that our results provide a resource for phylogenetic inference.

Ninjurin2 (from L289): The finding that Ninjurin2 expansion is found in two mantis species is novel, but the study lacks empirical evidence that this expansion indeed has anything to do with mimicry. The statement, "The expansion of Ninjurin2 genes in the genomes of the triangle dead leaf and orchid mantises would enhance the capacity for precise manipulation

of tissue or organ development, and consequently contribute to the formation of impeccable mimicry," is speculative and unjustified. The Ninjurin2 may have a totally different function and has nothing to do with mimicry. Also one needs to be very clear why mimicry actually means.

Reply: This section was removed.

OR genes (L352): "We speculate that the expansion of OR genes in the orchid mantis may enhance its ability to locate flowers, thereby maximizing its effectiveness in camouflage." OK. This statement has a little bit of evidence based on bioinformatic analysis of protein (Alphafold) and molecular docking. But the ability to smell flowers may have nothing to do with the effectiveness in camouflage. Please refrain from unjustified speculations.

Reply: This section was removed.

Reviewer #3: This paper is a worthwhile expansion of available mantid genomes and refinement of several existing species for which genomes have been produced. However it's conclusions go far, far beyond what can be supported by the data presented and the experiments performed. A radically cut-down version of this manuscript may be publishable if focused almost exclusively on the genomic aspects, but the phylogenetic and gene-function conclusions are not scientifically supported.

Reply: Thanks for the reviewer's kind suggestions. We have made a cut-down version of the manuscript by removing two result sections (Ninjurin2 and olfactory receptor). Besides, we tone down our conclusions on other two result sections (Metallic mantis evolves slower and phylogenic classification of Triangle dead leaf mantis), which were further merged into one section for concise.

Assembly and X chromosome evolution

These sections are the strongest but could still do with improvement. The use of common names for the species throughout the manuscript causes unnecessary bloat (triangle deaf leaf mantis vs D. truncata) and is why we use scientific names in the first place. While this might be OK if the paper concerned just 1 species, with 5 to cover it seriously impeded the clarity of writing. After their first mention in the abstract and intro, use the scientific names. In a related point the format for reporting many statistics that was used " A%, B%, C%, D%, E% something about the statistic and then the species names 'respectively' " (most heavily in lines 103-114) is almost unreadable. Statistics of this type are best reported in tables, and if they really must be in the text use either a range e.g. A% in D. truncata to B% in M. religiosa, or if you really must list all of them put the species name after each statistic e.g. A% (D. truncata), B% (M. religiosa) etc.

Reply: We have revised these sentences according to the reviewer's suggestions, they are

much simpler and more readable now.

Other points:

1. Claims about assembly quality (lines 121-122) should be qualified, simply citing previously published genomes in this group is insufficient.

Reply: We have deleted this sentence "The assembly quality of all our five mantises is much higher than those of previously published cockroaches, termites, and mantises" in the revised manuscript. This time, we no longer compare ours to that of cockroaches, termites, but only compare our mantis assembly to the published mantis assemblies (available in the next paragraph).

2. The XXY system is restricted to Mantidae based on the evidence presented in this and previous papers. Superfamily classification of mantids has been changed several times in recent years, with Mantoidea currently composed of Mantidae, Deroplatyidae and Dactylopterygidae. Some phylogenetic studies support this, in particular Svenson & Whiting 2008 (your citation [9]) and Liu et al. 2023 based on mt genomes. Others do not, including the phylogeny generated here and other mt genome based analyses (Ma et al. 2023, Lin et al. 2023). But all agree on the monophyly of Mantidae, so the more conservative approach is the map this trait to the family Mantidae, rather than your version of Mantoidea for which there is considerable dispute (see below).

Reply: We agree with the reviewer, and have changed all "Mantoidea" into "Mantidae" in the maintext as well as figures.

Phylogeny

The largest issue with the phylogeny section is that the taxon sampling is really just insufficient for the conclusions made. There are 12 superfamilies recognised with Mantodea which aren't included in the current analysis. What is included is 2 or 3 (depending on taxonomy) of the most derived mantid lineages (Mantidae, Hymenopodidae and Deroplatyidae), and one of the earlier diverging groups (Metallyctidae), but not the most divergent lineage Chaeteessoidea which is the sister to the rest in both molecular (Svenson & Whiting, 2008) and morphological analyses, and is as yet unsequenced for a genome. There is roughly 80 million years and 9 superfamilies worth of evolution between the first and second nodes in your tree (Metallyticus vs rest). All of this is very important and why we don't make excessive conclusions based on taxonomically unrepresentative samples. For example the conclusions regarding substitution rate in Metallyticus (line 250-254) are conflated by this huge gap. Node to leaf distances for each of the other 4 species are shorter than that for Metallyticus i.e. most of the observed divergence in these species is in the common ancestor of these four. Similarly, the sampling within Blattodea is grossly insufficient to make conclusions regarding ancestral states within that group, and thus to infer how much or how little mantids have diverged from that common ancestor. This makes the time-tree analysis misleading as the key divergence

nodes needed to reconstruct it are missing. While there is utility in including a phylogenomic analysis to provide context to some of your other analyses (such as synteny, gene family evolution etc.), it cannot be regarded as decisive from a classification standpoint and making conclusions about taxonomic changes etc is inappropriate. Most of these sections should be removed.

Reply: We agree with most of the reviewer's suggestions, and largely tone down our conclusion based on the phylogeny analyses. In the phylogeny tree, where branch length responsible to substitution rate, the total length from Mantodea to M.violacea is obviously shorter than the total length from Mnatodea to M.religiosa (or ***T.sinensis, D.truncata and H.coronatus***). Although the branch from Mantodea to M.violacea spans quite a long time, and currently can't know which period evolves fast and which period evolves slow, we just take an average that this branch may substitute evenly during the whole time. Only with this evidence, we can't conclude that M.violacea evolves slowly. But together with other evidences (M.violacea shares more othologous groups to cockroaches, and M.violacea is morphologically more similar to cockroaches), we make a preliminary but not solid inference that the lineage of M.violacea may evolves slower than the lineages of other mantises.

Besides, we also give up our claim on taxonomic changes. We just provide some genome-wide evidences for resolving the controversy in Mantodea phylogeny, and hope that our results will give some help to future taxonomy classification jobs performed by more professional groups.

Other points:

1. Concatenation of a supermatrix of all single copy orthologues is not methodologically rigorous and would not be an acceptable approach in any specialised phylogeny/evolution journal. There are many recent excellent examples of phylogenomic analyses of insects which could be drawn on for better methods and should be applied here.

Reply: Concatenation of a supermatrix of all single copy orthologues, has been widely applied in many genome projects for a long history. This method captures as more genomic data as possible, which has its unique advantage compared to other phylogenic construction methods. We will not give up this method.

2. The time tree approach is invalid as described. The calibration for divergence between Mantis and Blattella is much smaller than the inferred divergence between Mantodea and Blattodea (212-225 vs 320). This suggests a major error in the analysis, probably stemming from the fact that Mantis-Blattella is not a node in your tree - calibrations need to be made to specific nodes not vague regions of the tree (there are 4 nodes between those genera in your tree, which node was so calibrated?). Additionally the cited references are for methods not for the fossils etc. upon which the time calibration is used. Timetree is a data aggregator,

not a primary source for fossil dates. The 12 papers which TimeTree uses to calculate that range of 212-225 are listed in the websites output and one of those should be used to find a fossil which can actually be placed in your phylogeny based on the sampled taxa. Timetree is nice for undergrad teaching or framing an analysis, but it isn't a valid source for a calibration date as you use here.

Reply: We have removed this part in the revised manuscript.

Gene Family analysis
Both the analysis of Ninjurin2 and ORs is hugely short of the level needed to draw the conclusions which you draw.

Reply: These two sections (Ninjurin2 and ORs) were removed in the revised manuscript.

You find that Ninjurin2 family is expanded in Deroplatys+Hymenopus. OK but what other gene families are expanded in each lineage? Is two extra copies of this gene (max 4 vs mas 2 in other lineages) significant compared to other gene families in Mantodea? How do you know that it is significant? No data is presented to support this, just a deep-examination of one gene family. Is it associated with gross body morphology? Neither of the cited papers suggest so - one discusses its function in neurons and the other in immune cells, and both are in vertebrate systems. I see no initial evidence to focus on this gene in the presented materials - it isn't shown to be the ONLY gene which is expanded in the two species with mimetic morphology, and there is no evidence from other systems for this gene having a role in gross morphology regulation. The next step was to do expression analysis - this was both badly designed and the methods used inappropriate. The methods as written state that RNA was taken from various body tissues of females - so presumably an adult as mantids cannot be reliably sex-determined as junveniles. The growth of the body regions involved in mimicry (the various flanges, expansions etc.) happen during the molt cycle which is finished once you are sampling an adult. Any differential expression in an adult has nothing to do with body morphology as that has already formed. If Ninjurin2 were affecting morphology it would be doing so via expression in the juvenile stages esp. in the lead up to molts between the second-to-last and last nymphal instars where the most extreme morphological changes occur. The RNA sampling as conducted will not test the hypothesis you propose for Ninjurin2. The method of expression analysis is also flawed as no gene is listed for normalisation between samples and species is provided (e.g. a ubiquitous gene unlikely to respond to the effect being tested), thus the differences observed could be simply a factor of different success in RNAseq between samples. There is also no replication of RNAseq data collection at the tissue or species level reported (3 replicates per RNAseq test is considered minimal). Both these issues - lack of normalisation and lack of replication would render this section unpublishable in most journals dealing with expression analysis.

Reply: This section was removed in the revised manuscript.

The analysis of ORs similarly is poorly conceived and no context is provided. OR gene families are notoriously variable in numbers between even relatively closely related species and the rough doubling of ORs between Hymenopus and the other mantid species is not unusual. Is this particularly high or low compared to other insects - not presented. As shown in Fig 6B and stated in the text (lines 333-334) most of the extra copies in Hymenopus are recent duplicates of one of two OR homologues, and divergences within the larger of these clusters are very modest - smaller than the interspecies divergences for most of the other OR gene family members in Fig 6B. This lack of divergence strongly suggests that they aren't functionally distinct from each other, or from the homologues from this cluster found in other mantids - and yet this lack of divergence is proposed to allow Hymenopus to detect scents which the other mantids cannot? This is not shown in any rigorous way - for instance the Alphafold2 predictions for two Hymenopus homologues suggest ligand binding for certain volatiles, but there is no analysis of the homologues of these ORs from other mantids. Given the lack of divergence between the ORs in this cluster I'd not be surprised if the ligands fitted to all mantid ORs from this cluster. It isn't enough to demonstrate that Hymenopus can smell these volatiles, to demonstrate adaptive expansion of this gene family (which is what you conclude happened) you need to demonstrate that other mantids can't detect these volatiles. Which is not provided in this submission. Additionally, the conceptual basis of this conclusion is flawed - Hymenopodids as a group utlise a wide range of flowers as hunting grounds, they aren't specialised on orchids in any way. The term orchid mantid refers to their shape, not a host association - any flower will do such that their overall outline is concealed from predators. Finally, the refences listed from the volatiles chosen for ligand analysis is for the method, not for the specificity of the ligands to orchids as stated in the text (line 542). Quick searches show that several of these volatiles have quite wide use across plant groups, so this distinction between orchid specific and plant generalist volatiles needs further justification. Finally it is not listed which of the two ORs for which folding analysis was performed (Hcor.g30282 or Hcor.g30290) was used for ligand analysis. As stated above this ligand prediction needs to be performed on homologues from all mantid species if it is to have any import for the hypothesis proposed that Hymenopus has a unique behavioural response (hanging out on orchid flowers) driven by this specific OR expansion.

Reply: This section was removed in the revised manuscript.

Overall this paper has value but it includes far too many speculative stories underpinned by poorly conducted tests to be publishable in anything like its current form.

Additional comments and suggestions for improved english expression are provided in a marked up copy of the manuscript attached to this review.

Reply: Thanks for the careful comments for English expression, we have made modifications according to each comment raised by the reviewer.

--

Please also take a moment to check our website at for any additional comments that were saved as attachments. Please note that as GigaScience has a policy of open peer review, you will be able to see the names of the reviewers.

In compliance with data protection regulations, you may request that we remove your personal registration details at any time. (Use the following URL: https://www.editorialmanager.com/giga/login.asp?a=r). Please contact the publication office if you have any questions.
