# Supplementary material for: The genomes of 5 mantises provide insights into sex chromosome evolution and Mantodea phylogeny clarification
Source: Gigascience. 2025 Dec 18;15:giaf158. doi: 10.1093/gigascience/giaf158 (PMC12908712; doi:10.1093/gigascience/giaf158)
Supplement: giaf158_GIGA-D-25-00308_Revision_1 [file giaf158_giga-d-25-00308_revision_1.pdf]

GigaScience

The genomes of five mantises provide insights into evolution of sex chromosome and Mantodea lineages

--Manuscript Draft--

**Manuscript Number:**	GIGA-D-25-00308R1
**Full Title:**	The genomes of five mantises provide insights into evolution of sex chromosome and Mantodea lineages
**Article Type:**	Research
**Funding Information:**	Shenzhen Science and Technology Program (KQTD20180411143628272) — Professor Guirong Wang<br>Fund of Key Laboratory of Shenzhen (ZDSYS20141118170111640) — Dr Wei Fan
**Abstract:**	Background Praying mantises, members of the order Mantodea, play important roles in agriculture, medicine, bionics, and entertainment. However, the scarcity of genomic resources has hindered extensive studies on mantis evolution and behaviour.<br>Results Here, we present the chromosome-scale reference genomes of five mantis species: the European mantis (Mantis religiosa), Chinese mantis (Tenodera sinensis), triangle dead leaf mantis (Deroplatys truncata), orchid mantis (Hymenopus coronatus), and metallic mantis (Metallyticus violacea). The assembled genome sizes range ~2.3-4.2 Gb, with contig N50 size 1-109 Mb and 85-99% of sequence anchored to chromosomes. The annotated protein-coding gene number ranges 17,804-19,017, with BUSCO complete rate 96.7-98.4%. We found that transposable element expansion is the major force governing genome size in Mantodea, and suggest that translocations between the X chromosome and an autosome have occurred in the lineage of the family Mantidae. In addition, we found a much lower substitution rate for the lineage of M. violacea than the lineages of other mantises. Furthermore, our genome-wide analyses showed that D. truncata is sister to H. coronatus than M. religiosa and T. sinensis, helps resolve the phylogenic controversies of Deroplatys genus.<br>Conclusions The high-quality genome assemblies of the five mantises provide a valuable resource for evolution studies of Mantodea and genetic improvement and breeding of beneficial biological control agents.
**Corresponding Author:**	Wei Fan<br>Chinese Academy of Agricultural Sciences<br>shenzhen, guangdong CHINA
**Corresponding Author Secondary Information:**	
**Corresponding Author's Institution:**	Chinese Academy of Agricultural Sciences
**Corresponding Author's Secondary Institution:**	
**First Author:**	Wei Fan
**First Author Secondary Information:**	
**Order of Authors:**	Wei Fan
	Guirong Wang
	Hangwei Liu
	Fan Jiang
	Hengchao Wang
	Bo Zhang
	Yutong Zhang
	Hanbo Zhao

Order of Authors Secondary Information:	
Response to Reviewers:	Reviewer reports:<br><br>Reviewer #1: This manuscript presents chromosome-scale genomes for five mantis species, comparative repeat analyses, inferences about sex-chromosome evolution (X1/X2 vs. single X), rate heterogeneity (slow lineage in Metallyticus), and phylogenetic placement of Deroplatys. The datasets are potentially valuable; however, taxonomy/terminology, framing, and several core claims (especially on sex chromosomes) require substantial revision and additional detail.<br><br>Major comments<br>1) Taxonomic terminology and hierarchy (must fix throughout)<br>The usage of order/superfamily/family is repeatedly incorrect or inconsistent. Mantidae is a family, not a superfamily. Please audit the entire manuscript (title/abstract/main text/figures/legends/keywords) and ensure consistent, current placement within order Mantodea and the Dictyoptera clade. Add a short taxonomic note or cite an up-to-date checklist.<br><br>Reply: For Mantidae, we have changed “superfamily” into “family” anywhere in the maintext. To clarify the taxonomic hierarchy, we also added a sentence “Currently, Mantodea (order) and Blattodea (order) are placed within Dictyoptera (superorder). Mantidae is a representative mantis family, which belongs to Mantoidea (superfamily) in Mantodea.” in the maintext.<br><br><br>2) Abstract<br>Add key numbers to convey scale/quality (e.g., genome sizes ~2.3-4.3 Gb; contig/scaffold N50; % of sequence anchored to chromosomes; BUSCO range).<br><br>Reply: According to the reviewer’s suggestion. we have added a sentence “The assembled genome sizes range ~2.3-4.2 Gb, with contig N50 size 1-109 Mb and 85-99% of sequence anchored to chromosomes. The annotated protein-coding gene number ranges 17,804-19,017, with BUSCO complete rate 96.7-98.4%.” in Abstract.<br><br>Replace unclear wording:<br>"genetic breeding of efficient enemy insect" → "genetic improvement and breeding of beneficial biological control agents."<br><br>Reply: Corrected.<br><br><br>Phylogeny statement is vague. State the concrete relationship (e.g., Deroplatys truncata sister to Hymenopus coronatus) and soften to "helps resolve" rather than "providing important evidences for resolving."<br><br>Reply: We have made modifications based on the reviewer’s suggestions.<br><br><br>3) Introduction<br>The Introduction does not prepare readers for the sex-chromosome focus and underspecifies outstanding phylogenetic issues. Please:<br>(i) explicitly flag unresolved nodes your genomes will test (e.g., placement of Deroplatys; early branching of Metallyticus);<br>Reply: After “The metallic mantis (Metallyticus violacea), in the early diverging mantid lineage, has many morphological features similar to those of modern cockroaches,” we added “but the underlying mechanisms have not been discovered”.<br>“Morphological and molecular phylogenies are often inconsistent with previous classification schemes for some sublineages such as Deroplatys genus, because of the rapid radiation events and convergent evolution of ecomorphological strategies” has been revised into “For example, Deroplatys genus has been placed into Mantidae family traditionally, but these species are more similar to species in Hymenopodidae family in many aspects such as morphology and camouflage”.<br><br>(ii) briefly introduce known mantis karyotype diversity and why X1X2 vs. single X

Powered by Editorial Manager® and ProduXion Manager® from Aries Systems Corporation

matters;
Reply: We added a sentence in the end of paragraph one in Introduction: "Although most manties have the common XY sex determination system, some mantises such as M. religiosa and T. sinensis have the X1X2Y type [5-7], making them a special material for studying the evolution of sex chromosomes.".

(iii) trim peripheral material and end with a clear statement of aims/hypotheses.
Reply: Thanks for the reviewer's kind suggestions, but we don't want to trim the peripheral material as the reviewer thought. We have to make some explanations: (1) As we are a research group from agricultural institute, our original aim to sequence these genomes is taking them as the enemy insect, and providing genomic resource for the future genetic breeding jobs. (2) With the genomic sequences, we have some preliminary findings on TE content, X1X2Y chromosomes, slow evolution rate of M. violacea, and new placement for Deroplatys, but these are not our original aims/hypotheses. That's why we wrote the introduction in the current style.

4) Methods
Species identification: describe how each sampled species was confirmed (diagnostic morphology, DNA barcoding), voucher numbers, and deposition.

Reply: We added a sentence "The species were confirmed by morphological characters, and the photos for sequenced individuals were shown in Figure 1." In Methods.

Biological replicates: clearly state how many male and female individuals were sequenced per species (genome and transcriptome). If single-individual per sex was used, acknowledge this limitation explicitly.

Reply: For HiFi and HiC sequencing, only one female adult was used for each species. For T. sinensis, a male adult and another female adult were used for Illumina sequencing. For transcriptome sequencing, the seven different tissues from each female adult for each species were used. There is no replicate for transcriptome sequencing, as these data were mainly used to facilitate gene annotation. We have clarified these information in the revised manuscript.

Transcriptome tissues: two "midfoot" entries appear; the latter likely means hindfoot—please correct.

Reply: Corrected.

P316: "for each mantis, the protein sequences from the other 4 mantises were …"—list the four species explicitly to remove ambiguity. (maybe 5 mantises)

Reply: We revised this sentence into "for each mantis, the protein sequences from the other 4 sequenced mantises in this study were".

5) X-chromosome identification and strength of inference
Depth-based identification in T. sinensis is reasonable, but provide normalized male/female coverage ratios (e.g., CQ) with sliding-window plots, variance/CI, and GC/repeat normalization; specify the number of replicates per sex. For the other genera, assignments based on comparative synteny should be described as inference, not definitive identification.

Reply: Thanks for the reviewer's kind suggestions. We have shown the coverage depth values for all the sliding windows of each chromosome in Figure 3a, it is easy to see the variance of the depth. The average depth was shown as a red line for each chromosome, and the coverage ratio for chromosome 1-12 and chromosome X1-X2 is about 2 for the male. When seeing Figure 3a, it is easy to make a solid conclusion that X1 and X2 are the two sex chromosomes. There is no replicate for each sex. To be more rigorous, we changed "Macroscale synteny analysis identified the corresponding

X chromosomes in the other 4 species" into "Macroscale synteny analysis inferred the corresponding X chromosomes in the other 4 species".

Avoid "confirmed"; use "supports a model in which …" and separate plausible scenarios (fusion vs. fission vs. translocation). Acknowledge limited taxon sampling (two Mantidae spp.).

Reply: We changed "we confirmed" into "our results support a model in which". We changed "resulted from the fragmentation and fusion of one X chromosome and an autosome" into "from the translocation between one X chromosome and an autosome". We also added "Based on very limited taxon sampling, we made a set of preliminary conclusions in this study, which will be verified by future studies as more genomes being sequenced." in the Discussion part.

6) Breakpoint localization claim (~2.5 Mb)
Provide methods and support: genomic coordinates, anchor density, long-read overlaps across the putative break, and Hi-C contact changes; include an uncertainty interval. Clarify whether this interval is species-specific or an inference for the Mantidae ancestor.

Reply: Thanks for the reviewer's kind suggestion. In the old manuscript, we firstly used macroscale synteny to locate the breakpoint region, then we used genomic sequence alignment to further narrow down the range. In this revised manuscript, we found that small inversions, deletions, and other rearrangements occurred in these regions, making the conclusion from genomic sequence alignment not accurate. Therefore, we decide to only report the conservative result obtained from macroscale synteny analysis, which we believe to be confidence. M. religiosa was not suitable for this analysis, as its current genome assembly is too fragmental, and the old Fig. S10 was removed in the revised manuscript. This time, we only used T. sinensis for performing this analysis, and tone down our conclusion. We changed Fig 4F in the old manuscript into Fig S10 in the revised manuscript.

We revised the description in Results: "Furthermore, based on macroscale synteny analysis, we were able to identify the breakpoints as a site falling within a 6.65-Mb region on the X1 chromosome and a site falling within 2.56-Mb region on the X2 chromosome of T. sinensis. Inside these two regions, transposon and tandem repeats dominate the sequence (Figure S10), posing great difficulties for accurate genome assembly and inter-species genomic sequence alignment. In future, as the assembly continuity improves, it is possible to narrow down the breakpoint range, which will approach or surpass the resolution of traditional cytological technologies such as C-banding, silver staining and living-cell images of the meiosis process [16, 23]."

We also revised the legend of Fig S10: "Figure S10. Location of the broken site for translocation on the X1 and X2 chromosomes of T. sinensis. TE refers to transposable element, and TR refers to tandem repeats. The gene density was shown in heatmap, while the TE/TR density were shown in distribution curves. Through macroscale synteny analysis between T. sinensis and D. truncata using reciprocal-best orthologous genes as markers, the broken site on ChrX1 (length 318,123,395 bp) was located to a 6.65 Mb region (107,057,327- 113,703,447 bp), and the broken site on ChrX2 (length 396,970,917 bp) was located to a 2.56 Mb region (171,940,730-174,498,078 bp). X1L and X2L were derived from ancestral X chromosome, while X1R and X2R were derived from ancestral autosome. Each broken site region has an assembly gap inside, which are filled with absolute 1,000 N characters. Therefore, the size estimation of broken site region was not so accurate. In addition, there are abundant TR and TEs in the broken site range, making it difficult to identify the accurate position where translocation happened. M. religiosa was not suitable for this analysis, because its current genome assembly is too fragmental.".

7) Heterozygosity vs. contiguity (discussion too thin)
The statement that lower contiguity in M. religiosa/T. sinensis is due to higher heterozygosity needs numbers (k-mer heterozygosity estimates and spectra) and

Powered by Editorial Manager® and ProduXion Manager® from Aries Systems Corporation

clarification on phasing/purge_dups/trio-binning. Report exact contig N50/N90 and % duplication before/after purging. Expand the discussion with appropriate citations.

Reply: The contig length statistics does not change much before/after purging duplications. The main reason for contig length in this study is heterozygosity, which was shown in the peak heights of Kmer frequency curves in Figure S3.

We added a sentence in the legend of Figure S3: “With higher heterozygous rate, the left/right peak height ratios will be larger. The ratio of the left/right peak heights show that M. religiosa and T. sinensis have much higher heterozygosity than the other 3 mantis species.”.

We also added a sentence “Based on GCE heterozygosity model [15], the estimated heterozygous rates are approaching 2% for M. religiosa and T. sinensis, whereas about 0.05-1% for the other three species.” in the maintext.

8) Phylogeny and rate heterogeneity
Define the substitution-rate analysis (loci, clock model, calibrations, tests of rate constancy, CIs).

Reply: In our substitution-rate analysis, the input data was the multiple-aligned protein sequences of single-copy genes from each species. Then, command “raxmlHPC -m PROTGAMMALGX” was applied. In the model “PROTGAMMALGX”, “PROT” means protein, “GAMMA” means gamma distribution, “LG” refers to the amino acid substitution model, “X” means maximum likelihood. We have added more descriptions for “PROTGAMMALGX” in the method part.

Rephrase the teleological sentence: instead of "allowing M. violaceus to conserve more ancestral traits," use "consistent with retention of ancestral gene content/signals."

Reply: We changed “allowing M. violaceus to conserve more ancestral traits” into “consistent with its retention of ancestral traits.”.

The statement that M. violaceus "shares more orthologous genes with cockroaches" requires a precise metric (e.g., number of 1:1 orthologs from OrthoFinder; control for annotation size).

Reply: This conclusion is based on Figure 4A, which shows the percentage of orthologous groups (OG) shared with cockroach for each mantis species. We have added more description in the legend of Figure 4A: “When an OG contain one or more genes for both an analyzed mantis and the cockroach, it was counted for the analyzed mantis as shared OGs.”.

For the Deroplatys placement, report support (UFBoot/SH-aLRT/ASTRAL LPP; gene/site-concordance factors) and briefly discuss potential discordance (ILS/introgression) given rapid radiations.

Reply: Thanks very much for the reviewer’s suggestions. For the Deroplatys placement, our analysis can only give a very preliminary conclusion, mainly due to the very limited taxon sampling. Thus, it is out of our ability and interest to perform more professional analyses on phylogeny assignment.

9) The manuscript requires a thorough language edit by a fluent scientific editor.

Reply: We have tried our best to improve the language.

Reviewer #2: The manuscript presents chromosome-level genome assemblies for five species of mantis, describing their patterns of synteny and phylogenetic relationships. Most of the conclusions are well supported and the manuscript well written. The genomes will serve as a useful resource for future ecological and evolutionary studies in this group and beyond. Overall, I recommend it for publication in GigaScience.

Powered by Editorial Manager® and ProduXion Manager® from Aries Systems Corporation

However, several aspects need to be addressed first.

Major comments:
- The title needs to be changed as while the manuscript describes the evolution of additional sex chromosomes, there is no investigation into sex determination mechanisms. E.g. replace 'sex determination mechanism evolution' with 'sex chromosome evolution'.

Reply: Corrected.

- Without dating the tree, substitution rates and time are confounded, preventing the authors from making comments on substitution rates. Moreover, the large distances of evolutionary time between lineages make inferring substitution rates extremely hard. These claims should therefore be revised.

Reply: To explain how to estimate the substitution rate, we added a sentence in the maintext "Generally, the branch length is in proportional to the amino-acid substitution rate."

Besides, we added divergence time results in the maintext: "The divergence time was inferred by the Reltime-Branch lengths method, using one calibration constraint (70-80 Mya between M.violacea and M.religiosa). The results showed that four modern mantises (D.truncata, H.coronatus, M.religiosa, and T.sinensis) emerged within a short time period (26-31 Mya) (Figure S11), posing difficulties for phylogenic inferrence within this lineage." To emphasis this point, the original Figure S11 was moved to be Figure 4C.

- As the level of confidence in the phylogeny was not explored, the discussion should make it clear that more detailed phylogenomic analyses are required to get at some of the interesting questions that they would like to investigate in future.

Reply: We added "Based on very limited taxon sampling, we made a set of preliminary conclusions in this study, which will be verified by future studies as more genomes being sequenced." In the Discussion part.

- The generation of the Hi-C data is missing from the methods.

Reply: We added "Then, Hi-C data was generated using the same individuals applied for HiFi sequencing. Nuclear DNA was cross-linked by soaking leaf tissues in formaldehyde solution, and the cross-linked genomic DNA was extracted, digested, repaired, ligated to circular fragments, sheared into 350 bp inserts, converted to short-read sequencing library by Truseq DNA Library Prep Kit, and sequenced on Illumina NovaSeq 6000 platform (RRID:SCR_016387)." in the Methods.

- It is unclear to me why a different method for scaffolding was used for the genomes with fewer contigs. Please explain why the protocol used for the other genomes (the Arima mapping pipeline and YaHS) would not have been appropriate.

Reply: EndHiC is a software developed by our group, it was published in BMC bioinformatics in year 2022. EndHiC is especially suitable to construct chromosome-level scaffolds with relatively larger contigs (T. sinensis, D. truncata, H. coronatus, M. violacea), but it is not suitable for much shorter contigs (M. religiosa). So we turned to use YaHS, a more general algorithm, to resolve scaffolding for M. religiosa, whose contigs are very fragmental.

Minor comments

- The supplementary contains figures (e.g. figure S10-12) and includes methods that are not mentioned in the main text. Please make the supplementary consistent with the

main text and adapt numbering accordingly.

Reply: Corrected.

Abstract:
- "Metallyticus violacea" should be changed to "Metallyticus violacea" as this version is used everywhere else in the text and used in NCBI's taxonomy.

Reply: We have changed all “violaceus” into “violacea”.

- "deduced that translocations between" should be changed to "suggest that translocations between" as the data suggests a translocation but does not prove it.
Reply: Corrected.

- The term 'Deroplatys' is used in the abstract without contextualising what this group is, please briefly describe what this is for readers unfamiliar with the taxonomy. This is also the case in the introduction.
Reply: We added “genus” after “'Deroplatys'”, to show it is a genus.

Introduction:
- Please explain in the introduction the significance/impact of understanding the relationships within Mantodea.

Reply: We added a sentence “Clarifying the relationships within Mantodea will greatly benefit the functional studies as well as genetic breeding of mantises” in Introduction.

Results:
- The results list useful statistics for each species (lines 79-96). However, this information would be much better suited to a table to allow comparison between species and statistics. Ranges in the results could suffice to summaries the table. It is important for downstream analyses of sex chromosomes that females were sequenced (i.e. lacked any Y chromosomes).

Reply: Thanks for the reviewer’s kind suggestion. In the whole manuscript, we describe each species separately. To be consistent in the style, we insist to list the numbers for each species in our study. All these information are also available in Table 1 and Table S2, which are suitable for the readers to make comparison.

- Please briefly explain the GCE method in the text for those less familiar with this.

Reply: We changed “Using the GCE method,” to “Using the GCE method that estimates genome size with K-mer frequency distribution from sequencing reads”.

- When comparing the genomes of H. coronatus from this study and from another study, please explain why the X chromosome is better assembled here. For example, was manual curation performed and does this explain the difference in quality?

Reply: Our assembly of X chromosome is complete, while it was broken into 3 fragments in Huang’s assembly. This is the main difference.

- Given that the authors describe a strong correlation between genome size and repeat content, I suggest adapting Fig S7 which plots these two variables as a graph as part of Fig1/2, adding a statistical test for the strength of correlation while accounting for shared ancestry (i.e. including the effect of phylogeny when testing for correlation) to verify this relationship.

Reply: According to the reviewer’s suggestion, we have moved one plot of Fig S7 into Figure 2. We calculated the Pearson's correlation coefficient (r), and made a t-test to verify its independence.
The results were shown in the legends of Figure 2A “A linear trend Line in average of

all the points was shown. The Pearson's correlation coefficient (r) is as high as 0.99578, with t-test p-value < 0.001, suggesting that there is very strong correlation between genome size and TE content." and Fig S7 "A linear trend Line in average of all the points was shown. The Pearson's correlation coefficient (r) is as high as 0.894, with t-test p≈0.038<0.05, suggesting that there is strong correlation between genome size and TE ratio.".

- The authors write "Sex chromosomes evolved from autosomes and play important roles in tissue development, mating, and speciation [17-19]." which is not quite true, sex chromosomes can also evolve by other means e.g. from B chromosomes. Please revise this.

Reply: This sentence was revised to "Sex chromosomes evolved from autosomes or B chromosomes, and play important roles in tissue development, mating, and speciation".

- It would be valuable to add a short description of the types of chromosome rearrangements that are suggested to have occurred in these genomes (based on the synteny plot), to shape the varying karyotypes of 2n=14-21. At the moment, only sex chromosomes are described but the autosomes have also experienced changes.

Reply: We added short descriptions of chromosome rearrangements in the legends of Figure 3: "(B) The synteny band plot among T. sinensis, D. truncata and M. violacea, much more chromosome rearrangements were observed between M. violacea and the other mantises. (C) The dual synteny between M. religiosa and T. sinensis, all chromosomes have 1:1 relationship. (D) The dual synteny between D. truncata and H. coronatus, including 4 chromosome brokens and 3 inter-chromosome translocations.".

The statements about sex chromosome systems across insects need supporting with references

Reply: We have revised the reference in the revised manuscript "Sex chromosomes evolved from autosomes or B chromosomes, and play important roles in tissue development, mating, and speciation [17]. The types of sex chromosomes found in insects vary among species, and sex chromosome systems exhibit significant diversity across insect species [18-20].".

- When describing the sex chromosome result of T. sinensis it should be mentioned that the genome was generated from a female (i.e. lacked any Y chromosomes) and hence two half coverage chromosomes in male are two Xs (rather than X/Y for example).

Reply: We added a sentence in the maintext "In addition, the genome assembly of T. sinensis was derived from a female individual, which lacked the Y chromosome.".

- Some claims are too speculative based on the available data e.g. "M. violaceus shares much more OGs with cockroaches than the other 4 species (69% vs 61-63%) (Figure 4A), which may partly explain its strong morphological resemblance to cockroaches." (line 208) and similarly in the discussion "M. violaceus genome shares more orthologous genes with cockroaches than with the other mantises, and the mutation rate of M. violaceus genome is the lowest among all the mantises, allowing M. violaceus to conserve more ancestral traits." (line 244). This is also reflected in the title of this section being too strong "Low evolutionary rate allows M. violaceus conserve more ancestral traits" (line 194). Also, the authors do not describe the traits of M. violaceus which are "more ancestral".

Reply: We have tone-down on these conclusions, and described the ancestral traits for M. violacea in maintext "M. violacea belongs to the superfamily Metallyticoidea, exhibiting significant morphological differences compared to other mantises. M. violacea shares many characteristics with its cockroach relatives, including dull body

Powered by Editorial Manager® and ProduXion Manager® from Aries Systems Corporation

colouration, a prostrate body posture, and a relatively shorter prothorax. The cockroach-like body morphology was thought to be the ancestral traits of Mantodea (order).”.

- It would strengthen this section if the authors could assess the confidence in the assignment of D. truncata as sister to H. coronatus, or else make it clear that further analyses are needed.

Reply: Due to limited taxon sampling, it is difficult to assess the confidence in the assignment of D. truncata. Instead, we have added a sentence “Based on very limited taxon sampling, we made a set of preliminary conclusions in this study, which will be verified by future studies as more genomes being sequenced.” in Discussions.

- Please comment on why the N50 and N90 are so much higher in your assemblies than in previously published genomes e.g. differences in sequencing technology, assembly methods etc (line 228).

Reply: We added a comment sentence in the maintext “The difference in assembly continuity is manily caused by the applied sequencing technologies. We used HiFi long reads with over 99% accuracy, while they used common long reads with only ~85% accuracy.”.

- Heterozygosity is mentioned in a few places without being supported by any values (e.g. line 231)

Reply: We added the estimation of heterozygosity values in the maintext “Based on GCE heterozygosity model [15], the estimated heterozygous rates are approaching 2% for M. religiosa and T. sinensis, whereas about 0.05-1% for the other three species.”.

- Please could you clarify what you mean by "tissues with no or low heterozygosity are still a priority for de novo genome sequencing." and make it clearer that high heterozygosity can be problematic for genome assembly (line 232)

Reply: We have changed “tissues with no or low heterozygosity are still a priority for de novo genome sequencing” into “high heterozygosity can be problematic for genome assembly”.

- Please can you elaborate why Mantodea occupies "an important position in the evolution of insects" (line 235)

Reply: We revised this sentence into “Mantodea has occupied an important position in the evolution of insects, with distinctive morphology and camouflage behavior.”.

Comments on figures:
- Fig1F: the category of duplicated BUSCOs are missing, please add this. This also needs a Y-axis label.

Reply: We have divided complete genes (C) into single copy genes (S) and duplicated genes (D), and added a Y-axis label “Percent of Busco genes”.

- Fig2A: Please add a short description of how to interpret a radar chart and the main conclusion it supports.

Reply: We added a short description: “Each vertex refers to a type of TE, and species were differed by colors. LINEs, Tc1-IS630-Pogo, and Helitron have sharp peaks, indicating a burst of TEs for that type.” In the legend of Fig2A.

Powered by Editorial Manager® and ProduXion Manager® from Aries Systems Corporation

- Fig 2: As all panels have the same key I suggest having it just once and making it clear it applies to all panels in the figure legend. Please make the Y-axis in panels B,C and D more informative (i.e. counts of what)

Reply: The legend for Fig 2B,C,D was changed to “(B-D) The divergence (%) distribution of LINE, Tc1, and Helitron, respectively.”. For the Y-axis, “counts” was changed to “count of TE copies”

- Fig 3: The 'X chromosome' in the title should be plural given more than one evolved.
Reply: We changed “X chromosome” into “chromosomes”.

- Fig 3A: Please add an Y-axis label.
Reply: We added a Y-axis label “Depth”.

- Fig 3B-D: Please include details about the orthologs that are plotted i.e. total number per plot and what these orthologs are e.g. all single copy orthologues in both species.

Reply: We have added the plotted ortholog gene number in the figure legend: “(B) The synteny band plot among T. sinensis, D. truncata and M. violacea, using 9,117 reciprocal best orthologous genes between T. sinensis and D. truncata, as well as 8,765 reciprocal best orthologous genes between D. truncata and M. violacea. Much more chromosome rearrangements were observed between M. violacea and the other mantises. (C) The dual synteny between M. religiosa and T. sinensis, using 9,917 reciprocal best orthologous genes between M. religiosa and T. sinensis. All chromosomes have 1:1 relationship. (D) The dual synteny between D. truncata and H. coronatus, using 9,821 reciprocal best orthologous genes between D. truncata and H. coronatus. Four chromosome brokens and three inter-chromosome translocations were observed.”.

- Fig3B: The colouring of T. sinensis is strange. It would be helpful if X1 is the same colour as X to indicate shared orthologs.

Reply: We have updated the colors in Fig 3B-D, using black color for all the X chromosomes, and grep color for all the autosomes. This color scheme is also consistent with Fig 3E.
We added a sentence in the Fig 3 legend: “For B-E, black color represents for X chromosomes, while gray color represents for autosomes.”.

- Fig 3E: Please annotate the figure with the node where the hypothesised translocation occurred and label the resulting chromosomes as X chromosomes. Black should be changed to 'X1-chromosome' given that an X2 is made in the figure but is half grey half black due to it being part ancestral autosome and part sex chromosome.

Reply: We have labeled the node that translocation occurred on Fig 3E, and also labeled all the X, X1, X2 chromosomes in Fig 3E.

- Fig 3F: Please increase the space between the key for gene number and that for TR and TE as it is hard to understand at present. Please also explain in the figure legend what TR and TE denote and their relevance to the breakpoint. It would also be helpful to note in the legend how this figure illustrates how the breakpoint was identified.

Reply: We have increased the space between key for gene number and that for TR and TE. We also added a sentence in legend of Fig 3F “TE refer to transposable element, TR refer to tandem repeats. Within the broken site range, TE and TR dominate the sequence.”.

- Fig 4: I think that the figure legend title ("Orthologous groups and phylogeny of Mantodea") could be more informative. Also please clarify whether the following is referencing all OG groups in cockroaches that are found in each mantis, or that of mantis OGs found in cockroaches: "Percentage of orthologous groups (OG) shared with cockroach for each mantis species". Please add the substitution model used in

maximum-likelihood inference to the legend and add the scale that the branch lengths are given in.

Reply: We changed the figure legend title "Orthologous groups and phylogeny of Mantodea" into "Evolution of Mantodea lineages". We added a descriptive sentence in the legend "When an OG contain one or more genes for both an analyzed mantis and the cockroach, it was counted for the analyzed mantis as shared OGs.". We added "using LG amino acid substitution model" in the legend. The scale (length 0.1) was shown on the right-bottom corner of Figure 4B.

- Table 1: It would be helpful to add identified sex chromosomes and the sex that the genome is derived from.
Reply: Thanks for the reviewer's suggestion, but we think Table 1 is quite large now, showing statistics for the assembly and annotation. The sex chromosomes of each mantis have been clearly shown in Figure 3.

- Fig S11: Please add more details to the legend e.g. which model was used in maximum-likelihood (ML). Also, in the methods, two ML approaches are described while this figure shows one tree from ML and one from NJ. Please ensure the figure and methods are consistent. Also the case for Fig S13.

Reply: We have revised the Methods to describe the two methods in maintext: "Using the concatenated super protein sequence alignment, RAxML (v8.2.12) (RAxML, RRID:SCR_006086) was subsequently employed to construct a maximum-likelihood phylogenetic tree with the PROTGAMMALGX ("PROT" means protein sequence, "GAMMA" means gamma distribution, "LG" refers to amino acid substitution model, "X" means maximum likelihood estimation) model [47]. To verify the topology of the ML-tree, a NJ-tree was also constructed using the neighbor joining algorithm in MEGA (X) (MEGA, RRID:SCR_002805) [49]."

In Fig S11, we used another phylogeny building method (NJ, neighbor-joining) to test whether its result is consistent that of ML method (Figure 4B). If the two trees are consistent, the result will be more confident. We have added more description in the supplementary figure legend "The phylogenetic tree was built based on concatenated protein alignment from 6,988 single-copy orthologs (mantises), with NJ (neighbor joining) algorithm in MEGA. The topology of NJ phylogeny tree is largely consistent the ML (maximum likelihood) tree in Figure 4B.".
In addition, the legend of Fig S13 (now Fig S12) was revised into "The phylogenetic tree was built based on 2201 single-copy orthologs, with maximum likelihood methods ("raxmlHPC -m PROTGAMMALGX"). Dlob: D.lobata, Dtru: D. truncata, Hcor: H. coronatus, Mrel: M. religiosa,Tsin: T. sinensis, Mvio: M. violacea ,Zoot: Z. nevadensis, Bger: B. germanica.".

- Table S1: References are needed for the karyotype information.

Reply: We have added reference information for karyotype in the legend of Table S1: "The chromosome number of M. religiosa was obtained from del Cerro's paper (Synaptonemal complex analysis of the X1X2Y trivalent in Mantis religiosa L. males: inferences on the origin and maintenance of the sex-determining mechanism. Chromosome Research. 1998;6:5-11), while the chromosome number of T. sinensis was obtained from Yuan R'paper (The chromosome-level genome of Chinese praying mantis Tenodera sinensis (Mantodea: Mantidae) reveals its biology as a predator. GigaScience. 2023;12). The chromosome number of D. truncata, H. coronatus, and M. violacea were determined by genome assembly, as no karyotype information was available for these 3 mantis species.".

Methods
- The kits used for preparing libraries for illumina and RNA sequencing should be added. Also a description of the Hi-C data generation is missing.
Reply: The kits for DNA and RNA sequencing library construction are Truseq DNA Sample Prep Kit (illumina) and Truseq RNA Sample Prep Kit (illumina), respectively. We have added them in the Methods. We also added the Hi-C data generation in

Powered by Editorial Manager® and ProduXion Manager® from Aries Systems Corporation

	Methods "Then, Hi-C data was generated using the same individuals applied for HiFi sequencing. Nuclear DNA was cross-linked by soaking leaf tissues in formaldehyde solution, and the cross-linked genomic DNA was extracted, digested, repaired, ligated to circular fragments, sheared into 350 bp inserts, converted to short-read sequencing library by Truseq DNA Library Prep Kit, and sequenced on Illumina NovaSeq 6000 platform (RRID:SCR_016387).".<br><br>- Please add to line 322 that this analysis is referring to the identification of the X chromosome specifically in T. sinensis<br>Reply: We have revised "To identify the X chromosome" into "To identify the X chromosome of T. sinensis".<br><br>- Please briefly mention your justification of model choice and add the expansion of the RAxML notation of PROTGAMMALGX for those not familiar with this shorthand (i.e. LG + G4 + Fest)<br>Reply: We have used a relatively older but widely applied version of RAxML (Command "raxmlHPC -m PROTGAMMALGX"), and we added more description about it in Methods:<br>"PROTGAMMALGX ("PROT" means protein sequence, "GAMMA" means gamma distribution, "LG" refers to amino acid substitution model, "X" means maximum likelihood estimation) model".<br><br>- Please note how codon alignment was performed (line 361).<br>Reply: We have removed this sentence in the revised manuscript.<br><br>Suggested rephrasings:<br>- Valuable repository' with 'valuable resource' (line 35)<br>Reply: Corrected.<br><br>- Missing plural in last sentence of abstract "Mantodea and genetic breeding of efficient enemy insect" (line 37)<br>Reply: Corrected.<br><br>- An "A" or "The" is missing here: "Phylogenetic tree was constructed…" (line 209 and 216)<br>Reply: "The" was added before "phylogenetic tree" in these two places.<br><br>- "breakpoint to less than a 2.5-Mb range" (line 240) I think this should be "breakpoint to a less than 2.5Mb range"<br>Reply: Corrected.<br><br>- I suggest "phylogeny clarification" should be "phylogenetic clarification" (line 247)<br>Reply: Corrected.<br><br>- 'Whose contigs are more fragmental' could be 'whose assembly was fragmented into more contigs' (line 286)<br>Reply: Corrected.<br>--<br>Please also take a moment to check our website at for any additional comments that were saved as attachments. Please note that as GigaScience has a policy of open peer review, you will be able to see the names of the reviewers.<br>____<br>In compliance with data protection regulations, you may request that we remove your personal registration details at any time. (Use the following URL: https://www.editorialmanager.com/giga/login.asp?a=r). Please contact the publication office if you have any questions.
**Additional Information:**	
**Question**	**Response**
Are you submitting this manuscript to a special series or article collection?	No
**Experimental design and statistics**	Yes

Full details of the experimental design and statistical methods used should be given in the Methods section, as detailed in our Minimum Standards Reporting Checklist. Information essential to interpreting the data presented should be made available in the figure legends.<br><br>Have you included all the information requested in your manuscript?	
**Resources**<br><br>A description of all resources used, including antibodies, cell lines, animals and software tools, with enough information to allow them to be uniquely identified, should be included in the Methods section. Authors are strongly encouraged to cite Research Resource Identifiers (RRIDs) for antibodies, model organisms and tools, where possible.<br><br>Have you included the information requested as detailed in our Minimum Standards Reporting Checklist?	Yes
**Availability of data and materials**<br><br>All datasets and code on which the conclusions of the paper rely must be either included in your submission or deposited in publicly available repositories (where available and ethically appropriate), referencing such data using a unique identifier in the references and in the "Availability of Data and Materials" section of your manuscript.<br><br>Have you have met the above requirement as detailed in our Minimum Standards Reporting Checklist?	Yes
GigaScience has policies and guidelines	No

Powered by Editorial Manager® and ProduXion Manager® from Aries Systems Corporation

in place for the use of generative AI-writing tools such as ChatGPT. If you have used such writing tools to assist with writing the manuscript this must be declared and cited in the text. Authors should not list AI-writing tools and other AI-assisted technologies as an author or co-author and should acknowledge that they are fully responsible for text generated or refined by AI-writing tools.<p><br><br>A summary of use (particularly in the introduction or among methods) needs to be included at the end of the paper, and the outputs should also be included as a supplementary file hosted in GigaDB or other open repositories. Please read our guidelines for more information. <p><br><br>By submitting to GigaScience, you are aware of the journal's AI-writing tools policy, and if you have declared use of such tools below, you have acknowledged this where appropriate in your manuscript and have made a summary of use and outputs available. <p><br><b>AI-assisted writing tools have been used in the preparation of this manuscript?	

Powered by Editorial Manager® and ProduXion Manager® from Aries Systems Corporation

Manuscript Click here to access/download;Manuscript;Manuscript - R1.docx

The genomes of five mantises provide insights into evolution of sex chromosome sex determination mechanism and evolution of Mantodea lineages

Hangwei Liu[1,2,†], Lihong Lei[1,3,4,†], Fan Jiang[1], Bo Zhang[1], Hengchao Wang[1], Yutong Zhang[3], Hanbo Zhao[1], Guirong Wang[1,5,*] & Wei Fan[1,*]

[1] Guangdong Laboratory for Lingnan Modern Agriculture (Shenzhen Branch), Genome Analysis Laboratory of the Ministry of Agriculture and Rural Affairs, Agricultural Genomics Institute at Shenzhen, Chinese Academy of Agricultural Sciences, Shenzhen, Guangdong, 518120, China.

[2] College of Plant Protection, Yangzhou University, Yangzhou 225009, China

[3] School of Life Sciences, Henan University, Kaifeng 475004, China

[4] Shenzhen Research Institute of Henan University, Shenzhen 518000, China

[5] State Key Laboratory for Biology of Plant Diseases and Insect Pests, Institute of Plant Protection, Chinese Academy of Agricultural Sciences, Beijing, China.

†These authors contributed equally to this work.

* Correspondence should be addressed to wangguirong@caas.cn and fanwei@caas.cn.

Abstract

Background Praying mantises, members of the order Mantodea, play important roles in agriculture, medicine, bionics, and entertainment. However, the scarcity of genomic resources has hindered extensive studies on mantis evolution and behaviour.

Results Here, we present the chromosome-scale reference genomes of five mantis species: the European mantis (*Mantis religiosa*), Chinese mantis (*Tenodera sinensis*), triangle dead leaf mantis (*Deroplatys truncata*), orchid mantis (*Hymenopus coronatus*),

and metallic mantis (*Metallyticus* ~~*violaceus*~~*violacea*). The assembled genome sizes range ~2.3-4.2 Gb, with contig N50 size 1-109 Mb and 85-99% of sequence anchored to chromosomes. The annotated protein-coding gene number ranges 17,804-19,017, with BUSCO complete rate 96.7-98.4%. We found that transposable element expansion is the major force governing genome size in Mantodea, and ~~deduced~~ suggest that translocations between the X chromosome and an autosome have occurred in the lineage of the ~~super~~family Mantidae. In addition, we found a much lower substitution rate for the lineage of *M.* ~~*violaceus*~~*violacea* than the lineages of other mantises. Furthermore, our genome-wide analyses showed that *D. truncata* is ~~closer~~ sister to *H. coronatus* than *M. religiosa* and *T. sinensis*, ~~providing important evidences for~~helps resolve~~ing~~ the phylogenic controversies of *Deroplatys* genus.

Conclusions The high-quality genome assemblies of the five mantises provide a valuable resource ~~repository~~ for evolution studies of Mantodea and genetic improvement and breeding of beneficial biological control agents.~~genetic breeding of efficient enemy insect.~~

Keywords: Mantodea, genome, transposable element, X1X2Y, evolution

Background

Praying mantises are familiar insects that play important roles in agriculture, medicine, and bionics. As predators of many harmful insect species, praying mantises such as the European mantis (*Mantis religiosa*) and Chinese mantis (*Tenodera sinensis*) are widely acknowledged as natural enemies that control plant pests[1], benefiting organic planting where pesticide is prohibited. The mantis ootheca (egg capsule, egg chamber) is a traditional medicine used to cure frequent micturition, strengthen kidney health and prevent spermatorrhea in East Asian countries [2]. Most praying mantises have two sharp and strong forelegs, which are much larger and more powerful than their ancient ancestors. In addition, the femur and tibia of the forelegs are armed with strong spines along their posterior edges. When the femur and tibia fold on each other, a praying mantis can firmly grasp the prey. This distinctive body structure of the praying mantis

has been a significant source of inspiration in bionics of cutting blades [3, 4]. Although most manties have the common XY sex determination system, some mantises such as *M. religiosa* and *T. sinensis* have the X1X2Y type [5-7], making them a special material for studying the evolution of sex chromosomes.

The two closely related orders, Mantodea (mantises) and Blattodea (cockroaches and termites), are classified into the superorder Dictyoptera, and phylogenomic analyses revealed that Mantodea split from Blattodea during the Permian [8]. Mantodea has evolved into a group comprising ~2500 species with diverse morphological and ecological characteristics, with the highest diversity in tropical and subtropical habitats [9, 10]. From fossils of early Mantodea and Blattodea species, the common ancestor is thought to resemble modern cockroaches in many aspects [11]. The metallic mantis (*Metallyticus* ~~*violaceus*~~*violacea*), in the early diverging mantid lineage, has many morphological features similar to those of modern cockroaches, but the underlying mechanisms have not been discovered [12]. Although Mantodea are well supported as monophyletic, the phylogenetic relationships within Mantodea are still not well resolved. ~~. Morphological and molecular phylogenies are often inconsistent with previous classification schemes for some sublineages such as~~For example, *Deroplatys* genus has been placed into Mantidae family traditionally, but these species are more similar to species in Hymenopodidae family in many aspects such as morphology and camouflage ~~, because of the rapid radiation events and convergent evolution of ecomorphological strategies~~ [9, 13]. Clarifying the relationships within Mantodea will greatly benefit the functional studies as well as genetic breeding of mantises.

Compared with those of many other insect orders, the genomic resources of Mantodea are very limited, with only three chromosome-scale reference genomes available: the Chinese mantis (*T. sinensis*), orchid mantis (*H. coronatus*) and Malaysian dead leaf mantis (*Deroplatys lobata*) [14, 15]. Here, we present the chromosome-scale reference genomes of three other mantis species, the European mantis (*M. religiosa*), triangle dead leaf mantis (*D. truncata*), and metallic mantis (*M.* ~~*violaceus*~~*violacea*), as

Formatted: Font: Not Italic

Formatted: Pattern: Clear (White)

well as a more complete assembly of *T. sinensis* and *H. coronatus*, to promote the evolutionary and biological studies of Mantodea.

Results

Chromosome-scale genome assemblies of five mantis species

We generated 179 Gb (48X) (*M. religiosa*), 97 Gb (36X) (*T. sinensis*), 112 Gb (26X) (*D. truncata*), 177 Gb (56X) (*H. coronatus*), and 147 Gb (63X) (*M. ~~violaceus~~violacea*) PacBio HiFi data, and 127 Gb (35X) (*M. religiosa*), 112 Gb (42X) (*T. sinensis*), 153 Gb (35X) (*D. truncata*), 182 Gb (58X) (*H. coronatus*), and 183 Gb (79X) (*M. ~~violaceus~~violacea*) Illumina Hi-C data (Table S1, S2). The PacBio HiFi reads were used to assemble the contig sequences, with a total size of 3.6 Gb (*M. religiosa*), 2.6 Gb (*T. sinensis*), 4.2 Gb (*D. truncata*), 3.1 Gb (*H. coronatus*), and 2.3 Gb (*M. ~~violaceus~~violacea*) and N50 sizes of 1 Mb (*M. religiosa*), 13 Mb (*T. sinensis*), 44 Mb (*D. truncata*), 71 Mb (*H. coronatus*), and 109 Mb (*M. ~~violaceus~~violacea*). The Illumina Hi-C reads were mapped to the contig sequences, and the valid Hi-C read pairs were used for scaffolding assembly (Table S3), resulting in 85.39% (*M. religiosa*), 95.63% (*T. sinensis*), 97.47% (*D. truncata*), 98.27% (*H. coronatus*), and 98.51% (*M. ~~violaceus~~violacea*) of the contig sequences anchored into 14 (*M. religiosa*), 14 (*T. sinensis*), 16 (*D. truncata*), 21 (*H. coronatus*), and 17 (*M. ~~violaceus~~violacea*) chromosome-level scaffolds (Figure 1A-E, S1, S2; Table 1, S4). Notably, only the chromosome numbers for *M. religiosa* and *T. sinensis* have been karyotyped [16, 17], whereas the others are inferred only from the genome assembly. Using the GCE method that estimates genome size with K-mer frequency distribution from sequencing reads [18], the estimated genome sizes are 3.5 Gb (*M. religiosa*), 2.8 Gb (*T. sinensis*), 4.3 Gb (*D. truncata*), 3.1 Gb (*H. coronatus*), and 2.3 Gb (*M. ~~violaceus~~violacea*), consistent with assembled genome sizes. Based on GCE heterozygosity model [18], the estimated heterozygous rates are approaching 2% for *M. religiosa* and *T. sinensis*, whereas about 0.05-1% for the other three species. Owing to the higher heterozygosity rate (Figure S3), the contig sizes for the *M. religiosa* and *T. sinensis* are shorter than those for the other three species.

Recently, Huang et al. published a reference genome for *H. coronatus*, with a much shorter contig N50 size of 15.7 Mb [14], and Yuan et al. published a reference genome of *T. sinensis* with a contig N50 size of 2.36 Mb, which is also much shorter than that of this study [15]. The difference in assembly continuity is manily caused by the applied sequencing technologies. We used HiFi long reads with over 99% accuracy, while they used common long reads with only ~85% accuracy. From syntenic alignments of the two assemblies for *H. coronatus*, we found that most chromosomes were largely consistent except for the X chromosome (Figure S4). One complete X chromosome in our assembly corresponds to 3 fragmented chromosomes in Huang's assembly. The X chromosome is the largest chromosome, making it more difficult to assemble than the autosomes. Thus, our assembly of the X chromosome for *H. coronatus* is likely more complete. We also compared another reference genome published by the Huang group [14], the Malaysian dead leaf mantis (*Deroplatys lobata*, CRA010804 in the National Genomics Data Center, China), to our assembled reference genome of *D. truncata* (Figure S5). Belonging to the same genus, most chromosomes have high synteny, except for four chromosomes involved in chromosome-level rearrangements, which are more likely due to species divergence than assembly errors. Both reference genomes of *T. sinensis* (Yuan et al.[15] and the present study) showed high synteny for all chromosomes (Figure S6).

By integrating homology and transcription evidence, 19,017 (*M. religiosa*), 19,007 (*T. sinensis*), 18,156 (*D. truncata*), 18,536 (*H. coronatus*) and 17,804 (*M. ~~violaceus~~violacea*) protein-coding gene models were annotated as the reference genes (Table 1, Table S5). The BUSCO complete rates for the reference genes of these mantis species range from 96.7%-98.4% (Figure 1F), which are higher than or comparable to those of previously published mantis genomes [14] [15]. Furthermore, 97.2%-98.6% of the reference genes in these five mantis species were assigned functions according to at least one of the NCBI-NR, KEGG, InterPro or GO databases.

Distinct TE expansions in various mantid lineages

Formatted: Font: Not Italic

Increasing evidence has shown that transposable elements (TEs) contribute significantly to the genome size and influence the genome architecture, along with insertions, deletions, translocations, etc [19]. We analyzed the total TE content (ratio) among the 5 species and found that genome size was linearly correlated with TE abundance (Figure 2A, S7). Currently, Mantodea (order) and Blattodea (order) are placed within Dictyoptera (superorder). Mantidae is a representative mantis family, which belongs to Mantoidea (superfamily) in Mantodea. The two Mantidae species (*M. religiosa* and *T. sinensis*) have relatively smaller genome sizes (2.3-2.8 Gb) and lower TE contents (58-63%), than the other 3 mantises, with relatively larger genome sizes (3.1-3.5 Gb) and higher TE contents (67-68%), suggesting that genome size differences are mostly determined by TE contents in mantids.

Abundant retrotransposons, DNA transposons and rolling-circle transposons were found in these mantis genomes, however, their ratios in genome differ across species (Figure 2B~~A~~, Table S6,S7). For the two Mantidae species, LINEs are the largest components, and a sharp expansion of LINEs with divergence of approx. 7% was found in *M. religiosa* (Figure ~~2B~~2C). However, no recent large-scale expansion of LINEs has occurred in *T. sinensis*, which may explain why its genome size (2.8 Gb) is smaller than that of *M. religiosa* (3.5 Gb). In contrast, *D. truncata* and *H. coronatus* have massive DNA transposons, with Tc1 (especially Tc1-IS630-Pogo) being the largest component in these two species, consistent with the findings of a former study [14]. *D. truncata* has undergone both a recent sharp expansion and an ancient burst of Tc1 in its genome, leading to the largest genome size (4.3 Gb) found in this study, whereas only an ancient explosion of Tc1 was observed in *H. coronatus* (Figure ~~2C~~2D). Both *D. truncata* and *H. coronatus* also have a large rolling-circle transposon, *Helitrons*. Both a recent and an ancient burst of *Helitron* were observed in *D. truncata*, whereas only an ancient burst of *Helitron* was found in *H. coronatus* (Figure 2E~~D~~). *M. ~~violaceus~~violacea* shows no recent accumulation of any category of TEs, which may explain why its genome size (2.3 Gb) was the smallest among these mantises.

These results collectively suggest that TE expansion is the major force behind genome size variation in Mantodea. In addition, the components and divergence times

of the various TE types are distinct among the different mantid lineages.

Translocation between X chromosome and autosomes in Mantidae lineage

Sex chromosomes evolved from autosomes or B chromosomes, and play important roles in tissue development, mating, and speciation~~–~~ [20]~~[5-7, 20]~~. The types of sex chromosomes found in insects vary among species, and sex chromosome systems exhibit significant diversity across insect species [5-7]. Most insects have XY, ZW or XO sex chromosome systems, but there are other rare sex chromosome types, such as the X1X2Y type, which has two X chromosomes and one Y chromosome. Some hemipterans such as *Philaenus italosignus* [21] and some mantids such as *M. religiosa* [22], exhibit this sex chromosome type.

To identify the X chromosomes from the assembled pseudochromosomes, we generated 15X short-read sequencing data for the female and male *T. sinensis* individuals, respectively. Sequencing coverage revealed that all 14 chromosomes in female have comparable coverage depths, whereas in male, two chromosomes have approximately half the coverage depth (Figure 3A). It has been reported that most members of the ~~super~~family Mantidae have two X chromosomes, X1 and X2, derived from fusion or translocation between the X chromosome and an autosome [16]. In addition, the genome assembly of *T. sinensis* was derived from a female individual, which lacked the Y chromosome. Thus, the two chromosomes with half coverage depths are concluded to be the X1 and X2 chromosomes. Notably, they are the largest and second largest of our assembled pseudochromosomes, consistent with previous reports based on karyotyping [16, 23].

Macroscale synteny analysis identified the corresponding X chromosomes in the other 4 species, and allowed comparative analysis among the mantid X chromosomes. Synteny alignments revealed that both Mantidae species *M. religiosa* and *T. sinensis* have two sex chromosomes, X1 and X2; however, the other species have only one sex chromosome X. In addition, only part of X1 (X1L) and X2 (X2L) in Mantidae were aligned with the X chromosomes of the other 3 species (Figure 3B-D, S8). These results suggest that the ancestral mantid had one X chromosome and that the translocation of

large fragments between the X chromosome and an autosome occurred in Mantidae (Figure 3E). Previous studies have revealed that the common ancestor of Dictyoptera had a XY sex chromosome system [16, 23] (Figure S9). We inferred that the common ancestor of the Mantidae family evolved the X1X2Y sex chromosome system, and ~~we~~ our results ~~confirmed that~~support a model in which the generation of the X1 and X2 chromosomes resulted from the ~~fragmentation and fusion~~translocation between~~of~~ one X chromosome and an autosome.

Furthermore, based on – macroscale synteny analysis~~conserved sequence alignments~~, we were able to identify the breakpoints ~~range~~ as a site falling within a 6.65~~2.5~~-Mb region on the X1 chromosome and a site falling within 2.56-Mb region on the X2 chromosome of *T. sinensis*~~(Figure 3F, S10)~~. Inside these two regions, transposon and tandem repeats dominate the sequence (Figure S10), posing great difficulties for accurate genome assembly and inter-species genomic sequence alignment. In future, as the assembly continuity improves, it is possible to narrow down the breakpoint range, which will approach or surpass the resolution of traditional cytological technologies ~~Resolution by sequencing is much greater than that previously obtained via cytological techniques,~~ such as C-banding, silver staining and living-cell images of the meiosis process [16, 23].

Formatted: Font color: Auto

Formatted: Font: Italic, Font color: Auto

Formatted: Font color: Auto

Formatted: Font color: Auto

Formatted: Font color: Auto

Formatted: Font color: Auto

Formatted: Font color: Auto

Formatted: Font color: Auto

Formatted: Font color: Auto

Formatted: Font color: Auto

Formatted: Font color: Auto

Low evolutionary rate may allow~~s~~ *M.* ~~*violaceus*~~*violacea* conserve more ancestral traits

Comparative analysis of Mantodea genomes within a phylogenetic context is essential for understanding their evolution and diversity. Phylogenomic analyses were performed on these 5 Mantodea species, which span 5 genera and 3 families with diverse habitats and morphologies. Two Blattodea species, the German cockroach (*Blattella germanica*) [24] and the dampwood termite (*Zootermopsis nevadensis*) [25], were used as the outgroup (Table S8). From gene family clustering, 69,603 orthologous groups (OGs) were generated, including 4,014 single-copy OGs.

M. ~~*violaceus*~~*violacea* belongs to the superfamily Metallyticoidea, exhibiting

significant morphological differences compared to other mantises. *M. violacea* ~~and~~ shares many characteristics with its cockroach relatives~~ but exhibits significant morphological differences compared to other mantises~~, including dull body colouration, a prostrate body posture, and a relatively shorter prothorax. The cockroach-like body morphology was thought to be the ancestral traits of Mantodea (order). The Metallyticoidea lineage is sister to the other mantis lineages [26]. *M.* ~~*violaceus*~~*violacea* shares much more OGs with cockroaches than the other 4 species (69% vs 61-63%) (Figure 4A), which may partly explain its strong morphological resemblance to cockroaches.

The p~~P~~hylogenetic tree was constructed based on 4,014 single-copy OGs, and the substitution rates and divergence time along branches were estimated. Generally, the branch length is in proportional to the amino-acid substitution rate. The amino-acid substitution rate for *M.* ~~*violaceus*~~*violacea* branch was the lowest among those of Mantodea (Figure 4B), which may indicate that the evolution rate of *M.* ~~*violaceus*~~*violacea* branch was much slower than that of the other mantises. With a slower evolution rate, *M.* ~~*violaceus*~~*violacea* may preserve more characteristics of the Mantodea ancestor, which also partly explains its morphological resemblance to cockroaches. The divergence time was inferred by the Reltime-Branch lengths method, using one calibration constraint (70-80 Mya between M.violacea and M.religiosa). The results showed that four modern mantises (D.truncata, H.coronatus, M.religiosa, and T.sinensis) emerged within a short time period (26-31 Mya) (Figure 4C), posing difficulties for phylogenic inference within this lineage.

The p~~P~~hylogenetic tree also revealed that *D. truncata* is closer to *H. coronatus* (Hymenopodidae) than *M. religiosa and T. sinensis* (Mantidea), differing from the phylogenic assignment from some previous studies that place *D. truncata* within Mantidea (Figure 4B, ~~S11, ~~S1~~2~~1) [9, 13]. After adding the genomic data for *D. lobata*, both Deroplatys species sistered to *H. coronatus* (Figure ~~S13~~S12). Therefore, genome-wide data is helpful to clarify phylogeny controversies, providing important evidences for further species classification of Deroplatys.

Formatted: Font: Not Bold

Discussion

In this study, we generated chromosome-level genome assemblies for 5 mantis species via a combination of PacBio HiFi and Hi-C sequencing technologies. For *H. coronatus* and *T. sinensis*, both the contig N50 and N90 sizes of our assembly are approximately 5 times greater than those of the previously published reference genomes [14, 15]. In our results, assembly continuity for *M. religiosa* and *T. sinensis* is relatively lower than that for the other 3 mantises due to the differences in heterozygosity, suggesting that high heterozygosity can be problematic for genome assembly~~tissues with no or low heterozygosity are still a priority for *de novo* genome sequencing~~. Compared with those of cockroaches and termites, the much larger genome sizes of mantises are mainly the result of expansions of various types of transposable elements.

Mantodea has occupied an important position in the evolution of insects, with distinctive morphology and camouflage behavior. ~~and~~ O~~o~~ne of its major sublineages, the ~~super~~family Mantidae, has a special X1X2Y sex determination system. Through comparative genomics analysis, we inferred that the mantid common ancestor had only one X chromosome and translocation between the X chromosome and an autosome occurred in the ancestor of Mantidae. ~~We were able to narrow this breakpoint to less than a 2.5-Mb range on the original X chromosome, which will promote studies of sex determination systems and chromosome evolution.~~ *M.* ~~*violaceus*~~*violacea* genome shares more orthologous genes with cockroaches than with the other mantises, and the mutation rate of *M.* ~~*violaceus*~~*violacea* genome is the lowest among all the mantises, consistent with its retention of ancestral traits.~~allowing *M. violaceus* to conserve more ancestral traits.~~ Our phylogenetic analyses with genome-wide data also suggest that the two *Deroplatys* species are closer to *H. coronatus* than to the two Mantidea mantises, which may do some help for further phylogenetic~~y~~ clarification and accurate species classification of *Deroplatys*. Based on very limited taxon sampling, we made a set of preliminary conclusions in this study, which will be verified by future studies as more genomes being sequenced.

Although praying mantises are efficient predators, their hunting objects are not

Formatted: Font color: Custom Color(RGB(238,0,0))

specific to harmful insects, hindering their wide application in organic planting. Thus, the genomic resources generated in this study will also facilitate molecular breeding of the praying mantis, in order to make it a more applicable nature enemy insect.

Methods

Insect collection and sequencing

Mantis adults were collected at different locations: *M. religiosa* and *T. sinensis* from the forest of Guangzhou, China; *H. coronatus* from the rainforest of Xishuangbanna, China; and *D. truncata* and *M.* ~~*violaceus*~~*violacea* from two captive breeding centers in Beijing, China. The species were confirmed by morphological characters, and the photos for sequenced individuals were shown in Figure 1. All mantis samples for sequencing had the intestine removed to avoid contamination by bacteria, fungi, and residual prey bodies. All the tissues were cleaned with 30% ethanol and ddH_2O, and then immersed in liquid nitrogen for cryopreservation.

For Pacific Biosciences (PacBio) HiFi sequencing, libraries with ~15 kb insert sizes were constructed from a female adult of every mantis, and sequenced on a PacBio Sequel II system (RRID: SCR_017990). Subreads were generated with an N50 size of 14.5 kb, and consensus reads (CCS reads) were generated via ccs software (v.3.0.0) [27] with the following parameters: -min-passes 0 -min-rq 0.99 -min-length 100 -max-length 50,000. Then, Hi-C data was generated using the same individuals applied for HiFi sequencing. Nuclear DNA was cross-linked by soaking leaf tissues in formaldehyde solution, and the cross-linked genomic DNA was extracted, digested, repaired, ligated to circular fragments, sheared into 350 bp inserts, converted to short-read sequencing library by Truseq DNA Library Prep Kit, and sequenced on Illumina NovaSeq 6000 platform (RRID:SCR_016387). To identify the sex chromosome, ~~For Illumina~~short-read sequencing of a male adult and another female adult of *T. sinensis*~~, two short paired-end DNA library with a 400 bp insert size was constructed via standard Illumina protocols respectively, and sequenced~~ was performed on ~~an~~ Illumina NovaSeq 6000 platform (RRID:SCR_016387), using DNA library with a 400 bp insert size

constructed via Truseq DNA Sample Prep Kit (Illumina).~~.~~

Total RNA from the abdomen, hind leg, middle leg, foreleg, thorax, head, and eye~~head, eye, thorax, abdomen, forefoot, midfoot and midfoot~~ of a female adult for each species~~s~~ were~~as~~ extracted with TRIzol reagent (Invitrogen), and used to construct cDNA libraries with Truseq RNA Sample Prep Kit (illumina). Transcriptome sequencing data were generated via the Illumina NovaSeq 6000 system in PE150 mode.

Genome assembly and quality assessment

K-mer frequencies from HiFi reads of five mantises were calculated via Kmerfreq (https://github.com/fanagislab/kmerfreq), and then genome sizes were estimated via GCE (GCE, RRID:SCR_017332). The PacBio HiFi reads were assembled into contigs via Hifiasm (v0.14) (Hifiasm, RRID:SCR_021069)[28] with the following parameters: -l 1 -s 0.7. To filter duplicated contigs in the assembly, purge_dups (v1.2.3) (purge dups, RRID:SCR_021173) [27] was adopted with the following parameters: -2 -a 50. The completeness of the assembly was evaluated using BUSCO (v5.2.2) (BUSCO, RRID:SCR_015008) based on the OrthoDB (v10) (OrthoDB, RRID:SCR_011980) Insecta database [29].

For Hi-C scaffolding, two strategies were applied. For *M. religiosa*, whose assembly was fragmented into more contigs~~whose contigs are more fragmental~~, Hi-C reads were mapped to contigs via the Arima mapping pipeline (https://github.com/ArimaGenomics/mapping_pipeline), and then, YaHS (v1.2a.1) (YaHs, RRID:SCR_0229650) [30] was used to assemble the contigs into pseudo chromosomes. For the other four mantises, whose contigs are much larger, Hi-C reads were mapped to contigs by Bowtie 2 (v 2.2.2.7) (Bowtie 2, RRID:SCR_016368) [31], then HiC-Pro (v2.11.0-beta) (HiC-Pro, RRID:SCR_017643) [32] was adopted to identify valid ligation pairs and generate Hi-C link matrices among different contigs, and finally, the contigs were clustered, ordered, and oriented into pseudo-chromosomes using EndHiC (v1.0) (EndHiC, RRID:SCR_022110) [33] based on the Hi-C linkage information among contig ends.

Genome annotation

A *de novo* transposable element (TE) library was constructed with RepeatModeler (v2.0.2) (RepeatModeler, RRID:SCR_015027) with the parameters -engine ncbi-database [34], and then RepeatMasker (v4.1.0) (RepeatMasker, RRID:SCR_012954) was used to identify TEs in the reference genome, using both the *de novo* TE library and the public Repbase TE library (v26.05) (Repbase, RRID:SCR_021169). The tandem repeat elements in the genome were subsequently identified using Tandem Repeats Finder (TRF) (Tandem Repeats Finder, RRID:SCR_022193) (v4.09) [35].

The protein-coding gene models were annotated in two rounds. In the first round, the genes were predicted by integrating evidence from *de novo* gene predictions and transcriptome-based gene predictions. *De novo* gene prediction was performed on the TE-masked genome assembly with AUGUSTUS (v3.4.0) (Augustus, RRID:SCR_008417) [36]. For transcriptome-based gene prediction, the RNA-seq data were filtered by Fastp (v0.23.1) (fastp, RRID:SCR_016962) [37]and then mapped to the genome using Bowtie2 (v2.2.7) [31], and StringTie (v1.3.3b) (StringTie, RRID:SCR_016323) was then used to construct the gene models [38]. All the gene models obtained via the above two approaches were subsequently integrated with EVidenceModeler (v1.1.1) (EVidenceModeler, RRID:SCR_014659) [39]. In the second round, for each mantis, the protein sequences from the other 4 sequenced mantises in this study were mapped to this genome assembly with Exonerate (v2.4.0) (Exonerate, RRID:SCR_016088) [40], and incomplete gene models were filtered. Finally, for each mantis, the *de novo* gene predictions, the transcriptome-based gene predictions, and the homology-based gene predictions were integrated with EVidenceModeler (v1.1.1) to generate a high-confidence and nonredundant gene set.

The completeness of the gene sets was assessed using BUSCO based on OrthoDB (v10) for Insecta. For gene functional annotation, the mantis protein sequences were aligned to the KEGG (KEGG, RRID:SCR_012773), eggNOG (eggNOG, RRID:SCR_002456), NR, and UniProt (SwissProt) databases using DIAMOND (v0.9.24.125) (DIAMOND, RRID:SCR_009457) [41], and only the best hits with E-values less than 1e^{-5} were retained. Moreover, InterProScan (v5.38) (InterProScan,

RRID:SCR_005829) was used to annotate the protein domains and GO (Gene Ontology) terms [42].

X chromosome identification and analysis

To identify the X chromosome of *T. sinensis*, the clean Illumina paired reads from female and male samples were mapped to the genome of *T. sinensis* via BWA (v0.7.17-r1188) (BWA, RRID:SCR_010910) [43]. The bam files were filtered using SAMtools (v1.6) (SAMTOOLS, RRID:SCR_002105) [44] with the parameters '-q 60 -F 1804', and paired reads mapped onto different chromosomes were also filtered. To assess the sequencing depth of each chromosome, SAMtools depth (v1.6) was used to calculate the average base coverage. The two chromosomes in males whose sequencing depth was approximately half that of the other chromosomes, were identified as X-derived chromosomes. For consistency with the karyotype results for *T. sinensis* [22] and *M. religiosa* [16], the larger one was denoted X2, whereas the smaller one was denoted X1.

Pairwise collinearity analyses were conducted using the protein sequences of five mantis species as markers. DIAMOND (v0.9.24.125) with the parameters 'blastp -f 6' was used to align the protein sequences of each species pair, and the reciprocal best pairs were used as inputs for MCScanX (MCScanX, RRID:SCR_022067) to identify syntenic blocks [45]. The inter species syntenic genomic blocks were visualized via the R package Ideogram [46]. Based on the collinearity alignments of the five mantises, the X chromosomes of the other four mantises were also identified. In addition, the translocation sites on chromosomes X1 and X2 were inferred from the collinearity alignment.

Evolutionary analysis

Seven Dictyoptera species, including the five mantises sequenced in this study, as well as *B. germanica* (PRJNA203136 in NCBI) [24] and *Z. nevadensis* (PRJNA203242 in NCBI) [25], were used to construct orthologous groups (OG) and infer orthologous genes via OrthoFinder (v2.5.4) (OrthoFinder, RRID:SCR_017118) with the default parameters [47]. The protein sequences of single-copy genes from each species were

multiple aligned using MAFFT (v7.487) (MAFFT, RRID:SCR_011811) and then concatenated into one super protein sequence. Using the concatenated super protein sequence alignment, RAxML (v8.2.12) (RAxML, RRID:SCR_006086) was subsequently employed to construct a maximum-likelihood phylogenetic tree with the PROTGAMMALGX (“PROT” means protein sequence, “GAMMA” means gamma distribution, “LG” refers to amino acid substitution model, “X” means maximum likelihood estimation) model~~, and codon alignment of the super protein sequence were used to construct a maximum-likelihood phylogenetic tree with the GTRGAMMA model~~ [48]. To verify the topology of the ML-tree, a NJ-tree was also constructed using the neighbor joining algorithm in MEGA (X) (MEGA, RRID:SCR_002805) [49].

~~.~~

Abbreviations

BLAST: Basic Local Alignment Search Tool; bp: base pairs; BUSCO: Benchmarking Universal Single-Copy Orthologs; BWA: Burrows-Wheeler Aligner; CCS: circular consensus sequencing; Gb: gigabase pairs; GO: Gene Ontology; kb: kilobase pairs; KEGG: Kyoto Encyclopedia of Genes and Genomes; Ma: megaannus; Mb: megabase pairs; MYA: million years ago; NCBI: National Center for Biotechnology Information; NR: Non-Redundant; OG: orthologous groups; PacBio: Pacific Biosciences; PE: Paired end; RAxML: Randomized Axelerated Maximum Likelihood; TRF: Tandem Repeats Finder; TE: transposable element; TPM: transcripts per million; YaHS: yet another Hi-C scaffolding.

Acknowledgments

This work was supported by Shenzhen Science and Technology Program (Grant No. KQTD20180411143628272), ~~and Projects subsidized by Special Funds for Science Technology Innovation and Industrial Development of Shenzhen Dapeng New District (Grant No. PT202101-02);~~ Fund of Key Laboratory of Shenzhen (ZDSYS20141118170111640), and The Agricultural Science and Technology Innovation Program.

Data availability

The genomic and transcriptomic sequencing reads have been deposited in NCBI-SRA under the accession PRJNA987019, PRJNA989593, PRJNA989036, PRJNA988270, PRJNA989282 for *M. religiosa*, *T. sinensis*, *D. truncata*, *H. coronatus* and *M. ~~violaceus~~violacea*, respectively. The corresponding genome assemblies and annotations have been deposited at NCBI-Genome under the accessions JAUKNK000000000, JAUKNM000000000, JAUKNL000000000, JAUKNX000000000, JAUJEO000000000, and are also available at Figshare (10.6084/m9.figshare.23995398, 10.6084/m9.figshare.23995410, 10.6084/m9.figshare.23995152, 10.6084/m9.figshare.23988987, 10.6084/m9.figshare.23995434).

Author contributions

H.L. and L.L. prepared the sequencing samples, performed data analysis, and wrote the raw manuscript. W.F. and G.W. supervised the project and revised the manuscript. The other authors provided helpful suggestions, and all authors read and approved the final version of this manuscript.

Competing interests

The authors declare no competing interest.

Figures and tables

Figure 1. **Overall view of genome assembly and annotation**. Circos plots for *M. religiosa.* (A) *T. sinensis* (B) *D. truncata* (C) *H. coronatus* (D) and *M. ~~violaceus~~violacea* (E). Each circos plot has 4 tracks: track A represents chromosome length, track B represents gene density, track C represents transposable element (TE) density, and track D represents GC percentage. Feature density and GC percentage were calculated by sliding 1-Mb windows. (F) BUSCO assessment (database: Insecta from OrthoDB v10) of gene sets for five mantis species. M means missing, F means fragmented, and C means complete.

Figure 2. **TE distribution in five mantis genomes.** (A) The relationship between genome size

and TE content. A linear trend Line in average of all the points was shown. The Pearson's correlation coefficient (r) is as high as 0.99578, with t-test p-value < 0.001, suggesting that there is very strong correlation between genome size and TE content. (B~~A~~) The radar chart for major components of TE. Each vertex refers to a type of TE, and species were differed by colors. LINEs, Tc1-IS630-Pogo, and Helitron have sharp peaks, indicating a burst of TEs for that type. (C-E) The divergence (%) distribution of LINE, Tc1, and *Helitron*~~.~~, respectively. ~~(B) The divergence (%) distribution of LINE. (C) The divergence (%) distribution of Tc1. (D) The divergence (%) distribution of *Helitron*.~~

Figure 3. **Evolution of X chromosomes in Mantodea.** (A) Identification of X chromosome in *T. sinensis* by comparing depths between male and female individual. The sequencing depth distributions were plotted in 500 Kb windows. The red line represents the average sequencing depth for each chromosome. (B) The synteny band plot among *T. sinensis*, *D. truncata* and *M.* ~~*violaceus*~~*violacea*~~.~~, using 9,117 reciprocal best orthologous genes between *T. sinensis* and *D. truncata*, as well as 8,765 reciprocal best orthologous genes between *D. truncata* and *M. violacea*. Much more chromosome rearrangements were observed between *M. violacea* and the other mantises. (C) The dual synteny between *M. religiosa* and *T. sinensis*~~.~~, using 9,917 reciprocal best orthologous genes between *M. religiosa* and *T. sinensis*. All chromosomes have 1:1 relationship. (D) The dual synteny between *D. truncata* and *H. coronatus*~~.~~, using 9,821 reciprocal best orthologous genes between *D. truncata* and *H. coronatus*. Four chromosome brokens and three inter-chromosome translocations were observed. ~~–~~(E) The diagram shows the evolutionary process of the X chromosome along various lineages of Mantodea. ~~(F) The range of the broken site for translocation on the X1 and X2 chromosomes of *T. sinensis*, which are 2.51 Mb and 0.02 Mb, respectively.~~For B-E, black color represents for X chromosomes, while gray color represents for autosomes.

Figure 4. ~~**Orthologous groups and phylogeny**~~**Evolution of Mantodea lineages**. (A) Percentage of orthologous groups (OG) shared with cockroach for each mantis species. When an OG contain one or more genes for both an analyzed mantis and the cockroach, it was

Formatted: Font: Not Italic

Formatted: Font color: Auto

counted for the analyzed mantis as shared OGs. (B) Phylogeny is based on ~~codon~~ protein sequence alignment of 4,014 single copy genes (mantises, cockroaches, and termites) with Maximum Likelihood (ML) method using LG amino acid substitution model. The branch length is in proportional with the substitution rate. (C) The time tree was inferred using the Reltime-Branch lengths method in MEGA, with input of user-supplied branch lengths derived from Maximum Likelihood (ML) method. The time tree was computed using 1 calibration constraint (70-80 Mya between M.violacea and M.religiosa, doi: 10.1126/science.1257570).

Table 1. Statistics of genome assembly and annotation

**Genomic features**	***M. religiosa***	***T. sinensis***	***D. truncata***	***H. coronatus***	***M. ~~violaceus~~violacea***
**Genome assembly**					
Estimated genome size by K-mer (bp)	**3,519,843,697**	**2,865,686,147**	**4,337,798,490**	**3,167,239,197**	**2,331,221,057**
Total assembly size (bp)	**3,680,002,721**	**2,687,426,722**	**4,290,792,545**	**3,127,590,514**	**2,322,129,794**
Contig N50 size (bp)	**1,407,320**	**12,728,340**	**44,444,664**	**71,519,735**	**109,157,195**
Scaffold N50 size (bp)	**210,326,877**	**190,002,057**	**248,405,437**	**159,059,693**	**125,733,329**
# of assembly-inferred chromosomes	**14**	**14**	**16**	**21**	**17**
% sequence anchored to chromosome	**85.39%**	**95.63%**	**97.47%**	**98.27%**	**98.51%**
**Genome annotation**					
Length and % of tandem sequences (bp)	**396,842,330 (10.8%)**	**403,304,947 (15.0%)**	**471,243,565 (11.0%)**	**238,530,960 (7.6%)**	**186,949,249 (8.1%)**
Length and % of TE sequences (bp)	**2,501,898,483 (68%)**	**1,710,668,926 (64%)**	**2,928,636,453 (68%)**	**2,122,785,940 (68%)**	**1,351,077,317 (58%)**
Number of protein-coding gene models	**19,017**	**19,007**	**18,156**	**18,536**	**17,804**
Mean CDS length (bp)	**1551**	**1782**	**1601**	**1523**	**1152**

Mean exon number	**6.07**	**5.93**	**6.34**	**6.33**	**5.54**

References

1. Rankin EEW, Shmerling AJ, Knowlton JL and Hoey-Chamberlain R. Diets of two non-native praying mantids (Tenodera sinensis and Mantis religiosa) show consumption of arthropods across all ecological roles. Food Webs. 2023;35 doi:ARTN e00280
10.1016/j.fooweb.2023.e00280.

2. Song JH, Cha JM, Moon BC, Kim WJ, Yang S and Choi G. Mantidis Ootheca (mantis egg case) original species identification via morphological analysis and DNA barcoding. J Ethnopharmacol. 2020;252 doi:ARTN 112574
10.1016/j.jep.2020.112574.

3. Yu HY, Han ZW, Zhang JQ and Zhang SJ. Bionic design of tools in cutting: Reducing adhesion, abrasion or friction. Wear. 2021;482 doi:ARTN 203955
10.1016/j.wear.2021.203955.

4. Li M, Yang YW, Guo L, Chen DH, Sun HL and Tong J. Design and Analysis of Bionic Cutting Blades Using Finite Element Method. Appl Bionics Biomech. 2015;2015 doi:Artn 471347
10.1155/2015/471347.

5. Bachtrog D, Kirkpatrick M, Mank JE, McDaniel SF, Pires JC, Rice W, et al. Are all sex chromosomes created equal? Trends in Genetics. 2011;27 9:350-7. doi:https://doi.org/10.1016/j.tig.2011.05.005.

6. Bachtrog D, Mank JE, Peichel CL, Kirkpatrick M, Otto SP, Ashman TL, et al. Sex determination: why so many ways of doing it? PLoS Biol. 2014;12 7:e1001899. doi:10.1371/journal.pbio.1001899.

7. Rowe L, Chenoweth SF and Agrawal AF. The Genomics of Sexual Conflict. Am Nat. 2018;192 2:274-86. doi:10.1086/698198.

8. Evangelista DA, Wipfler B, Bethoux O, Donath A, Fujita M, Kohli MK, et al. An integrative phylogenomic approach illuminates the evolutionary history of cockroaches and termites (Blattodea). Proc Biol Sci. 2019;286 1895:20182076. doi:10.1098/rspb.2018.2076.

9. Svenson GJ and Whiting MF. Reconstructing the origins of praying mantises (Dictyoptera, Mantodea): the roles of Gondwanan vicariance and morphological convergence. Cladistics. 2009;25 5:468-514. doi:10.1111/j.1096-0031.2009.00263.x.

10. Svenson GJ, Hardy NB, Wightman HMC and Wieland F. Of flowers and twigs: phylogenetic revision of the plant-mimicking praying mantises (Mantodea: Empusidae and Hymenopodidae) with a new suprageneric classification. Syst Entomol. 2015;40 4:789-834. doi:10.1111/syen.12134.

11. Hornig MK, Haug JT and Haug C. An exceptionally preserved 110 million years old praying mantis provides new insights into the predatory behaviour of early mantodeans. PeerJ. 2017;5:e3605. doi:10.7717/peerj.3605.

12. Fukui M, Fujita M, Tomizuka S, Mashimo Y, Shimizu S, Lee CY, et al. Egg structure and outline of embryonic development of the basal mantodean, Metallyticus splendidus Westwood, 1835 (Insecta, Mantodea, Metallyticidae). Arthropod Struct Dev. 2018;47 1:64-73. doi:10.1016/j.asd.2017.11.001.

13. Ma Y, Zhang LP, Lin YJ, Yu DN, Storey KB and Zhang JY. Phylogenetic relationships and divergence dating of Mantodea using mitochondrial phylogenomics. Syst Entomol. 2023; doi:10.1111/syen.12596.

14. Huang G, Song L, Du X, Huang X and Wei F. Evolutionary genomics of camouflage innovation in the orchid mantis. Nat Commun. 2023;14 1:4821. doi:10.1038/s41467-023-40355-1.
15. Yuan R, Zheng B, Li Z, Ma X, Shu X, Qu Q, et al. The chromosome-level genome of Chinese praying mantis Tenodera sinensis (Mantodea: Mantidae) reveals its biology as a predator. GigaScience. 2023;12 doi:10.1093/gigascience/giad090.
16. del Cerro AL, Cunado, N. & Santos, J.L. Synaptonemal complex analysis of the X1X2Y trivalent in Mantis religiosa L. males: inferences on the origin and maintenance of the sex-determining mechanism. Chromosome Research. 1998;6:5-11. doi:https://doi.org/10.1023/A:1009258122785.
17. Li XT and Nicklas RB. Mitotic Forces Control a Cell-Cycle Checkpoint. Nature. 1995;373 6515:630-2. doi:DOI 10.1038/373630a0.
18. Liu B, Shi Y, Yuan J, Hu X, Zhang H, Li N, et al. Estimation of genomic characteristics by analyzing k-mer frequency in de novo genome projects. arXiv: Genomics. 2013.
19. Elliott TA and Gregory TR. Do larger genomes contain more diverse transposable elements? Bmc Evol Biol. 2015;15 doi:ARTN 69
10.1186/s12862-015-0339-8.
20. Ellegren H. Sex-chromosome evolution: recent progress and the influence of male and female heterogamety (vol 12, pg 157, 2011). Nat Rev Genet. 2011;12 10:736-. doi:10.1038/nrg3081.
21. Maryańska-Nadachowska A, Kuznetsova VG, Lachowska D and Drosopoulos S. Mediterranean species of the spittlebug genus Philaenus : Modes of chromosome evolution. Journal of Insect Science. 2012;12 1 doi:10.1673/031.012.5401.
22. King R. Chromosomes of three species of mantidae. Journal of Morphology. 2005;52:525 - 33. doi:10.1002/jmor.1050520208.
23. Paliulis LV, Stowe EL, Hashemi L, Pedraza-Aguado N, Striese C, Tulok S, et al. Chromosome number, sex determination, and meiotic chromosome behavior in the praying mantid Hierodula membranacea. PLoS One. 2022;17 8:e0272978. doi:10.1371/journal.pone.0272978.
24. Harrison MC, Jongepier E, Robertson HM, Arning N, Bitard-Feildel T, Chao H, et al. Hemimetabolous genomes reveal molecular basis of termite eusociality. Nat Ecol Evol. 2018;2 3:557-66. doi:10.1038/s41559-017-0459-1.
25. Terrapon N, Li C, Robertson HM, Ji L, Meng X, Booth W, et al. Molecular traces of alternative social organization in a termite genome. Nature Communications. 2014;5 1:3636. doi:10.1038/ncomms4636.
26. Svenson GJ and Whiting MF. Phylogeny of Mantodea based on molecular data: evolution of a charismatic predator. 2004;29 3:359-70. doi:https://doi.org/10.1111/j.0307-6970.2004.00240.x.
27. Guan D, McCarthy SA, Wood J, Howe K, Wang Y and Durbin R. Identifying and removing haplotypic duplication in primary genome assemblies. Bioinformatics. 2020;36 9:2896-8. doi:10.1093/bioinformatics/btaa025 %J Bioinformatics.
28. Cheng H, Concepcion GT, Feng X, Zhang H and Li H. Haplotype-resolved de novo assembly using phased assembly graphs with hifiasm. Nature Methods. 2021;18 2:170-5. doi:10.1038/s41592-020-01056-5.
29. Simão FA, Waterhouse RM, Ioannidis P, Kriventseva EV and Zdobnov EM. BUSCO: assessing genome assembly and annotation completeness with single-copy orthologs. Bioinformatics. 2015;31 19:3210-2. doi:10.1093/bioinformatics/btv351 %J Bioinformatics.
30. Zhou C, McCarthy SA and Durbin R. YaHS: yet another Hi-C scaffolding tool. Bioinformatics.

2023;39 1 doi:10.1093/bioinformatics/btac808.

31. Langmead B and Salzberg SL. Fast gapped-read alignment with Bowtie 2. Nat Methods. 2012;9 4:357-9. doi:10.1038/nmeth.1923.

32. Servant N, Varoquaux N, Lajoie BR, Viara E, Chen CJ, Vert JP, et al. HiC-Pro: an optimized and flexible pipeline for Hi-C data processing. Genome Biol. 2015;16:259. doi:10.1186/s13059-015-0831-x.

33. Wang S, Wang H, Jiang F, Wang A, Liu H, Zhao H, et al. EndHiC: assemble large contigs into chromosome-level scaffolds using the Hi-C links from contig ends. BMC Bioinformatics. 2022;23 1:528. doi:10.1186/s12859-022-05087-x.

34. Flynn JM, Hubley R, Goubert C, Rosen J, Clark AG, Feschotte C, et al. RepeatModeler2 for automated genomic discovery of transposable element families. Proceedings of the National Academy of Sciences. 2020;117 17:9451-7. doi:10.1073/pnas.1921046117.

35. Benson G. Tandem repeats finder: a program to analyze DNA sequences. Nucleic Acids Res. 1999;27 2:573-80. doi:10.1093/nar/27.2.573 %J Nucleic Acids Research.

36. Stanke M, Keller O, Gunduz I, Hayes A, Waack S and Morgenstern B. AUGUSTUS: ab initio prediction of alternative transcripts. Nucleic Acids Research. 2006;34 suppl_2:W435-W9. doi:10.1093/nar/gkl200 %J Nucleic Acids Research.

37. Chen S, Zhou Y, Chen Y and Gu J. fastp: an ultra-fast all-in-one FASTQ preprocessor. Bioinformatics. 2018;34 17:i884-i90. doi:10.1093/bioinformatics/bty560 %J Bioinformatics.

38. Pertea M, Pertea GM, Antonescu CM, Chang TC, Mendell JT and Salzberg SL. StringTie enables improved reconstruction of a transcriptome from RNA-seq reads. Nat Biotechnol. 2015;33 3:290-5. doi:10.1038/nbt.3122.

39. Haas BJ, Salzberg SL, Zhu W, Pertea M, Allen JE, Orvis J, et al. Automated eukaryotic gene structure annotation using EVidenceModeler and the Program to Assemble Spliced Alignments. Genome Biology. 2008;9 1:R7. doi:10.1186/gb-2008-9-1-r7.

40. Slater GS and Birney E. Automated generation of heuristics for biological sequence comparison. BMC Bioinformatics. 2005;6:31. doi:10.1186/1471-2105-6-31.

41. Buchfink B, Reuter K and Drost HG. Sensitive protein alignments at tree-of-life scale using DIAMOND. Nat Methods. 2021;18 4:366-8. doi:10.1038/s41592-021-01101-x.

42. Jones P, Binns D, Chang HY, Fraser M, Li W, McAnulla C, et al. InterProScan 5: genome-scale protein function classification. Bioinformatics. 2014;30 9:1236-40. doi:10.1093/bioinformatics/btu031.

43. Li H and Durbin R. Fast and accurate short read alignment with Burrows-Wheeler transform. Bioinformatics. 2009;25 14:1754-60. doi:10.1093/bioinformatics/btp324.

44. Li H, Handsaker B, Wysoker A, Fennell T, Ruan J, Homer N, et al. The Sequence Alignment/Map format and SAMtools. Bioinformatics. 2009;25 16:2078-9. doi:10.1093/bioinformatics/btp352.

45. Wang Y, Tang H, Debarry JD, Tan X, Li J, Wang X, et al. MCScanX: a toolkit for detection and evolutionary analysis of gene synteny and collinearity. Nucleic Acids Res. 2012;40 7:e49. doi:10.1093/nar/gkr1293.

46. Hao Z, Lv D, Ge Y, Shi J, Weijers D, Yu G, et al. RIdeogram: drawing SVG graphics to visualize and map genome-wide data on the idiograms. PeerJ Comput Sci. 2020;6:e251. doi:10.7717/peerj-cs.251.

47. Emms DM and Kelly S. OrthoFinder: phylogenetic orthology inference for comparative

genomics. Genome Biol. 2019;20 1:238. doi:10.1186/s13059-019-1832-y.
48. Kozlov AM, Darriba D, Flouri T, Morel B and Stamatakis A. RAxML-NG: a fast, scalable and user-friendly tool for maximum likelihood phylogenetic inference. Bioinformatics. 2019;35 21:4453-5. doi:10.1093/bioinformatics/btz305.
49. Kumar S, Stecher G, Li M, Knyaz C and Tamura K. MEGA X: Molecular Evolutionary Genetics Analysis across Computing Platforms. Mol Biol Evol. 2018;35 6:1547-9. doi:10.1093/molbev/msy096.

Click here to access/download;Figure;Figure1.pdf

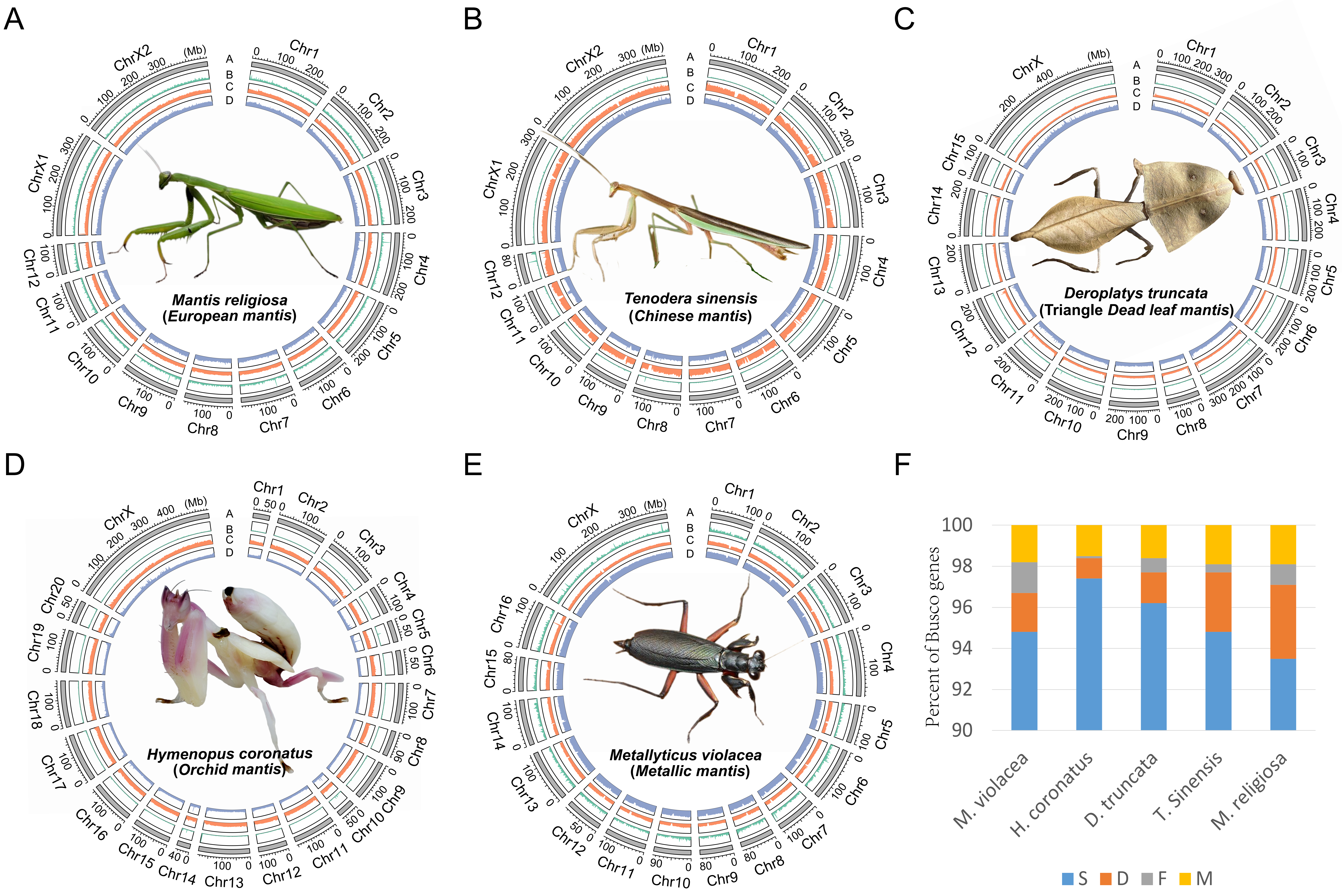

Figure 2

Click here to access/download;Figure;Figure2.pdf

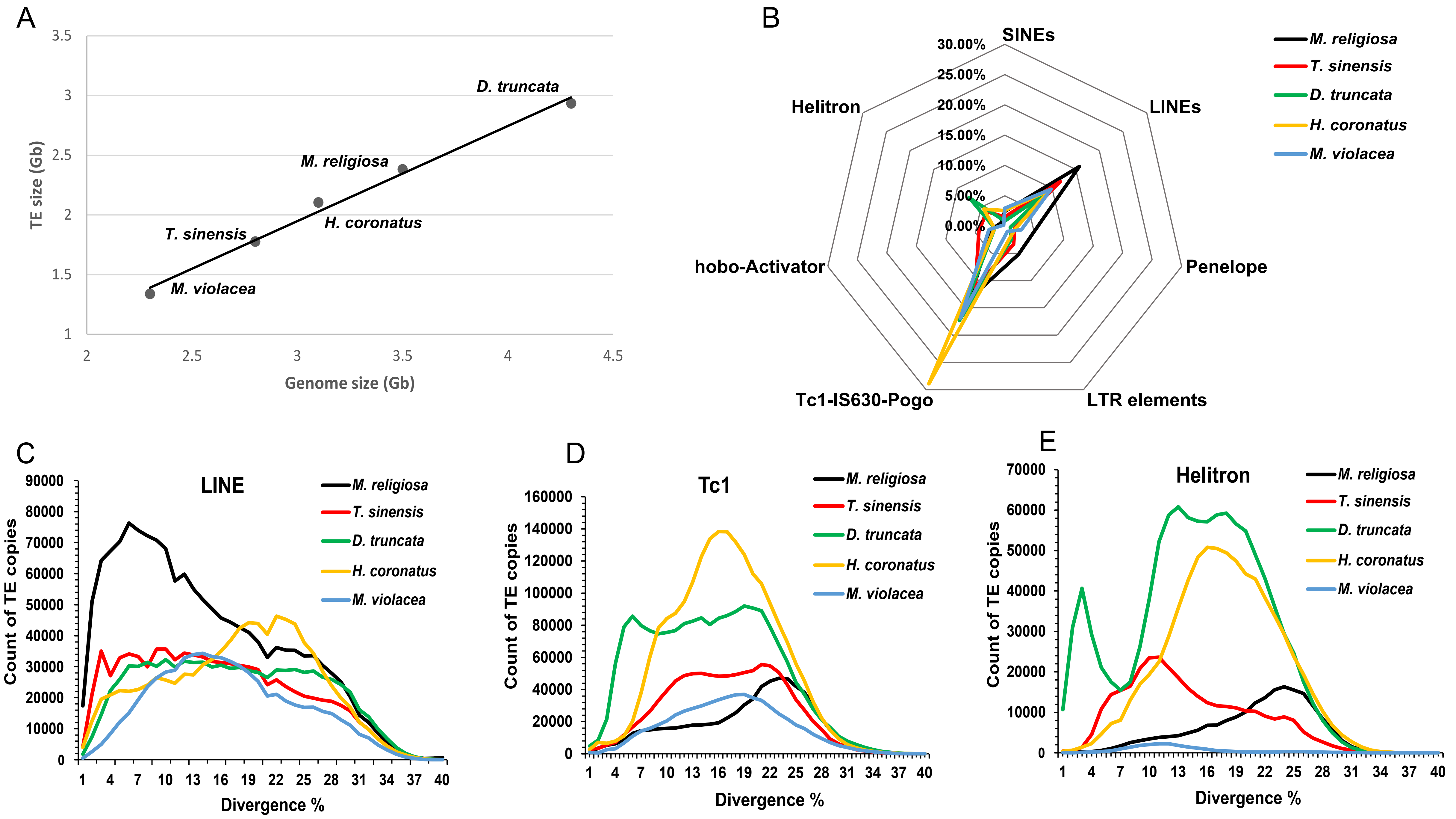

Figure 3

Click here to access/download;Figure;Figure3.pdf

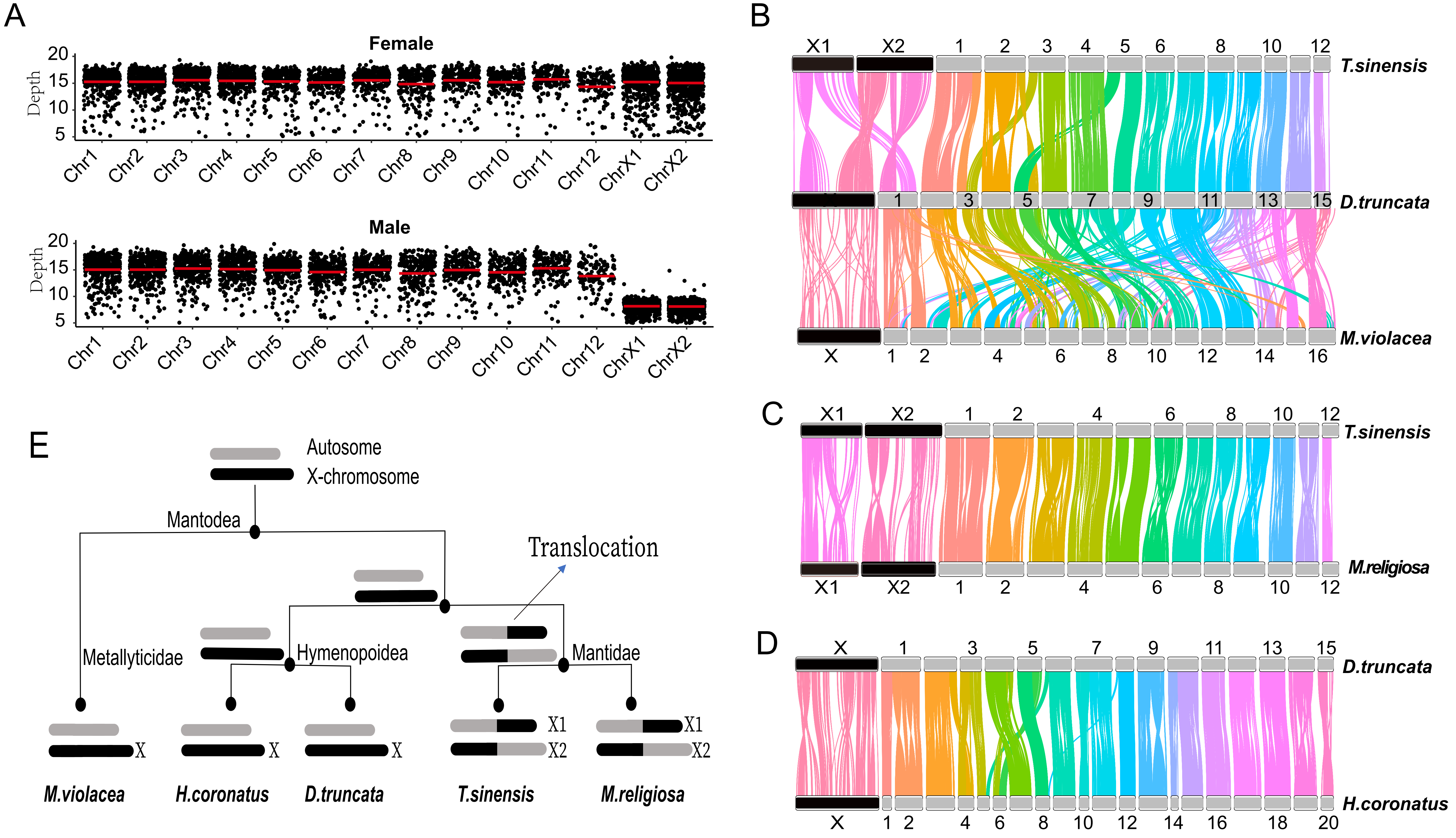

Figure 4 Click here to access/download;Figure;Figure4.pdf

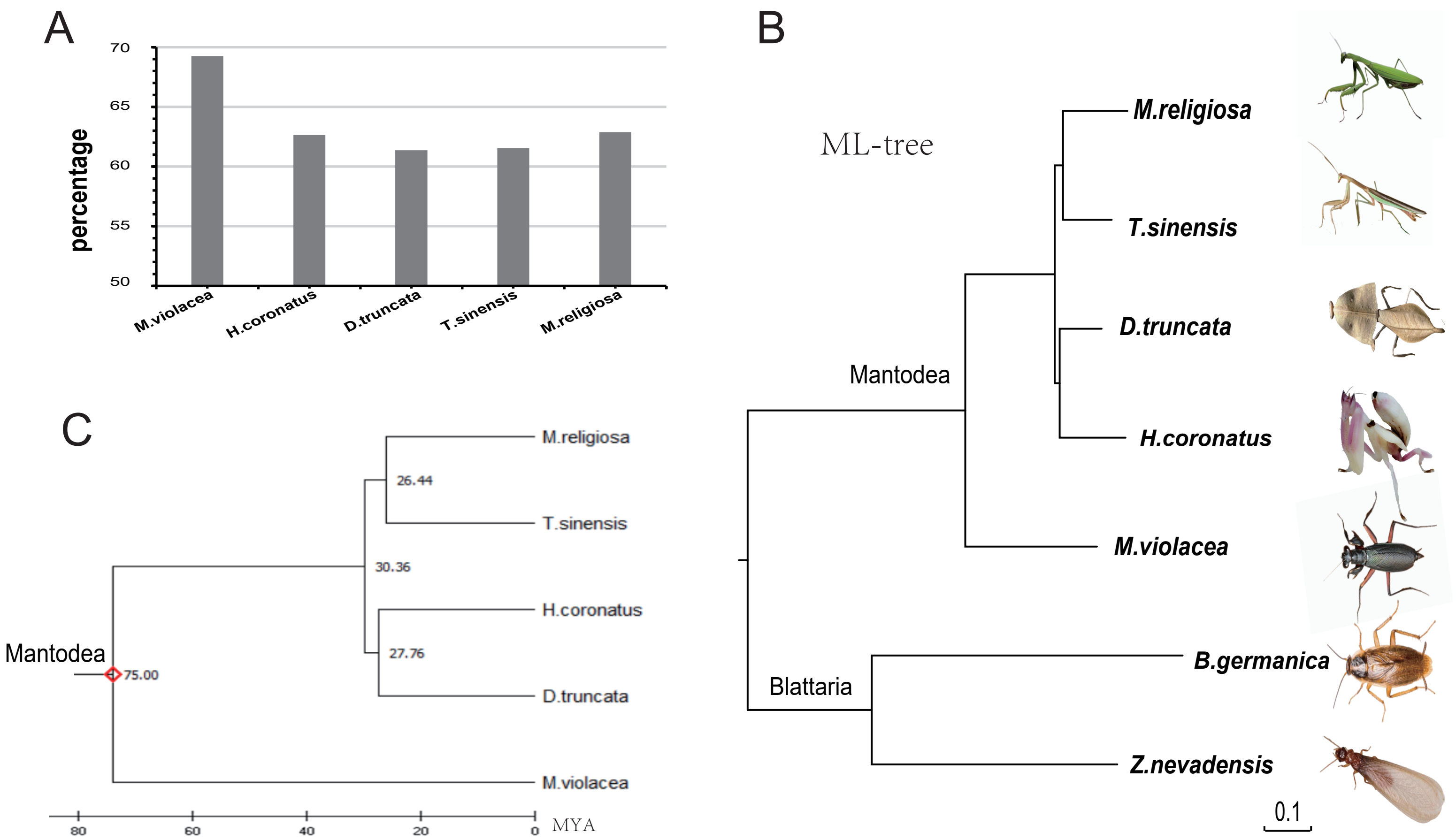

Click here to access/download
Supplementary Material
Supplemental materials - R1.docx
