# Supplementary material for: The genomes of 5 mantises provide insights into sex chromosome evolution and Mantodea phylogeny clarification
Source: Gigascience. 2025 Dec 18;15:giaf158. doi: 10.1093/gigascience/giaf158 (PMC12908712; doi:10.1093/gigascience/giaf158)
Supplement: giaf158_GIGA-D-25-00308_Revision_2 [file giaf158_giga-d-25-00308_revision_2.pdf]

GigaScience

The genomes of five mantises provide insights into sex chromosome evolution and Mantodea phylogeny clarification

--Manuscript Draft--

**Manuscript Number:**	GIGA-D-25-00308R2
**Full Title:**	The genomes of five mantises provide insights into sex chromosome evolution and Mantodea phylogeny clarification
**Article Type:**	Research
**Funding Information:**	Shenzhen Science and Technology Program (KQTD20180411143628272) — Professor Guirong Wang<br>Fund of Key Laboratory of Shenzhen (ZDSYS20141118170111640) — Dr Wei Fan
**Abstract:**	Background Praying mantises, members of the order Mantodea, play important roles in agriculture, medicine, bionics, and entertainment. However, the scarcity of genomic resources has hindered extensive studies on mantis evolution and behaviour.<br>Results Here, we present the chromosome-scale reference genomes of five mantis species: the European mantis (Mantis religiosa), Chinese mantis (Tenodera sinensis), triangle dead leaf mantis (Deroplatys truncata), orchid mantis (Hymenopus coronatus), and metallic mantis (Metallyticus violacea). The assembled genome sizes range ~2.3-4.2 Gb, with contig N50 size 1-109 Mb and 85-99% of sequence anchored to chromosomes. The annotated protein-coding gene number ranges 17,804-19,017, with BUSCO complete rate 96.7-98.4%. We found that transposable element expansion is the major force governing genome size in Mantodea, and suggest that translocations between the X chromosome and an autosome have occurred in the lineage of the family Mantidae. In addition, we found a much lower substitution rate for the lineage of M. violacea than the lineages of other mantises. Furthermore, our genome-wide analyses showed that D. truncata is sister to H. coronatus than M. religiosa and T. sinensis, helps resolve the phylogenic controversies of Deroplatys genus.<br>Conclusions The high-quality genome assemblies of the five mantises provide a valuable resource for evolution studies of Mantodea and genetic improvement and breeding of beneficial biological control agents.
**Corresponding Author:**	Wei Fan<br>Chinese Academy of Agricultural Sciences<br>shenzhen, guangdong CHINA
**Corresponding Author Secondary Information:**	
**Corresponding Author's Institution:**	Chinese Academy of Agricultural Sciences
**Corresponding Author's Secondary Institution:**	
**First Author:**	Wei Fan
**First Author Secondary Information:**	
**Order of Authors:**	Wei Fan<br>Guirong Wang<br>Hangwei Liu<br>Fan Jiang<br>Hengchao Wang<br>Bo Zhang<br>Yutong Zhang<br>Hanbo Zhao

Powered by Editorial Manager® and ProduXion Manager® from Aries Systems Corporation

Order of Authors Secondary Information:	
Response to Reviewers:	Reviewer reports:<br><br>Reviewer #1: Thank you for your efforts in addressing the comments and suggestions from the first round of review. I have carefully reviewed the revised manuscript and am pleased with the revisions made.<br><br>Reply: Thanks.<br><br>Reviewer #2: I thank the authors for addressing some of my previous comments. However, a number of issues remain as discussed below.<br><br>Major comments:<br>There is no reason why a slow rate of substitution should partly explain the morphological resemblance of M. violacea to cockroaches as substitution rates are independent of morphological evolution. Therefore, this interpretation should be removed throughout e.g. "Low evolutionary rate may allow M. violacea conserve more ancestral traits" (line 228) and "the mutation rate of M. violacea genome is the lowest among all the mantises, consistent with its retention of ancestral traits" (line 288)<br><br>Reply: According to the reviewer's suggestion, we removed our conclusion on substitution rate and morphological evolution. The subtitle "Low evolutionary rate may allow M. violacea conserve more ancestral traits" was changed into "Mantodea phylogeny provide taxonomic evidences for D. truncata". The sentence "With a slower evolution rate, M. violacea may preserve more characteristics of the Mantodea ancestor, which also partly explains its morphological resemblance to cockroaches." was removed. The discussion "and the mutation rate of M. violacea genome is the lowest among all the mantises, consistent with its retention of ancestral traits" was also removed.<br><br><br>The comment "Generally, the branch length is in proportional to the amino-acid substitution rate." is also incorrect. Branch lengths in an undated tree are the number of substitutions per site in the sequence alignment. The number of substitutions is determined by the substitution rate and time since divergence. A long branch can be due to a low substitution rate but long divergence time and vice versa. In a dated phylogeny, branch lengths show the amount of time. Moreover, the authors also still do not explain how substitution rates were inferred. I am also not sure if substitution rates can be reliably inferred from a tree which is dated with MEGA as I do not think this method takes into account rate heterogeneity. An explanation of how substitution rates were inferred should be added, if this analysis is kept.<br><br>Reply: We agree with the reviewer. The old description "Generally, the branch length is in proportional to the amino-acid substitution rate. The amino-acid substitution rate for M. violacea branch was the lowest among those of Mantodea (Figure 4B), which may indicate that the evolution rate of M. violacea branch was much slower than that of the other mantises." was revised into "From the phylogenetic tree, we observed that the branch length from the Mantodea node to the M. violacea node is much shorter than those from the Mantodea node to the species nodes of other mantises (Figure 4B). Branch length in an undated tree represents the number of substitutions per site in the sequence alignment. Thus, the shorter branch length of M. violacea means fewer substitutions accumulated from Mantodea ancestor to M. violacea.".<br><br>While the authors now mention the method of dating in the Figure 4 legend, the dating method is not included in the methods section. This should be added.<br><br>Reply: The dating method was added in Methods section: "The time tree was inferred using the Reltime-Branch lengths method in MEGA, with input of user-supplied branch lengths derived from Maximum Likelihood (ML) method. The time tree was computed using 1 calibration constraint (70-80 Mya between M.violacea and M.religiosa, doi: 10.1126/science.1257570)."<br>To be not duplicated, the legend of Figure 4 was shortened into "(C) Time tree inferred by the Reltime-Branch lengths method in MEGA, with 1 calibration constraint (70-80 Mya between M.violacea and M.religiosa)."

Minor comments:

Thank the authors for removing the mention of sex determination in the title. The new title ("The genomes of five mantises provide insights into evolution of sex chromosome and Mantodea lineages") should be made more specific as "provide insights into Mantodea lineages" is vague. As currently written, 'sex chromosome' should be plural.

Reply: “evolution of sex chromosome and Mantodea lineages” was revised into “sex chromosome evolution and Mantodea phylogeny clarification”.

The explanation of the results and questions raised by phylogenetic studies could still be improved. As it reads, it is very brief and could be expanded to set up the questions explored in the paper more. Please also expand the explanation of why studying mantis sex chromosomes is important.

Reply: Thanks for the reviewer’s kind expectation, but it is out of our interest and ability to perform more deeper phylogenetic analysis in this study. According to the reviewer’s suggestion, we added a sentence in the maintext “Studying mantis sex chromosomes will uncover the formation mechanism underling the X1X2Y type.”.

Please also change "most manties have the common XY sex determination system" to "most manties have XY sex chromosomes" (line 59) as the observation of sex chromosomes alone does not indicate the sex determination mechanism.

Reply: Corrected.

If I understand the responses correctly, the photos in Figure 1 correspond to the specimens that were sequenced. If this is the case, then it should be explained in the legend of the figure that the images are of the sequenced specimens.

Reply: We added a sentence “The images of the sequenced specimens were shown in the center of circos plots.” in the legend of Figure 1.

I thank the authors for adding some detail for how divergence times were estimated. However, there is no explanation for why the calibration constraint of 70-80 mya between M.violacea and M.religiosa was chosen. This needs justification.

Reply: The calibration data for Mantodea is quite few. We got the calibration between M.violacea and M.religiosa from a recently published paper in Science: Phylogenomics resolves the timing and pattern of insect evolution (doi: 10.1126/science.1257570). This study inferred the phylogeny and dating time for major insect lineages from 1478 protein-coding genes, which were constructed from transcriptome data. We thought this is the most accurate estimation of dating time between M.violacea and M.religiosa in the world till now, so we chose it.

The authors now note in the discussion that taxon sampling was a major limitation in the phylogenomic analyses. It should also be highlighted that other phylogenomic approaches should also be used, including coalescent based approaches that are better suited for exploring lineages that quickly diversified than concatenation which was used here.

Reply: In this study, we have adopted a traditionally widely-adopted concatenation method for phylogeny construction. We added a discussion in the maintext: “In addition, more advanced phylogenomic methods such as coalescent based approaches may improve the accuracy of phylogenic inference for lineages that quickly diversified.”

I thank the authors for explaining why they used EndHiC instead of YaHS for some assemblies. This explanation should be added to the methods to make this clear to readers.

Powered by Editorial Manager® and ProduXion Manager® from Aries Systems Corporation

	Reply: We added a sentence in Methods: “YaHS is capable to construct chromosome-level scaffolds with relatively shorter contigs, while EndHiC is more suitable for relatively larger contigs.”.<br><br>As the genome statistics which are given in long lists are in Table 1 there is no reason for these to be repeated in full in the main text. Please do use ranges and refer to the table rather than reporting the information for each species. This makes the text unnecessarily verbose and harder to read.<br><br>Reply: Corrected.<br><br>The explanation of why the genomes of H. coronatus differ between studies is still unclear. Please could the authors clarify if they manually curated the X chromosomes and add whether they did this or not to the methods. Documenting manual curation is extremely important for reproducibility. If manual curation was performed, the total number of manual breaks, joins and removals of sequence should be stated. Please also mention in the main text whether the difference in assembly in the X chromosome was due to the use of curation, or if not why a single fragment was produced in the current version while previously it was in 3 fragments.<br><br>Reply: There is no manual curation in our HiC scaffolding, so we did not mention it in the Methods. The reasons may be: we used Hifi sequencing, thus our contig N50 length is much longer, and we used EndHiC, which is more powerful in building chromosome-level scaffolds than other software.<br>We added an explanation sentence in the maintext: “With much longer assembled contigs and EndHiC that is specially designed for scaffolding large contigs, we achieved a complete assembly of the X chromosome for H. coronatus.”.<br><br>I thank the author for adding the correlation of genome size and repeat content to the main text. However, it is essential that the statistical test takes into account phylogenetic non-independence between taxa. Please use a statistical approach that does this e.g. phylogenetic generalised least squares regression.<br><br>Reply: According to the reviewer’s suggestion, we changed to use phylogenetic generalised least squares regression. We added the result in Figure 2A legend: “Phylogenetic Generalised Least Squares Regression (PGLS) was applied, resulting in R=0.954 and p = 0.008 < 0.01”.<br><br>I thank the authors for adding in the example I gave of B chromosomes, however how the sentence is currently written gives the impression that the list is exhaustive. Please either add other ways in which sex chromosomes evolve or else make it clear that just a couple of examples are listed.<br><br>Reply: The sentence “Sex chromosomes evolved from autosomes or B chromosomes” was revised into “Sex chromosomes generally evolved from autosomes and occasionally from B chromosomes”.<br><br>"The cockroach-like body morphology was thought to be the ancestral traits of Mantodea<br>(order)." (line 241). This should be supported by a reference(s).<br><br>Reply: We added a reference paper: “Hornig MK, Haug JT and Haug C. An exceptionally preserved 110 million years old praying mantis provides new insights into the predatory behaviour of early mantodeans. PeerJ. 2017;5:e3605. doi:10.7717/peerj.3605.”.<br><br>The description of the divergence time estimation (line 255) should come at the start of the paragraph rather than the end as this is what allowed rates to be examined.<br><br>Reply: We have removed the analysis of substitution rate, so it does not need to change the placement for divergence time estimation.

"We used HiFi long reads with over 99% accuracy, while they used common long reads with only ~85% Accuracy."." - please could you explain what "common long reads" are. PacBio HiFi sequencing is a frequently used long-read sequencing method so I'm not sure what the authors are referring to. To avoid ambiguity, please be specific. I am also unsure why technology itself makes a big difference - instead it's likely due to differences in average read length, is this the case?

Reply: PacBio sequencing can generate one-pass reads with 85% base accuracy, and multi-pass reads with 99% base accuracy (HiFi reads). Multi-pass means sequencing a DNA fragment several times, and finally call a consensus from a multiple-sequence alignment. The read length for one-pass and multiple-pass has no difference. To be specific, we changed “common long reads” into “one-pass long reads”.

The sentence on why Mantodea occupies "an important position in the evolution of insects" (line 280) still needs to be clearer. Please elaborate.

Reply: This sentence was revised into “Mantodea has occupied an important position in the evolution of insects, with distinctive morphology, camouflage behavior, and unusual sex determination mechanism.”.

This needs further detail e.g. is 4,014 referring to the number of single-copy OGs in all mantises and outgroups? "From gene family clustering, 69,603 orthologous groups (OGs) were generated, including 4,014 single-copy OGs."

Reply: Yes. The orthogroups were constructed using genes from all the mantises. Each orthogroup contains genes of all mantises derived from a single gene in their latest common ancestor. Single-copy OG means that the OG contain only one gene for each mantis.

The sentence was revised into “From gene family clustering, 69,603 orthologous groups (OGs) were generated for all the analyzed species, including 4,014 single-copy OGs in which each mantis must be present and have only a single gene.”.

The description of the radar chart in figure 2A is still insufficient. Please explicitly state what a higher vertex means and the corresponding percentages - e.g. are these the percentage of genome occupied by TEs, or total percentage of TEs corresponding to a particular TE type?

Reply: They are the percentage of genome occupied by TEs. We have added a sentence in Figure 2A legend: “The percentage for each height level of the vertex represents the percentage of genome occupied by TEs.”.

Figure 3B: The colours are still not correctly tracking between the chromosomes of each species. E.g. chr 1 of T. sinensis is pink to me while the homologous chromosomes in D. truncata is orange. This should be fixed. The legend describes "grep color". I'm not familiar with what this is.

Reply: We’d like to explain this misunderstanding. The color for synteny links between each homologous chromosome in different species were automatically determined by the MCScanX software. The color was randomly assigned, just to be different among various homologous chromosomes, so the users can identify the synteny blocks easily. It was difficult for us to modify the color for each syntenic link.
We used black box to represent sex chromosome, and use gray box to represent autosome chromosome. The sentence “For B-E, black color represents for X chromosomes, while gray color represents for autosomes” was revised into “For subfigures B-E, black box represents for X chromosomes, while gray box represents for autosomes.”.

Powered by Editorial Manager® and ProduXion Manager® from Aries Systems Corporation

The legend also has an unclear sentence "Much more chromosome rearrangements were observed between M. violacea and the other mantises". Please revise this to make the message clearer.

Reply: This sentence was revised into “Much more chromosome rearrangements were observed between M. violacea and D. truncata, in comparison to those between T. sinensis and D. truncata.”.

Figure 4: The y-axis is just 'percentage'. Please elaborate what the percentage is of. The figure title for Figure 4 "Evolution of Mantodea lineages" is very vague, please make it specific such that it summarises the key message of the figure.

Reply: “percentage” was revised into “Percent of OG”, and "Evolution of Mantodea lineages” was revised into “Orthologous groups and phylogeny of Mantodea”.

With regards to adding the identified sex chromosomes and the sex that the genome is derived from to Table 1. I understand that the authors do not add more rows to Table 1. suggest instead adding this information within the existing rows e.g. "# of assembly-inferred chromosomes" could be 13A + 1Z rather than 14, and adding the sex sequenced in brackets next to the species name in the header row, thus conveying this information without adding more rows.

Reply: Thanks for the reviewer’s suggestion. We have changed the chromosome number into “12+X1+X212+X1+X215+X20+X16+X” in Table 1, and added a note under this table : “# of assembly-inferred chromosomes” refers to autosome and sex chromosome number in female.

Figure S11: "The topology of NJ phylogeny tree is largely consistent the ML (maximum likelihood) tree in Figure 4B.". The topology appears the same to me between these two figures. Suggest removing this figure and just saying that the same topology was obtained by NJ in the main text as the result is the same.

Reply: According to the reviewer’ suggestion, Figure S11 was removed. In addition, we added a sentence in the maintext: “and the same topology was obtained by NJ method,”.

Typos and suggested rephrasing:

Line 152: I do not think it's typical to put the taxonomic levels in brackets next to each taxon. Perhaps rephrase this e.g. "Currently the orders of Mantodea and Blattodea are placed within the superorder Dictyoptera"

Reply: Corrected.

Line 122: typo - "manily" → "mainly"

Reply: Corrected.

"using reciprocal-best orthologous genes" - should this be "reciprocal best hit". Please change in all instances.

Reply: We have changed all “reciprocal-best orthologous genes” into “reciprocal best hits”.

Line 500: Suggest changing "4 chromosome brokens" to "4 chromosome breaks"

Reply: Corrected.

	Ideally a single textface should be used in figures. Some figures use multiple text faces.<br><br>Reply: We have revised the textface in all figures to make them consistent.
**Additional Information:**	
**Question**	**Response**
Are you submitting this manuscript to a special series or article collection?	No
**Experimental design and statistics**<br><br>Full details of the experimental design and statistical methods used should be given in the Methods section, as detailed in our Minimum Standards Reporting Checklist. Information essential to interpreting the data presented should be made available in the figure legends.<br><br>Have you included all the information requested in your manuscript?	Yes
**Resources**<br><br>A description of all resources used, including antibodies, cell lines, animals and software tools, with enough information to allow them to be uniquely identified, should be included in the Methods section. Authors are strongly encouraged to cite Research Resource Identifiers (RRIDs) for antibodies, model organisms and tools, where possible.<br><br>Have you included the information requested as detailed in our Minimum Standards Reporting Checklist?	Yes
**Availability of data and materials**<br><br>All datasets and code on which the conclusions of the paper rely must be either included in your submission or deposited in publicly available repositories (where available and ethically appropriate), referencing such data using	Yes

a unique identifier in the references and in the “Availability of Data and Materials” section of your manuscript.<br><br>Have you have met the above requirement as detailed in our Minimum Standards Reporting Checklist?	
GigaScience has policies and guidelines in place for the use of generative AI-writing tools such as ChatGPT. If you have used such writing tools to assist with writing the manuscript this must be declared and cited in the text. Authors should not list AI-writing tools and other AI-assisted technologies as an author or co-author and should acknowledge that they are fully responsible for text generated or refined by AI-writing tools.<p><br><br>A summary of use (particularly in the introduction or among methods) needs to be included at the end of the paper, and the outputs should also be included as a supplementary file hosted in GigaDB or other open repositories. Please read our guidelines for more information. <p><br><br>By submitting to GigaScience, you are aware of the journal's AI-writing tools policy, and if you have declared use of such tools below, you have acknowledged this where appropriate in your manuscript and have made a summary of use and outputs available. <p><br><b>AI-assisted writing tools have been used in the preparation of this manuscript?	No

Manuscript

Click here to access/download;Manuscript;Manuscript - R2 - final 1216.docx

The genomes of five mantises provide insights into sex chromosome evolution and Mantodea phylogeny clarification

Hangwei Liu[1,2,†], Lihong Lei[1,3,4,†], Fan Jiang[1], Bo Zhang[1], Hengchao Wang[1], Yutong Zhang[3], Hanbo Zhao[1], Guirong Wang[1,5,*] & Wei Fan[1,*]

[1] Guangdong Laboratory for Lingnan Modern Agriculture (Shenzhen Branch), Genome Analysis Laboratory of the Ministry of Agriculture and Rural Affairs, Agricultural Genomics Institute at Shenzhen, Chinese Academy of Agricultural Sciences, Shenzhen, Guangdong, 518120, China.

[2] College of Plant Protection, Yangzhou University, Yangzhou 225009, China

[3] School of Life Sciences, Henan University, Kaifeng 475004, China

[4] Shenzhen Research Institute of Henan University, Shenzhen 518000, China

[5] State Key Laboratory for Biology of Plant Diseases and Insect Pests, Institute of Plant Protection, Chinese Academy of Agricultural Sciences, Beijing, China.

†These authors contributed equally to this work.

* Correspondence should be addressed to wangguirong@caas.cn and fanwei@caas.cn.

Hangwei Liu [0000-0002-4931-1307]; Lihong Lei [0000-0002-2524-7523]; Fan Jiang [0000-0003-1359-0970]; Bo Zhang [0009-0004-1350-0087]; Hengchao Wang [0000-0002-8754-4195]; Yutong Zhang [0009-0009-5053-6165]; Hanbo Zhao [0000-0001-9371-777X]; Guirong Wang [0000-0002-9391-931X]; Wei Fan [0000-0001-5036-8733]

Abstract

Background Praying mantises, members of the order Mantodea, play important roles

in agriculture, medicine, bionics, and entertainment. However, the scarcity of genomic resources has hindered extensive studies on mantis evolution and behaviour.

Results Here, we present the chromosome-scale reference genomes of five mantis species: the European mantis (*Mantis religiosa*), Chinese mantis (*Tenodera sinensis*), triangle dead leaf mantis (*Deroplatys truncata*), orchid mantis (*Hymenopus coronatus*), and metallic mantis (*Metallyticus violacea*). The assembled genome sizes range ~2.3-4.2 Gb, with contig N50 size 1-109 Mb and 85-99% of sequence anchored to chromosomes. The annotated protein-coding gene number ranges 17,804-19,017, with BUSCO complete rate 96.7-98.4%. We found that transposable element expansion is the major force governing genome size in Mantodea, and suggest that translocations between the X chromosome and an autosome have occurred in the lineage of the family Mantidae. In addition, we found the lineage of *M. violacea* has accumulated fewer substitutions than the lineages of other mantises. Furthermore, our genome-wide analyses showed that *D. truncata* is sister to *H. coronatus* than *M. religiosa* and *T. sinensis*, helps resolve the phylogenic controversies of *Deroplatys* genus.

Conclusions The high-quality genome assemblies of the five mantises provide a valuable resource for evolution studies of Mantodea and genetic improvement and breeding of beneficial biological control agents.

Keywords: Mantodea, genome, transposable element, X1X2Y, evolution

Background

Praying mantises are familiar insects that play important roles in agriculture, medicine, and bionics. As predators of many harmful insect species, praying mantises such as the European mantis (*Mantis religiosa*, NCBI:txid7507) and Chinese mantis (*Tenodera sinensis*, NCBI:txid406589) are widely acknowledged as natural enemies that control plant pests[1], benefiting organic planting where pesticide is prohibited. The mantis ootheca (egg capsule, egg chamber) is a traditional medicine used to cure frequent micturition, strengthen kidney health and prevent spermatorrhea in East Asian countries [2]. Most praying mantises have two sharp and strong forelegs, which are much larger

and more powerful than their ancient ancestors. In addition, the femur and tibia of the forelegs are armed with strong spines along their posterior edges. When the femur and tibia fold on each other, a praying mantis can firmly grasp the prey. This distinctive body structure of the praying mantis has been a significant source of inspiration in bionics of cutting blades [3, 4]. Although most manties have XY sex chromosomes, some mantises such as *M. religiosa* and *T. sinensis* have the X1X2Y type [5-7], making them a special material for studying the evolution of sex chromosomes.

The two closely related orders, Mantodea (mantises) and Blattodea (cockroaches and termites), are classified into the superorder Dictyoptera, and phylogenomic analyses revealed that Mantodea split from Blattodea during the Permian [8]. Mantodea has evolved into a group comprising ~2500 species with diverse morphological and ecological characteristics, with the highest diversity in tropical and subtropical habitats [9, 10]. From fossils of early Mantodea and Blattodea species, the common ancestor is thought to resemble modern cockroaches in many aspects [11]. The metallic mantis (*Metallyticus violacea*, NCBI:txid406581), in the early diverging mantid lineage, has many morphological features similar to those of modern cockroaches, but the underlying mechanisms have not been discovered [12]. Although Mantodea are well supported as monophyletic, the phylogenetic relationships within Mantodea are still not well resolved. For example, *Deroplatys* genus has been placed into Mantidae family traditionally, but these species are more similar to species in Hymenopodidae family in many aspects such as morphology and camouflage [9, 13]. Clarifying the relationships within Mantodea will greatly benefit the functional studies as well as genetic breeding of mantises.

Compared with those of many other insect orders, the genomic resources of Mantodea are very limited, with only three chromosome-scale reference genomes available: the Chinese mantis (*T. sinensis*, NCBI:txid406589), orchid mantis (*H. coronatus*, NCBI:txid267205) and Malaysian dead leaf mantis (*Deroplatys lobata*, NCBI:txid1661821) [14, 15]. Here, we present the chromosome-scale reference

genomes of three other mantis species, the European mantis (*M. religiosa*), triangle dead leaf mantis (*D. truncata*), and metallic mantis (*M. violacea*), as well as a more complete assembly of *T. sinensis* and *H. coronatus*, to promote the evolutionary and biological studies of Mantodea.

Results

Chromosome-scale genome assemblies of five mantis species

We generated 26-63 X PacBio HiFi data, and 35-79 X Illumina Hi-C data for *M. religiosa*, *T. sinensis*, *D. truncata*, *H. coronatus*, and *M. violacea*, respectively (Table S1, S2). The PacBio HiFi reads were used to assemble the contig sequences, with a total size of 2.3-4.2 Gb and N50 sizes of 1-109 Mb. The Illumina Hi-C reads were mapped to the contig sequences, and the valid Hi-C read pairs were used for scaffolding assembly (Table S3), resulting in 85.39-98.51% of the contig sequences anchored into 14-21 chromosome-level scaffolds (Figure 1A-E, S1, S2; Table 1, S4). Notably, only the chromosome numbers for *M. religiosa* and *T. sinensis* have been karyotyped [16, 17], whereas the others are inferred only from the genome assembly. Using the GCE method that estimates genome size with K-mer frequency distribution from sequencing reads [18], the estimated genome sizes are 3.5 Gb (*M. religiosa*), 2.8 Gb (*T. sinensis*), 4.3 Gb (*D. truncata*), 3.1 Gb (*H. coronatus*), and 2.3 Gb (*M. violacea*), consistent with assembled genome sizes. Based on GCE heterozygosity model [18], the estimated heterozygous rates are approaching 2% for *M. religiosa* and *T. sinensis*, whereas about 0.05-1% for the other three species. Owing to the higher heterozygosity rate (Figure S3), the contig sizes for the *M. religiosa* and *T. sinensis* are shorter than those for the other three species.

Recently, Huang et al. published a reference genome for *H. coronatus*, with a much shorter contig N50 size of 15.7 Mb [14], and Yuan et al. published a reference genome of *T. sinensis* with a contig N50 size of 2.36 Mb, which is also much shorter than that of this study [15]. The difference in assembly continuity is mainly caused by the applied sequencing technologies. We used HiFi long reads with over 99% accuracy, while they

used one-pass long reads with only ~85% accuracy. From syntenic alignments of the two assemblies for *H. coronatus*, we found that most chromosomes were largely consistent except for the X chromosome (Figure S4). One complete X chromosome in our assembly corresponds to 3 fragmented chromosomes in Huang's assembly. The X chromosome is the largest chromosome, making it more difficult to assemble than the autosomes. With much longer assembled contigs and EndHiC that is specially designed for scaffolding large contigs, we achieved a complete assembly of the X chromosome for *H. coronatus*. We also compared another reference genome published by the Huang group [14], the Malaysian dead leaf mantis (*Deroplatys lobata*, CRA010804 in the National Genomics Data Center, China), to our assembled reference genome of *D. truncata* (Figure S5). Belonging to the same genus, most chromosomes have high synteny, except for four chromosomes involved in chromosome-level rearrangements, which are more likely due to species divergence than assembly errors. Both reference genomes of *T. sinensis* (Yuan et al.[15] and the present study) showed high synteny for all chromosomes (Figure S6).

By integrating homology and transcription evidence, 17,804-19,017 protein-coding gene models were annotated as the reference genes for each mantis (Table 1, Table S5). The BUSCO complete rates for the reference genes of these mantis species range from 96.7%-98.4% (Figure 1F), which are higher than or comparable to those of previously published mantis genomes [14] [15]. Furthermore, 97.2%-98.6% of the reference genes in these five mantis species were assigned functions according to at least one of the NCBI-NR, KEGG, InterPro or GO databases.

Distinct TE expansions in various mantid lineages

Increasing evidence has shown that transposable elements (TEs) contribute significantly to the genome size and influence the genome architecture, along with insertions, deletions, translocations, etc [19]. We analyzed the total TE content (ratio) among the 5 species and found that genome size was linearly correlated with TE abundance (Figure 2A, S7). Currently the orders of Mantodea and Blattodea are placed within the superorder Dictyoptera. Mantidae is a representative mantis family, which

belongs to Mantoidea (superfamily) in Mantodea. The two Mantidae species (*M. religiosa* and *T. sinensis*) have relatively smaller genome sizes (2.3-2.8 Gb) and lower TE contents (58-63%), than the other 3 mantises, with relatively larger genome sizes (3.1-3.5 Gb) and higher TE contents (67-68%), suggesting that genome size differences are mostly determined by TE contents in mantids.

Abundant retrotransposons, DNA transposons and rolling-circle transposons were found in these mantis genomes, however, their ratios in genome differ across species (Figure 2B, Table S6, S7). For the two Mantidae species, LINEs are the largest components, and a sharp expansion of LINEs with divergence of approx. 7% was found in *M. religiosa* (Figure 2C). However, no recent large-scale expansion of LINEs has occurred in *T. sinensis*, which may explain why its genome size (2.8 Gb) is smaller than that of *M. religiosa* (3.5 Gb). In contrast, *D. truncata* and *H. coronatus* have massive DNA transposons, with Tc1 (especially Tc1-IS630-Pogo) being the largest component in these two species, consistent with the findings of a former study [14]. *D. truncata* has undergone both a recent sharp expansion and an ancient burst of Tc1 in its genome, leading to the largest genome size (4.3 Gb) found in this study, whereas only an ancient explosion of Tc1 was observed in *H. coronatus* (Figure 2D). Both *D. truncata* and *H. coronatus* also have a large rolling-circle transposon, *Helitrons*. Both a recent and an ancient burst of *Helitron* were observed in *D. truncata*, whereas only an ancient burst of *Helitron* was found in *H. coronatus* (Figure 2E). *M. violacea* shows no recent accumulation of any category of TEs, which may explain why its genome size (2.3 Gb) was the smallest among these mantises.

These results collectively suggest that TE expansion is the major force behind genome size variation in Mantodea. In addition, the components and divergence times of the various TE types are distinct among the different mantid lineages.

Translocation between X chromosome and autosomes in Mantidae lineage

Sex chromosomes generally evolved from autosomes and occasionally from B chromosomes, and play important roles in tissue development, mating, and speciation [20]. The types of sex chromosomes found in insects vary among species, and sex

chromosome systems exhibit significant diversity across insect species [5-7]. Most insects have XY, ZW or XO sex chromosome systems, but there are other rare sex chromosome types, such as the X1X2Y type, which has two X chromosomes and one Y chromosome. Some hemipterans such as *Philaenus italosignus* [21] and some mantids such as *M. religiosa* [22], exhibit this sex chromosome type. Studying mantis sex chromosomes will uncover the formation mechanism underling the X1X2Y type.

To identify the X chromosomes from the assembled pseudochromosomes, we generated 15X short-read sequencing data for the female and male *T. sinensis* individuals, respectively. Sequencing coverage revealed that all 14 chromosomes in female have comparable coverage depths, whereas in male, two chromosomes have approximately half the coverage depth (Figure 3A). It has been reported that most members of the family Mantidae have two X chromosomes, X1 and X2, derived from fusion or translocation between the X chromosome and an autosome [16]. In addition, the genome assembly of *T. sinensis* was derived from a female individual, which lacked the Y chromosome. Thus, the two chromosomes with half coverage depths are concluded to be the X1 and X2 chromosomes. Notably, they are the largest and second largest of our assembled pseudochromosomes, consistent with previous reports based on karyotyping [16, 23].

Macroscale synteny analysis identified the corresponding X chromosomes in the other 4 species, and allowed comparative analysis among the mantid X chromosomes. Synteny alignments revealed that both Mantidae species *M. religiosa* and *T. sinensis* have two sex chromosomes, X1 and X2; however, the other species have only one sex chromosome X. In addition, only part of X1 (X1L) and X2 (X2L) in Mantidae were aligned with the X chromosomes of the other 3 species (Figure 3B-D, S8). These results suggest that the ancestral mantid had one X chromosome and that the translocation of large fragments between the X chromosome and an autosome occurred in Mantidae (Figure 3E). Previous studies have revealed that the common ancestor of Dictyoptera had a XY sex chromosome system [16, 23] (Figure S9). We inferred that the common ancestor of the Mantidae family evolved the X1X2Y sex chromosome system, and our results support a model in which the generation of the X1 and X2 chromosomes resulted

from the translocation between one X chromosome and an autosome.

Furthermore, based on macroscale synteny analysis, we were able to identify the breakpoints as a site falling within a 6.65-Mb region on the X1 chromosome and a site falling within 2.56-Mb region on the X2 chromosome of *T. sinensis*. Inside these two regions, transposon and tandem repeats dominate the sequence (Figure S10), posing great difficulties for accurate genome assembly and inter-species genomic sequence alignment. In future, as the assembly continuity improves, it is possible to narrow down the breakpoint range, which will approach or surpass the resolution of traditional cytological technologies such as C-banding, silver staining and living-cell images of the meiosis process [16, 23].

Mantodea phylogeny provide taxonomic evidences for *D. truncata*

Comparative analysis of Mantodea genomes within a phylogenetic context is essential for understanding their evolution and diversity. Phylogenomic analyses were performed on these 5 Mantodea species, which span 5 genera and 3 families with diverse habitats and morphologies. Two Blattodea species, the German cockroach (*Blattella germanica*) [24] and the dampwood termite (*Zootermopsis nevadensis*) [25], were used as the outgroup (Table S8). From gene family clustering, 69,603 orthologous groups (OGs) were generated for all the analyzed species, including 4,014 single-copy OGs in which each mantis must be present and have only a single copy.

M. violacea belongs to the superfamily Metallyticoidea, exhibiting significant morphological differences compared to other mantises. *M. violacea* shares many characteristics with its cockroach relatives, including dull body colouration, a prostrate body posture, and a relatively shorter prothorax. The cockroach-like body morphology was thought to be the ancestral traits of Mantodea order [11]. The Metallyticoidea lineage is sister to the other mantis lineages [26]. *M. violacea* shares much more OGs with cockroaches than the other 4 species (69% vs 61-63%) (Figure 4A), which may partly explain its strong morphological resemblance to cockroaches.

The phylogenetic tree was constructed based on 4,014 single-copy OGs, and the divergence time along branches were estimated. From the phylogenetic tree, we

observed that the branch length from the Mantodea node to the *M. violacea* node is much shorter than those from the Mantodea node to the species nodes of other mantises (Figure 4B). Branch length in an undated tree represents the number of substitutions per site in the sequence alignment. Thus, the shorter branch length of *M. violacea* means fewer substitutions accumulated from Mantodea ancestor to *M. violacea*. The divergence time was inferred by the Reltime-Branch lengths method, using one calibration constraint (70-80 Mya between M.violacea and M.religiosa). The results showed that four modern mantises (D.truncata, H.coronatus, M.religiosa, and T.sinensis) emerged within a short time period (26-31 Mya) (Figure 4C), posing difficulties for phylogenic inference within this lineage.

The phylogenetic tree also revealed that *D. truncata* is closer to *H. coronatus* (Hymenopodidae) than *M. religiosa and T. sinensis* (Mantidea) (Figure 4B), and the same topology was obtained by NJ method, differing from the phylogenic assignment from some previous studies that place *D. truncata* within Mantidea [9, 13]. After adding the genomic data for *D. lobata*, both Deroplatys species sistered to *H. coronatus* (Figure S11). Therefore, genome-wide data is helpful to clarify phylogeny controversies, providing important evidences for further species classification of Deroplatys.

Discussion

In this study, we generated chromosome-level genome assemblies for 5 mantis species via a combination of PacBio HiFi and Hi-C sequencing technologies. For *H. coronatus* and *T. sinensis*, both the contig N50 and N90 sizes of our assembly are approximately 5 times greater than those of the previously published reference genomes [14, 15]. In our results, assembly continuity for *M. religiosa* and *T. sinensis* is relatively lower than that for the other 3 mantises due to the differences in heterozygosity, suggesting that high heterozygosity can be problematic for genome assembly. Compared with those of cockroaches and termites, the much larger genome sizes of mantises are mainly the result of expansions of various types of transposable elements.

Mantodea has occupied an important position in the evolution of insects, with

distinctive morphology, camouflage behavior, and unusual sex determination mechanism. One of its major sublineages, the family Mantidae, has a special X1X2Y sex determination system. Through comparative genomics analysis, we inferred that the mantid common ancestor had only one X chromosome and translocation between the X chromosome and an autosome occurred in the ancestor of Mantidae. *M. violacea* genome shares more orthologous genes with cockroaches than with the other mantises. Our phylogenetic analyses with genome-wide data also suggest that the two *Deroplatys* species are closer to *H. coronatus* than to the two Mantidea mantises, which may do some help for further phylogenetic clarification and accurate species classification of *Deroplatys*. Based on very limited taxon sampling, we made a set of preliminary conclusions in this study, which will be verified by future studies as more genomes being sequenced. In addition, more advanced phylogenomic methods such as coalescent based approaches may improve the accuracy of phylogenic inference for lineages that quickly diversified.

Although praying mantises are efficient predators, their hunting objects are not specific to harmful insects, hindering their wide application in organic planting. Thus, the genomic resources generated in this study will also facilitate molecular breeding of the praying mantis, in order to make it a more applicable nature enemy insect.

Methods

Insect collection and sequencing

Mantis adults were collected at different locations: *M. religiosa* and *T. sinensis* from the forest of Guangzhou, China; *H. coronatus* from the rainforest of Xishuangbanna, China; and *D. truncata* and *M. violacea* from two captive breeding centers in Beijing, China. The species were confirmed by morphological characters, and the photos for sequenced individuals were shown in Figure 1. All mantis samples for sequencing had the intestine removed to avoid contamination by bacteria, fungi, and residual prey bodies. All the tissues were cleaned with 30% ethanol and ddH_2O, and then immersed in liquid nitrogen for cryopreservation.

For Pacific Biosciences (PacBio) HiFi sequencing, libraries with ~15 kb insert sizes were constructed from a female adult of every mantis, and sequenced on a PacBio Sequel II system (RRID: SCR_017990). Subreads were generated with an N50 size of 14.5 kb, and consensus reads (CCS reads) were generated via ccs software (v.3.0.0) [27] with the following parameters: -min-passes 0 -min-rq 0.99 -min-length 100 -max-length 50,000. Then, Hi-C data was generated using the same individuals applied for HiFi sequencing. Nuclear DNA was cross-linked by soaking leaf tissues in formaldehyde solution, and the cross-linked genomic DNA was extracted, digested, repaired, ligated to circular fragments, sheared into 350 bp inserts, converted to short-read sequencing library by Truseq DNA Library Prep Kit, and sequenced on Illumina NovaSeq 6000 platform (RRID:SCR_016387). To identify the sex chromosome, short-read sequencing of a male adult and another female adult of *T. sinensis* was performed on Illumina NovaSeq 6000 platform (RRID:SCR_016387), using DNA library with a 400 bp insert size constructed via Truseq DNA Sample Prep Kit (Illumina).

Total RNA from the abdomen, hind leg, middle leg, foreleg, thorax, head, and eye of a female adult for each species were extracted with TRIzol reagent (Invitrogen), and used to construct cDNA libraries with Truseq RNA Sample Prep Kit (illumina). Transcriptome sequencing data were generated via the Illumina NovaSeq 6000 system in PE150 mode.

Genome assembly and quality assessment

K-mer frequencies from HiFi reads of five mantises were calculated via Kmerfreq [18], and then genome sizes were estimated via GCE (GCE, RRID:SCR_017332). The PacBio HiFi reads were assembled into contigs via Hifiasm (v0.14) (Hifiasm, RRID:SCR_021069)[28] with the following parameters: -l 1 -s 0.7. To filter duplicated contigs in the assembly, purge_dups (v1.2.3) (purge dups, RRID:SCR_021173) [27] was adopted with the following parameters: -2 -a 50. The completeness of the assembly was evaluated using BUSCO (v5.2.2) (BUSCO, RRID:SCR_015008) based on the OrthoDB (v10) (OrthoDB, RRID:SCR_011980) Insecta database [29].

For Hi-C scaffolding, two strategies YaHS and EndHiC were applied. YaHS is capable

to construct chromosome-level scaffolds with relatively shorter contigs, while EndHiC is more suitable for relatively larger contigs. For *M. religiosa*, whose assembly was fragmented into more contigs, Hi-C reads were mapped to contigs via the Arima mapping pipeline (ArimaGenomics), and then, YaHS (v1.2a.1) (YaHs, RRID:SCR_0229650) [30] was used to assemble the contigs into pseudo chromosomes. For the other four mantises, whose contigs are much larger, Hi-C reads were mapped to contigs by Bowtie 2 (v 2.2.2.7) (Bowtie 2, RRID:SCR_016368) [31], then HiC-Pro (v2.11.0-beta) (HiC-Pro, RRID:SCR_017643) [32] was adopted to identify valid ligation pairs and generate Hi-C link matrices among different contigs, and finally, the contigs were clustered, ordered, and oriented into pseudo-chromosomes using EndHiC (v1.0) (EndHiC, RRID:SCR_022110) [33] based on the Hi-C linkage information among contig ends.

Genome annotation

A *de novo* transposable element (TE) library was constructed with RepeatModeler (v2.0.2) (RepeatModeler, RRID:SCR_015027) with the parameters -engine ncbi-database [34], and then RepeatMasker (v4.1.0) (RepeatMasker, RRID:SCR_012954) was used to identify TEs in the reference genome, using both the *de novo* TE library and the public Repbase TE library (v26.05) (Repbase, RRID:SCR_021169). The tandem repeat elements in the genome were subsequently identified using Tandem Repeats Finder (TRF) (Tandem Repeats Finder, RRID:SCR_022193) (v4.09) [35].

The protein-coding gene models were annotated in two rounds. In the first round, the genes were predicted by integrating evidence from *de novo* gene predictions and transcriptome-based gene predictions. *De novo* gene prediction was performed on the TE-masked genome assembly with AUGUSTUS (v3.4.0) (Augustus, RRID:SCR_008417) [36]. For transcriptome-based gene prediction, the RNA-seq data were filtered by Fastp (v0.23.1) (fastp, RRID:SCR_016962) [37]and then mapped to the genome using Bowtie2 (v2.2.7) [31], and StringTie (v1.3.3b) (StringTie, RRID:SCR_016323) was then used to construct the gene models [38]. All the gene models obtained via the above two approaches were subsequently integrated with EVidenceModeler (v1.1.1) (EVidenceModeler, RRID:SCR_014659) [39]. In the

second round, for each mantis, the protein sequences from the other 4 sequenced mantises in this study were mapped to this genome assembly with Exonerate (v2.4.0) (Exonerate, RRID:SCR_016088) [40], and incomplete gene models were filtered. Finally, for each mantis, the *de novo* gene predictions, the transcriptome-based gene predictions, and the homology-based gene predictions were integrated with EVidenceModeler (v1.1.1) to generate a high-confidence and nonredundant gene set.

The completeness of the gene sets was assessed using BUSCO based on OrthoDB (v10) for Insecta. For gene functional annotation, the mantis protein sequences were aligned to the KEGG (KEGG, RRID:SCR_012773), eggNOG (eggNOG, RRID:SCR_002456), NR, and UniProt (SwissProt) databases using DIAMOND (v0.9.24.125) (DIAMOND, RRID:SCR_009457) [41], and only the best hits with E-values less than $1e^{-5}$ were retained. Moreover, InterProScan (v5.38) (InterProScan, RRID:SCR_005829) was used to annotate the protein domains and GO (Gene Ontology) terms [42].

X chromosome identification and analysis

To identify the X chromosome of *T. sinensis*, the clean Illumina paired reads from female and male samples were mapped to the genome of *T. sinensis* via BWA (v0.7.17-r1188) (BWA, RRID:SCR_010910) [43]. The bam files were filtered using SAMtools (v1.6) (SAMTOOLS, RRID:SCR_002105) [44] with the parameters '-q 60 -F 1804', and paired reads mapped onto different chromosomes were also filtered. To assess the sequencing depth of each chromosome, SAMtools depth (v1.6) was used to calculate the average base coverage. The two chromosomes in males whose sequencing depth was approximately half that of the other chromosomes, were identified as X-derived chromosomes. For consistency with the karyotype results for *T. sinensis* [22] and *M. religiosa* [16], the larger one was denoted X2, whereas the smaller one was denoted X1.

Pairwise collinearity analyses were conducted using the protein sequences of five mantis species as markers. DIAMOND (v0.9.24.125) with the parameters 'blastp -f 6' was used to align the protein sequences of each species pair, and the reciprocal best pairs were used as inputs for MCScanX (MCScanX, RRID:SCR_022067) to identify

syntenic blocks [45]. The inter species syntenic genomic blocks were visualized via the R package Ideogram [46]. Based on the collinearity alignments of the five mantises, the X chromosomes of the other four mantises were also identified. In addition, the translocation sites on chromosomes X1 and X2 were inferred from the collinearity alignment.

Evolutionary analysis

Seven Dictyoptera species, including the five mantises sequenced in this study, as well as *B. germanica* (PRJNA203136 in NCBI) [24] and *Z. nevadensis* (PRJNA203242 in NCBI) [25], were used to construct orthologous groups (OG) and infer orthologous genes via OrthoFinder (v2.5.4) (OrthoFinder, RRID:SCR_017118) with the default parameters [47]. The protein sequences of single-copy genes from each species were multiple aligned using MAFFT (v7.487) (MAFFT, RRID:SCR_011811) and then concatenated into one super protein sequence. Using the concatenated super protein sequence alignment, RAxML (v8.2.12) (RAxML, RRID:SCR_006086) was subsequently employed to construct a maximum-likelihood phylogenetic tree with the PROTGAMMALGX ("PROT" means protein sequence, "GAMMA" means gamma distribution, "LG" refers to amino acid substitution model, "X" means maximum likelihood estimation) model [48]. To verify the topology of the ML-tree, a NJ-tree was also constructed using the neighbor joining algorithm in MEGA (X) (MEGA, RRID:SCR_002805) [49]. The time tree was inferred using the Reltime-Branch lengths method in MEGA, with input of user-supplied branch lengths derived from Maximum Likelihood (ML) method. The time tree was computed using 1 calibration constraint (70-80 Mya between M.violacea and M.religiosa, doi: 10.1126/science.1257570).

Abbreviations

BLAST: Basic Local Alignment Search Tool; bp: base pairs; BUSCO: Benchmarking Universal Single-Copy Orthologs; BWA: Burrows-Wheeler Aligner; CCS: circular consensus sequencing; Gb: gigabase pairs; GO: Gene Ontology; kb: kilobase pairs;

KEGG: Kyoto Encyclopedia of Genes and Genomes; Ma: megaannus; Mb: megabase pairs; MYA: million years ago; NCBI: National Center for Biotechnology Information; NR: Non-Redundant; OG: orthologous groups; PacBio: Pacific Biosciences; PE: Paired end; RAxML: Randomized Axelerated Maximum Likelihood; TRF: Tandem Repeats Finder; TE: transposable element; TPM: transcripts per million; YaHS: yet another Hi-C scaffolding.

Acknowledgments

This work was supported by Shenzhen Science and Technology Program (Grant No. KQTD20180411143628272), Fund of Key Laboratory of Shenzhen (ZDSYS20141118170111640), and The Agricultural Science and Technology Innovation Program.

Data availability

The genomic and transcriptomic sequencing reads have been deposited in NCBI-SRA under the accession PRJNA987019, PRJNA989593, PRJNA989036, PRJNA988270, PRJNA989282 for *M. religiosa*, *T. sinensis*, *D. truncata*, *H. coronatus* and *M. violacea*, respectively. The corresponding genome assemblies and annotations have been deposited at NCBI-Genome under the accessions JAUKNK000000000, JAUKNM000000000, JAUKNL000000000, JAUKNX000000000, JAUJEO000000000, and are also available at Figshare [50-54]. Other data supporting this work are available in the GigaScience GigaDB database [55].

Author contributions

H.L. and L.L. prepared the sequencing samples, performed data analysis, and wrote the raw manuscript. W.F. and G.W. supervised the project and revised the manuscript. The other authors provided helpful suggestions, and all authors read and approved the final version of this manuscript.

Competing interests

The authors declare no competing interest.

Figures and tables

Figure 1. **Overall view of genome assembly and annotation**. Circos plots for *M. religiosa.* (A) *T. sinensis* (B) *D. truncata* (C) *H. coronatus* (D) and *M. violacea* (E). Each circos plot has 4 tracks: track A represents chromosome length, track B represents gene density, track C represents transposable element (TE) density, and track D represents GC percentage. Feature density and GC percentage were calculated by sliding 1-Mb windows. The images of the sequenced specimens were shown in the center of circos plots. (F) BUSCO assessment (database: Insecta from OrthoDB v10) of gene sets for five mantis species. M means missing, F means fragmented, and C means complete.

Figure 2. **TE distribution in five mantis genomes.** (A) The relationship between genome size and TE content. A linear trend Line in average of all the points was shown. Phylogenetic Generalised Least Squares Regression (PGLS) was applied, resulting in R=0.954 and p = 0.008 < 0.01, suggesting that there is very strong correlation between genome size and TE content. (B) The radar chart for major components of TE. Each vertex refers to a type of TE, and species were differed by colors. The percentage for each height level of the vertex represents the percentage of genome occupied by TEs. LINEs, Tc1-IS630-Pogo, and Helitron have sharp peaks, indicating a burst of TEs for that type. (C-E) The divergence (%) distribution of LINE, Tc1, and *Helitron*, respectively.

Figure 3. **Evolution of X chromosomes in Mantodea.** (A) Identification of X chromosome in *T. sinensis* by comparing depths between male and female individual. The sequencing depth distributions were plotted in 500 Kb windows. The red line represents the average sequencing depth for each chromosome. (B) The synteny band plot among *T. sinensis*, *D. truncata* and *M. violacea*, using 9,117 reciprocal best hits between *T. sinensis* and *D. truncata*, as well as 8,765 reciprocal best hits between *D. truncata* and *M. violacea*. Much more chromosome rearrangements were observed between *M. violacea* and *D. truncata*, in comparison to those between *T. sinensis* and *D. truncata*. (C) The dual synteny between *M. religiosa* and *T. sinensis*, using 9,917 reciprocal best hits between *M. religiosa* and *T. sinensis*. All chromosomes have

1:1 relationship. (D) The dual synteny between *D. truncata* and *H. coronatus*, using 9,821 reciprocal best orthologous genes between *D. truncata* and *H. coronatus*. Four chromosome breaks and three inter-chromosome translocations were observed. (E) The diagram shows the evolutionary process of the X chromosome along various lineages of Mantodea. For subfigures B-E, black box represents for X chromosomes, while gray box represents for autosomes.

Figure 4. **Orthologous groups and phylogeny of Mantodea** . (A) Percentage of orthologous groups (OG) shared with cockroach for each mantis species. When an OG contain one or more genes for both an analyzed mantis and the cockroach, it was counted for the analyzed mantis as shared OGs. (B) Phylogeny is based on protein sequence alignment of 4,014 single copy genes (mantises, cockroaches, and termites) with Maximum Likelihood (ML) method using LG amino acid substitution model. The branch length is in proportional with the substitution rate. (C) Time tree inferred by the Reltime-Branch lengths method in MEGA, with 1 calibration constraint (70-80 Mya between M.violacea and M.religiosa).

Table 1. Statistics of genome assembly and annotation

**Genomic features**	***M. religiosa***	***T. sinensis***	***D. truncata***	***H. coronatus***	***M. violacea***
**Genome assembly**					
Estimated genome size by K-mer (bp)	**3,519,843,697**	**2,865,686,147**	**4,337,798,490**	**3,167,239,197**	**2,331,221,057**
Total assembly size (bp)	**3,680,002,721**	**2,687,426,722**	**4,290,792,545**	**3,127,590,514**	**2,322,129,794**
Contig N50 size (bp)	**1,407,320**	**12,728,340**	**44,444,664**	**71,519,735**	**109,157,195**
Scaffold N50 size (bp)	**210,326,877**	**190,002,057**	**248,405,437**	**159,059,693**	**125,733,329**
# of assembly-inferred chromosomes	**12+X1+X2**	**12+X1+X2**	**15+X**	**20+X**	**16+X**
% sequence anchored to chromosome	**85.39%**	**95.63%**	**97.47%**	**98.27%**	**98.51%**
**Genome annotation**					
Length and % of tandem sequences (bp)	**396,842,330 (10.8%)**	**403,304,947 (15.0%)**	**471,243,565 (11.0%)**	**238,530,960 (7.6%)**	**186,949,249 (8.1%)**
Length and % of TE sequences (bp)	**2,501,898,483**	**1,710,668,926**	**2,928,636,453**	**2,122,785,940**	**1,351,077,317**

	**(68%)**	**(64%)**	**(68%)**	**(68%)**	**(58%)**
Number of protein-coding gene models	**19,017**	**19,007**	**18,156**	**18,536**	**17,804**
Mean CDS length (bp)	**1551**	**1782**	**1601**	**1523**	**1152**
Mean exon number	**6.07**	**5.93**	**6.34**	**6.33**	**5.54**

Note: "# of assembly-inferred chromosomes" refers to autosome and sex chromosome number in female.

References

1. Rankin EEW, Shmerling AJ, Knowlton JL and Hoey-Chamberlain R. Diets of two non-native praying mantids (Tenodera sinensis and Mantis religiosa) show consumption of arthropods across all ecological roles. Food Webs. 2023;35 doi:ARTN e00280 10.1016/j.fooweb.2023.e00280.
2. Song JH, Cha JM, Moon BC, Kim WJ, Yang S and Choi G. Mantidis Ootheca (mantis egg case) original species identification via morphological analysis and DNA barcoding. J Ethnopharmacol. 2020;252 doi:ARTN 112574 10.1016/j.jep.2020.112574.
3. Yu HY, Han ZW, Zhang JQ and Zhang SJ. Bionic design of tools in cutting: Reducing adhesion, abrasion or friction. Wear. 2021;482 doi:ARTN 203955 10.1016/j.wear.2021.203955.
4. Li M, Yang YW, Guo L, Chen DH, Sun HL and Tong J. Design and Analysis of Bionic Cutting Blades Using Finite Element Method. Appl Bionics Biomech. 2015;2015 doi:Artn 471347 10.1155/2015/471347.
5. Bachtrog D, Kirkpatrick M, Mank JE, McDaniel SF, Pires JC, Rice W, et al. Are all sex chromosomes created equal? Trends in Genetics. 2011;27 9:350-7. doi:https://doi.org/10.1016/j.tig.2011.05.005.
6. Bachtrog D, Mank JE, Peichel CL, Kirkpatrick M, Otto SP, Ashman TL, et al. Sex determination: why so many ways of doing it? PLoS Biol. 2014;12 7:e1001899. doi:10.1371/journal.pbio.1001899.
7. Rowe L, Chenoweth SF and Agrawal AF. The Genomics of Sexual Conflict. Am Nat. 2018;192 2:274-86. doi:10.1086/698198.
8. Evangelista DA, Wipfler B, Bethoux O, Donath A, Fujita M, Kohli MK, et al. An integrative phylogenomic approach illuminates the evolutionary history of cockroaches and termites (Blattodea). Proc Biol Sci. 2019;286 1895:20182076. doi:10.1098/rspb.2018.2076.
9. Svenson GJ and Whiting MF. Reconstructing the origins of praying mantises (Dictyoptera, Mantodea): the roles of Gondwanan vicariance and morphological convergence. Cladistics. 2009;25 5:468-514. doi:10.1111/j.1096-0031.2009.00263.x.
10. Svenson GJ, Hardy NB, Wightman HMC and Wieland F. Of flowers and twigs: phylogenetic revision of the plant-mimicking praying mantises (Mantodea: Empusidae and Hymenopodidae) with a new suprageneric classification. Syst Entomol. 2015;40 4:789-834. doi:10.1111/syen.12134.
11. Hornig MK, Haug JT and Haug C. An exceptionally preserved 110 million years old praying mantis provides new insights into the predatory behaviour of early mantodeans. PeerJ. 2017;5:e3605. doi:10.7717/peerj.3605.
12. Fukui M, Fujita M, Tomizuka S, Mashimo Y, Shimizu S, Lee CY, et al. Egg structure and outline of embryonic development of the basal mantodean, Metallyticus splendidus Westwood, 1835 (Insecta, Mantodea, Metallyticidae). Arthropod Struct Dev. 2018;47 1:64-73. doi:10.1016/j.asd.2017.11.001.
13. Ma Y, Zhang LP, Lin YJ, Yu DN, Storey KB and Zhang JY. Phylogenetic relationships and divergence dating of Mantodea using mitochondrial phylogenomics. Syst Entomol. 2023; doi:10.1111/syen.12596.

14. Huang G, Song L, Du X, Huang X and Wei F. Evolutionary genomics of camouflage innovation in the orchid mantis. Nat Commun. 2023;14 1:4821. doi:10.1038/s41467-023-40355-1.
15. Yuan R, Zheng B, Li Z, Ma X, Shu X, Qu Q, et al. The chromosome-level genome of Chinese praying mantis Tenodera sinensis (Mantodea: Mantidae) reveals its biology as a predator. GigaScience. 2023;12 doi:10.1093/gigascience/giad090.
16. del Cerro AL, Cunado, N. & Santos, J.L. Synaptonemal complex analysis of the X1X2Y trivalent in Mantis religiosa L. males: inferences on the origin and maintenance of the sex-determining mechanism. Chromosome Research. 1998;6:5-11. doi:https://doi.org/10.1023/A:1009258122785.
17. Li XT and Nicklas RB. Mitotic Forces Control a Cell-Cycle Checkpoint. Nature. 1995;373 6515:630-2. doi:DOI 10.1038/373630a0.
18. Liu B, Shi Y, Yuan J, Hu X, Zhang H, Li N, et al. Estimation of genomic characteristics by analyzing k-mer frequency in de novo genome projects. arXiv: Genomics. 2013.
19. Elliott TA and Gregory TR. Do larger genomes contain more diverse transposable elements? Bmc Evol Biol. 2015;15 doi:ARTN 69
10.1186/s12862-015-0339-8.
20. Ellegren H. Sex-chromosome evolution: recent progress and the influence of male and female heterogamety (vol 12, pg 157, 2011). Nat Rev Genet. 2011;12 10:736-. doi:10.1038/nrg3081.
21. Maryańska-Nadachowska A, Kuznetsova VG, Lachowska D and Drosopoulos S. Mediterranean species of the spittlebug genus Philaenus : Modes of chromosome evolution. Journal of Insect Science. 2012;12 1 doi:10.1673/031.012.5401.
22. King R. Chromosomes of three species of mantidae. Journal of Morphology. 2005;52:525 - 33. doi:10.1002/jmor.1050520208.
23. Paliulis LV, Stowe EL, Hashemi L, Pedraza-Aguado N, Striese C, Tulok S, et al. Chromosome number, sex determination, and meiotic chromosome behavior in the praying mantid Hierodula membranacea. PLoS One. 2022;17 8:e0272978. doi:10.1371/journal.pone.0272978.
24. Harrison MC, Jongepier E, Robertson HM, Arning N, Bitard-Feildel T, Chao H, et al. Hemimetabolous genomes reveal molecular basis of termite eusociality. Nat Ecol Evol. 2018;2 3:557-66. doi:10.1038/s41559-017-0459-1.
25. Terrapon N, Li C, Robertson HM, Ji L, Meng X, Booth W, et al. Molecular traces of alternative social organization in a termite genome. Nature Communications. 2014;5 1:3636. doi:10.1038/ncomms4636.
26. Svenson GJ and Whiting MF. Phylogeny of Mantodea based on molecular data: evolution of a charismatic predator. 2004;29 3:359-70. doi:https://doi.org/10.1111/j.0307-6970.2004.00240.x.
27. Guan D, McCarthy SA, Wood J, Howe K, Wang Y and Durbin R. Identifying and removing haplotypic duplication in primary genome assemblies. Bioinformatics. 2020;36 9:2896-8. doi:10.1093/bioinformatics/btaa025 %J Bioinformatics.
28. Cheng H, Concepcion GT, Feng X, Zhang H and Li H. Haplotype-resolved de novo assembly using phased assembly graphs with hifiasm. Nature Methods. 2021;18 2:170-5. doi:10.1038/s41592-020-01056-5.
29. Simão FA, Waterhouse RM, Ioannidis P, Kriventseva EV and Zdobnov EM. BUSCO: assessing genome assembly and annotation completeness with single-copy orthologs. Bioinformatics. 2015;31 19:3210-2. doi:10.1093/bioinformatics/btv351 %J Bioinformatics.
30. Zhou C, McCarthy SA and Durbin R. YaHS: yet another Hi-C scaffolding tool. Bioinformatics.

2023;39 1 doi:10.1093/bioinformatics/btac808.

31. Langmead B and Salzberg SL. Fast gapped-read alignment with Bowtie 2. Nat Methods. 2012;9 4:357-9. doi:10.1038/nmeth.1923.

32. Servant N, Varoquaux N, Lajoie BR, Viara E, Chen CJ, Vert JP, et al. HiC-Pro: an optimized and flexible pipeline for Hi-C data processing. Genome Biol. 2015;16:259. doi:10.1186/s13059-015-0831-x.

33. Wang S, Wang H, Jiang F, Wang A, Liu H, Zhao H, et al. EndHiC: assemble large contigs into chromosome-level scaffolds using the Hi-C links from contig ends. BMC Bioinformatics. 2022;23 1:528. doi:10.1186/s12859-022-05087-x.

34. Flynn JM, Hubley R, Goubert C, Rosen J, Clark AG, Feschotte C, et al. RepeatModeler2 for automated genomic discovery of transposable element families. Proceedings of the National Academy of Sciences. 2020;117 17:9451-7. doi:10.1073/pnas.1921046117.

35. Benson G. Tandem repeats finder: a program to analyze DNA sequences. Nucleic Acids Res. 1999;27 2:573-80. doi:10.1093/nar/27.2.573 %J Nucleic Acids Research.

36. Stanke M, Keller O, Gunduz I, Hayes A, Waack S and Morgenstern B. AUGUSTUS: ab initio prediction of alternative transcripts. Nucleic Acids Research. 2006;34 suppl_2:W435-W9. doi:10.1093/nar/gkl200 %J Nucleic Acids Research.

37. Chen S, Zhou Y, Chen Y and Gu J. fastp: an ultra-fast all-in-one FASTQ preprocessor. Bioinformatics. 2018;34 17:i884-i90. doi:10.1093/bioinformatics/bty560 %J Bioinformatics.

38. Pertea M, Pertea GM, Antonescu CM, Chang TC, Mendell JT and Salzberg SL. StringTie enables improved reconstruction of a transcriptome from RNA-seq reads. Nat Biotechnol. 2015;33 3:290-5. doi:10.1038/nbt.3122.

39. Haas BJ, Salzberg SL, Zhu W, Pertea M, Allen JE, Orvis J, et al. Automated eukaryotic gene structure annotation using EVidenceModeler and the Program to Assemble Spliced Alignments. Genome Biology. 2008;9 1:R7. doi:10.1186/gb-2008-9-1-r7.

40. Slater GS and Birney E. Automated generation of heuristics for biological sequence comparison. BMC Bioinformatics. 2005;6:31. doi:10.1186/1471-2105-6-31.

41. Buchfink B, Reuter K and Drost HG. Sensitive protein alignments at tree-of-life scale using DIAMOND. Nat Methods. 2021;18 4:366-8. doi:10.1038/s41592-021-01101-x.

42. Jones P, Binns D, Chang HY, Fraser M, Li W, McAnulla C, et al. InterProScan 5: genome-scale protein function classification. Bioinformatics. 2014;30 9:1236-40. doi:10.1093/bioinformatics/btu031.

43. Li H and Durbin R. Fast and accurate short read alignment with Burrows-Wheeler transform. Bioinformatics. 2009;25 14:1754-60. doi:10.1093/bioinformatics/btp324.

44. Li H, Handsaker B, Wysoker A, Fennell T, Ruan J, Homer N, et al. The Sequence Alignment/Map format and SAMtools. Bioinformatics. 2009;25 16:2078-9. doi:10.1093/bioinformatics/btp352.

45. Wang Y, Tang H, Debarry JD, Tan X, Li J, Wang X, et al. MCScanX: a toolkit for detection and evolutionary analysis of gene synteny and collinearity. Nucleic Acids Res. 2012;40 7:e49. doi:10.1093/nar/gkr1293.

46. Hao Z, Lv D, Ge Y, Shi J, Weijers D, Yu G, et al. RIdeogram: drawing SVG graphics to visualize and map genome-wide data on the idiograms. PeerJ Comput Sci. 2020;6:e251. doi:10.7717/peerj-cs.251.

47. Emms DM and Kelly S. OrthoFinder: phylogenetic orthology inference for comparative

genomics. Genome Biol. 2019;20 1:238. doi:10.1186/s13059-019-1832-y.

48. Kozlov AM, Darriba D, Flouri T, Morel B and Stamatakis A. RAxML-NG: a fast, scalable and user-friendly tool for maximum likelihood phylogenetic inference. Bioinformatics. 2019;35 21:4453-5. doi:10.1093/bioinformatics/btz305.

49. Kumar S, Stecher G, Li M, Knyaz C and Tamura K. MEGA X: Molecular Evolutionary Genetics Analysis across Computing Platforms. Mol Biol Evol. 2018;35 6:1547-9. doi:10.1093/molbev/msy096.

50. Lei L (2023). Genome assembly of Mantis religiosa. figshare. Dataset. https://doi.org/10.6084/m9.figshare.23995398.v2

51. Lei L (2023). Genome assembly of Tenodera Sinensis. figshare. Dataset. https://doi.org/10.6084/m9.figshare.23995410.v2

52. Lei L (2023). Genome assembly of Deroplatys truncata. figshare. Dataset. https://doi.org/10.6084/m9.figshare.23995152.v2

53. Lei L (2023). Genome assembly of Hymenopus coronatus. figshare. Dataset. https://doi.org/10.6084/m9.figshare.23988987.v2

54. Lei L (2023). Genome assembly of Metallyticus violaceus. figshare. Dataset. https://doi.org/10.6084/m9.figshare.23995434.v2

55. Liu H; Lei L; Jiang F; Zhang B; Wang H; Zhang Y; Zhao H; Wang G; Fan W; (2025): Supporting data for "The genomes of five mantises provide insights into sex chromosome evolution and Mantodea phylogeny clarification" GigaScience Database. https://doi.org/10.5524/102788

Figure 1

Click here to access/download;Figure;Figure1.pdf

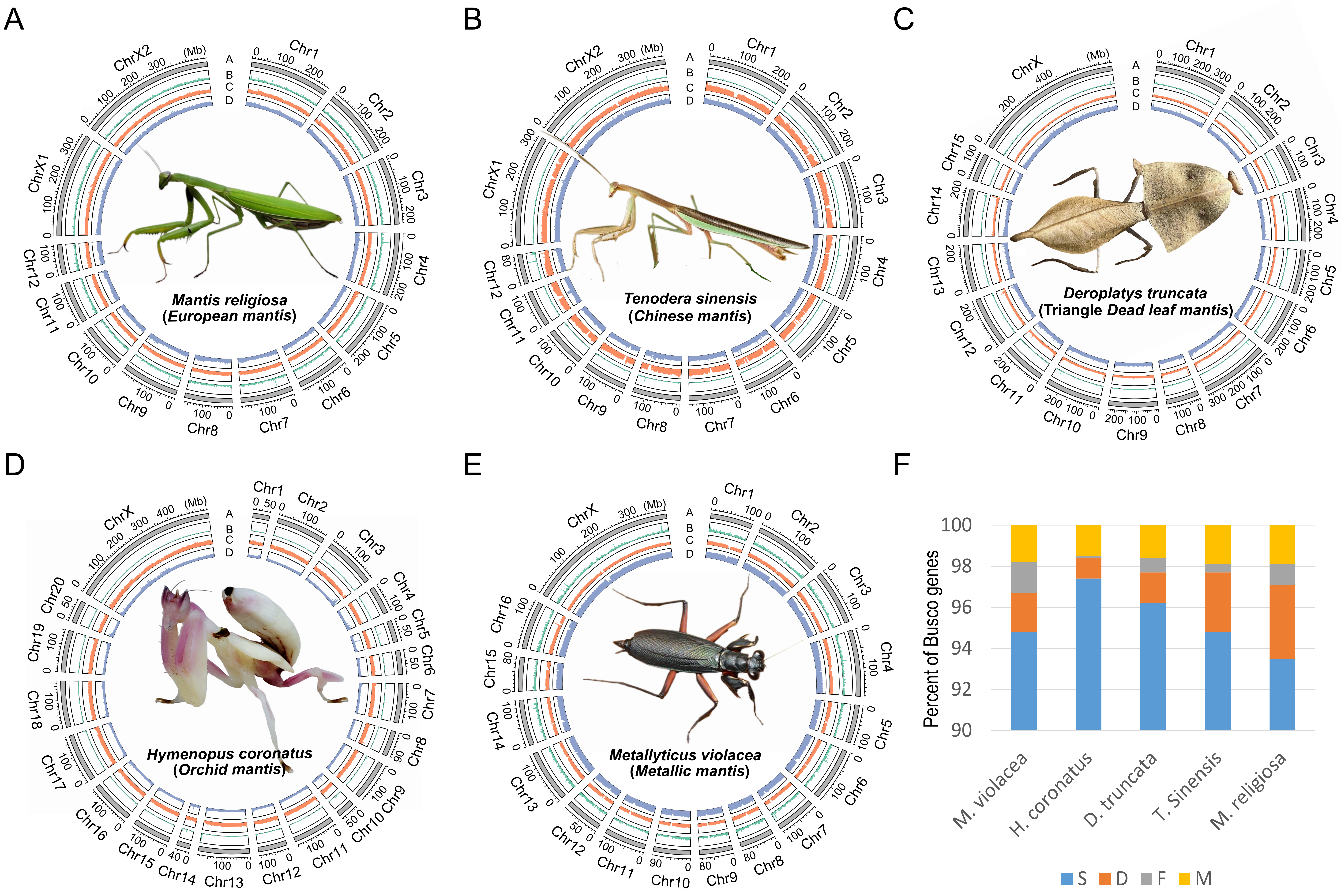

Figure 2

Click here to access/download;Figure;Figure2.pdf

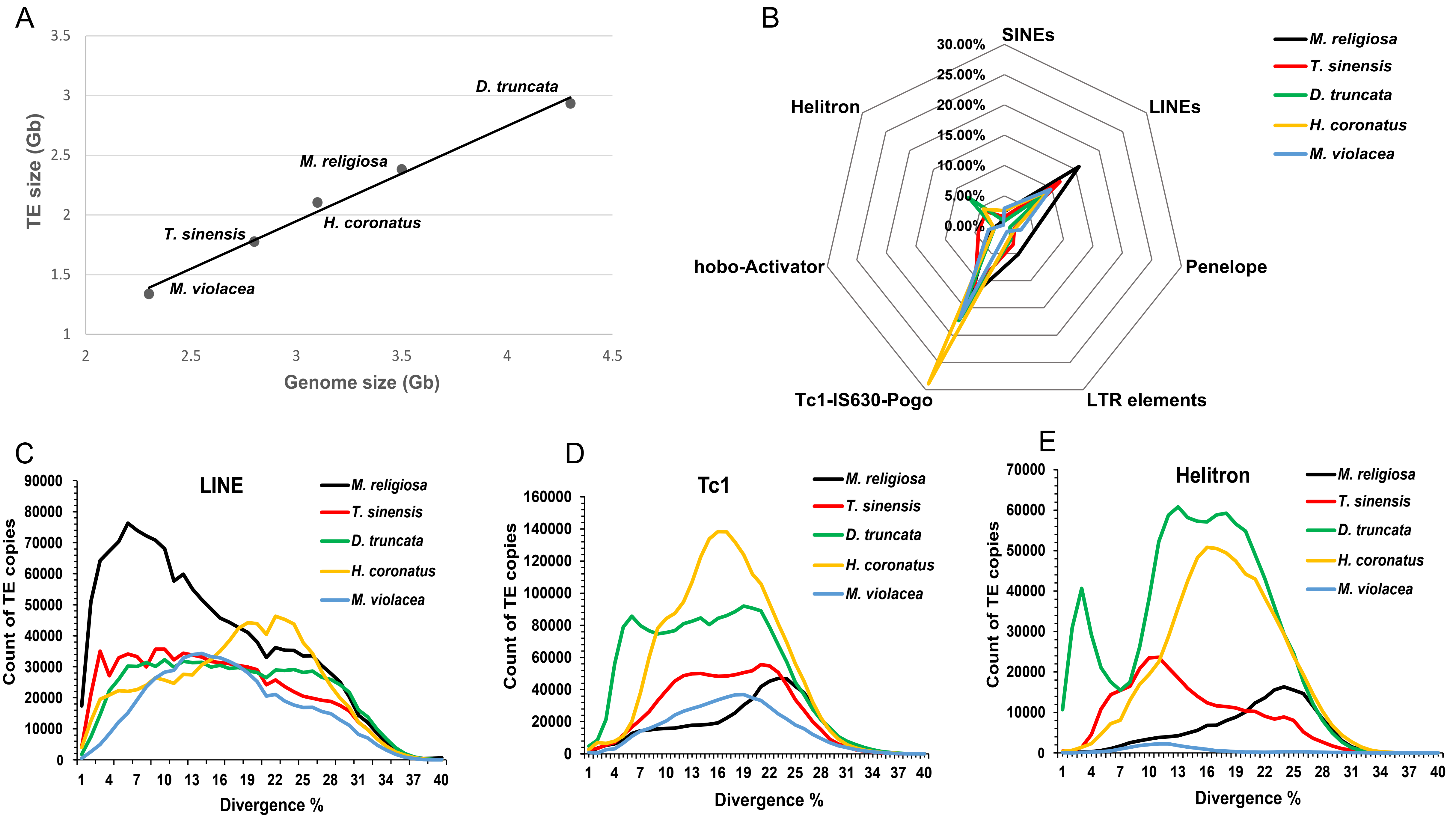

Figure 3

Click here to access/download;Figure;Figure3.pdf

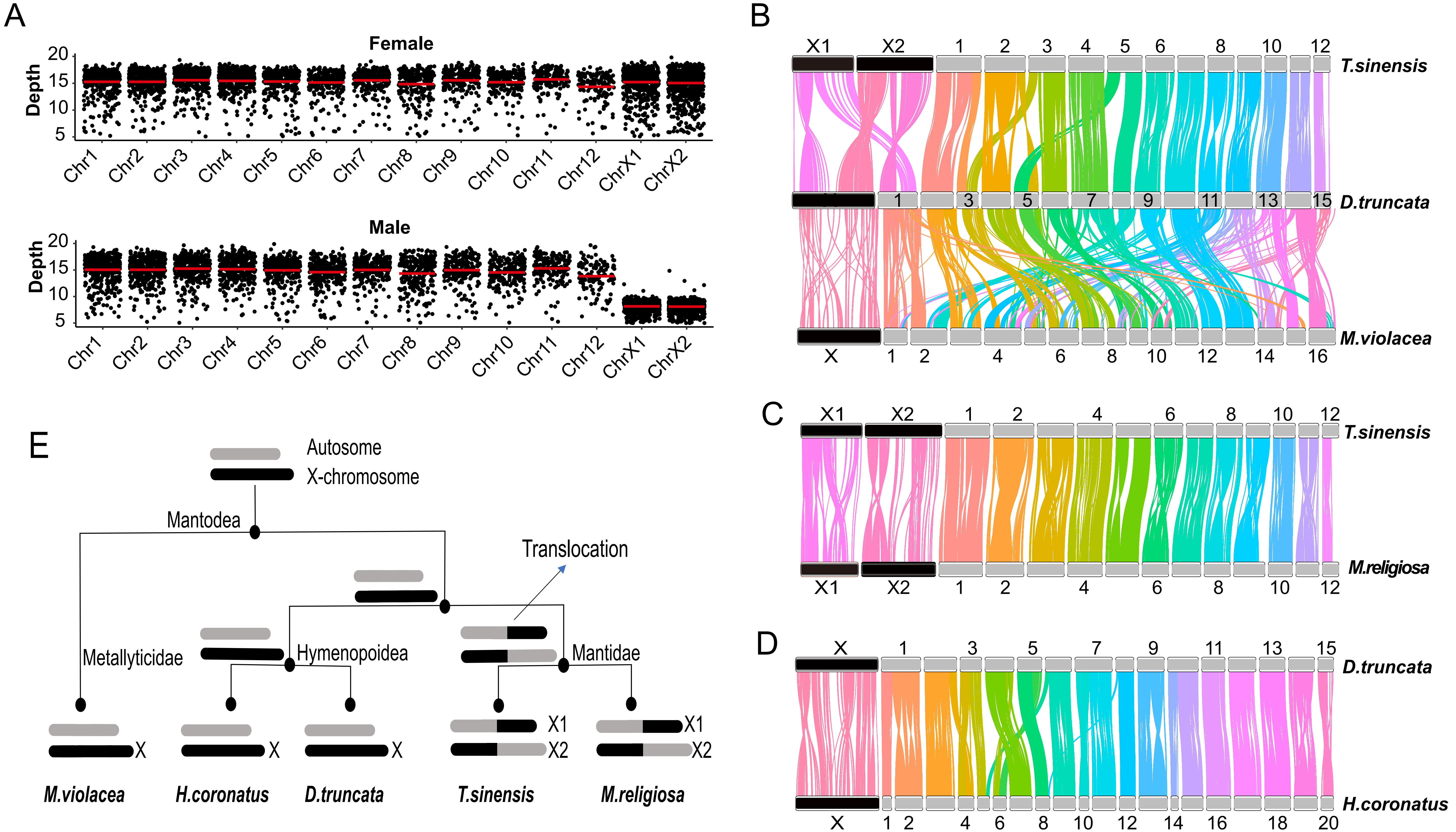

Figure 4 Click here to access/download;Figure;Figure4.pdf

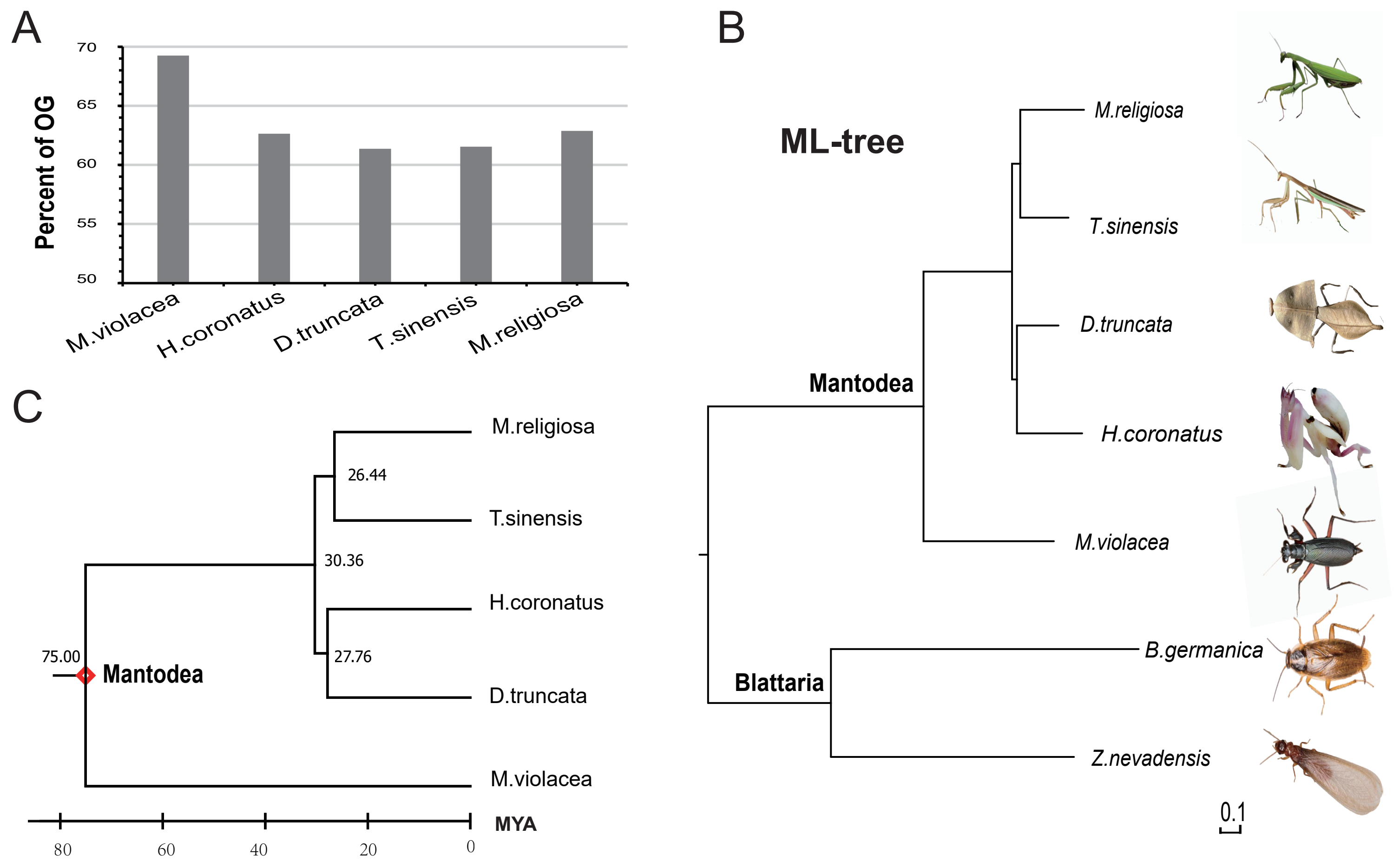

Click here to access/download
Supplementary Material
Supplemental materials - R2.docx
