# Supplementary material for: The genomes of 5 mantises provide insights into sex chromosome evolution and Mantodea phylogeny clarification
Source: Gigascience. 2025 Dec 18;15:giaf158. doi: 10.1093/gigascience/giaf158 (PMC12908712; doi:10.1093/gigascience/giaf158)
Supplement: giaf158_Authors_Response_To_Reviewer_Comments_original_submission [file giaf158_authors_response_to_reviewer_comments_original_submission.pdf]

# Author's Response To Reviewer Comments

Reviewer reports:

Reviewer #1: This manuscript presents chromosome-scale genomes for five mantis species, comparative repeat analyses, inferences about sex-chromosome evolution (X1/X2 vs. single X), rate heterogeneity (slow lineage in Metallyticus), and phylogenetic placement of Deroplatys. The datasets are potentially valuable; however, taxonomy/terminology, framing, and several core claims (especially on sex chromosomes) require substantial revision and additional detail.

Major comments
1) Taxonomic terminology and hierarchy (must fix throughout)
The usage of order/superfamily/family is repeatedly incorrect or inconsistent. Mantidae is a family, not a superfamily. Please audit the entire manuscript (title/abstract/main text/figures/legends/keywords) and ensure consistent, current placement within order Mantodea and the Dictyoptera clade. Add a short taxonomic note or cite an up-to-date checklist.

Reply: For Mantidae, we have changed “superfamily” into “family” anywhere in the maintext. To clarify the taxonomic hierarchy, we also added a sentence “Currently, Mantodea (order) and Blattodea (order) are placed within Dictyoptera (superorder). Mantidae is a representative mantis family, which belongs to Mantoidea (superfamily) in Mantodea.” in the maintext.

2) Abstract
Add key numbers to convey scale/quality (e.g., genome sizes ~2.3-4.3 Gb; contig/scaffold N50; % of sequence anchored to chromosomes; BUSCO range).

Reply: According to the reviewer’s suggestion. we have added a sentence “The assembled genome sizes range ~2.3-4.2 Gb, with contig N50 size 1-109 Mb and 85-99% of sequence anchored to chromosomes. The annotated protein-coding gene number ranges 17,804-19,017, with BUSCO complete rate 96.7-98.4%.” in Abstract.

Replace unclear wording:
"genetic breeding of efficient enemy insect" → "genetic improvement and breeding of beneficial biological control agents."

Reply: Corrected.

Phylogeny statement is vague. State the concrete relationship (e.g., Deroplatys truncata sister to Hymenopus coronatus) and soften to "helps resolve" rather than "providing important evidences for resolving."

Reply: We have made modifications based on the reviewer’s suggestions.

3) Introduction
The Introduction does not prepare readers for the sex-chromosome focus and underspecifies outstanding phylogenetic issues. Please:
(i) explicitly flag unresolved nodes your genomes will test (e.g., placement of Deroplatys; early branching of Metallyticus);
Reply: After “The metallic mantis (Metallyticus violacea), in the early diverging mantid lineage, has many morphological features similar to those of modern cockroaches,” we added “but the underlying mechanisms have not been discovered”.
“Morphological and molecular phylogenies are often inconsistent with previous classification schemes for

some sublineages such as Deroplatys genus, because of the rapid radiation events and convergent evolution of ecomorphological strategies" has been revised into "For example, Deroplatys genus has been placed into Mantidae family traditionally, but these species are more similar to species in Hymenopodidae family in many aspects such as morphology and camouflage".

(ii) briefly introduce known mantis karyotype diversity and why X1X2 vs. single X matters;
Reply: We added a sentence in the end of paragraph one in Introduction: "Although most manties have the common XY sex determination system, some mantises such as M. religiosa and T. sinensis have the X1X2Y type [5-7], making them a special material for studying the evolution of sex chromosomes.".

(iii) trim peripheral material and end with a clear statement of aims/hypotheses.
Reply: Thanks for the reviewer's kind suggestions, but we don't want to trim the peripheral material as the reviewer thought. We have to make some explanations: (1) As we are a research group from agricultural institute, our original aim to sequence these genomes is taking them as the enemy insect, and providing genomic resource for the future genetic breeding jobs. (2) With the genomic sequences, we have some preliminary findings on TE content, X1X2Y chromosomes, slow evolution rate of M. violacea, and new placement for Deroplatys, but these are not our original aims/hypotheses. That's why we wrote the introduction in the current style.

4) Methods
Species identification: describe how each sampled species was confirmed (diagnostic morphology, DNA barcoding), voucher numbers, and deposition.

Reply: We added a sentence "The species were confirmed by morphological characters, and the photos for sequenced individuals were shown in Figure 1." In Methods.

Biological replicates: clearly state how many male and female individuals were sequenced per species (genome and transcriptome). If single-individual per sex was used, acknowledge this limitation explicitly.

Reply: For HiFi and HiC sequencing, only one female adult was used for each species. For T. sinensis, a male adult and another female adult were used for Illumina sequencing. For transcriptome sequencing, the seven different tissues from each female adult for each species were used. There is no replicate for transcriptome sequencing, as these data were mainly used to facilitate gene annotation. We have clarified these information in the revised manuscript.

Transcriptome tissues: two "midfoot" entries appear; the latter likely means hindfoot—please correct.

Reply: Corrected.

P316: "for each mantis, the protein sequences from the other 4 mantises were …"—list the four species explicitly to remove ambiguity. (maybe 5 mantises)

Reply: We revised this sentence into "for each mantis, the protein sequences from the other 4 sequenced mantises in this study were".

5) X-chromosome identification and strength of inference
Depth-based identification in T. sinensis is reasonable, but provide normalized male/female coverage ratios (e.g., CQ) with sliding-window plots, variance/CI, and GC/repeat normalization; specify the number of replicates per sex. For the other genera, assignments based on comparative synteny should be described as inference, not definitive identification.

Reply: Thanks for the reviewer's kind suggestions. We have shown the coverage depth values for all the sliding windows of each chromosome in Figure 3a, it is easy to see the variance of the depth. The average

depth was shown as a red line for each chromosome, and the coverage ratio for chromosome 1-12 and chromosome X1-X2 is about 2 for the male. When seeing Figure 3a, it is easy to make a solid conclusion that X1 and X2 are the two sex chromosomes. There is no replicate for each sex. To be more rigorous, we changed “Macroscale synteny analysis identified the corresponding X chromosomes in the other 4 species” into “Macroscale synteny analysis inferred the corresponding X chromosomes in the other 4 species”.

Avoid "confirmed"; use "supports a model in which …" and separate plausible scenarios (fusion vs. fission vs. translocation). Acknowledge limited taxon sampling (two Mantidae spp.).

Reply: We changed “we confirmed” into “our results support a model in which”. We changed “resulted from the fragmentation and fusion of one X chromosome and an autosome” into “from the translocation between one X chromosome and an autosome”. We also added “Based on very limited taxon sampling, we made a set of preliminary conclusions in this study, which will be verified by future studies as more genomes being sequenced.” in the Discussion part.

6) Breakpoint localization claim (~2.5 Mb)
Provide methods and support: genomic coordinates, anchor density, long-read overlaps across the putative break, and Hi-C contact changes; include an uncertainty interval. Clarify whether this interval is species-specific or an inference for the Mantidae ancestor.

Reply: Thanks for the reviewer’s kind suggestion. In the old manuscript, we firstly used macroscale synteny to locate the breakpoint region, then we used genomic sequence alignment to further narrow down the range. In this revised manuscript, we found that small inversions, deletions, and other rearrangements occurred in these regions, making the conclusion from genomic sequence alignment not accurate. Therefore, we decide to only report the conservative result obtained from macroscale synteny analysis, which we believe to be confidence. M. religiosa was not suitable for this analysis, as its current genome assembly is too fragmental, and the old Fig. S10 was removed in the revised manuscript. This time, we only used T. sinensis for performing this analysis, and tone down our conclusion. We changed Fig 4F in the old manuscript into Fig S10 in the revised manuscript.

We revised the description in Results: “Furthermore, based on macroscale synteny analysis, we were able to identify the breakpoints as a site falling within a 6.65-Mb region on the X1 chromosome and a site falling within 2.56-Mb region on the X2 chromosome of T. sinensis. Inside these two regions, transposon and tandem repeats dominate the sequence (Figure S10), posing great difficulties for accurate genome assembly and inter-species genomic sequence alignment. In future, as the assembly continuity improves, it is possible to narrow down the breakpoint range, which will approach or surpass the resolution of traditional cytological technologies such as C-banding, silver staining and living-cell images of the meiosis process [16, 23].”

We also revised the legend of Fig S10: “Figure S10. Location of the broken site for translocation on the X1 and X2 chromosomes of T. sinensis. TE refers to transposable element, and TR refers to tandem repeats. The gene density was shown in heatmap, while the TE/TR density were shown in distribution curves. Through macroscale synteny analysis between T. sinensis and D. truncata using reciprocal-best orthologous genes as markers, the broken site on ChrX1 (length 318,123,395 bp) was located to a 6.65 Mb region (107,057,327- 113,703,447 bp), and the broken site on ChrX2 (length 396,970,917 bp) was located to a 2.56 Mb region (171,940,730-174,498,078 bp). X1L and X2L were derived from ancestral X chromosome, while X1R and X2R were derived from ancestral autosome. Each broken site region has an assembly gap inside, which are filled with absolute 1,000 N characters. Therefore, the size estimation of broken site region was not so accurate. In addition, there are abundant TR and TEs in the broken site range, making it difficult to identify the accurate position where translocation happened. M. religiosa was not suitable for this analysis, because its current genome assembly is too fragmental.”.

7) Heterozygosity vs. contiguity (discussion too thin)
The statement that lower contiguity in M. religiosa/T. sinensis is due to higher heterozygosity needs

numbers (k-mer heterozygosity estimates and spectra) and clarification on phasing/purge_dups/trio-binning. Report exact contig N50/N90 and % duplication before/after purging. Expand the discussion with appropriate citations.

Reply: The contig length statistics does not change much before/after purging duplications. The main reason for contig length in this study is heterozygosity, which was shown in the peak heights of Kmer frequency curves in Figure S3.

We added a sentence in the legend of Figure S3: "With higher heterozygous rate, the left/right peak height ratios will be larger. The ratio of the left/right peak heights show that M. religiosa and T. sinensis have much higher heterozygosity than the other 3 mantis species.".

We also added a sentence "Based on GCE heterozygosity model [15], the estimated heterozygous rates are approaching 2% for M. religiosa and T. sinensis, whereas about 0.05-1% for the other three species." in the maintext.

8) Phylogeny and rate heterogeneity
Define the substitution-rate analysis (loci, clock model, calibrations, tests of rate constancy, CIs).

Reply: In our substitution-rate analysis, the input data was the multiple-aligned protein sequences of single-copy genes from each species. Then, command "raxmlHPC -m PROTGAMMALGX" was applied. In the model "PROTGAMMALGX", "PROT" means protein, "GAMMA" means gamma distribution, "LG" refers to the amino acid substitution model, "X" means maximum likelihood. We have added more descriptions for "PROTGAMMALGX" in the method part.

Rephrase the teleological sentence: instead of "allowing M. violaceus to conserve more ancestral traits," use "consistent with retention of ancestral gene content/signals."

Reply: We changed "allowing M. violaceus to conserve more ancestral traits" into "consistent with its retention of ancestral traits.".

The statement that M. violaceus "shares more orthologous genes with cockroaches" requires a precise metric (e.g., number of 1:1 orthologs from OrthoFinder; control for annotation size).

Reply: This conclusion is based on Figure 4A, which shows the percentage of orthologous groups (OG) shared with cockroach for each mantis species. We have added more description in the legend of Figure 4A: "When an OG contain one or more genes for both an analyzed mantis and the cockroach, it was counted for the analyzed mantis as shared OGs.".

For the Deroplatys placement, report support (UFBoot/SH-aLRT/ASTRAL LPP; gene/site-concordance factors) and briefly discuss potential discordance (ILS/introgression) given rapid radiations.

Reply: Thanks very much for the reviewer's suggestions. For the Deroplatys placement, our analysis can only give a very preliminary conclusion, mainly due to the very limited taxon sampling. Thus, it is out of our ability and interest to perform more professional analyses on phylogeny assignment.

9) The manuscript requires a thorough language edit by a fluent scientific editor.

Reply: We have tried our best to improve the language.

Reviewer #2: The manuscript presents chromosome-level genome assemblies for five species of mantis, describing their patterns of synteny and phylogenetic relationships. Most of the conclusions are well supported and the manuscript well written. The genomes will serve as a useful resource for future ecological and evolutionary studies in this group and beyond. Overall, I recommend it for publication in

GigaScience. However, several aspects need to be addressed first.

Major comments:
- The title needs to be changed as while the manuscript describes the evolution of additional sex chromosomes, there is no investigation into sex determination mechanisms. E.g. replace 'sex determination mechanism evolution' with 'sex chromosome evolution'.

Reply: Corrected.

- Without dating the tree, substitution rates and time are confounded, preventing the authors from making comments on substitution rates. Moreover, the large distances of evolutionary time between lineages make inferring substitution rates extremely hard. These claims should therefore be revised.

Reply: To explain how to estimate the substitution rate, we added a sentence in the maintext "Generally, the branch length is in proportional to the amino-acid substitution rate."

Besides, we added divergence time results in the maintext: "The divergence time was inferred by the Reltime-Branch lengths method, using one calibration constraint (70-80 Mya between M.violacea and M.religiosa). The results showed that four modern mantises (D.truncata, H.coronatus, M.religiosa, and T.sinensis) emerged within a short time period (26-31 Mya) (Figure S11), posing difficulties for phylogenic inferrence within this lineage." To emphasis this point, the original Figure S11 was moved to be Figure 4C.

- As the level of confidence in the phylogeny was not explored, the discussion should make it clear that more detailed phylogenomic analyses are required to get at some of the interesting questions that they would like to investigate in future.

Reply: We added "Based on very limited taxon sampling, we made a set of preliminary conclusions in this study, which will be verified by future studies as more genomes being sequenced." In the Discussion part.

- The generation of the Hi-C data is missing from the methods.

Reply: We added "Then, Hi-C data was generated using the same individuals applied for HiFi sequencing. Nuclear DNA was cross-linked by soaking leaf tissues in formaldehyde solution, and the cross-linked genomic DNA was extracted, digested, repaired, ligated to circular fragments, sheared into 350 bp inserts, converted to short-read sequencing library by Truseq DNA Library Prep Kit, and sequenced on Illumina NovaSeq 6000 platform (RRID:SCR_016387)." in the Methods.

- It is unclear to me why a different method for scaffolding was used for the genomes with fewer contigs. Please explain why the protocol used for the other genomes (the Arima mapping pipeline and YaHS) would not have been appropriate.

Reply: EndHiC is a software developed by our group, it was published in BMC bioinformatics in year 2022. EndHiC is especially suitable to construct chromosome-level scaffolds with relatively larger contigs (T. sinensis, D. truncata, H. coronatus, M. violacea), but it is not suitable for much shorter contigs (M. religiosa). So we turned to use YaHS, a more general algorithm, to resolve scaffolding for M. religiosa, whose contigs are very fragmental.

Minor comments

- The supplementary contains figures (e.g. figure S10-12) and includes methods that are not mentioned in the main text. Please make the supplementary consistent with the main text and adapt numbering accordingly.

Reply: Corrected.

Abstract:
- "Metallyticus violacea" should be changed to "Metallyticus violacea" as this version is used everywhere else in the text and used in NCBI's taxonomy.

Reply: We have changed all “violaceus” into “violacea”.

- "deduced that translocations between" should be changed to "suggest that translocations between" as the data suggests a translocation but does not prove it.
Reply: Corrected.

- The term 'Deroplatys' is used in the abstract without contextualising what this group is, please briefly describe what this is for readers unfamiliar with the taxonomy. This is also the case in the introduction.
Reply: We added “genus” after “'Deroplatys'”, to show it is a genus.

Introduction:
- Please explain in the introduction the significance/impact of understanding the relationships within Mantodea.

Reply: We added a sentence “Clarifying the relationships within Mantodea will greatly benefit the functional studies as well as genetic breeding of mantises” in Introduction.

Results:
- The results list useful statistics for each species (lines 79-96). However, this information would be much better suited to a table to allow comparison between species and statistics. Ranges in the results could suffice to summaries the table. It is important for downstream analyses of sex chromosomes that females were sequenced (i.e. lacked any Y chromosomes).

Reply: Thanks for the reviewer’s kind suggestion. In the whole manuscript, we describe each species separately. To be consistent in the style, we insist to list the numbers for each species in our study. All these information are also available in Table 1 and Table S2, which are suitable for the readers to make comparison.

- Please briefly explain the GCE method in the text for those less familiar with this.

Reply: We changed “Using the GCE method,” to “Using the GCE method that estimates genome size with K-mer frequency distribution from sequencing reads”.

- When comparing the genomes of H. coronatus from this study and from another study, please explain why the X chromosome is better assembled here. For example, was manual curation performed and does this explain the difference in quality?

Reply: Our assembly of X chromosome is complete, while it was broken into 3 fragments in Huang’s assembly. This is the main difference.

- Given that the authors describe a strong correlation between genome size and repeat content, I suggest adapting Fig S7 which plots these two variables as a graph as part of Fig1/2, adding a statistical test for the strength of correlation while accounting for shared ancestry (i.e. including the effect of phylogeny when testing for correlation) to verify this relationship.

Reply: According to the reviewer’s suggestion, we have moved one plot of Fig S7 into Figure 2. We

calculated the Pearson's correlation coefficient (r), and made a t-test to verify its independence. The results were shown in the legends of Figure 2A “A linear trend Line in average of all the points was shown. The Pearson's correlation coefficient (r) is as high as 0.99578, with t-test p-value < 0.001, suggesting that there is very strong correlation between genome size and TE content.” and Fig S7 “A linear trend Line in average of all the points was shown. The Pearson's correlation coefficient (r) is as high as 0.894, with t-test p≈0.038<0.05, suggesting that there is strong correlation between genome size and TE ratio.”.

- The authors write "Sex chromosomes evolved from autosomes and play important roles in tissue development, mating, and speciation [17-19]." which is not quite true, sex chromosomes can also evolve by other means e.g. from B chromosomes. Please revise this.

Reply: This sentence was revised to “Sex chromosomes evolved from autosomes or B chromosomes, and play important roles in tissue development, mating, and speciation”.

- It would be valuable to add a short description of the types of chromosome rearrangements that are suggested to have occurred in these genomes (based on the synteny plot), to shape the varying karyotypes of 2n=14-21. At the moment, only sex chromosomes are described but the autosomes have also experienced changes.

Reply: We added short descriptions of chromosome rearrangements in the legends of Figure 3: “(B) The synteny band plot among T. sinensis, D. truncata and M. violacea, much more chromosome rearrangements were observed between M. violacea and the other mantises. (C) The dual synteny between M. religiosa and T. sinensis, all chromosomes have 1:1 relationship. (D) The dual synteny between D. truncata and H. coronatus, including 4 chromosome brokens and 3 inter-chromosome translocations.”.

The statements about sex chromosome systems across insects need supporting with references

Reply: We have revised the reference in the revised manuscript “Sex chromosomes evolved from autosomes or B chromosomes, and play important roles in tissue development, mating, and speciation [17]. The types of sex chromosomes found in insects vary among species, and sex chromosome systems exhibit significant diversity across insect species [18-20].”.

- When describing the sex chromosome result of T. sinensis it should be mentioned that the genome was generated from a female (i.e. lacked any Y chromosomes) and hence two half coverage chromosomes in male are two Xs (rather than X/Y for example).

Reply: We added a sentence in the maintext “In addition, the genome assembly of T. sinensis was derived from a female individual, which lacked the Y chromosome.”.

- Some claims are too speculative based on the available data e.g. "M. violaceus shares much more OGs with cockroaches than the other 4 species (69% vs 61-63%) (Figure 4A), which may partly explain its strong morphological resemblance to cockroaches." (line 208) and similarly in the discussion "M. violaceus genome shares more orthologous genes with cockroaches than with the other mantises, and the mutation rate of M. violaceus genome is the lowest among all the mantises, allowing M. violaceus to conserve more ancestral traits." (line 244). This is also reflected in the title of this section being too strong "Low evolutionary rate allows M. violaceus conserve more ancestral traits" (line 194). Also, the authors do not describe the traits of M. violaceus which are "more ancestral".

Reply: We have tone-down on these conclusions, and described the ancestral traits for M. violacea in maintext “M. violacea belongs to the superfamily Metallyticoidea, exhibiting significant morphological differences compared to other mantises. M. violacea shares many characteristics with its cockroach relatives, including dull body colouration, a prostrate body posture, and a relatively shorter prothorax. The

cockroach-like body morphology was thought to be the ancestral traits of Mantodea (order).”.

- It would strengthen this section if the authors could assess the confidence in the assignment of D. truncata as sister to H. coronatus, or else make it clear that further analyses are needed.

Reply: Due to limited taxon sampling, it is difficult to assess the confidence in the assignment of D. truncata. Instead, we have added a sentence “Based on very limited taxon sampling, we made a set of preliminary conclusions in this study, which will be verified by future studies as more genomes being sequenced.” in Discussions.

- Please comment on why the N50 and N90 are so much higher in your assemblies than in previously published genomes e.g. differences in sequencing technology, assembly methods etc (line 228).

Reply: We added a comment sentence in the maintext “The difference in assembly continuity is manily caused by the applied sequencing technologies. We used HiFi long reads with over 99% accuracy, while they used common long reads with only ~85% accuracy.”.

- Heterozygosity is mentioned in a few places without being supported by any values (e.g. line 231)

Reply: We added the estimation of heterozygosity values in the maintext “Based on GCE heterozygosity model [15], the estimated heterozygous rates are approaching 2% for M. religiosa and T. sinensis, whereas about 0.05-1% for the other three species.”.

- Please could you clarify what you mean by "tissues with no or low heterozygosity are still a priority for de novo genome sequencing." and make it clearer that high heterozygosity can be problematic for genome assembly (line 232)

Reply: We have changed “tissues with no or low heterozygosity are still a priority for de novo genome sequencing” into “high heterozygosity can be problematic for genome assembly”.

- Please can you elaborate why Mantodea occupies "an important position in the evolution of insects" (line 235)

Reply: We revised this sentence into “Mantodea has occupied an important position in the evolution of insects, with distinctive morphology and camouflage behavior.”.

Comments on figures:
- Fig1F: the category of duplicated BUSCOs are missing, please add this. This also needs a Y-axis label.

Reply: We have divided complete genes (C) into single copy genes (S) and duplicated genes (D), and added a Y-axis label “Percent of Busco genes”.

- Fig2A: Please add a short description of how to interpret a radar chart and the main conclusion it supports.

Reply: We added a short description: “Each vertex refers to a type of TE, and species were differed by colors. LINEs, Tc1-IS630-Pogo, and Helitron have sharp peaks, indicating a burst of TEs for that type.” In the legend of Fig2A.

- Fig 2: As all panels have the same key I suggest having it just once and making it clear it applies to all panels in the figure legend. Please make the Y-axis in panels B,C and D more informative (i.e. counts of what)

Reply: The legend for Fig 2B,C,D was changed to "(B-D) The divergence (%) distribution of LINE, Tc1, and Helitron, respectively.". For the Y-axis, "counts" was changed to "count of TE copies"

- Fig 3: The 'X chromosome' in the title should be plural given more than one evolved.
Reply: We changed "X chromosome" into "chromosomes".

- Fig 3A: Please add an Y-axis label.
Reply: We added a Y-axis label "Depth".

- Fig 3B-D: Please include details about the orthologs that are plotted i.e. total number per plot and what these orthologs are e.g. all single copy orthologues in both species.

Reply: We have added the plotted ortholog gene number in the figure legend: "(B) The synteny band plot among T. sinensis, D. truncata and M. violacea, using 9,117 reciprocal best orthologous genes between T. sinensis and D. truncata, as well as 8,765 reciprocal best orthologous genes between D. truncata and M. violacea. Much more chromosome rearrangements were observed between M. violacea and the other mantises. (C) The dual synteny between M. religiosa and T. sinensis, using 9,917 reciprocal best orthologous genes between M. religiosa and T. sinensis. All chromosomes have 1:1 relationship. (D) The dual synteny between D. truncata and H. coronatus, using 9,821 reciprocal best orthologous genes between D. truncata and H. coronatus. Four chromosome brokens and three inter-chromosome translocations were observed.".

- Fig3B: The colouring of T. sinensis is strange. It would be helpful if X1 is the same colour as X to indicate shared orthologs.

Reply: We have updated the colors in Fig 3B-D, using black color for all the X chromosomes, and grep color for all the autosomes. This color scheme is also consistent with Fig 3E.
We added a sentence in the Fig 3 legend: "For B-E, black color represents for X chromosomes, while gray color represents for autosomes.".

- Fig 3E: Please annotate the figure with the node where the hypothesised translocation occurred and label the resulting chromosomes as X chromosomes. Black should be changed to 'X1-chromosome' given that an X2 is made in the figure but is half grey half black due to it being part ancestral autosome and part sex chromosome.

Reply: We have labeled the node that translocation occurred on Fig 3E, and also labeled all the X, X1, X2 chromosomes in Fig 3E.

- Fig 3F: Please increase the space between the key for gene number and that for TR and TE as it is hard to understand at present. Please also explain in the figure legend what TR and TE denote and their relevance to the breakpoint. It would also be helpful to note in the legend how this figure illustrates how the breakpoint was identified.

Reply: We have increased the space between key for gene number and that for TR and TE. We also added a sentence in legend of Fig 3F "TE refer to transposable element, TR refer to tandem repeats. Within the broken site range, TE and TR dominate the sequence.".

- Fig 4: I think that the figure legend title ("Orthologous groups and phylogeny of Mantodea") could be more informative. Also please clarify whether the following is referencing all OG groups in cockroaches that are found in each mantis, or that of mantis OGs found in cockroaches: "Percentage of orthologous groups (OG) shared with cockroach for each mantis species". Please add the substitution model used in maximum-

likelihood inference to the legend and add the scale that the branch lengths are given in.

Reply: We changed the figure legend title “Orthologous groups and phylogeny of Mantodea” into “Evolution of Mantodea lineages”. We added a descriptive sentence in the legend “When an OG contain one or more genes for both an analyzed mantis and the cockroach, it was counted for the analyzed mantis as shared OGs.”. We added “using LG amino acid substitution model” in the legend. The scale (length 0.1) was shown on the right-bottom corner of Figure 4B.

- Table 1: It would be helpful to add identified sex chromosomes and the sex that the genome is derived from.
Reply: Thanks for the reviewer’s suggestion, but we think Table 1 is quite large now, showing statistics for the assembly and annotation. The sex chromosomes of each mantis have been clearly shown in Figure 3.

- Fig S11: Please add more details to the legend e.g. which model was used in maximum-likelihood (ML). Also, in the methods, two ML approaches are described while this figure shows one tree from ML and one from NJ. Please ensure the figure and methods are consistent. Also the case for Fig S13.

Reply: We have revised the Methods to describe the two methods in maintext: “Using the concatenated super protein sequence alignment, RAxML (v8.2.12) (RAxML, RRID:SCR_006086) was subsequently employed to construct a maximum-likelihood phylogenetic tree with the PROTGAMMALGX (“PROT” means protein sequence, “GAMMA” means gamma distribution, “LG” refers to amino acid substitution model, “X” means maximum likelihood estimation) model [47]. To verify the topology of the ML-tree, a NJ-tree was also constructed using the neighbor joining algorithm in MEGA (X) (MEGA, RRID:SCR_002805) [49].”

In Fig S11, we used another phylogeny building method (NJ, neighbor-joining) to test whether its result is consistent that of ML method (Figure 4B). If the two trees are consistent, the result will be more confident. We have added more description in the supplementary figure legend “The phylogenetic tree was built based on concatenated protein alignment from 6,988 single-copy orthologs (mantises), with NJ (neighbor joining) algorithm in MEGA. The topology of NJ phylogeny tree is largely consistent the ML (maximum likelihood) tree in Figure 4B.”.
In addition, the legend of Fig S13 (now Fig S12) was revised into “The phylogenetic tree was built based on 2201 single-copy orthologs, with maximum likelihood methods (“raxmlHPC -m PROTGAMMALGX”). Dlob: D.lobata, Dtru: D. truncata, Hcor: H. coronatus, Mrel: M. religiosa,Tsin: T. sinensis, Mvio: M. violacea ,Zoot: Z. nevadensis, Bger: B. germanica.”.

- Table S1: References are needed for the karyotype information.

Reply: We have added reference information for karyotype in the legend of Table S1:
“The chromosome number of M. religiosa was obtained from del Cerro’s paper (Synaptonemal complex analysis of the X1X2Y trivalent in Mantis religiosa L. males: inferences on the origin and maintenance of the sex-determining mechanism. Chromosome Research. 1998;6:5-11), while the chromosome number of T. sinensis was obtained from Yuan R’paper (The chromosome-level genome of Chinese praying mantis Tenodera sinensis (Mantodea: Mantidae) reveals its biology as a predator. GigaScience. 2023;12). The chromosome number of D. truncata, H. coronatus, and M. violacea were determined by genome assembly, as no karyotype information was available for these 3 mantis species.”.

Methods
- The kits used for preparing libraries for illumina and RNA sequencing should be added. Also a description of the Hi-C data generation is missing.
Reply: The kits for DNA and RNA sequencing library construction are Truseq DNA Sample Prep Kit (illumina) and Truseq RNA Sample Prep Kit (illumina), respectively. We have added them in the Methods. We also added the Hi-C data generation in Methods “Then, Hi-C data was generated using the same individuals applied for HiFi sequencing. Nuclear DNA was cross-linked by soaking leaf tissues in formaldehyde solution, and the cross-linked genomic DNA was extracted, digested, repaired, ligated to circular fragments, sheared into 350 bp inserts, converted to short-read sequencing library by Truseq DNA

Library Prep Kit, and sequenced on Illumina NovaSeq 6000 platform (RRID:SCR_016387).".

- Please add to line 322 that this analysis is referring to the identification of the X chromosome specifically in T. sinensis
Reply: We have revised "To identify the X chromosome" into "To identify the X chromosome of T. sinensis".

- Please briefly mention your justification of model choice and add the expansion of the RAxML notation of PROTGAMMALGX for those not familiar with this shorthand (i.e. LG + G4 + Fest)
Reply: We have used a relatively older but widely applied version of RAxML (Command "raxmlHPC -m PROTGAMMALGX"), and we added more description about it in Methods:
"PROTGAMMALGX ("PROT" means protein sequence, "GAMMA" means gamma distribution, "LG" refers to amino acid substitution model, "X" means maximum likelihood estimation) model".

- Please note how codon alignment was performed (line 361).
Reply: We have removed this sentence in the revised manuscript.

Suggested rephrasings:
- Valuable repository' with 'valuable resource' (line 35)
Reply: Corrected.

- Missing plural in last sentence of abstract "Mantodea and genetic breeding of efficient enemy insect" (line 37)
Reply: Corrected.

- An "A" or "The" is missing here: "Phylogenetic tree was constructed…" (line 209 and 216)
Reply: "The" was added before "phylogenetic tree" in these two places.

- "breakpoint to less than a 2.5-Mb range" (line 240) I think this should be "breakpoint to a less than 2.5Mb range"
Reply: Corrected.

- I suggest "phylogeny clarification" should be "phylogenetic clarification" (line 247)
Reply: Corrected.

- 'Whose contigs are more fragmental' could be 'whose assembly was fragmented into more contigs' (line 286)
Reply: Corrected.
--
Please also take a moment to check our website at for any additional comments that were saved as attachments. Please note that as GigaScience has a policy of open peer review, you will be able to see the names of the reviewers.

________________________________________

In compliance with data protection regulations, you may request that we remove your personal registration details at any time. (Use the following URL: https://www.editorialmanager.com/giga/login.asp?a=r). Please contact the publication office if you have any questions.

Editorial Manager®

## Cookie Preference Center

We use cookies which are necessary to make our site work. We may also use additional cookies to analyze, improve and personalize our content and your digital experience. For more information, see our Cookie Policy.

You may choose not to allow some types of cookies. However, blocking some types may impact your experience of our site and the services we are able to offer. See the different category headings below to find out more or change your settings.

Allow all

## Manage Consent Preferences

### Strictly Necessary Cookies

**Always active**

These cookies are necessary for the website to function and cannot be switched off in our systems. They are usually only set in response to actions made by you which amount to a request for services, such as setting your privacy preferences, logging in or filling in forms. You can set your browser to block or alert you about these cookies, but some parts of the site will not then work.

### Functional Cookies

These cookies enable the website to provide enhanced functionality and personalisation. They may be set by us or by third party providers whose services we have added to our pages. If you do not allow these cookies then some or all of these services may not function properly.

### Performance Cookies

These cookies allow us to count visits and traffic sources so we can measure and improve the performance of our site. They help us to know which pages are the most and least popular and see how visitors move around the site.

**Targeting Cookies**

These cookies may be set through our site by our advertising partners. They may be used by those companies to build a profile of your interests and show you relevant adverts on other sites. If you do not allow these cookies, you will experience less targeted advertising.

### Cookie List

Clear

Apply Cancel
Consent Leg.Interest

Confirm my choices

Powered by onetrust
