# Supplementary material for: The genomes of 5 mantises provide insights into sex chromosome evolution and Mantodea phylogeny clarification
Source: Gigascience. 2025 Dec 18;15:giaf158. doi: 10.1093/gigascience/giaf158 (PMC12908712; doi:10.1093/gigascience/giaf158)
Supplement: giaf158_Authors_Response_To_Reviewer_Comments_Revision_1 [file giaf158_authors_response_to_reviewer_comments_revision_1.pdf]

Author's Response To Reviewer Comments

Reviewer reports:

Reviewer #1: Thank you for your efforts in addressing the comments and suggestions from the first round of review. I have carefully reviewed the revised manuscript and am pleased with the revisions made.

Reply: Thanks.

Reviewer #2: I thank the authors for addressing some of my previous comments. However, a number of issues remain as discussed below.

Major comments:
There is no reason why a slow rate of substitution should partly explain the morphological resemblance of M. violacea to cockroaches as substitution rates are independent of morphological evolution. Therefore, this interpretation should be removed throughout e.g. "Low evolutionary rate may allow M. violacea conserve more ancestral traits" (line 228) and "the mutation rate of M. violacea genome is the lowest among all the mantises, consistent with its retention of ancestral traits" (line 288)

Reply: According to the reviewer's suggestion, we removed our conclusion on substitution rate and morphological evolution. The subtitle "Low evolutionary rate may allow M. violacea conserve more ancestral traits" was changed into "Mantodea phylogeny provide taxonomic evidences for D. truncata". The sentence "With a slower evolution rate, M. violacea may preserve more characteristics of the Mantodea ancestor, which also partly explains its morphological resemblance to cockroaches." was removed. The discussion "and the mutation rate of M. violacea genome is the lowest among all the mantises, consistent with its retention of ancestral traits" was also removed.

The comment "Generally, the branch length is in proportional to the amino-acid substitution rate." is also incorrect. Branch lengths in an undated tree are the number of substitutions per site in the sequence alignment. The number of substitutions is determined by the substitution rate and time since divergence. A long branch can be due to a low substitution rate but long divergence time and vice versa. In a dated phylogeny, branch lengths show the amount of time. Moreover, the authors also still do not explain how substitution rates were inferred. I am also not sure if substitution rates can be reliably inferred from a tree which is dated with MEGA as I do not think this method takes into account rate heterogeneity. An explanation of how substitution rates were inferred should be added, if this analysis is kept.

Reply: We agree with the reviewer. The old description "Generally, the branch length is in proportional to the amino-acid substitution rate. The amino-acid substitution rate for M. violacea branch was the lowest among those of Mantodea (Figure 4B), which may indicate that the evolution rate of M. violacea branch was much slower than that of the other mantises." was revised into "From the phylogenetic tree, we observed that the branch length from the Mantodea node to the M. violacea node is much shorter than those from the Mantodea node to the species nodes of other mantises (Figure 4B). Branch length in an undated tree represents the number of substitutions per site in the sequence alignment. Thus, the shorter branch length of M. violacea means fewer substitutions accumulated from Mantodea ancestor to M. violacea.".

While the authors now mention the method of dating in the Figure 4 legend, the dating method is not included in the methods section. This should be added.

Reply: The dating method was added in Methods section: "The time tree was inferred using the Reltime-Branch lengths method in MEGA, with input of user-supplied branch lengths derived from Maximum Likelihood (ML) method. The time tree was computed using 1 calibration constraint (70-80 Mya between M.violacea and M.religiosa, doi: 10.1126/science.1257570)."
To be not duplicated, the legend of Figure 4 was shortened into "(C) Time tree inferred by the Reltime-

Branch lengths method in MEGA, with 1 calibration constraint (70-80 Mya between M.violacea and M.religiosa).”

Minor comments:

Thank the authors for removing the mention of sex determination in the title. The new title ("The genomes of five mantises provide insights into evolution of sex chromosome and Mantodea lineages") should be made more specific as "provide insights into Mantodea lineages" is vague. As currently written, 'sex chromosome' should be plural.

Reply: “evolution of sex chromosome and Mantodea lineages” was revised into “sex chromosome evolution and Mantodea phylogeny clarification”.

The explanation of the results and questions raised by phylogenetic studies could still be improved. As it reads, it is very brief and could be expanded to set up the questions explored in the paper more. Please also expand the explanation of why studying mantis sex chromosomes is important.

Reply: Thanks for the reviewer’s kind expectation, but it is out of our interest and ability to perform more deeper phylogenetic analysis in this study. According to the reviewer’s suggestion, we added a sentence in the maintext “Studying mantis sex chromosomes will uncover the formation mechanism underling the X1X2Y type.”.

Please also change "most manties have the common XY sex determination system" to "most manties have XY sex chromosomes" (line 59) as the observation of sex chromosomes alone does not indicate the sex determination mechanism.

Reply: Corrected.

If I understand the responses correctly, the photos in Figure 1 correspond to the specimens that were sequenced. If this is the case, then it should be explained in the legend of the figure that the images are of the sequenced specimens.

Reply: We added a sentence “The images of the sequenced specimens were shown in the center of circos plots.” in the legend of Figure 1.

I thank the authors for adding some detail for how divergence times were estimated. However, there is no explanation for why the calibration constraint of 70-80 mya between M.violacea and M.religiosa was chosen. This needs justification.

Reply: The calibration data for Mantodea is quite few. We got the calibration between M.violacea and M.religiosa from a recently published paper in Science: Phylogenomics resolves the timing and pattern of insect evolution (doi: 10.1126/science.1257570). This study inferred the phylogeny and dating time for major insect lineages from 1478 protein-coding genes, which were constructed from transcriptome data. We thought this is the most accurate estimation of dating time between M.violacea and M.religiosa in the world till now, so we chose it.

The authors now note in the discussion that taxon sampling was a major limitation in the phylogenomic analyses. It should also be highlighted that other phylogenomic approaches should also be used, including coalescent based approaches that are better suited for exploring lineages that quickly diversified than concatenation which was used here.

Reply: In this study, we have adopted a traditionally widely-adopted concatenation method for phylogeny construction. We added a discussion in the maintext: “In addition, more advanced phylogenomic methods such as coalescent based approaches may improve the accuracy of phylogenic inference for lineages that quickly diversified.”

I thank the authors for explaining why they used EndHiC instead of YaHS for some assemblies. This explanation should be added to the methods to make this clear to readers.

Reply: We added a sentence in Methods: "YaHS is capable to construct chromosome-level scaffolds with relatively shorter contigs, while EndHiC is more suitable for relatively larger contigs.".

As the genome statistics which are given in long lists are in Table 1 there is no reason for these to be repeated in full in the main text. Please do use ranges and refer to the table rather than reporting the information for each species. This makes the text unnecessarily verbose and harder to read.

Reply: Corrected.

The explanation of why the genomes of H. coronatus differ between studies is still unclear. Please could the authors clarify if they manually curated the X chromosomes and add whether they did this or not to the methods. Documenting manual curation is extremely important for reproducibility. If manual curation was performed, the total number of manual breaks, joins and removals of sequence should be stated. Please also mention in the main text whether the difference in assembly in the X chromosome was due to the use of curation, or if not why a single fragment was produced in the current version while previously it was in 3 fragments.

Reply: There is no manual curation in our HiC scaffolding, so we did not mention it in the Methods. The reasons may be: we used Hifi sequencing, thus our contig N50 length is much longer, and we used EndHiC, which is more powerful in building chromosome-level scaffolds than other software.
We added an explanation sentence in the maintext: "With much longer assembled contigs and EndHiC that is specially designed for scaffolding large contigs, we achieved a complete assembly of the X chromosome for H. coronatus.".

I thank the author for adding the correlation of genome size and repeat content to the main text. However, it is essential that the statistical test takes into account phylogenetic non-independence between taxa. Please use a statistical approach that does this e.g. phylogenetic generalised least squares regression.

Reply: According to the reviewer's suggestion, we changed to use phylogenetic generalised least squares regression. We added the result in Figure 2A legend: "Phylogenetic Generalised Least Squares Regression (PGLS) was applied, resulting in $R=0.954$ and $p = 0.008 < 0.01$".

I thank the authors for adding in the example I gave of B chromosomes, however how the sentence is currently written gives the impression that the list is exhaustive. Please either add other ways in which sex chromosomes evolve or else make it clear that just a couple of examples are listed.

Reply: The sentence "Sex chromosomes evolved from autosomes or B chromosomes" was revised into "Sex chromosomes generally evolved from autosomes and occasionally from B chromosomes".

"The cockroach-like body morphology was thought to be the ancestral traits of Mantodea (order)." (line 241). This should be supported by a reference(s).

Reply: We added a reference paper: "Hornig MK, Haug JT and Haug C. An exceptionally preserved 110 million years old praying mantis provides new insights into the predatory behaviour of early mantodeans. PeerJ. 2017;5:e3605. doi:10.7717/peerj.3605.".

The description of the divergence time estimation (line 255) should come at the start of the paragraph rather than the end as this is what allowed rates to be examined.

Reply: We have removed the analysis of substitution rate, so it does not need to change the placement for

divergence time estimation.

"We used HiFi long reads with over 99% accuracy, while they used common long reads with only ~85% Accuracy."." - please could you explain what "common long reads" are. PacBio HiFi sequencing is a frequently used long-read sequencing method so I'm not sure what the authors are referring to. To avoid ambiguity, please be specific. I am also unsure why technology itself makes a big difference - instead it's likely due to differences in average read length, is this the case?

Reply: PacBio sequencing can generate one-pass reads with 85% base accuracy, and multi-pass reads with 99% base accuracy (HiFi reads). Multi-pass means sequencing a DNA fragment several times, and finally call a consensus from a multiple-sequence alignment. The read length for one-pass and multiple-pass has no difference. To be specific, we changed “common long reads” into “one-pass long reads”.

The sentence on why Mantodea occupies "an important position in the evolution of insects" (line 280) still needs to be clearer. Please elaborate.

Reply: This sentence was revised into “Mantodea has occupied an important position in the evolution of insects, with distinctive morphology, camouflage behavior, and unusual sex determination mechanism.”.

This needs further detail e.g. is 4,014 referring to the number of single-copy OGs in all mantises and outgroups? "From gene family clustering, 69,603 orthologous groups (OGs) were generated, including 4,014 single-copy OGs."

Reply: Yes. The orthogroups were constructed using genes from all the mantises. Each orthogroup contains genes of all mantises derived from a single gene in their latest common ancestor. Single-copy OG means that the OG contain only one gene for each mantis.

The sentence was revised into “From gene family clustering, 69,603 orthologous groups (OGs) were generated for all the analyzed species, including 4,014 single-copy OGs in which each mantis must be present and have only a single gene.”.

The description of the radar chart in figure 2A is still insufficient. Please explicitly state what a higher vertex means and the corresponding percentages - e.g. are these the percentage of genome occupied by TEs, or total percentage of TEs corresponding to a particular TE type?

Reply: They are the percentage of genome occupied by TEs. We have added a sentence in Figure 2A legend: “The percentage for each height level of the vertex represents the percentage of genome occupied by TEs.”.

Figure 3B: The colours are still not correctly tracking between the chromosomes of each species. E.g. chr 1 of T. sinensis is pink to me while the homologous chromosomes in D. truncata is orange. This should be fixed. The legend describes "grep color". I'm not familiar with what this is.

Reply: We’d like to explain this misunderstanding. The color for synteny links between each homologous chromosome in different species were automatically determined by the MCScanX software. The color was randomly assigned, just to be different among various homologous chromosomes, so the users can identify the synteny blocks easily. It was difficult for us to modify the color for each syntenic link.
We used black box to represent sex chromosome, and use gray box to represent autosome chromosome. The sentence “For B-E, black color represents for X chromosomes, while gray color represents for autosomes” was revised into “For subfigures B-E, black box represents for X chromosomes, while gray box represents for autosomes.”.

The legend also has an unclear sentence "Much more chromosome rearrangements were observed between M. violacea and the other mantises". Please revise this to make the message clearer.

Reply: This sentence was revised into “Much more chromosome rearrangements were observed between M. violacea and D. truncata, in comparison to those between T. sinensis and D. truncata.”.

Figure 4: The y-axis is just 'percentage'. Please elaborate what the percentage is of.
The figure title for Figure 4 "Evolution of Mantodea lineages" is very vague, please make it specific such that it summarises the key message of the figure.

Reply: “percentage” was revised into “Percent of OG”, and "Evolution of Mantodea lineages” was revised into “Orthologous groups and phylogeny of Mantodea”.

With regards to adding the identified sex chromosomes and the sex that the genome is derived from to Table 1. I understand that the authors do not add more rows to Table 1. suggest instead adding this information within the existing rows e.g. "# of assembly-inferred chromosomes" could be 13A + 1Z rather than 14, and adding the sex sequenced in brackets next to the species name in the header row, thus conveying this information without adding more rows.

Reply: Thanks for the reviewer’s suggestion. We have changed the chromosome number into “12+X1+X2 12+X1+X2 15+X 20+X 16+X” in Table 1, and added a note under this table : “# of assembly-inferred chromosomes” refers to autosome and sex chromosome number in female.

Figure S11: "The topology of NJ phylogeny tree is largely consistent the ML (maximum likelihood) tree in Figure 4B.". The topology appears the same to me between these two figures. Suggest removing this figure and just saying that the same topology was obtained by NJ in the main text as the result is the same.

Reply: According to the reviewer’ suggestion, Figure S11 was removed. In addition, we added a sentence in the maintext: “and the same topology was obtained by NJ method,”.

Typos and suggested rephrasing:

Line 152: I do not think it's typical to put the taxonomic levels in brackets next to each taxon. Perhaps rephrase this e.g. "Currently the orders of Mantodea and Blattodea are placed within the superorder Dictyoptera"

Reply: Corrected.

Line 122: typo - "manily" → "mainly"

Reply: Corrected.

"using reciprocal-best orthologous genes" - should this be "reciprocal best hit". Please change in all instances.

Reply: We have changed all “reciprocal-best orthologous genes” into “reciprocal best hits”.

Line 500: Suggest changing "4 chromosome brokens" to "4 chromosome breaks"

Reply: Corrected.

Ideally a single textface should be used in figures. Some figures use multiple text faces.

Reply: We have revised the textface in all figures to make them consistent.

Editorial Manager®

Cookie Preference Center

We use cookies which are necessary to make our site work. We may also use additional cookies to analyze, improve and personalize our content and your digital experience. For more information, see our Cookie Policy.

You may choose not to allow some types of cookies. However, blocking some types may impact your experience of our site and the services we are able to offer. See the different category headings below to find out more or change your settings.

Allow all

Manage Consent Preferences

Strictly Necessary Cookies

Always active

These cookies are necessary for the website to function and cannot be switched off in our systems. They are usually only set in response to actions made by you which amount to a request for services, such as setting your privacy preferences, logging in or filling in forms. You can set your browser to block or alert you about these cookies, but some parts of the site will not then work.

Functional Cookies

These cookies enable the website to provide enhanced functionality and personalisation. They may be set by us or by third party providers whose services we have added to our pages. If you do not allow these cookies then some or all of these services may not function properly.

Performance Cookies

These cookies allow us to count visits and traffic sources so we can measure and improve the performance of our site. They help us to know which pages are the most and least popular and see how visitors move around the site.

Targeting Cookies

These cookies may be set through our site by our advertising partners. They may be used by those companies to build a profile of your interests and show you relevant adverts on other sites. If you do not allow these cookies, you will experience less targeted advertising.

Cookie List

Clear

Apply Cancel
Consent Leg.Interest

Confirm my choices

Powered by onetrust
