# Supplementary material for: The genomes of 5 mantises provide insights into sex chromosome evolution and Mantodea phylogeny clarification
Source: Gigascience. 2025 Dec 18;15:giaf158. doi: 10.1093/gigascience/giaf158 (PMC12908712; doi:10.1093/gigascience/giaf158)
Supplement: giaf158_Reviewer_1_Report_original_submission — Xue-xin Chen -- 9/10/2025 [file giaf158_reviewer_1_report_original_submission.pdf]

**Reviewer Report**

**Title: The genomes of five mantises provide insights into sex chromosome evolution and Mantodea phylogeny clarification**

**Version: Original Submission** **Date:** 9/10/2025

**Reviewer name: Xue-xin Chen**

**Reviewer Comments to Author:**

This manuscript presents chromosome-scale genomes for five mantis species, comparative repeat analyses, inferences about sex-chromosome evolution (X1/X2 vs. single X), rate heterogeneity (slow lineage in Metallyticus), and phylogenetic placement of Deroplatys. The datasets are potentially valuable; however, taxonomy/terminology, framing, and several core claims (especially on sex chromosomes) require substantial revision and additional detail.

Major comments

1) Taxonomic terminology and hierarchy (must fix throughout)

The usage of order/superfamily/family is repeatedly incorrect or inconsistent. Mantidae is a family, not a superfamily. Please audit the entire manuscript (title/abstract/main text/figures/legends/keywords) and ensure consistent, current placement within order Mantodea and the Dictyoptera clade. Add a short taxonomic note or cite an up-to-date checklist.

2) Abstract

Add key numbers to convey scale/quality (e.g., genome sizes ~2.3-4.3 Gb; contig/scaffold N50; % of sequence anchored to chromosomes; BUSCO range).

Replace unclear wording:

"genetic breeding of efficient enemy insect" â†' "genetic improvement and breeding of beneficial biological control agents."

Phylogeny statement is vague. State the concrete relationship (e.g., Deroplatys truncata sister to Hymenopus coronatus) and soften to "helps resolve" rather than "providing important evidences for resolving."

3) Introduction

The Introduction does not prepare readers for the sex-chromosome focus and underspecifies outstanding phylogenetic issues. Please:

(i) explicitly flag unresolved nodes your genomes will test (e.g., placement of Deroplatys; early branching of Metallyticus);

(ii) briefly introduce known mantis karyotype diversity and why X1X2 vs. single X matters;

(iii) trim peripheral material and end with a clear statement of aims/hypotheses.

4) Methods

Species identification: describe how each sampled species was confirmed (diagnostic morphology, DNA barcoding), voucher numbers, and deposition.

Biological replicates: clearly state how many male and female individuals were sequenced per species (genome and transcriptome). If single-individual per sex was used, acknowledge this limitation explicitly.

Transcriptome tissues: two "midfoot" entries appear; the latter likely means hindfootâ€"please correct.
P316: "for each mantis, the protein sequences from the other 4 mantises were …"â€"list the four species explicitly to remove ambiguity. (maybe 5 mantises)
5) X-chromosome identification and strength of inference
Depth-based identification in T. sinensis is reasonable, but provide normalized male/female coverage ratios (e.g., CQ) with sliding-window plots, variance/CI, and GC/repeat normalization; specify the number of replicates per sex. For the other genera, assignments based on comparative synteny should be described as inference, not definitive identification.
Avoid "confirmed"; use "supports a model in which …" and separate plausible scenarios (fusion vs. fission vs. translocation). Acknowledge limited taxon sampling (two Mantidae spp.).
6) Breakpoint localization claim (~2.5 Mb)
Provide methods and support: genomic coordinates, anchor density, long-read overlaps across the putative break, and Hi-C contact changes; include an uncertainty interval. Clarify whether this interval is species-specific or an inference for the Mantidae ancestor.
7) Heterozygosity vs. contiguity (discussion too thin)
The statement that lower contiguity in M. religiosa/T. sinensis is due to higher heterozygosity needs numbers (k-mer heterozygosity estimates and spectra) and clarification on phasing/purge_dups/trio-binning. Report exact contig N50/N90 and % duplication before/after purging. Expand the discussion with appropriate citations.
8) Phylogeny and rate heterogeneity
Define the substitution-rate analysis (loci, clock model, calibrations, tests of rate constancy, CIs).
Rephrase the teleological sentence: instead of "allowing M. violaceus to conserve more ancestral traits," use "consistent with retention of ancestral gene content/signals."
The statement that M. violaceus "shares more orthologous genes with cockroaches" requires a precise metric (e.g., number of 1:1 orthologs from OrthoFinder; control for annotation size).
For the Deroplatys placement, report support (UFBoot/SH-aLRT/ASTRAL LPP; gene/site-concordance factors) and briefly discuss potential discordance (ILS/introgression) given rapid radiations.
9) The manuscript requires a thorough language edit by a fluent scientific editor.

**Methods**

Are the methods appropriate to the aims of the study, are they well described, and are necessary controls included? Choose an item.

**Conclusions**

Are the conclusions adequately supported by the data shown? Choose an item.

**Reporting Standards**

Does the manuscript adhere to the journal's guidelines on [minimum standards of reporting?]() Choose an item.

Choose an item.

**Statistics**

Are you able to assess all statistics in the manuscript, including the appropriateness of statistical tests used? Choose an item.

**Quality of Written English**

Please indicate the quality of language in the manuscript: Choose an item.

**Declaration of Competing Interests**

Please complete a declaration of competing interests, considering the following questions:

- Have you in the past five years received reimbursements, fees, funding, or salary from an organisation that may in any way gain or lose financially from the publication of this manuscript, either now or in the future?
- Do you hold any stocks or shares in an organisation that may in any way gain or lose financially from the publication of this manuscript, either now or in the future?
- Do you hold or are you currently applying for any patents relating to the content of the manuscript?
- Have you received reimbursements, fees, funding, or salary from an organization that holds or has applied for patents relating to the content of the manuscript?
- Do you have any other financial competing interests?
- Do you have any non-financial competing interests in relation to this paper?

If you can answer no to all of the above, write 'I declare that I have no competing interests' below. If your reply is yes to any, please give details below.

I declare that I have no competing interests.

I agree to the open peer review policy of the journal. I understand that my name will be included on my report to the authors and, if the manuscript is accepted for publication, my named report including any attachments I upload will be posted on the website along with the authors' responses. I agree for my report to be made available under an Open Access Creative Commons CC-BY license (http://creativecommons.org/licenses/by/4.0/). I understand that any comments which I do not wish to be included in my named report can be included as confidential comments to the editors, which will not be published.

Choose an item.

To further support our reviewers, we have joined with Publons, where you can gain additional credit to further highlight your hard work (see: https://publons.com/journal/530/gigascience). On publication of this paper, your review will be automatically added to Publons, you can then choose whether or not to claim your Publons credit. I understand this statement.

Yes Choose an item.
