# Supplementary material for: The genomes of 5 mantises provide insights into sex chromosome evolution and Mantodea phylogeny clarification
Source: Gigascience. 2025 Dec 18;15:giaf158. doi: 10.1093/gigascience/giaf158 (PMC12908712; doi:10.1093/gigascience/giaf158)
Supplement: giaf158_Reviewer_2_Report_original_submission — Charlotte Julie Wright, MPhil -- 9/21/2025 [file giaf158_reviewer_2_report_original_submission.pdf]

Reviewer Report

Title: The genomes of five mantises provide insights into sex chromosome evolution and Mantodea phylogeny clarification

Version: Original Submission **Date:** 9/21/2025

Reviewer name: Charlotte Julie Wright, MPhil

Reviewer Comments to Author:

The manuscript presents chromosome-level genome assemblies for five species of mantis, describing their patterns of synteny and phylogenetic relationships. Most of the conclusions are well supported and the manuscript well written. The genomes will serve as a useful resource for future ecological and evolutionary studies in this group and beyond. Overall, I recommend it for publication in GigaScience. However, several aspects need to be addressed first.

Major comments:

- The title needs to be changed as while the manuscript describes the evolution of additional sex chromosomes, there is no investigation into sex determination mechanisms. E.g. replace 'sex determination mechanism evolution' with 'sex chromosome evolution'.
- Without dating the tree, substitution rates and time are confounded, preventing the authors from making comments on substitution rates. Moreover, the large distances of evolutionary time between lineages make inferring substitution rates extremely hard. These claims should therefore be revised.
- As the level of confidence in the phylogeny was not explored, the discussion should make it clear that more detailed phylogenomic analyses are required to get at some of the interesting questions that they would like to investigate in future.
- The generation of the Hi-C data is missing from the methods.
- It is unclear to me why a different method for scaffolding was used for the genomes with fewer contigs. Please explain why the protocol used for the other genomes (the Arima mapping pipeline and YaHS) would not have been appropriate.

Minor comments

- The supplementary contains figures (e.g. figure S10-12) and includes methods that are not mentioned in the main text. Please make the supplementary consistent with the main text and adapt numbering accordingly.

Abstract:

- "Metallyticus violacea" should be changed to "Metallyticus violacea" as this version is used everywhere else in the text and used in NCBI's taxonomy.
- "deduced that translocations between" should be changed to "suggest that translocations between" as the data suggests a translocation but does not prove it.
- The term 'Deroplatys' is used in the abstract without contextualising what this group is, please briefly describe what this is for readers unfamiliar with the taxonomy. This is also the case in the introduction.

Introduction:

- Please explain in the introduction the significance/impact of understanding the relationships within

Mantodea.
Results:
- The results list useful statistics for each species (lines 79-96). However, this information would be much better suited to a table to allow comparison between species and statistics. Ranges in the results could suffice to summaries the table. It is important for downstream analyses of sex chromosomes that females were sequenced (i.e. lacked any Y chromosomes).
- Please briefly explain the GCE method in the text for those less familiar with this.
- When comparing the genomes of H. coronatus from this study and from another study, please explain why the X chromosome is better assembled here. For example, was manual curation performed and does this explain the difference in quality?
- Given that the authors describe a strong correlation between genome size and repeat content, I suggest adapting Fig S7 which plots these two variables as a graph as part of Fig1/2, adding a statistical test for the strength of correlation while accounting for shared ancestry (i.e. including the effect of phylogeny when testing for correlation) to verify this relationship.
- The authors write "Sex chromosomes evolved from autosomes and play important roles in tissue development, mating, and speciation [17-19]." which is not quite true, sex chromosomes can also evolve by other means e.g. from B chromosomes. Please revise this.
- It would be valuable to add a short description of the types of chromosome rearrangements that are suggested to have occurred in these genomes (based on the synteny plot), to shape the varying karyotypes of 2n=14-21. At the moment, only sex chromosomes are described but the autosomes have also experienced changes.
The statements about sex chromosome systems across insects need supporting with references
- When describing the sex chromosome result of T. sinensis it should be mentioned that the genome was generated from a female (i.e. lacked any Y chromosomes) and hence two half coverage chromosomes in male are two Xs (rather than X/Y for example).
- Some claims are too speculative based on the available data e.g. "M. violaceus shares much more OGs with cockroaches than the other 4 species (69% vs 61-63%) (Figure 4A), which may partly explain its strong morphological resemblance to cockroaches." (line 208) and similarly in the discussion "M. violaceus genome shares more orthologous genes with cockroaches than with the other mantises, and the mutation rate of M. violaceus genome is the lowest among all the mantises, allowing M. violaceus to conserve more ancestral traits." (line 244). This is also reflected in the title of this section being too strong "Low evolutionary rate allows M. violaceus conserve more ancestral traits" (line 194). Also, the authors do not describe the traits of M. violaceus which are "more ancestral".
- It would strengthen this section if the authors could assess the confidence in the assignment of D. truncata as sister to H. coronatus, or else make it clear that further analyses are needed.
- Please comment on why the N50 and N90 are so much higher in your assemblies than in previously published genomes e.g. differences in sequencing technology, assembly methods etc (line 228).
- Heterozygosity is mentioned in a few places without being supported by any values (e.g. line 231)
- Please could you clarify what you mean by "tissues with no or low heterozygosity are still a priority for de novo genome sequencing." and make it clearer that high heterozygosity can be problematic for genome assembly (line 232)
- Please can you elaborate why Mantodea occupies "an important position in the evolution of insects"

(line 235)
Comments on figures:
- Fig1F: the category of duplicated BUSCOs are missing, please add this. This also needs a Y-axis label.
- Fig2A: Please add a short description of how to interpret a radar chart and the main conclusion it supports.
- Fig 2: As all panels have the same key I suggest having it just once and making it clear it applies to all panels in the figure legend. Please make the Y-axis in panels B,C and D more informative (i.e. counts of what)
- Fig 3: The 'X chromosome' in the title should be plural given more than one evolved.
- Fig 3A: Please add an Y-axis label.
- Fig 3B-D: Please include details about the orthologs that are plotted i.e. total number per plot and what these orthologs are e.g. all single copy orthologues in both species.
- Fig3B: The colouring of T. sinensis is strange. It would be helpful if X1 is the same colour as X to indicate shared orthologs.
- Fig 3E: Please annotate the figure with the node where the hypothesised translocation occurred and label the resulting chromosomes as X chromosomes. Black should be changed to 'X1-chromosome' given that an X2 is made in the figure but is half grey half black due to it being part ancestral autosome and part sex chromosome.
- Fig 3F: Please increase the space between the key for gene number and that for TR and TE as it is hard to understand at present. Please also explain in the figure legend what TR and TE denote and their relevance to the breakpoint. It would also be helpful to note in the legend how this figure illustrates how the breakpoint was identified.
- Fig 4: I think that the figure legend title ("Orthologous groups and phylogeny of Mantodea") could be more informative. Also please clarify whether the following is referencing all OG groups in cockroaches that are found in each mantis, or that of mantis OGs found in cockroaches: "Percentage of orthologous groups (OG) shared with cockroach for each mantis species". Please add the substitution model used in maximum-likelihood inference to the legend and add the scale that the branch lengths are given in.
- Table 1: It would be helpful to add identified sex chromosomes and the sex that the genome is derived from.
- Fig S7: Please add more details to the legend e.g. which model was used in maximum-likelihood (ML). Also, in the methods, two ML approaches are described while this figure shows one tree from ML and one from NJ. Please ensure the figure and methods are consistent. Also the case for Fig S13.
- Table S1: References are needed for the karyotype information.
Methods
- The kits used for preparing libraries for illumina and RNA sequencing should be added. Also a description of the Hi-C data generation is missing.
- Please add to line 322 that this analysis is referring to the identification of the X chromosome specifically in T. sinensis
- Please briefly mention your justification of model choice and add the expansion of the RAxML notation of PROTGAMMALGX for those not familiar with this shorthand (i.e. LG + G4 + Fest)
- Please note how codon alignment was performed (line 361).
Suggested rephrasings:

- Valuable repository' with 'valuable resource' (line 35)
- Missing plural in last sentence of abstract "Mantodea and genetic breeding of efficient enemy insect" (line 37)
- An "A" or "The" is missing here: "Phylogenetic tree was constructed…" (line 209 and 216)
- "breakpoint to less than a 2.5-Mb range" (line 240) I think this should be "breakpoint to a less than 2.5Mb range"
- I suggest "phylogeny clarification" should be "phylogenetic clarification" (line 247)
- 'Whose contigs are more fragmental' could be 'whose assembly was fragmented into more contigs' (line 286)

Methods

Are the methods appropriate to the aims of the study, are they well described, and are necessary controls included? Choose an item.

Conclusions

Are the conclusions adequately supported by the data shown? Choose an item.

Reporting Standards

Does the manuscript adhere to the journal's guidelines on minimum standards of reporting? Choose an item.

Choose an item.

Statistics

Are you able to assess all statistics in the manuscript, including the appropriateness of statistical tests used? Choose an item.

Quality of Written English

Please indicate the quality of language in the manuscript: Choose an item.

Declaration of Competing Interests

Please complete a declaration of competing interests, considering the following questions:

- Have you in the past five years received reimbursements, fees, funding, or salary from an organisation that may in any way gain or lose financially from the publication of this manuscript, either now or in the future?
- Do you hold any stocks or shares in an organisation that may in any way gain or lose financially from the publication of this manuscript, either now or in the future?

- Do you hold or are you currently applying for any patents relating to the content of the manuscript?
- Have you received reimbursements, fees, funding, or salary from an organization that holds or has applied for patents relating to the content of the manuscript?
- Do you have any other financial competing interests?
- Do you have any non-financial competing interests in relation to this paper?

If you can answer no to all of the above, write 'I declare that I have no competing interests' below. If your reply is yes to any, please give details below.

I declare that I have no competing interests

I agree to the open peer review policy of the journal. I understand that my name will be included on my report to the authors and, if the manuscript is accepted for publication, my named report including any attachments I upload will be posted on the website along with the authors' responses. I agree for my report to be made available under an Open Access Creative Commons CC-BY license (http://creativecommons.org/licenses/by/4.0/). I understand that any comments which I do not wish to be included in my named report can be included as confidential comments to the editors, which will not be published.

Choose an item.

To further support our reviewers, we have joined with Publons, where you can gain additional credit to further highlight your hard work (see: https://publons.com/journal/530/gigascience). On publication of this paper, your review will be automatically added to Publons, you can then choose whether or not to claim your Publons credit. I understand this statement.

Yes Choose an item.
