# Supplementary material for: The genomes of 5 mantises provide insights into sex chromosome evolution and Mantodea phylogeny clarification
Source: Gigascience. 2025 Dec 18;15:giaf158. doi: 10.1093/gigascience/giaf158 (PMC12908712; doi:10.1093/gigascience/giaf158)
Supplement: giaf158_Reviewer_2_Report_revision_1 — Charlotte Julie Wright, MPhil -- 10/24/2025 [file giaf158_reviewer_2_report_revision_1.pdf]

Reviewer Report

Title: The genomes of five mantises provide insights into sex chromosome evolution and Mantodea phylogeny clarification

Version: Revision 1 **Date:** 10/24/2025

Reviewer name: Charlotte Julie Wright, MPhil

Reviewer Comments to Author:

I thank the authors for addressing some of my previous comments. However, a number of issues remain as discussed below.
Major comments:
There is no reason why a slow rate of substitution should partly explain the morphological resemblance of M. violacea to cockroaches as substitution rates are independent of morphological evolution. Therefore, this interpretation should be removed throughout e.g. "Low evolutionary rate may allow M. violacea conserve more ancestral traits" (line 228) and "the mutation rate of M. violacea genome is the lowest among all the mantises,consistent with its retention of ancestral traits" (line 288)
The comment "Generally, the branch length is in proportional to the amino-acid substitution rate." is also incorrect. Branch lengths in an undated tree are the number of substitutions per site in the sequence alignment. The number of substitutions is determined by the substitution rate and time since divergence. A long branch can be due to a low substitution rate but long divergence time and vice versa. In a dated phylogeny, branch lengths show the amount of time. Moreover, the authors also still do not explain how substitution rates were inferred. I am also not sure if substitution rates can be reliably inferred from a tree which is dated with MEGA as I do not think this method takes into account rate heterogeneity. An explanation of how substitution rates were inferred should be added, if this analysis is kept.
While the authors now mention the method of dating in the Figure 4 legend, the dating method is not included in the methods section. This should be added.
Minor comments:
Thank the authors for removing the mention of sex determination in the title. The new title ("The genomes of five mantises provide insights into evolution of sex chromosome and Mantodea lineages") should be made more specific as "provide insights into Mantodea lineages" is vague. As currently written, 'sex chromosome' should be plural.
The explanation of the results and questions raised by phylogenetic studies could still be improved. As it reads, it is very brief and could be expanded to set up the questions explored in the paper more.Please also expand the explanation of why studying mantis sex chromosomes is important.
Please also change "most manties have the common XY sex determination system" to "most manties have XY sex chromosomes" (line 59) as the observation of sex chromosomes alone does not indicate the sex determination mechanism.
If I understand the responses correctly, the photos in Figure 1 correspond to the specimens that were sequenced. If this is the case, then it should be explained in the legend of the figure that the images are

of the sequenced specimens.
I thank the authors for adding some detail for how divergence times were estimated. However, there is no explanation for why the calibration constraint of 70-80 mya between M.violacea and M.religiosa was chosen. This needs justification.
The authors now note in the discussion that taxon sampling was a major limitation in the phylogenomic analyses. It should also be highlighted that other phylogenomic approaches should also be used, including coalescent based approaches that are better suited for exploring lineages that quickly diversified than concatenation which was used here.
I thank the authors for explaining why they used EndHiC instead of YaHS for some assemblies. This explanation should be added to the methods to make this clear to readers.
As the genome statistics which are given in long lists are in Table 1 there is no reason for these to be repeated in full in the main text. Please do use ranges and refer to the table rather than reporting the information for each species. This makes the text unnecessarily verbose and harder to read.
The explanation of why the genomes of H. coronatus differ between studies is still unclear. Please could the authors clarify if they manually curated the X chromosomes and add whether they did this or not to the methods. Documenting manual curation is extremely important for reproducibility. If manual curation was performed, the total number of manual breaks, joins and removals of sequence should be stated. Please also mention in the main text whether the difference in assembly in the X chromosome was due to the use of curation, or if not why a single fragment was produced in the current version while previously it was in 3 fragments.
I thank the reviewer for adding the correlation of genome size and repeat content to the main text. However, it is essential that the statistical test takes into account phylogenetic non-independence between taxa. Please use a statistical approach that does this e.g. phylogenetic generalised least squares regression.
I thank the authors for adding in the example I gave of B chromosomes, however how the sentence is currently written gives the impression that the list is exhaustive. Please either add other ways in which sex chromosomes evolve or else make it clear that just a couple of examples are listed.
"The cockroach-like body morphology was thought to be the ancestral traits of Mantodea (order)." (line 241). This should be supported by a reference(s).
The description of the divergence time estimation (line 255) should come at the start of the paragraph rather than the end as this is what allowed rates to be examined.
"We used HiFi long reads with over 99% accuracy, while they used common long reads with only ~85% Accuracy."." - please could you explain what "common long reads" are. PacBio HiFi sequencing is a frequently used long-read sequencing method so I'm not sure what the authors are referring to. To avoid ambiguity, please be specific. I am also unsure why technology itself makes a big difference - instead it's likely due to differences in average read length, is this the case?
The sentence on why Mantodea occupies "an important position in the evolution of insects" (line 280) still needs to be clearer. Please elaborate.
This needs further detail e.g. is 4,014 referring to the number of single-copy OGs in all mantises and outgroups? "From gene family clustering, 69,603 orthologous groups (OGs) were generated, including 4,014 single-copy OGs."
The description of the radar chart in figure 2A is still insufficient. Please explicitly state what a higher

vertex means and the corresponding percentages - e.g. are these the percentage of genome occupied by TEs, or total percentage of TEs corresponding to a particular TE type?
Figure 3B: The colours are still not correctly tracking between the chromosomes of each species. E.g. chr 1 of T. sinensis is pink to me while the homologous chromosomes in D. truncata is orange. This should be fixed. The legend describes "grep color". I'm not familiar with what this is. The legend also has an unclear sentence "Much more chromosome rearrangements were observed between M. violacea and the other mantises". Please revise this to make the message clearer.
Figure 4: The y-axis is just 'percentage'. Please elaborate what the percentage is of.
The figure title for Figure 4 "Evolution of Mantodea lineages" is very vague, please make it specific such that it summarises the key message of the figure.
With regards to adding the identified sex chromosomes and the sex that the genome is derived from to Table 1. I understand that the authors do not add more rows to Table 1. suggest instead adding this information within the existing rows e.g. "# of assembly-inferred chromosomes" could be 13A + 1Z rather than 14, and adding the sex sequenced in brackets next to the species name in the header row, thus conveying this information without adding more rows.
Figure S11: "The topology of NJ phylogeny tree is largely consistent the ML (maximum likelihood) tree in Figure 4B.". The topology appears the same to me between these two figures. Suggest removing this figure and just saying that the same topology was obtained by NJ in the main text as the result is the same.
Typos and suggested rephrasing:
Line 152: I do not think it's typical to put the taxonomic levels in brackets next to each taxon. Perhaps rephrase this e.g. "Currently the orders of Mantodea and Blattodea are placed within the superorder Dictyoptera"
Line 122: typo - "manily" â†' "mainly"
"using reciprocal-best orthologous genes" - should this be "reciprocal best hit". Please change in all instances.
Line 500: Suggest changing "4 chromosome brokens" to "4 chromosome breaks"
Ideally a single textface should be used in figures. Some figures use multiple text faces.

Methods

Are the methods appropriate to the aims of the study, are they well described, and are necessary controls included? Choose an item.

Conclusions

Are the conclusions adequately supported by the data shown? Choose an item.

Reporting Standards

Does the manuscript adhere to the journal's guidelines on minimum standards of reporting? Choose an item.

Choose an item.

Statistics

Are you able to assess all statistics in the manuscript, including the appropriateness of statistical tests used? Choose an item.

Quality of Written English

Please indicate the quality of language in the manuscript: Choose an item.

Declaration of Competing Interests

Please complete a declaration of competing interests, considering the following questions:

- Have you in the past five years received reimbursements, fees, funding, or salary from an organisation that may in any way gain or lose financially from the publication of this manuscript, either now or in the future?
- Do you hold any stocks or shares in an organisation that may in any way gain or lose financially from the publication of this manuscript, either now or in the future?
- Do you hold or are you currently applying for any patents relating to the content of the manuscript?
- Have you received reimbursements, fees, funding, or salary from an organization that holds or has applied for patents relating to the content of the manuscript?
- Do you have any other financial competing interests?
- Do you have any non-financial competing interests in relation to this paper?

If you can answer no to all of the above, write 'I declare that I have no competing interests' below. If your reply is yes to any, please give details below.

I declare that I have no competing interests

I agree to the open peer review policy of the journal. I understand that my name will be included on my report to the authors and, if the manuscript is accepted for publication, my named report including any attachments I upload will be posted on the website along with the authors' responses. I agree for my report to be made available under an Open Access Creative Commons CC-BY license (http://creativecommons.org/licenses/by/4.0/). I understand that any comments which I do not wish to be included in my named report can be included as confidential comments to the editors, which will not be published.

Choose an item.

To further support our reviewers, we have joined with Publons, where you can gain additional credit to further highlight your hard work (see: https://publons.com/journal/530/gigascience). On publication of this paper, your review will be automatically added to Publons, you can then choose whether or not to claim your Publons credit. I understand this statement.

Yes Choose an item.
